# The tumor immune microenvironmental analysis of 2,033 transcriptomes across 7 cancer types

**DOI:** 10.1038/s41598-020-66449-0

**Published:** 2020-06-12

**Authors:** Sungjae Kim, Ahreum Kim, Jong-Yeon Shin, Jeong-Sun Seo

**Affiliations:** 1 Precision Medicine Center, Seoul National University Bundang Hospital, Seongnam, 13605 Republic of Korea; 2 Precision Medicine Institute, Macrogen Inc., Seongnam, 13605 Republic of Korea; 3 Department of Biomedical Sciences, Seoul National University Graduate School, Seoul, 03080 Republic of Korea; 4 CHA University School of Medicine, Seongnam, 13488 Republic of Korea; 5 Gong-Wu Genomic Medicine Institute, Seoul National University Bundang Hospital, Seongnam, 13605 Republic of Korea

**Keywords:** Cancer genomics, Genome informatics, Immunogenetics, Immunotherapy, Tumour immunology

## Abstract

Understanding the tumor microenvironment is important to efficiently identify appropriate patients for immunotherapies in a variety of cancers. Here, we presented the tumor microenvironmental analysis of 2,033 cancer samples across 7 cancer types: colon adenocarcinoma, skin cutaneous melanoma, kidney renal papillary cell carcinoma, sarcoma, pancreatic adenocarcinoma, glioblastoma multiforme, and pheochromocytoma / paraganglioma from The Cancer Genome Atlas cohort. Unsupervised hierarchical clustering based on the gene expression profiles separated the cancer samples into two distinct clusters, and characterized those into immune-competent and immune-deficient subtypes using the estimated abundances of infiltrated immune and stromal cells. We demonstrated differential tumor microenvironmental characteristics of immune-competent subtypes across 7 cancer types, particularly immunosuppressive tumor microenvironment features in kidney renal papillary cell carcinoma with significant poorer survival rates and immune-supportive features in sarcoma and skin cutaneous melanoma. Additionally, differential genomic instability patterns between the subtypes were found across the cancer types, and discovered that immune-competent subtypes in most of cancer types had significantly higher immune checkpoint gene expressions. Overall, this study suggests that our subtyping approach based on transcriptomic data could contribute to precise prediction of immune checkpoint inhibitor responses in a wide range of cancer types.

## Introduction

The tumor microenvironment (TME) is composed of many different types of cells such as fibroblasts and myofibroblasts, neuroendocrine cells, extracellular matrix, stromal cells, and immune cells^1^. As TME significantly contributes to the cancer development and malignancy^2^, understanding of TME is important. The paradigm of clinical cancer treatment has been shifted towards the use of immune checkpoint inhibitor (ICI) treatments, which target T cell inhibitory receptors^3^. The ICIs have shown promising clinical effects in several types of cancer, especially in non-small-cell lung cancer (NSCLC)^4^. However, most of the patients in different types of cancer still show non-responsiveness to the treatment, and instead suffer intolerable side effects^5,6^. The predictive and prognostic biomarkers for ICIs have been developed by estimating the expression levels of immune checkpoint genes including *PD-1*, *PD-L1* and *CTLA4* as well as mutational burden in cancer samples, but the heterogeneity of tumor microenvironment around tumor cells was not considered^7^. In addition, the expressions of immune checkpoint genes and mutational burden are not sufficient to select the adequate patients and predict the responses to ICIs in several cancer types^8,9^.

The classifications of immunological associated subtypes in cancer have demonstrated its clinical significance as prognostic and predictive factors that could be used for a personalized cancer immunotherapy^10–12^. For instance, enhanced cytolytic immune functions in infiltrating lymphocytes CD8 T cells improved efficacy of immunotherapy^5,6^, and the relative contribution of each immune cells was considered to estimate the anti-tumor response^13,14^. Since immunosuppression from abnormalities of the TME critically interrupts immunotherapeutic approaches, understanding the TME and characterizing novel immune subtypes have been extensively researched to predict immunotherapy responses and enhance antitumor activity by targeting TME-induced ICI resistance^15,16^.

Here, we provide tumor microenvironmental analysis across 2,033 individuals in 7 cancer types from The Cancer Genome Atlas (TCGA) using our developed transcriptomic approach. The purpose of this extensive analysis is to elucidate the immunological characteristics and its association between cancer and TME in different types of cancer and to suggest potential stratification tool for ICI response prediction.

### TCGA abbreviations

BLCA; Bladder urothelial carcinoma, BRCA; Breast invasive carcinoma, CESC; Cervical squamous cell carcinoma and endocervical adenocarcinoma, CHOL; Cholangiocarcinoma, COAD; Colon adenocarcinoma, ESCA; Esophageal carcinoma, GBM; Glioblastoma multiforme, HNSC; Head and Neck squamous cell carcinoma, KICH; Kidney chromophobe, KIRC; Kidney renal clear cell carcinoma, KIRP; Kidney renal papillary cell carcinoma, LIHC; Liver hepatocellular carcinoma, PAAD; Pancreatic adenocarcinoma, PCPG; Pheochromocytoma and paraganglioma, PRAD; Prostate adenocarcinoma, READ; Rectum adenocarcinoma, SARC; Sarcoma, SKCM; Skin cutaneous melanoma, STAD; Stomach adenocarcinoma, THCA; Thyroid carcinoma, THYM; Thymoma, UCEC; Uterine corpus endometrial carcinoma.

## Results

### Unsupervised hierarchical clustering and immune characterization using TME scores separated 2,033 cancer samples into TME-related immune subtypes of 7 cancer types from TCGA cohorts

We conducted unsupervised hierarchical clustering of 7,762 cancer samples and 622 non-cancer controls across 22 cancer types using gene expression data. Among these cancer types, non-cancer controls in BLCA, BRCA, CESC, ESCA, HNSC, KIRC, PRAD, STAD, THCA, THYM and UCEC were separated into 2 or 3 clusters simultaneously along with cancer samples, which indicated that clusters cannot be defined into cancer-specific subtypes. Additionally, there was only one cancer sample at one of the clusters in READ. We thus excluded these 12 cancer types that were not clearly differentiated, and identified that 2,508 cancer samples in 10 cancer types were clearly separated into subtypes by the clustering.

**Figure 1:**
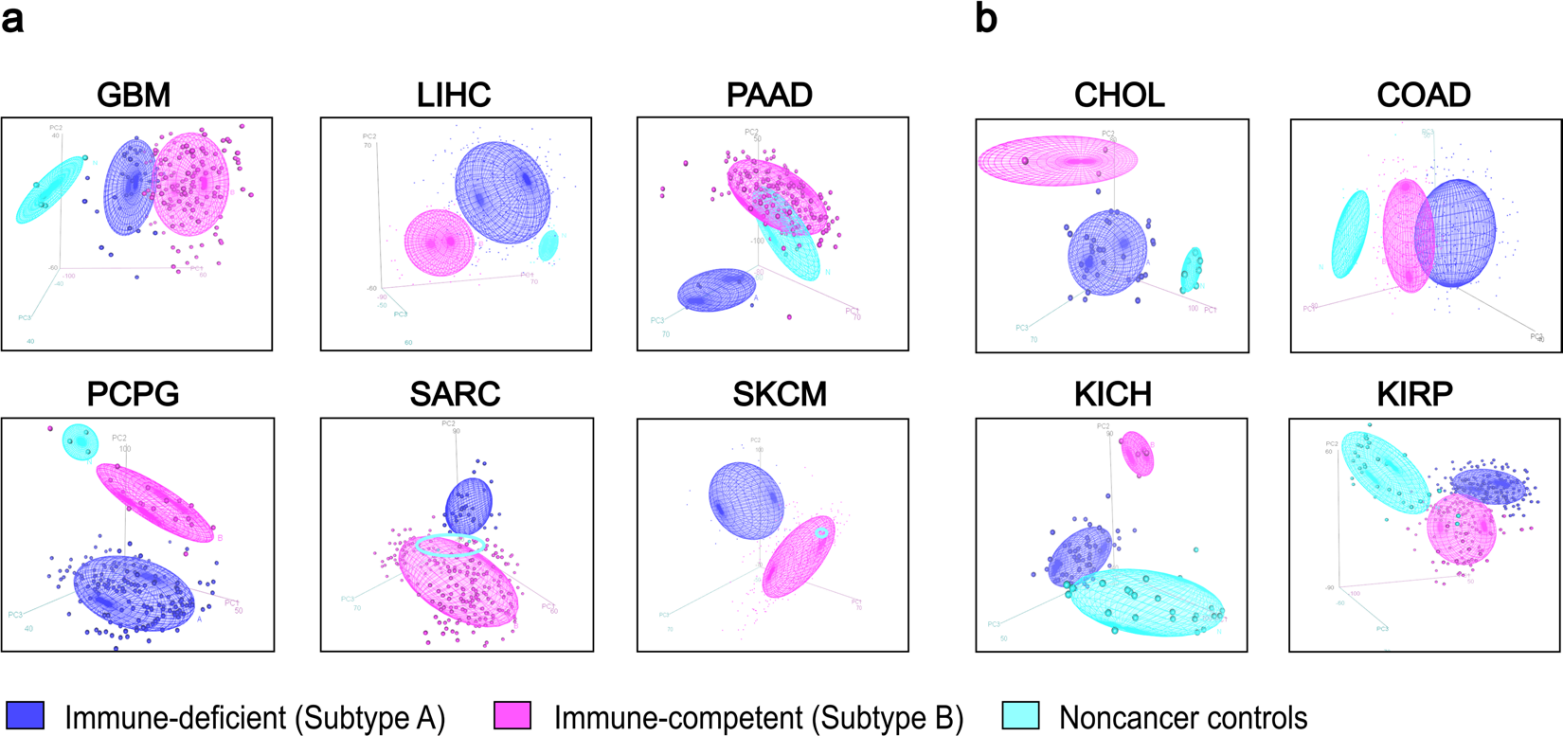
Unsupervised hierarchical clustering separated cancer samples into two distinctive clusters. PCA at different number of clusters. In the plots, blue, purple and cyan represents immune-deficient (subtype A), immune-competent (subtype B) and non-cancer controls, respectively. (**a**), At k = 2. (**b**), At *k* = 3.

The subtyping approach distinguished samples in 6 cancer types at *k* = 2 and 4 types at *k* = 3 via additional clustering. The principal component analysis (PCA) represented clustering patterns between the immune subtypes of cancer samples and non-cancer controls. At *k* = 2, immune-competent subtypes were closely clustered with non-cancer controls in PAAD, PCPG, SARC and SKCM while immune-deficient subtypes were closely clustered with controls in GBM and LICH (Fig. 1a). At *k* = 3, non-cancer controls, subtype A and B were distinctly clustered in CHOL, COAD, KICH and KIRP (Fig. 1b). Cluster dendrograms further demonstrated differential clustering patterns in 10 cancer types (Supplementary Fig. 1). Gene set enrichment analysis of the 1,000 most variables genes between cancer and controls for clustering in 10 cancer types demonstrated these genes overlapped with at least one immune-related gene set except KICH (Supplementary Table 2).

The subtypes of 2,508 cancer samples were defined by comparing their tumor microenvironmental (TME) characteristics based on the estimated abundance of infiltrating immune and stromal cells in tumor tissues, called immune and stromal scores, respectively, from Estimation of STromal and Immune cells in MAlignant Tumor tissues using Expression data (ESTIMATE)^13^. Samples with relatively lower mean scores were classified as immune-deficient subtype (subtype A) and those with relatively higher mean scores as immune-competent subtype (subtype B).

**Figure 2:**
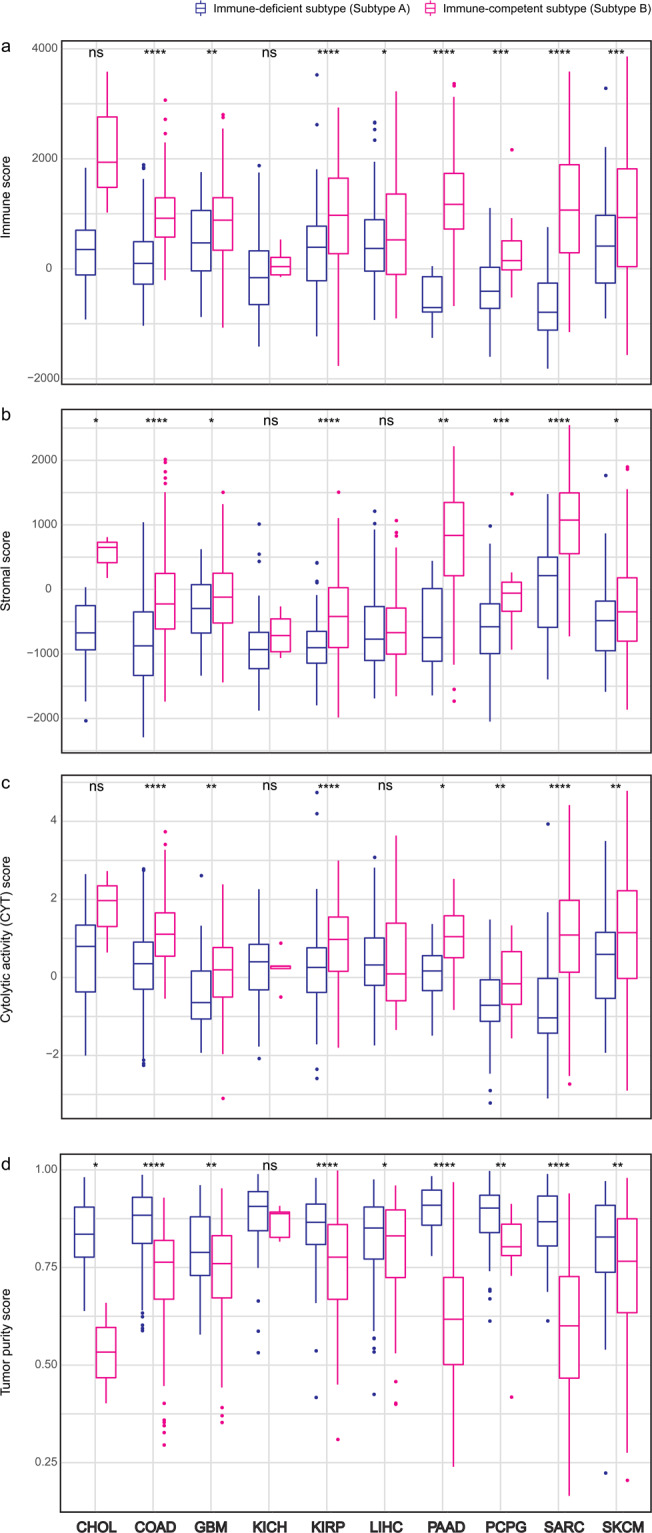
Comparison of predicted tumor microenvironmental related scores between immune subtypes across 10 cancer types. (**a**), Immune score. (**b**), Stromal score. (**c**), Tumor purity. (**d**), Cytolytic activity (CYT) score. The level of significance denoted as: ns., non-significant, *p < 0.05, **p < 0.01, ***p < 0.001 and ****p < 0.0001. Statistical significances between subtypes were measured by unpaired Student *t* test.

To verify the significance of TME characteristics in immune-competent subtypes across 10 cancer types, we compared immune and stromal scores using ESTIMATE, and estimated levels of cytolytic activity (CYT) scores using Tumor IMmune Estimation Resource (TIMER)^14^. Also tumor purity scores were compared and calculated using ESTIMATE, which showed strong correlations with the purity scores inferred by ABSOLUTE algorithm using copy number alteration (CNA) and somatic mutation data^17^ (Supplementary Fig. 2). Immune-competent subtypes in 10 cancer types showed enriched immune, stromal, CYT and lower tumor purity scores (Fig. 2a–d, respectively). We discovered significant differences in these well-established TME predictors between the immune subtypes in COAD, GBM, KIRP, PAAD, PCPG, SARC and SKCM. Although correlation between tumor purity data from ESTIMATE and ABSOLUTE was poor in PAAD (R2 = 0.16 and P = 5.08 × 10-2; Supplementary Fig. 2), we included this cancer for further analysis due to strong evidences of low tumor purity: significantly elevated immune, stromal and CYT scores.

In summary, we carried out unsupervised hierarchical clustering on 7,762 samples in 22 cancer types, and 11 cancers that were not clearly differentiated were excluded. A total of 2,675 samples in 11 cancer types were clearly differentiated at *k* = 2 and *k* = 3. Among these clearly differentiated cancer types, READ was excluded due to dramatically unbalanced distribution of the samples between subtypes, and we also excluded CHOL, KICH and LIHC based on criteria for TME characteristics using four estimated scores. Therefore, a total of 2,033 samples in 7 cancer types were selected and analyzed for further investigation (Supplementary Fig. 3.). Among these samples, 728 cancer samples were identified as immune-deficient subtypes and 1,305 samples as immune-competent subtypes in 7 cancer types (Table 1).

**Table 1:** Number of samples that are distinguished by unsupervised hierarchical clustering in 7 cancer types.

TCGA Cancer Type	Immune-deficient subtype (Subtype A)	Immune-competent subtype (Subtype B)	Non-cancer Controls	Total
Colon adenocarcinoma (COAD)	264	216	41	521
Glioblastoma multiforme (GBM)	37	132	5	174
Kidney renal papillary cell carcinoma (KIRP)	172	117	32	321
Pancreatic adenocarcinoma (PAAD)	8	170	4	182
Pheochromocytoma and paraganglioma (PCPG)	164	19	3	186
Sarcoma (SARC)	29	234	2	265
Skin cutaneous melanoma (SKCM)	54	417	1	472
Total	728	1,305	88	2,121

### Immune-competent subtypes in 7 cancer types demonstrated differential TME characteristics

**Figure 3:**
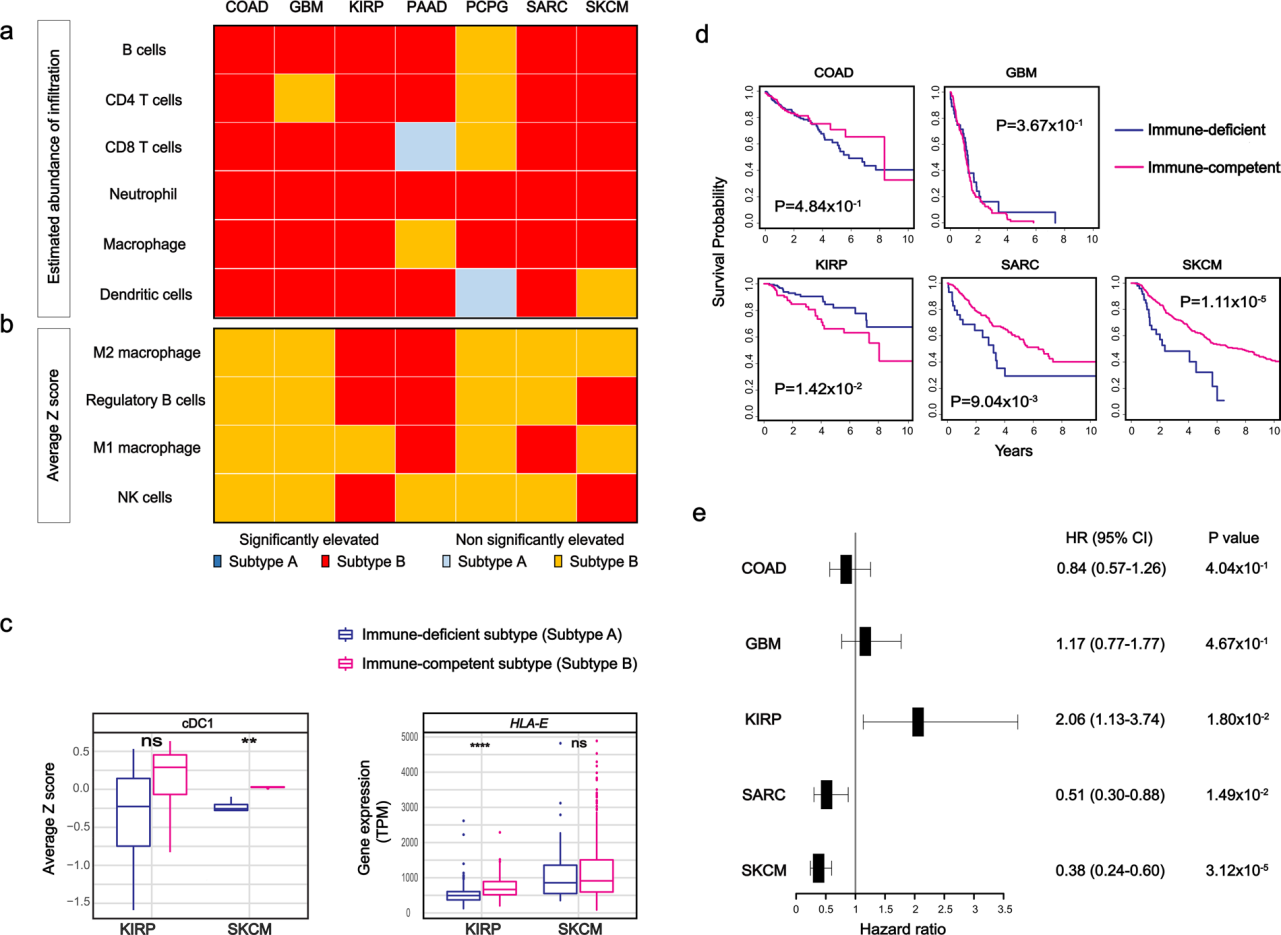
Estimated infiltration of immune cells, transcriptomic signatures for tumor microenvironment related immune cells and survival analysis of 7 cancer types. (**a**) Diagram of the estimated abundance of B cells, CD4^+^ T cells, CD8^+^ T cells, neutrophils, macrophages and dendritic cells inferred by TIMER were compared between the subtypes. Statistical significance was measured by Student *t* test. (**b**) Diagram showing the status of elevated expression of signature genes for M1 macrophage, M2 macrophage, regulatory B cell, and NK cell in immune-competent subtypes across 7 cancer types using the average z-scores of the genes. For subtype B, yellow color and red color squares represent elevation without and with statistical significance, respectively. For subtype A, blue color and sky blue color squares, respectively. Statistical significances between subtypes were measured by unpaired Student *t* test. (**c**) Expression pattern of NK antitumor activities in KIRP and SKCM. Average z-score for cDC1 and gene expression in TPM between the subtypes in KIRP and SKCM. ns., non-significant, *p < 0.05, **p < 0.01, ***p < 0.001 and ****p < 0.0001. Statistical significances between subtypes were measured by unpaired Student *t* test. d, Kaplan-Meier survival curves of COAD, GBM, KIRP, SARC and SKCM. Blue lines represent immune-deficient and magenta lines for immune-competent subtypes. Statistical significance was measured by log rank test. (**e**) Hazard ratio between subtypes by univariate Cox regression. Forest plot illustrates hazard ratio and 95% confidence intervals in COAD, GBM, KIRP, SARC and SKCM. Positive rates represent that subtype B is negatively associated with survival and negative rates represent that subtype A is negatively associated with survival.

To further elucidate TME features of immune-competent subtypes in 7 different types of cancer, we compared the estimated abundances of infiltrated immune cells, including B cells, CD4^+^ T cells, CD8^+^ T cells, neutrophils, macrophages and dendritic cells (DC) from TIMER, which were appropriate for inter-sample comparison and validated using multiple approaches^14,18^. We found that most of these immune cells were significantly infiltrated in the TME of immune-competent subtypes across 7 cancer types (Fig. 3a).

Specifically, M1 macrophages, M2 macrophages and regulatory B (B_reg_) cells were known to show differential effects on the TME: M2 macrophages and B_reg_ were involved in immune evasion, and reduced the sensitivity to immune-checkpoint inhibitors, while M1 macrophages along with CD8^+^ T cells and DC played roles in anti-tumor activity^19,20^. Therefore, we investigated average z scores of signature gene expressions for M1 macrophages, M2 macrophages^21,22^, and B_reg_ cell infiltrations that were experimentally validated^23,24^ between the subtypes in this study. As immune-competent subtypes in 7 cancers demonstrated relatively increased abundance of infiltrated immune cells, the expressions of signature genes for M1 macrophages, M2 macrophages and B_reg_ cells were also elevated in these TME highlighted subtypes. The signature genes for both M2 macrophage and B_reg_ were significantly elevated in immune-competent subtypes of KIRP and PAAD among 7 types of cancer and only B_reg_ cells in immune-competent subtypes of SKCM. While the signature genes for M1 macrophages were significantly elevated in immune-competent subtypes of PAAD and SARC (Fig. 3b). We also compared several immune-related molecules that were up-regulated within high cytolytic activity subtypes in colon cancer^25^ for clarification of TME within our immune-competent subtypes. The majority of expressions of immune-related genes were also elevated in immune-competent subtypes across 7 cancers which clearly indicated high proportion of immune molecules within these subtypes (Supplementary Fig. 4).

In addition to signature gene expressions for these immune cells and molecules, we compared the expression levels to estimate the abundance of NK cells^26^, which were well known to be involved in anti-tumor effects in the TME^27,28^. We discovered that immune-competent subtypes showed significantly elevated expressions of signature genes for NK cell infiltration in both KIRP and SKCM among 7 cancers (Fig. 3b). We further analyzed detailed transcriptomic signatures of anti-tumor effects induced by NK cells within two types of cancer: KIRP and SKCM as immune-competent subtypes in these cancer types showed significant elevation of genes related to NK cell infiltration compared to the other cancer types. Recruiting conventional type 1 dendritic cells (cDC1) into the TME by NK cells induced anti-tumor effects^29^. Interestingly, we found that the expressions of signature genes for cDC1 were only significant in SKCM (P = 9.60 × 10-3; Fig. 3c). In contrast, only immune-competent subtypes in KIRP had significantly increased expression of HLA class 1 molecules antigen E (*HLA-E*), which suppressed NK cell activity^30^ (P = 4.76 × 10-5; Fig. 3c). At gene levels, immune-competent subtypes in KIRP and immune-deficient subtypes in SKCM showed decreased expression of C-type lectin domain containing 9 A (*CLEC9A*) (P = 4.82 × 10-1 and P = 4.08 × 10-3, respectively; Supplementary Fig. 5). The expressions of other markers for cDC1, including *XCR1*, *CLNK* and *BATF3* were increased in both immune-competent subtypes of SKCM and KIRP. We also investigated NK inhibitory receptors: *KLRC1* (*NKG2A*) and *KLRD1* (*CD94*) that suppressed NK cell anti-tumor activity via binding *HLA-E*^30^ to evaluate differential NK cell-mediated effects in KIRP and SKCM. However, they were increased in both immune-competent subtypes of KIRP and SKCM (Supplementary Fig. 5).

We conducted survival analysis between the subtypes across 7 cancer types to support immune characteristics of TME in the immune-competent subtypes and to evaluate clinical significance of immune subtyping. We excluded PAAD and PCPG for the survival analysis because of the following reasons: overall survival (OS) was used as an endpoint since it is recommended to be used for the majority of TCGA cancer types except PCPG due to insufficient follow-up time^31^, and all of immune-deficient subtypes were identified as alive in PAAD. Our survival analysis found that immune-competent subtypes had significantly poorer survival rates than immune-deficient subtypes in KIRP (P = 1.42 × 10-2; Fig. 3d). In contrast to KIRP, immune-deficient subtypes showed poorer survival rates than immune-competent subtypes in SARC and SKCM (P = 9.04 × 10-3 and P = 1.11 × 10-5, respectively; Fig. 3d). Unlike KIRP, SARC and SKCM, non-significant differences in survival rates between the subtypes in COAD and GBM were found (P = 4.84 × 10-1 and P = 3.67 × 10-1, respectively; Fig. 3d).

We also performed univariate Cox regression analysis to discover hazard ratio (HR) of immune-competent subtypes in 7 cancer types. In KIRP and GBM, immune-competent subtypes had HR of 2.06 and 1.17 (95% CI = 1.13–3.74 with P = 1.80 × 10^−2^ and 95% CI = 0.77–1.77 with P = 4.67 × 10^−1^; Fig. 3e), while HR of 0.84, 0.51 and 0.38 in SARC and SKCM, respectively (95% CI = 0.57–1.26 with P = 4.04 × 10^−1^, 95% CI = 0.30–0.88 with P = 1.49 × 10^−2^ and 95% CI = 0.24–0.60 with P = 3.12 × 10^−5^, respectively; Fig. 3e).

### Comparison of genomic instability and the immune checkpoint gene expressions between the subtypes in 7 cancer types

**Figure 4:**
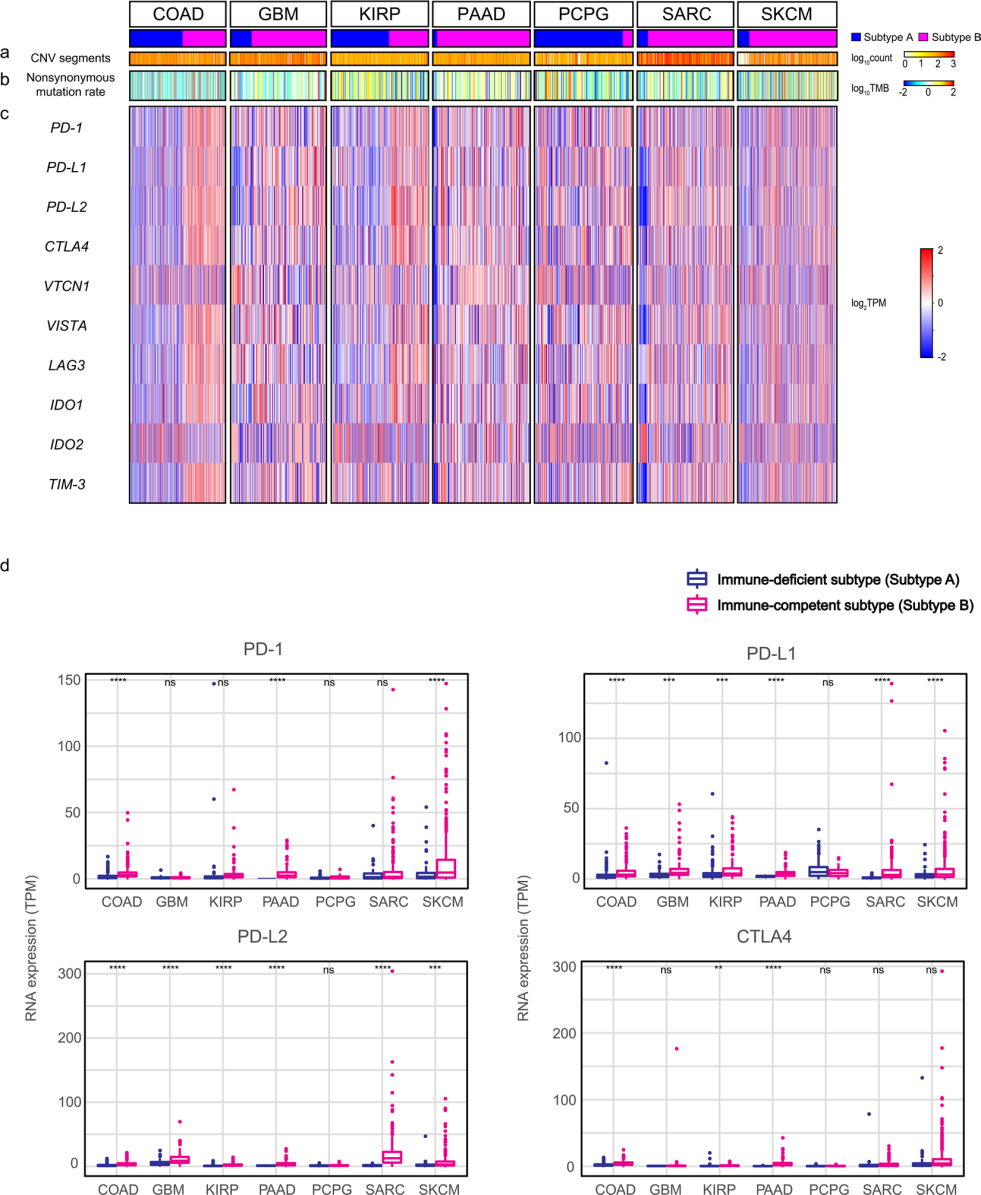
Comparison of the genomic instability scores and the expressions of immune checkpoint genes between immune subtypes in 7 cancer types. (**a**), Comparison of the sCNV burden, measured by the log_10_-transformed total number of segments in each sample’s copy number profile between the subtypes across 7 cancer types. (**b**), Comparison of the log_10_-transformed the number of nonsynonymous mutations per Mb in the genome between the subtypes across 7 cancer types. (**c**), Heatmaps of the log_2_-transformed expression levels (TPM) of 10 immune checkpoint genes between the subtypes across 7 cancer types. (**d**), RNA expressions (TPM) of four immune checkpoint genes in immune-deficient and immune-competent subtypes across 7 cancers. The level of significance denoted as: ns., non-significant, *p < 0.05, **p < 0.01, ***p < 0.001 and ****p < 0.0001. Statistical significances of the genomic instability scores and the expression of the genes between subtypes were measured by unpaired Student *t* test.

We illustrated the comparison of genomic instability status including somatic copy number variations (sCNV) and tumor mutation burden (TMB) between the subtypes across 7 cancer types. Number of sCNV segments in immune-deficient subtypes of COAD and PCPG (P = 6.40 × 10-2 and 2.20 × 10-1, respectively; Fig. 4a) was increased, which was consistent with previous results of TME highlighted subtypes in LUSC, COAD and READ^11,25^. In contrast, elevated segment number was observed in immune-competent subtypes of GBM, KIRP, PAAD, SARC and SKCM (P = 1.70 × 10-2, P = 1.20 × 10-1, P = 1.20 × 10-1, P = 4.80 × 10-1 and P = 6.40 × 10-1, respectively; Fig. 4a). We identified significantly amplified or deleted loci and genes within both subtypes across 7 cancers except few subtypes (Supplementary Table 3 and 4, respectively), and recurrent amplifications and deletions at several loci with immune checkpoint genes were found in KIRP, PAAD, PCPG, SARC and SKCM (Supplementary Fig. 6).

For nonsynonymous TMB, immune-deficient subtypes in GBM, KIRP, and PCPG (P = 3.50 × 10^−1^, P = 1.40 × 10^−2^, and P = 7.40 × 10^−3^, respectively; Fig. 4b) and immune-competent subtypes in COAD, PAAD, SARC and SKCM had increased non-silent mutation rates (P = 7.90 × 10^−2^, P = 2.50 × 10^−1^, P = 2.40 × 10^−3^, and P = 2.40 × 10^−4^, respectively; Fig. 4b). Along with TMB, we calculated SNV neoepitope loads. Predicted neoepitope loads were also elevated in immune-competent subtypes of COAD, PAAD, SARC and SKCM (P = 3.50 × 10^−2^, P = 7.60 × 10^−2^, P = 1.10 × 10^−2^, and P = 4.10 × 10^−2^; Supplementary Fig. 7), and immune-deficient subtypes of GBM, KIRP and PCPG (P = 7.20 × 10^−2^, 3.40 × 10^−2^, and 8.70 × 10^−2^; Supplementary Fig. 7). Additionally, we conducted correlation analysis between CYT scores and neoepitope loads within immune-deficient and immune-competent subtypes across 7 cancer types. We observed no differences in these correlation patterns between the immune subtypes, and most of cancer types showed weak correlations within both subtypes across 7 cancer types except COAD, which had noticeable correlations in both subtypes (R2 = 0.47 and P = 3.18 × 10^−13^ in immune-deficient subtypes and R2 = 0.51 and P = 1.60 × 10^−12^ in immune-competent subtypes; Supplementary Fig. 8) as previously reported^25^.

To investigate the potentials of immune-competent subtypes as efficient targets for immune checkpoint inhibitors, we compared the expressions of immune checkpoint genes: *PD-1*, *PD-L1*, *PD-L2* and *CTLA4* which are well-known targets of immune checkpoint inhibitors^3^ between the subtypes in 7 cancer types. Immune-competent subtypes in 7 cancers had increased expression of these genes compared to immune-deficient subtypes except *PD-1* expression in GBM and *PD-L1* in PCPG, and *CTLA4* in SARC. Particularly, the expressions of *PD-L1* and *PD-L2* in immune-competent subtypes were significantly elevated in 7 types except PCPG. In addition to those ligands, the expressions of *PD-1* were significantly increased in COAD, PAAD and SKCM and those of *CTLA4* in COAD, KIRP, PAAD and PCPG (Fig. 4c). There are several immune checkpoint genes including *VTCN1, VISTA, LAG3, IDO1, IDO2 and TIM3*^32–34^ that are emerging in the development of immunotherapy, and we compared the expressions of these genes between the immune subtypes. The patterns of differences between expressions across 7 cancers were inconsistent. The majority of immune-competent subtypes had increased expressions. However, immune-competent subtypes in several cancer types had relatively lower expressions than immune-deficient subtypes, particularly *VTCN1* in GBM and *LAG3* and *IDO1* in PCPG were significantly elevated in immune-deficient subtypes (Fig. 4c). Comparing absolute RNA expressions of four immune-checkpoint molecules across 7 cancers types also demonstrated elevated expressions in the immune-competent subtypes. Furthermore, in terms of cancer types, relatively higher expressions of *PD-1* and *CTLA4* in SARC and SKCM compared to the others, elevated expressions of *PD-L2* in GBM, SARC and SKCM, and similar levels of *PD-L1* expressions across cancer types were observed (Fig. 4d).

## Discussion

Predictive methods for ICI response including immunohistochemistry (IHC) of immune checkpoint genes and emerging prognostic markers using genomic data such as TMB and CNV burdens^35,36^ are conventionally used, but more precise estimations with non-complex approaches still remain to be investigated and developed. Here, we represent a computational strategy to potentially maximize choosing targets for ICIs based on only transcriptomic data that gives analytical advantages in measuring the expressions over IHC^37^.

Using unsupervised hierarchical clustering based on the gene expression, we presented the TME-dependent differentiation of cancer samples in 7 cancer types from TCGA. The identified immune-competent subtypes in these cancers showed TME signatures: significantly elevated immune and stromal cell infiltration and cytolytic activities, and lower tumor purity. The characteristics of TME in these subtypes were differentially identified using survival data along with the expressions of signature genes for several immune cells and molecules which were known to be involved in the TME. They often exhibit immunosuppressive characteristics that inhibit the functions of effector T cells, and reduce the efficacy of immunotherapy^38^. Interestingly from our results, the signatures for immunosuppressive TME were clearly represented in only KIRP, shown by significantly poorer OS with elevated expression for signature genes of M2 macrophage and B_reg_, and clear transcriptomic patterns of impaired NK cell induced anti-tumor activities.

Meanwhile, immune-competent subtypes showed significantly improved OS in SARC and SKCM. These subtypes in SARC had significantly increased signature gene expressions for M1 macrophage and estimated infiltration of CD8^+^ T cells and DC which are associated with anti-tumor activity^19,20^. In SKCM, we demonstrated that transcriptomic patterns in immune-competent subtypes potentially reflected anti-tumor activity from NK cell even though the expressions of signature genes for immunosuppressive B_reg_ were significantly increased in SKCM. The results from SARC and SKCM implied that infiltrated immune cells in TME were not associated with immune evasion, and possibly formed immune-supportive TME in immune competent subtypes.

We then evaluated genomic instabilities including sCNV burden, TMB, and neoepitope loads between the immune subtypes. Differences in number of CNV segments between subtypes in the majority of cancer types showed inconsistent results in sCNV with previous TME studies from LUSC and colorectal cancers (CRC)^11,25^. At gene levels, recurrent amplifications and deletions at immune checkpoint genes were found in several subtypes of cancers but not in TME-dependent manner. We also demonstrated that immune-competent subtypes had significantly increased TMB and neoepitope loads in SARC and SKCM. Subtypes in COAD also had increased these genomic instabilities without significance in TMB elevation, and correlation analysis suggested that neoepitope loads may play a role in CYT activity in both immune subtypes of COAD. In GBM, KIRP, and PCPG, immune-competent subtypes had lower TMB and neoepitope loads. Particularly, these subtypes in KIRP showed significant difference like LUSC^11^.

The fact that expressions of immune checkpoint genes were significantly elevated in the immune-competent subtypes and higher expressions in certain types of cancer could potentially suggest and optimize usage of ICIs. However, we need to compare these immune-related subtypes in terms of responses to ICIs, to evaluate the prognostic value of this immune subtyping in 7 cancer types in the future.

Since we applied a single subtyping approach to different types of cancer originated from different types of tissue, inconsistency in identifying the characteristics of TME in immune-competent subtypes across 7 cancers was discovered. Also M1 and M2 macrophages are not always associated with anti-tumor effect and tumor-associated macrophages, respectively^39^ as macrophage populations are tissue- and tumor-specific. Hence experimental validation of immune cell infiltration is needed to be conduct to clarify the TME characteristics for each cancer type in the future.

Although further studies are required on this approach to become clinically significant, our subtyping provides convenient prediction for identifying the prognostic and predictive factors that could guide personalized cancer immunotherapies using only transcriptomic data. We believe that considering the immune subtypes in a TME dependent manner will be a decent diagnostic biomarker and predictor for responses to immunotherapy.

## Materials and Methods

### The Cancer Genome Atlas (TCGA) data sets

RNA sequencing data in TCGA, which are composed of cancer and non-cancer control data were used for characterizing the tumor microenvironment and classifying immune subtypes in 22 cancer types. We excluded ACC, DLBC, LAML, LGG, MESO, OV, TGCT, UCS and UVM as there were no non-cancer controls in those types, and also excluded previously studied LUAD and LUSC. The raw reads of RNA expression datasets as htseq count format were obtained in the TCGA^40^. The list of TCGA cancer type abbreviations is available (see URL).

### Identification of immune subtypes

Immune subtypes of TCGA cancers were identified by unsupervised complete linkage clustering method, then we cut cluster trees into 2 and 3 groups to identify subtypes. Raw reads were processed and transformed to variance stabilizing data (VSD) using R package ‘DESeq2’^41^, then 1,000 most variable genes were used for subtype classification of cancer samples. The PCA was plotted using first three principal components with a 95% confidence interval.

### Processing RNA-seq based gene expression

For normalized RNA data, fragments per kilobase million (FPKM) was computed from the raw reads from HTseq counts by using the R package ’edgeR’^42^, and the FPKM expression values were adjusted to log2 and centered median expression values by cluster 3.0^43^. For comparing the expressions for immune checkpoint gene between the subtypes, log2 FPKM was used. We converted FPKM to transcripts per kilobase million (TPM) using the formula as described below^44^.

$$TPM=\frac{FPKM\,of\,gene\,A\,}{Sum\,of\,FPKMs\,of\,all\,genes}\,\ast \,{10}^{6}$$

### Estimated abundance of stromal and immune cells

The composition of stromal and immune cells (stromal, immune, and tumor purity) and CYT score in each tumor samples were estimated by ESTIMATE and TIMER algorithms^13,14^. The infiltrating immune cells (B cells, CD4 T cells, CD8 T cells, neutrophils, macrophages, and dendritic cells) were compared in each cancer type and subtype in TCGA cohorts. The previously validated immune type specific gene sets were used for identifying the impacts and roles of the immune cells on tumor microenvironment.

### Calculation of Z-scores

The Z-score of each signature gene was calculated as below.

$$Z\mbox{-}score=\frac{FPKM\,of\,tumor\,samples-mean\,FPKM\,of\,noncancer\,control\,samples\,}{Standard\,deviation\,of\,mean\,FPKM\,of\,noncancer\,control\,samples}$$

Since there was only one non-cancer control sample in SKCM, we calculated Z scores for SKCM using mean FPKM of cancer samples instead of values from non-cancer control samples.

### Scores for genomic instability

The mutation rates, scores for sCNV and neoantigen loads were derived from previous studies^45,46^. We excluded the samples without available genomic instability data for this analysis. Silent and non-silent mutation rates were the number of mutations divided by target length of the genome in Mb. In sCNV burden, ‘Number of segments’ equals to total number of segments in each sample’s copy number profiles.

### Identification of recurrent sCNV

The copy number SEG file of TCGA samples was downloaded^17^. GISTIC analysis^47^ was conducted to find recurrent amplification and deletion in the subtypes of 7 cancer types. The confidence level for the significant region was 0.90 and the q value was 0.25.

### Statistical analyses

R-3.3.0 was used for statistical analyses. In the case that the sample size was bigger than 30, we used the unpaired student’s t test for comparison between immune subtypes. The p-value for overall survival curve was compared by the log-rank test. The Cox hazardous ratio was estimated by using the R package ‘survminer’. The boxplot with the quantitative data was presented as mean ± standard deviation. For correlation, Pearson’s product-moment correlation was used.

### URLs

TCGA abbreviations list: https://gdc.cancer.gov/resources-tcga-users/tcga-code-tables/tcga-study-abbreviations

## Supplementary information

Supplementary information.
