# Supplementary material for: The tumor immune microenvironmental analysis of 2,033 transcriptomes across 7 cancer types
Source: Sci Rep. 2020 Jun 12;10:9536. doi: 10.1038/s41598-020-66449-0 (PMC7293350; doi:10.1038/s41598-020-66449-0)
Supplement: Supplementary file 1 — Supplementary information. [file 41598_2020_66449_MOESM1_ESM.pdf]

Supplementary Information

The tumor immune microenvironmental analysis of 2,033 transcriptomes across 7 cancer types

Sungjae Kim[1,2,3,6], Ahreum Kim[4,6], Jong-Yeon Shin[2], Jeong-Sun Seo[1,2,3,5]

[1]Precision Medicine Center, Seoul National University Bundang Hospital, Seongnam, 13605, Republic of Korea

[2]Precision Medicine Institute, Macrogen Inc., Seongnam, 13605, Republic of Korea

[3]Department of Biomedical Sciences, Seoul National University Graduate School, Seoul, 03080, Republic of Korea

[4]CHA University School of Medicine, Seongnam, 13488, Republic of Korea

[5]Gong-Wu Genomic Medicine Institute, Seoul National University Bundang Hospital, Seongnam, 13605, Republic of Korea

[6]These authors contributed equally to this work

Corresponding author:

Jeong-Sun Seo, MD, PhD

Precision Medicine Center,

Seoul National University Bundang Hospital,

172 Dolma-ro, Seongnam, Bundang-gu, Gyeonggi-do 13605, Republic of Korea,

Tel: +82-10-8963-8246; Fax: +82-31-600-3011; E-mail: jeongsunseo@gmail.com

Supplementary Figures

Supplementary Fig 1. Dendrogram plots of 10 cancer types at *k=2* and *k=3*.

Supplementary Fig 2. Correlation plots between purity inferred by ESTIMATE and ABSOLUTE of 7 cancer types. The x-axis corresponds to the purity data from ESTIMATE (A) and y-axis to the data from ABSOLUTE (B). Correlation coefficient and p-values were calculated using Pearson's product-moment correlation.

Supplementary Fig 3. Sample inclusion flow chart.

Supplementary Fig 4. Diagram showing the status of elevated expression of several immune molecules. For subtype B, yellow color and red color squares represent elevation without and with statistical significance, respectively. For subtype A, blue color and sky blue color squares, respectively. Statistical significances between subtypes were measured by unpaired Student t test.

Supplementary Fig 5. The expression profile of signature genes for cDC1 and NK cell suppression in KIRP and SKCM. The expression heatmap between the immune subtypes. **a,** in KIRP. **b,** in SKCM. Statistical significance was measured by Student *t* test.

Supplementary Fig 6. Significantly amplified (red) and deleted (blue) regions in immune-deficient (subtype A) and immune-competent (subtype B) across 7 cancer types. Immune checkpoint genes are presented at the loci if included.

Supplementary Fig 7. Predicted neoepitope loads between the subtypes across 7 cancer types.

Supplementary Fig 8. Correlation plots between predicted SNV neoantigen loads and cytolytic (CYT) scores of immune-deficient subtypes (left) and immune-competent subtypes (right) across 7 cancer types.

Supplementary Tables

Supplementary Table 1. Top 1,000 most variable genes used for unsupervised hierarchical clustering in 10 cancer types

Supplementary Table 2. Gene set enrichment analysis results of top 1,000 most variable genes used for unsupervised hierarchical clustering in 10 cancer types

Supplementary Table 3. Significantly amplified and deleted loci across 7 cancer types

Supplementary Table 4. Significantly amplified and deleted genes across 7 cancer types

Supplementary Table 5. Immune subtypes in 7 cancer types

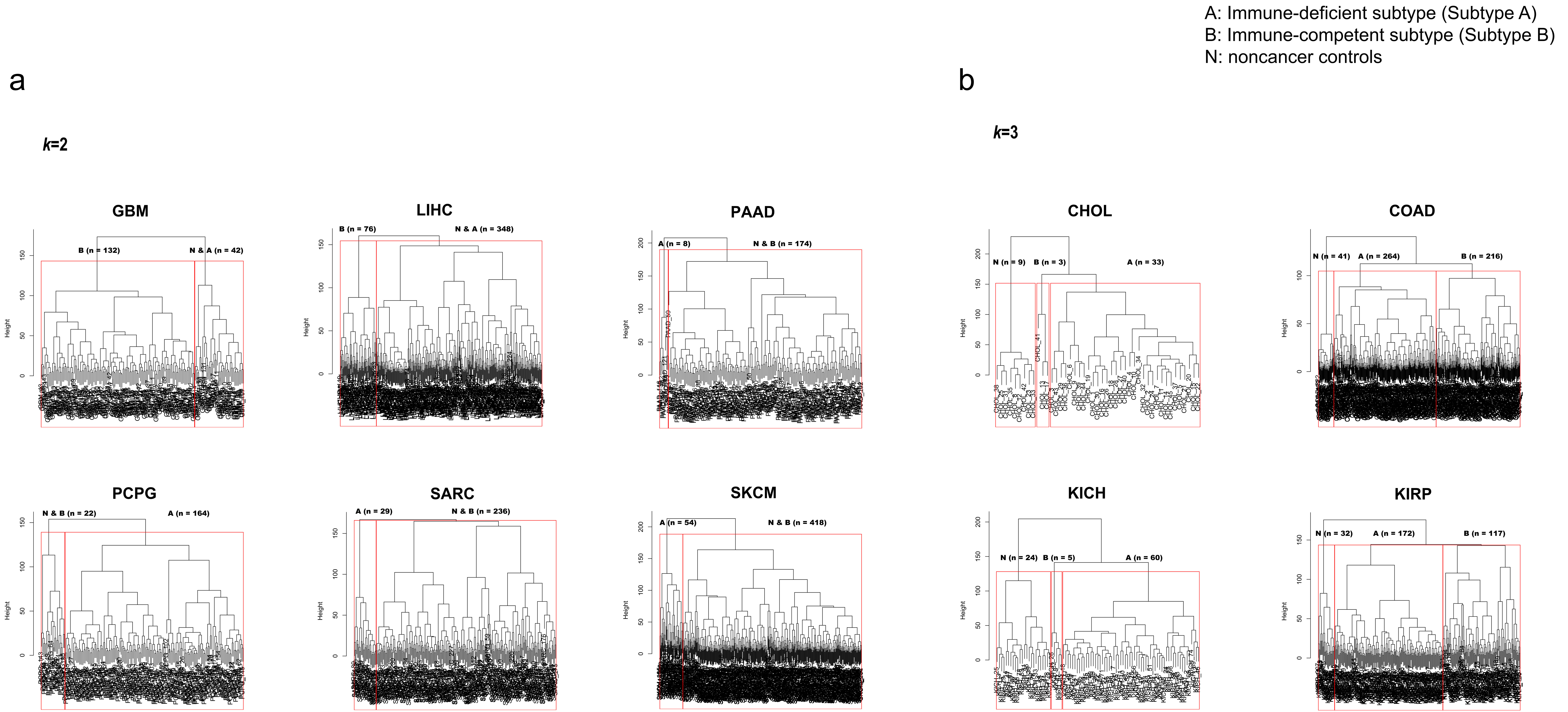
A: Immune-deficient subtype (Subtype A)
B: Immune-competent subtype (Subtype B)
N: noncancer controls
a
k=2
GBM
B (n = 132)
N & A (n = 42)
LIHC
B (n = 76)
N & A (n = 348)
PAAD
A (n = 8)
N & B (n = 174)
PCPG
N & B (n = 22)
A (n = 164)
SARC
A (n = 29)
N & B (n = 236)
SKCM
A (n = 54)
N & B (n = 418)
b
k=3
CHOL
N (n = 9)
B (n = 3)
A (n = 33)
COAD
N (n = 41)
A (n = 264)
B (n = 216)
KICH
N (n = 24)
B (n = 5)
A (n = 60)
KIRP
N (n = 32)
A (n = 172)
B (n = 117)
Height

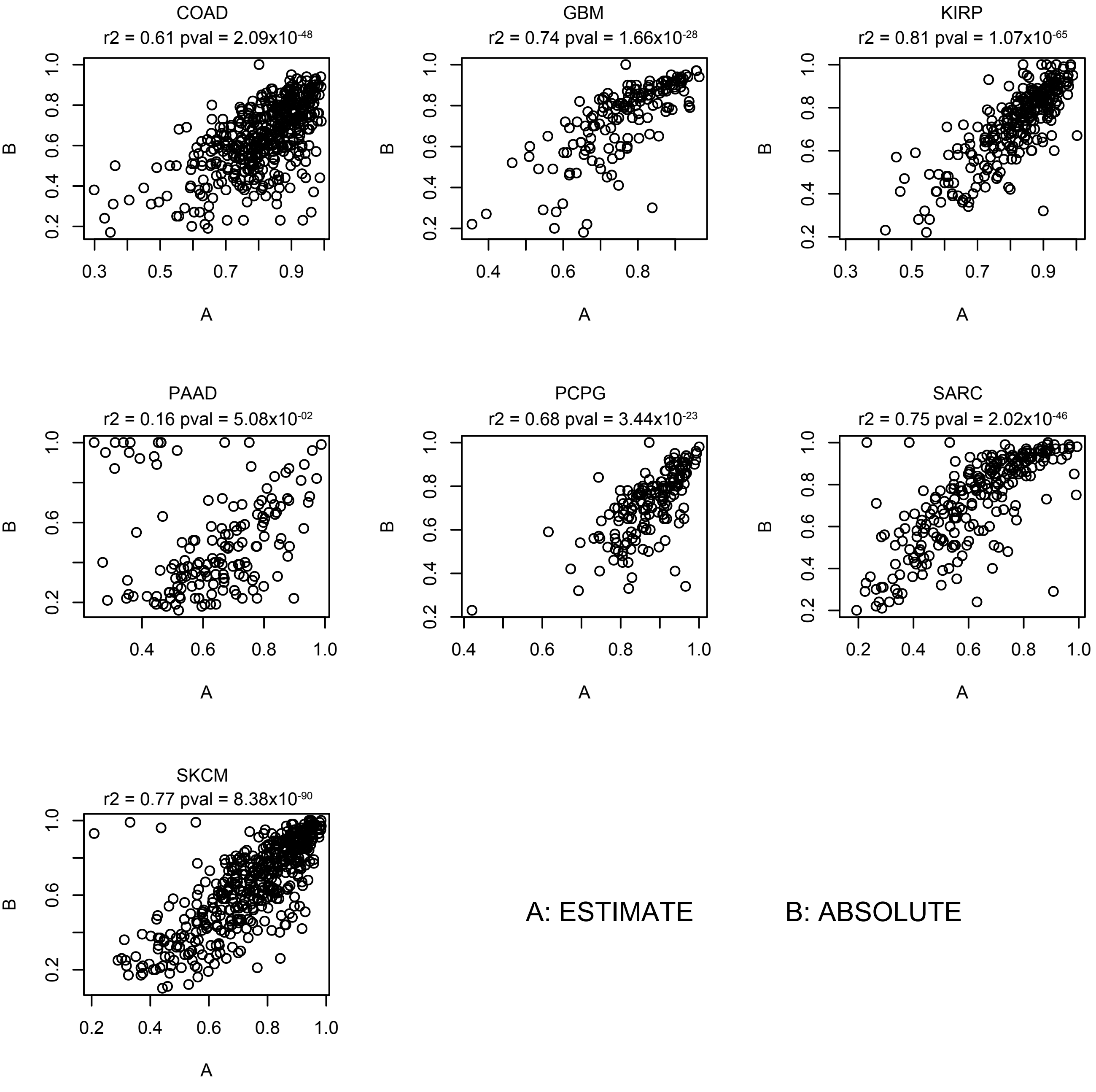
COAD
r2 = 0.61 pval = 2.09x10-48
GBM
r2 = 0.74 pval = 1.66x10-28
KIRP
r2 = 0.81 pval = 1.07x10-65
PAAD
r2 = 0.16 pval = 5.08x10-02
PCPG
r2 = 0.68 pval = 3.44x10-23
SARC
r2 = 0.75 pval = 2.02x10-46
SKCM
r2 = 0.77 pval = 8.38x10-90
A
B
A: ESTIMATE
B: ABSOLUTE

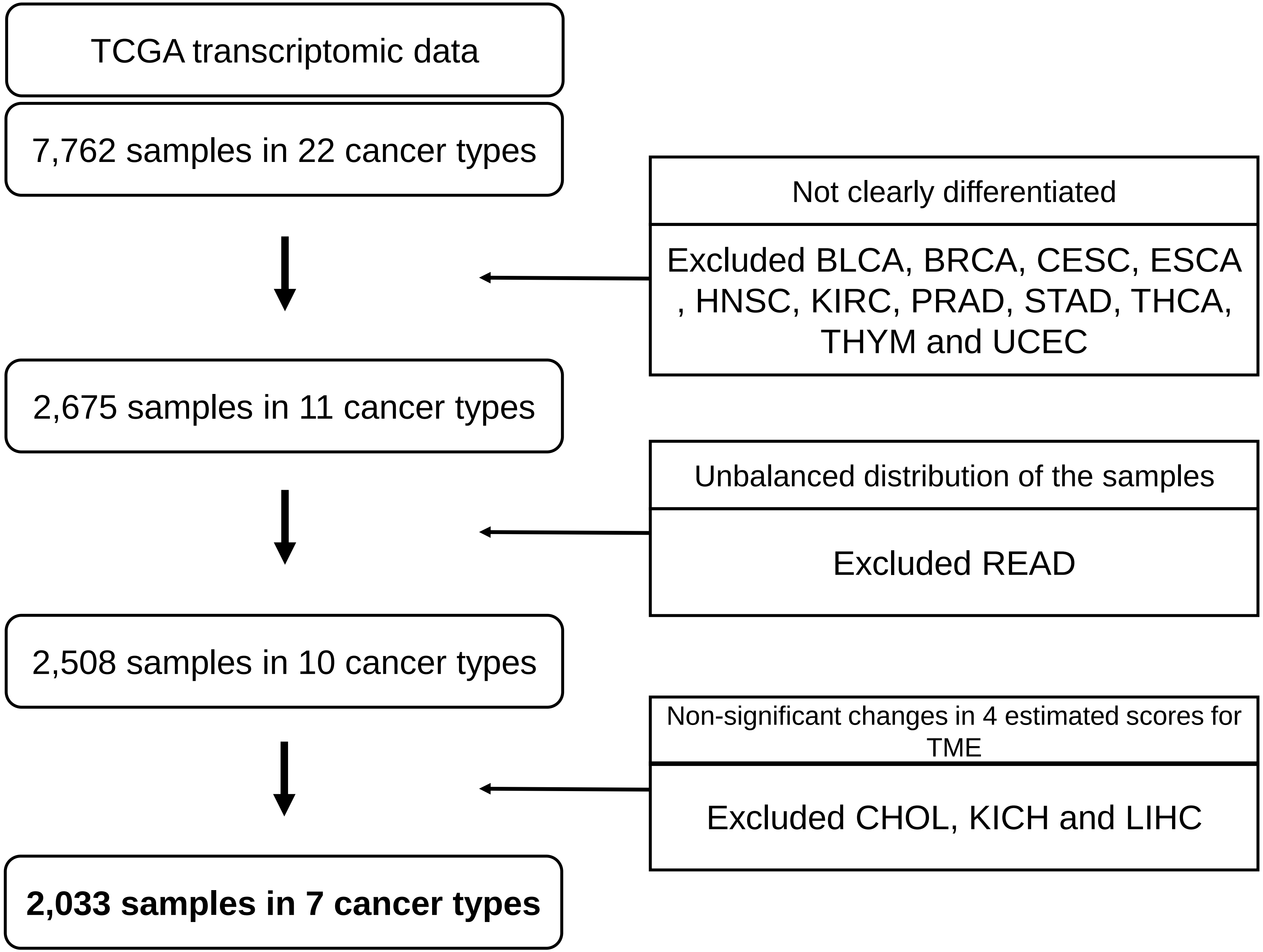
TCGA transcriptomic data
7,762 samples in 22 cancer types
Not clearly differentiated
Excluded BLCA, BRCA, CESC, ESCA , HNSC, KIRC, PRAD, STAD, THCA, THYM and UCEC
2,675 samples in 11 cancer types
Unbalanced distribution of the samples
Excluded READ
2,508 samples in 10 cancer types
Non-significant changes in 4 estimated scores for TME
Excluded CHOL, KICH and LIHC
2,033 samples in 7 cancer types

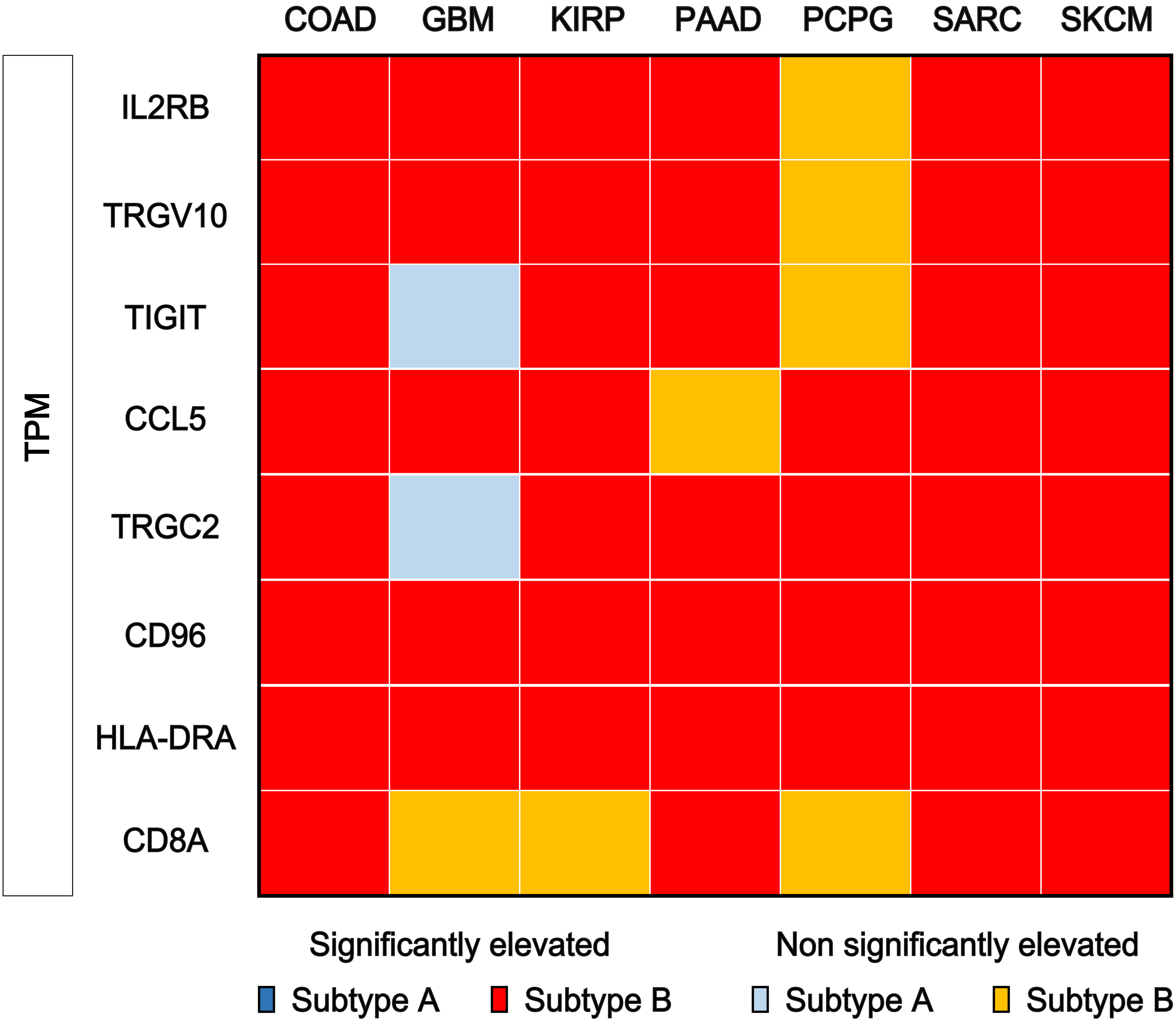
COAD
GBM
KIRP
PAAD
PCPG
SARC
SKCM
TPM
IL2RB
TRGV10
TIGIT
CCL5
TRGC2
CD96
HLA-DRA
CD8A
Significantly elevated
Non significantly elevated
Subtype A
Subtype B
Subtype A
Subtype B

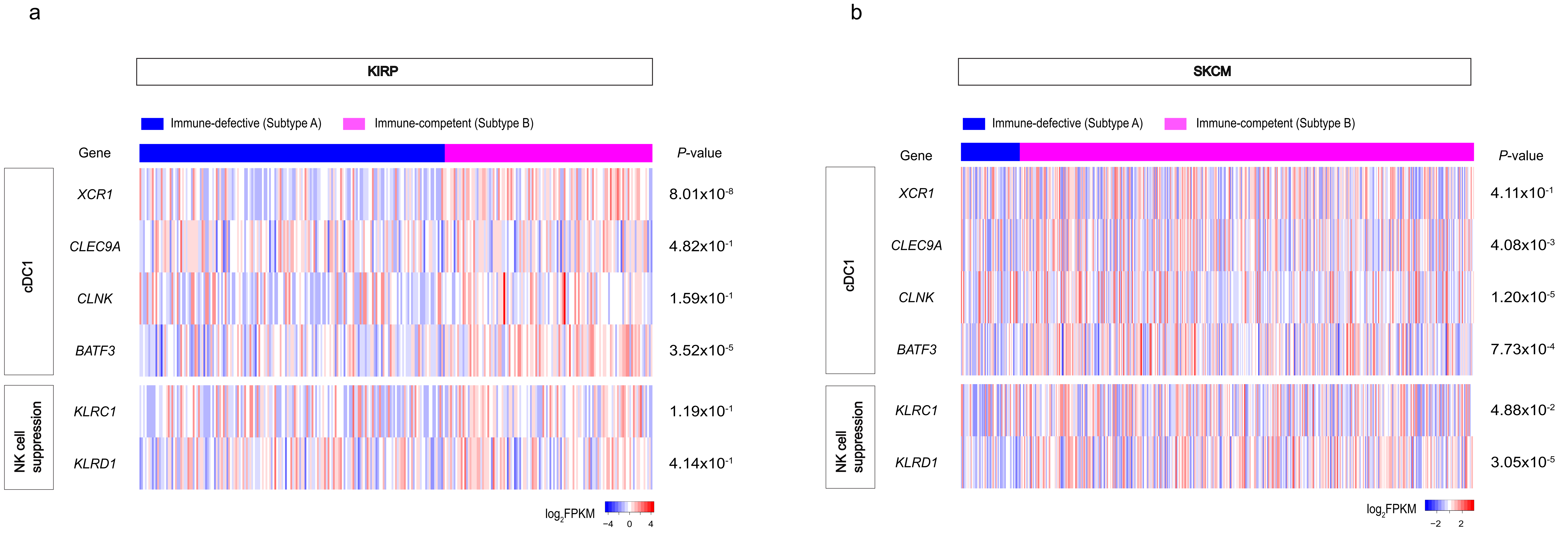
a
KIRP
Immune-defective (Subtype A)
Immune-competent (Subtype B)
Gene
P-value
cDC1
XCR1
8.01x10-8
CLEC9A
4.82x10-1
CLNK
1.59x10-1
BATF3
3.52x10-5
NK cell suppression
KLRC1
1.19x10-1
KLRD1
4.14x10-1
log2FPKM
−4
0
4
b
SKCM
Immune-defective (Subtype A)
Immune-competent (Subtype B)
Gene
P-value
cDC1
XCR1
4.11x10-1
CLEC9A
4.08x10-3
CLNK
1.20x10-5
BATF3
7.73x10-4
NK cell suppression
KLRC1
4.88x10-2
KLRD1
3.05x10-5
log2FPKM
−2
2

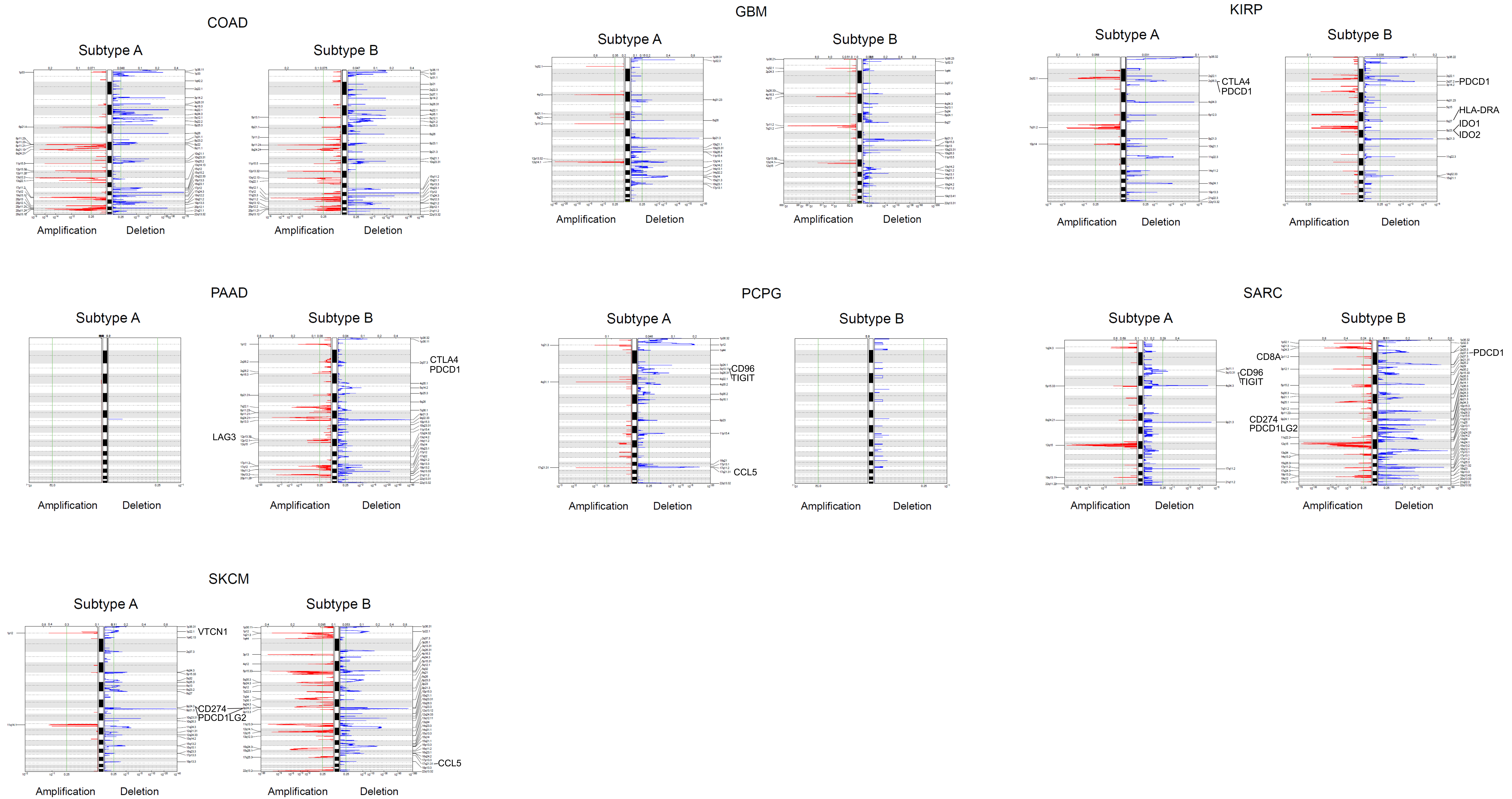
COAD
Subtype A
Subtype B
Amplification
Deletion
Amplification
Deletion
GBM
Subtype A
Subtype B
Amplification
Deletion
Amplification
Deletion
KIRP
Subtype A
Subtype B
CTLA4
PDCD1
PDCD1
HLA-DRA
IDO1
IDO2
Amplification
Deletion
Amplification
Deletion
PAAD
Subtype A
Subtype B
CTLA4
PDCD1
LAG3
Amplification
Deletion
Amplification
Deletion
PCPG
Subtype A
Subtype B
CD96
TIGIT
CCL5
Amplification
Deletion
Amplification
Deletion
SARC
Subtype A
Subtype B
CD8A
CD96
TIGIT
CD274
PDCD1LG2
PDCD1
Amplification
Deletion
Amplification
Deletion
SKCM
Subtype A
Subtype B
VTCN1
CD274
PDCD1LG2
CCL5
Amplification
Deletion
Amplification
Deletion

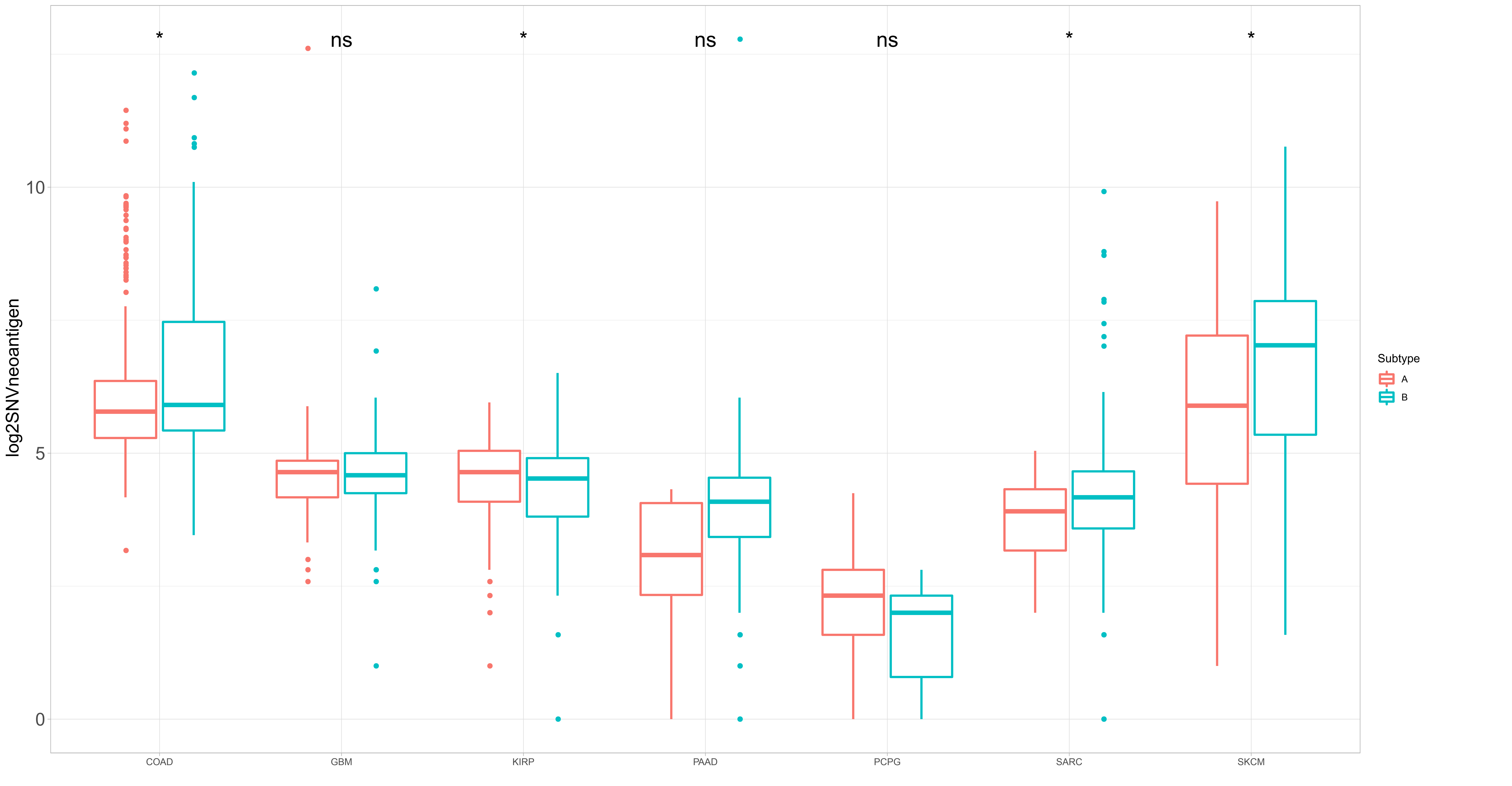

*
ns
*
ns
ns
*
*
log2SNVneoantigen
10
5
0
COAD
GBM
KIRP
PAAD
PCPG
SARC
SKCM
Subtype
A
B

Immune-deficient subtype (Subtype A)

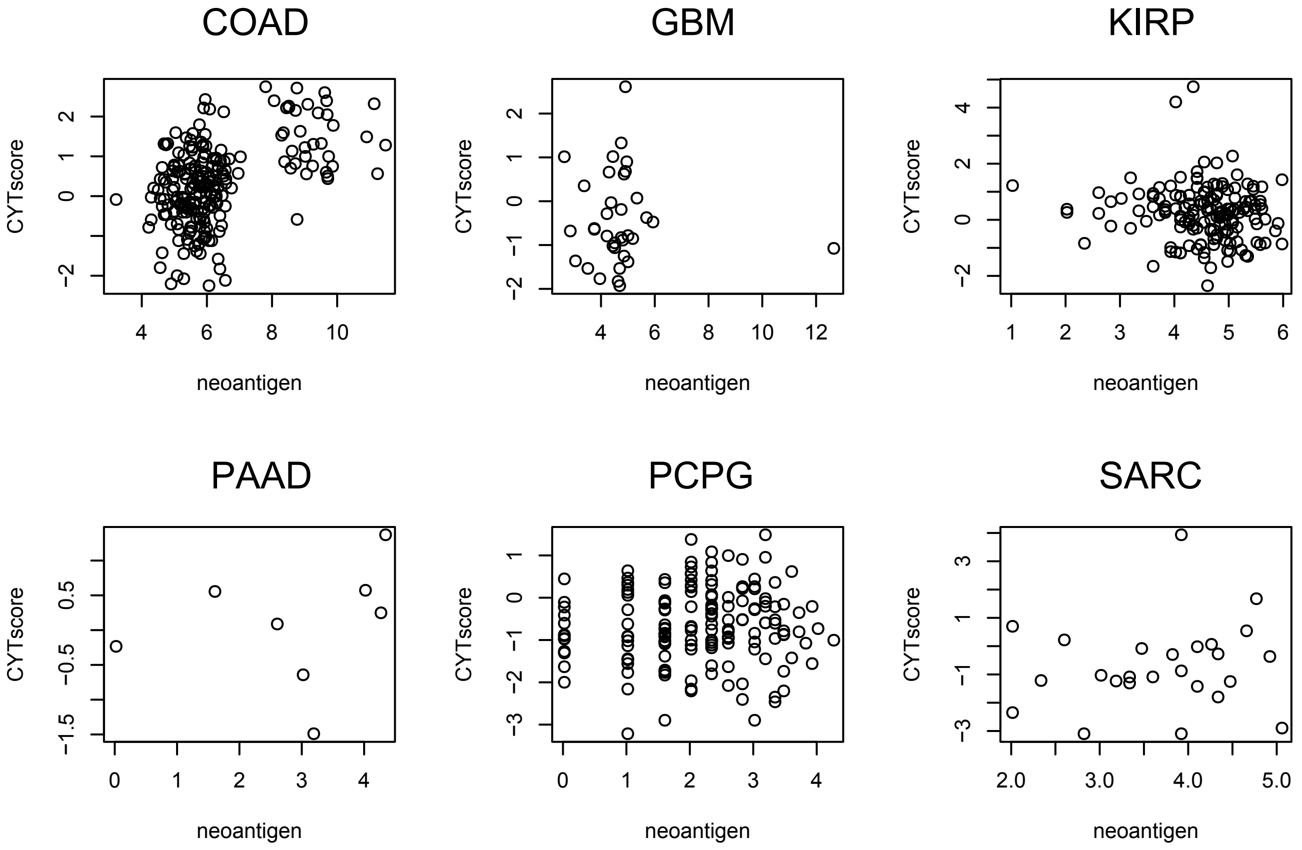

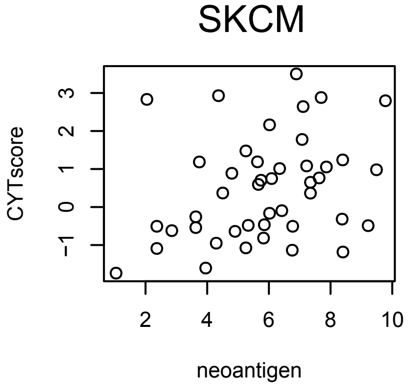

Cancer type	Pearson's correlation coefficient	p-value
COAD	0.47	3.18.E-13
GBM	-0.07	6.91.E-01
KIRP	-0.10	2.20.E-01
PAAD	0.27	5.25.E-01
PCPG	0.06	4.41.E-01
SARC	0.13	5.30.E-01
SKCM	0.30	4.71.E-02

Immune-competent subtype (Subtype B)

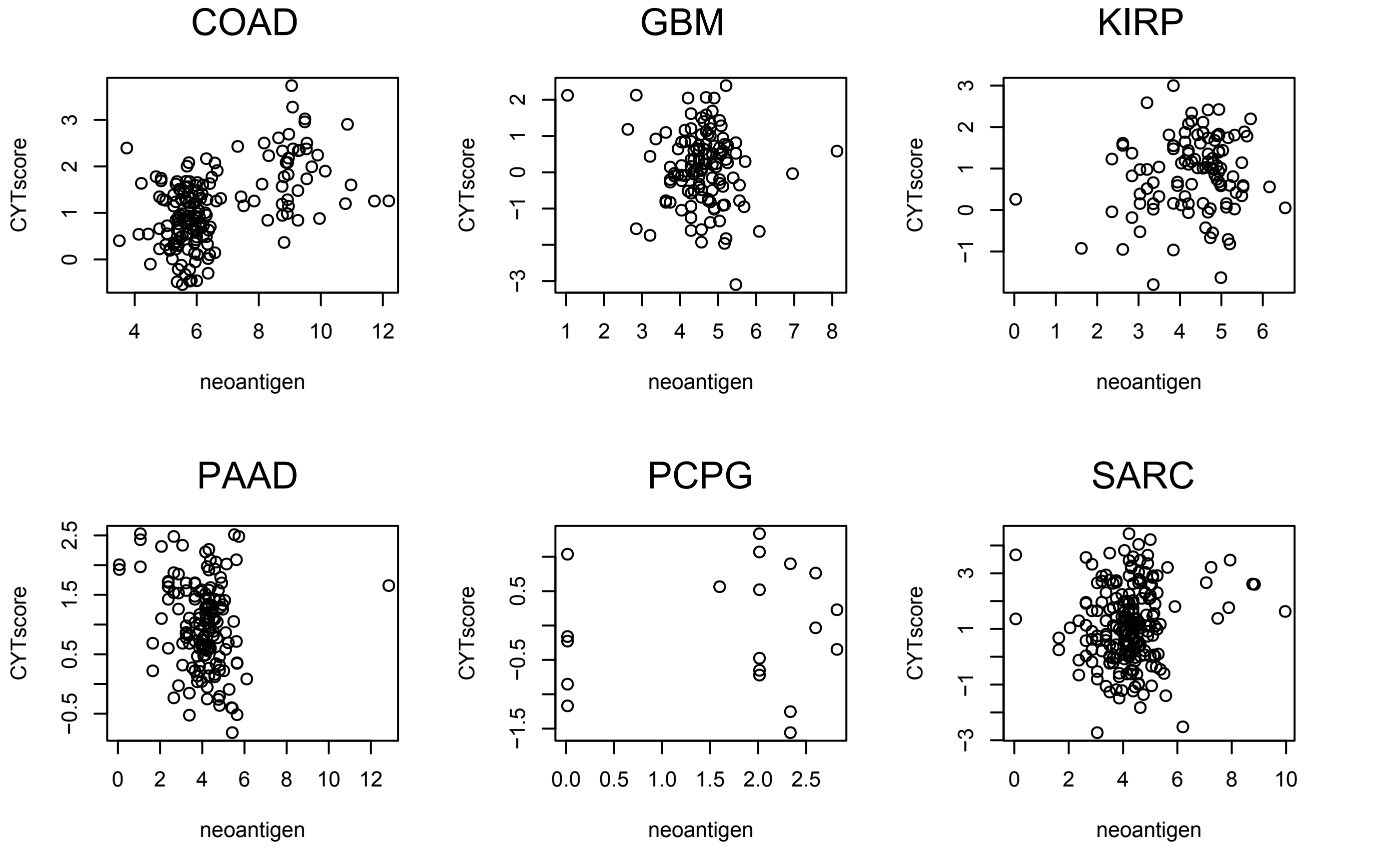

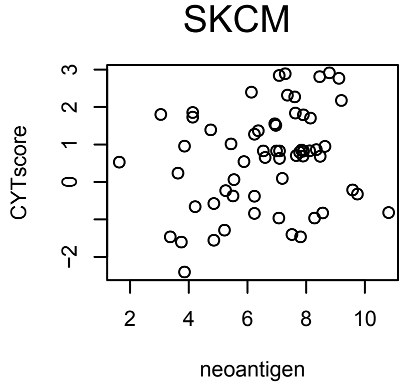

Cancer type	Pearson's correlation coefficient	p-value
COAD	0.51	1.60.E-12
GBM	-0.12	2.03.E-01
KIRP	0.15	1.35.E-01
PAAD	-0.20	1.57.E-02
PCPG	0.11	6.44.E-01
SARC	0.12	8.77.E-02
SKCM	0.23	7.87.E-02

Supplementary Table 1. Top 1,000 most variable genes used for unsupervised hierarchical clustering in

CHOL	COAD	GBM	KICH	KIRP	LIHC	PAAD	PCPG	SARC
RPS4Y1	RPS4Y1	PSPHP1	NKX6-1	UMOD	REG3A	PNLIP	RPS4Y1	RPS4Y1
PLG	REG1A	RPS4Y1	RPS4Y1	AQP2	RPS4Y1	CPA1	DDX3Y	DLK1
XIST	XIST	XIST	FXYD4	RPS4Y1	MYH4	CELA3A	CYP11B1	RP6-170F5.
HP	CLCA1	GSTM1	SPINK1	XIST	DLK1	CTRB2	XIST	DES
APOA1	REG4	KDM5D	LHX9	FABP7	CPLX2	CTRB1	KDM5D	DDX3Y
APOC3	OLFM4	IRX1	DDX3Y	SLC12A1	CYP3A4	CPB1	USP9Y	MYH11
MTRNR2L1	REG3A	DDX3Y	XIST	FGB	PLA2G2A	CLPS	HSD3B2	PCP4
TXLNG2P	DUSP27	SAA1	SST	DDX3Y	PGC	CELA3B	EIF1AY	KDM5D
APOA2	REG1B	LTF	PSCA	KNG1	CYP2A6	CPA2	SST	MYOCD
KDM5D	DDX3Y	EIF1AY	ATP1A3	FGG	APOA4	REG1B	NLGN4Y	MMP13
DDX3Y	ITLN1	USP9Y	USP9Y	TMEM213	REG1A	CELA2A	TXLNG2P	PLA2G2A
ADH4	TXLNG2P	UTY	KDM5D	KDM5D	CYP2E1	AMY2A	ADCYAP1	COL11A1
AGXT	MUC2	TXLNG2P	TMPRSS11I	USP9Y	XIST	REG3A	KIAA0125	XIST
OLFM4	ZG16	SAA2	UNC5D	ATP6V0A4	CYP1A2	CTRC	PNMT	CAPN6
USP9Y	KDM5D	H19	AQP6	SLC6A19	SPINK1	PNLIPRP2	UTY	EIF1AY
SULT2A1	SPINK4	TTTY15	TMEM52B	CLDN8	C9	SYCN	TTTY15	TXLNG2P
SERPINC1	PRAC	CDKN2A	RP6-170F5.	PLG	DCAF4L2	GP2	NPY	ACTC1
SLC6A19	IGHA2	ZFY	CRTAC1	SLC6A3	PEG10	PRSS1	CYP17A1	MYOC
AGR2	PIGR	TBX5	SALL3	SAA1	GSTM1	PNLIPRP1	ZFY	GREM1
EIF1AY	SLC26A3	DLX6-AS1	EIF1AY	FXYD4	DDX3Y	PLA2G1B	CYP21A2	CHRDL2
APOA5	DEFA5	PI3	LINC00645	RHCG	IGF2	CEL	NGB	ACTG2
HMGCS2	LCN15	IGKC	HHATL	UTY	CRP	GCG	CRH	COMP
GSTA1	DMBT1	IGHG1	PAH	TXLNG2P	ALDH3A1	XIST	PCDH11Y	NLGN4Y
MUC5B	EIF1AY	SAA2-SAA4	SCG3	AC079466.	RP11-51M1	REG3G	GNG8	USP9Y
C9	IGF2	C6orf15	CHL1	SLC22A6	MAGEA3	RPS4Y1	ECEL1	SCARA5
FDCSP	UGT2B17	PLA2G2A	VSIG2	EIF1AY	EEF1A2	ENSG0000(	ARHGAP36	IGHG1
F9	RN7SK	LINC00689	CALCA	FGA	AKR1B10	REG1A	AQP2	TCEAL2
AHSG	CTSE	POSTN	CYS1	SLC4A1	PAGE4	INS	POU3F3	CES1
KLK11	RPL41P1	NLGN4Y	SERPINA5	SCNN1G	CYP2A7	DDX3Y	GSTA1	PI16
EVPL	MUC17	RNA45S5	GALR1	ZFY	SSX1	KDM5D	CACNG7	IGKC
UTY	MUC5AC	GPR17	POU3F4	FOXI1	EIF1AY	PGC	CNTNAP4	LRRC15
KRT6B	MUC5B	OPALIN	CRH	ALDOB	KDM5D	CELA2B	MTRNR2L1	UTY
CEACAM7	PLA2G2A	IGHG2	UTY	PPP1R1A	MAGEA6	PPY	SLC32A1	PRAME
PRAME	DEFA6	IGHA1	TXLNG2P	MTRNR2L1	MUC5B	TXLNG2P	CHRM1	DCX
CPLX2	NOTUM	NNAT	ADH4	REG1A	HSD17B13	REG1P	GAL	ZFY
DLK1	CEL	ENSG0000(	PIK3C2G	CYP4A11	MME	REG4	NR5A1	ADH1B
ALB	CLCA4	IGKV4-1	CHRM1	AC019117.	KRT23	USP9Y	TMEM114	BMP7
TF	RP4-765C7	COL20A1	PSPHP1	GPR110	MAGEA1	MTRNR2L1	SYT13	IGHG3
REG1A	ENSG0000(	VIPR2	GPR123	BSND	AC079466.	TTR	TMEM132I	GABBR2
MSLN	ENSG0000(	C5orf38	PCP4	KCNJ1	COX7B2	DPCR1	PSPHP1	IGHG4
CTSE	UTY	KLRC2	BMP7	ATP6V0D2	THRSP	CRP	UCN3	ASB5
HRG	CYP2W1	ENSG0000(	PTPN20A	REN	AC073236.	EIF1AY	PCDH11X	PRKY
KNG1	LEFTY1	MOBP	KCNJ3	CLCNKB	SLC22A31	CLDN18	TMEM132(	IGHG2
CPN2	IGHA1	AC104135.	CDH19	LBP	SLC6A11	SERPINI2	SFRP5	WIF1
MUC6	KRT23	HMX1	ADCYAP1	SLC22A12	SLCO1B3	UTY	STAR	IGHM

RBP4	J01415.11	SLC17A7	STXBP5L	NLGN4Y	EPCAM	MUC17	RP11-672L	COL4A6
ORM1	L1TD1	CA10	MUCL1	PAH	MAGEC2	RP11-331F	AC018730.	HSPB3
CXCL5	DUOXA2	TTTY14	CA10	UGT3A1	SAA1	MUC5AC	PPP1R1B	CASQ2
BHMT	CLDN18	HOXD13	ZFY	C5orf46	SAA2	AQP8	FSTL5	NEFM
UCA1	DUOX2	HOXB13	SALL1	SORCS3	TXLNG2P	CLCA1	SERPINA5	CRABP1
NLGN4Y	J01415.10	TAC1	NRK	AQP6	AFP	CRISP3	CYP11A1	MAPK4
PPP1R1B	IGJ	GABRA1	AZGP1	CA9	PSPHP1	CEACAM5	SLC4A10	HOXC12
SLCO1B1	RP11-462G	HP	PKP3	RBP4	PRAME	CR2	SLC6A15	CNN1
SLC25A47	LY6G6D	PRKY	STXBP6	C10orf99	IGHG1	KRT6A	OLFM3	RP11-48O2
FABP1	CA4	SIX6	DHRS2	PROM1	CHRNA4	MUC2	CRABP1	PPAPDC1A
ZFY	GNG4	IGJ	PSG4	HABP2	NTS	IAPP	RP11-473A	KIF1A
CYP2A6	KRT8P36	RP11-89N1	GRIK5	SLC34A2	CYP1A1	OLFM4	GABRA1	IGLC3
ACSM2A	DES	IGKV1-5	LRFN5	HMGCS2	C5orf27	RBPJL	SMOC1	FOXF1-AS1
AFM	FABP1	IGLV3-21	PIP	C8orf22	HGFAC	ZFY	STRA6	ENSG0000(
CYP3A4	TCN1	IGKV3-20	CDH12	SLC22A8	S100P	APCS	TAC1	HMGA2
PAEP	HMGCS2	CXCL13	CYP1A1	SLPI	MUC13	KRT20	EYA1	C8orf22
CCL19	SFRP2	GRIN1	RP11-849I1	SLC5A8	CTNND2	PRKY	CSMD3	KCNH2
PRKY	ZFY	CYP4F24P	FOXI2	MUC15	CTD-2195N	PSPHP1	COL20A1	TMEM132(
PADI3	MEGT1	CRABP1	CYP1A2	NDNF	USH1C	PSCA	CALCA	IGKV4-1
IGKV4-1	CEACAM7	IGHG3	C10orf71	SST	AGR2	ADIPOQ	EPHA5	CADM3
F2	IGHG4	GABRG2	NLRP2	SLC12A3	SLC22A12	SLC30A8	SULT2A1	MFAP5
SLC2A2	ENSG0000(	HOXC10	SLC4A1	PIGR	COL2A1	MUC6	MGST1	ACTA1
GP2	USP9Y	DKK1	SLC22A2	PSPHP1	MTRNR2L1	LY6D	KLK3	COL2A1
CEACAM5	MUC6	IL8	NMRK2	NPHS2	UGT3A1	TFF1	MC2R	IGKV3-11
IGHG1	B3GNT6	TRDC	CLIC6	IGFBP1	XPNPEP2	MUC16	NPFFR2	C7
AQP5	MTND4P12	HOXC13	GSTM1	HP	RHBG	KRT5	GABRG3	IGLV3-21
UGT1A10	CLDN2	MARCO	TFAP2C	DMRT2	HEPACAM	G6PC2	KCNIP1	PLN
TFF2	IGHG1	CXCL6	SMOC1	TMPRSS2	SDS	NLGN4Y	PIRT	IGLC2
UPK1B	CA1	MOG	TTR	PLA2G4F	TRIM71	TFF2	TAC3	DKK1
CDC20B	RP11-474D	CHGA	MOG	SIM1	CYP8B1	CUZD1	CLVS2	MEG3
GSTA2	HULC	DCX	RP11-115D	ALPI	LGR5	PADI1	CNGB1	SLC7A3
ECEL1	DPEP1	APCDD1L	KNG1	NPTX2	CHI3L1	ITLN1	TMEM196	TCF21
CYP8B1	IGKV4-1	IRX2	PDZRN4	STAC2	CYP7A1	PDIA2	GRIK1	PSPHP1
FGB	NXPE4	DLK1	FRMD7	DUSP9	ADH4	SPINK4	OPRM1	IGKV3-20
ALDOB	NPSR1	MAG	ENSG0000(	AC010127.	PRSS8	CTD-2377D	GLP1R	RP11-471J1
CRP	PCSK1	LUZP2	ANO4	HEPACAM2	PTH2R	TM4SF20	GHRH	VIT
DKK1	IGLV3-21	PDYN	DUSP9	PNCK	IGF2BP1	IGHG4	C1QL2	GS1-600G8
ADH1A	CXCL5	GJB6	LRAT	KRT223P	SLC6A2	SPRR3	SH3GL3	PAX3
HAO1	MS4A12	F13A1	NLGN4Y	SLC13A1	TM4SF20	DMBT1	DIRAS3	RPE65
C1orf186	CDHR1	SMOC1	SIX6	CLCNKA	UGT2B17	KLK7	NPY2R	OGN
CFHR1	PSPHP1	TMSB15A	ENSG0000(	SLC17A3	KRT19	APOA4	TFAP2C	LUM
RP11-799O	IGHM	PITX2	PTGFR	CASR	GCGR	SI	PPP1R17	CXCL14
FGF19	PRKY	RP11-231C	HMGCS2	KLK1	UTY	RP11-462G	RPH3A	TTTY15
SAA2	B4GALNT2	TBX5-AS1	MTRNR2L1	GSTM1	BPIFB2	ENSG0000(	AADAC	SALL1
CA9	CKMT2	C1orf158	ENSG0000(	SLC5A12	CTAG2	BPIFB1	IGHG1	IGHA1
SLC22A7	IGHV4-39	POU4F1	PLCXD3	CHRDL1	RELN	KLK1	GDF10	LONRF2
RP11-554I8	FAM3B	IGLC3	RP13-436F	PVALB	NOTUM	TTTY15	GDA	UNC5D

CYP1A2	TFF1	IGLC2	TPTEP1	FGF9	FXYD2	PLA2G2A	RELN	KRT14
IGLV2-14	PPBP	PCSK2	CDH7	GRIA4	SPP1	KCNK16	PRKY	HOXB13
ASGR2	ACSL6	MMP13	LINC00284	GC	SLC22A11	NPSR1	LRRTM4	LMOD1
UGT2B4	EREG	IGLV2-14	SLC6A15	GGT6	PRSS3	PCSK2	PAX5	C20orf166-
SLC13A5	AQP8	GLRA3	TRHDE-AS1	CD70	ISX	KLK6	FNDC9	ABCB5
HPX	VSIG2	MMP7	GLDN	SLC9A4	SAA2-SAA4	GSTM1	TFPI2	SLC6A15
ST8SIA3	IRX2	WBSCR17	FREM2	UGT1A9	MAGEA12	CXCL5	POF1B	PRG4
SLC44A4	IGHV5-51	CPLX2	ENSG0000(	CPN2	AP1M2	DLK1	HPGD	CHL1
CR2	C10orf99	WIF1	GDA	PRKY	MT1G	ONECUT3	CDH10	IGLV2-23
TMPRSS4	IGKV1-5	IGLV2-23	MAOB	MYEOV	ZIC1	UPK1B	LHX9	CHI3L1
CDH16	IGHG3	CXCL5	FAM70A	PRIMA1	HPGD	RP6-149D1	EYA4	IGKV1-5
S100P	ZIC2	DSG2	PALM3	ESRP1	RP11-539E	SPRR1B	CDH12	HAS1
SAA1	IGLV1-40	CCK	CFTR	SERPINA5	GCK	RP11-680B	NKX6-1	DCN
TTR	IGLV3-19	KLK6	KCTD8	SAA2	FAM3B	CGB5	IGSF10	CA3
VTCN1	MAP7D2	SLN	CYSLTR2	SLC6A18	ENSG0000(	MS4A1	HAND1	IGLV3-19
ENSG0000(	LYPD8	CNDP1	WNT2	CTB-27N1.:	GLYAT	MYBPC1	PON1	IBSP
IGKV3-11	IGHG2	HOXB8	CTB-27N1.:	EPO	SLC25A47	B3GNT6	HSPB7	MARCO
UGT1A8	CHGA	NEFM	STAP1	CYP4A22	MAGEC1	GC	RBBP8NL	WBSCR17
SUCNR1	CHP2	IGLV1-40	HEPACAM2	RP11-1C1.4	NPC1L1	CHGA	RP11-424G	IGLL5
MAT1A	IGLV2-23	IGFN1	DPP10	TTTY15	GSTA2	SST	TUBB4A	SFRP4
SPINK1	CA9	IGHA2	IGHG1	SLC34A1	UGT1A4	CHGB	IGKC	IGFN1
SLC5A8	IGLV2-11	PCDH15	ADAM2	NELL1	CTNNA2	SERPINB3	CDH19	RP11-371I1
ALDH3B2	IGLV2-14	CHRNA1	RIMS2	ATP6V1B1	GPR88	A2ML1	FRMPD4	LMO3
IGKC	SPP1	KCNE1L	VWC2	C12orf36	HPD	ALB	SCRT2	IGLV1-40
PSPHP1	CALB1	HOTAIR	GPRC6A	ATP6V1G3	PDZK1IP1	LEFTY1	GCGR	PCDH10
ENSG0000(	IGHV1-18	BMP5	FOXP2	RP11-371I1	TINAG	KLK8	KCNT1	JPH2
RP11-1C1.4	IGKC	IGHM	PAK7	MT1G	DHRS2	IGHD	AC138430.:	CCL21
KRT17	IGKV3-11	INA	IYD	TMEM174	ASPG	GABRP	APCDD1L-A	TRDN
NTS	RNU4-2	RP11-219E	GALNTL6	MYH8	IGKC	FGA	RP11-60L3.	IGHGP
HPD	KLK6	SLC14A1	GYG2P1	RAB25	ODAM	CEACAM7	MME	CBLN1
HOXB13	IGLV3-25	COL6A3	FGF14	CLDN19	DKK1	FDCSP	GLRA3	LMO1
SLC10A1	AC016735.	STMN2	WSCD2	CYP24A1	RP11-697M	FER1L6	IGHG3	ASB2
SERPIND1	FOXJ1	ST8SIA3	RP4-765C7	SFRP2	UROC1	FGG	SP5	SMOC1
GSTM1	ENSG0000(	FREM3	FOXI1	PCK1	SLC22A1	FABP1	MTND4P12	WT1
HPR	IGLL5	ENSG0000(	TRGC1	CCL21	HAMP	HOXA13	GPC5	CNTN1
MBL2	DSC3	DLX5	TTTY15	LPPR5	GNG4	CPLX2	GPR98	RBM24
SSTR5-AS1	IGHV3-23	HOXB4	RP11-527H	CAPN6	SLC10A1	HMGCS2	KRT9	RP11-887P
IGLV3-21	PRHOXNB	SFRP2	RNF212	RP11-690G	CXCL6	EPYC	KCNJ6	TENM3
MUC5AC	MSLN	CHI3L1	MTND4P12	ADCY8	DEFB1	DES	NPBWR2	TNNT3
PLEKHS1	SI	SLC18A3	PRKY	FTCD	UPK3A	AC016735.	TRPC5	MTRNR2L1
IGHV3-23	COL10A1	RP11-178A	CHGB	KRT19	CPS1	CTSE	SLITRK1	IGF2
KLK10	FIBCD1	HOXC11	TRHDE	TEX15	PDX1	KRT16	PPAPDC1A	WNT2
CXCL13	ALDH1L1	COL11A1	KCNJ16	SPTBN2	TRIM55	S100P	IRX4	TMEM179
PRSS21	IGLV4-69	EYA4	THRSP	HRG	PTGDS	DSG3	TTTY14	IGHV1-18
LECT2	HEPACAM2	ARMC3	CPXM2	KISS1R	LCN2	TCL1A	NELL1	CTC-296K1
ANGPTL3	COMP	NR0B1	CLNK	SLC17A4	RP11-456A	LYPD2	FGF10	IGLV3-25
SERPINA11	MAGEA3	RP11-300M	RSPO3	PRAME	B4GALNT2	VSIG1	COX4I2	IGLV2-14

ORM2	MTND1P23	CACNG3	TBATA	G6PC	SSTR5-AS1	SIM1	JPH3	PRL
FGG	SLC39A2	NSG2	TMPRSS11A	IGHG1	SFRP5	CELP	SOHLH1	TCF23
GGT6	IGHV3-15	SCRT2	MME	C7	TAT	PSAPL1	AC013402.	NRK
LPA	IGLV6-57	CSF3	CTB-92J24.	BIRC7	IGHG4	GAD2	PMP2	RIMS2
ARG1	MUC12	GSX1	UNC80	IL20RB	RP11-101E	MIR205HG	ENSG0000(	WNT7B
ADH1C	IGHV4-59	ST8SIA2	RP11-161M	RHBG	KNDC1	SFRP5	CPB1	TENM2
RTL1	IGLV3-10	TENM3	KCNJ1	CALB1	MMP7	FAM83A	PTPRZ1	VGLL2
SFRP2	NPTX2	IGLL5	L1CAM	SLC26A7	C19orf21	CASP14	GSTM1	XPNPEP2
PRAP1	IGLC2	VSTM2A	PTPRD	FAM83F	CYP17A1	MUC5B	NRK	IGHV4-39
IGHV4-39	IGLC3	PCDHGB7	SLC3A1	MCHR1	DUOX2	MSLN	ABP1	IGHV3-23
CST1	IGKV3-20	IL2RA	CASR	ABP1	ZFY	KRT13	KCNJ3	GRIK5
TTTY15	ADH1C	IBSP	RP11-19E1	MAL	HSD11B1	ABCC8	NXPH4	PRIMA1
ENSG0000(	ABHD12B	14-Sep	LRRTM1	MTND4P12	SLC13A3	FGL1	JAKMIP1	CTC-296K1
IGHG2	DSG3	MBP	FLJ00388	GSTA2	KRT7	PRSS3P1	FAM19A4	OSTN
NLRP2	HMGB1P5	TLX1	KRT7	SFRP1	RP6-170F5.	BTNL3	CACNG3	PRPH
MUC16	IGLV8-61	SP8	TTTY14	OSTM1-AS1	GREM2	CRYBA2	RFX6	HAND2
UGT2B10	ENPP3	KCNC2	TRPC3	SLC5A1	CA9	PTF1A	MLPH	NKX2-5
IGHA1	SLITRK6	PCDH11X	MUC15	TFCP2L1	C19orf77	AMBP	SIK1	PROM1
KRT81	IGKV1-9	MYT1L	RASSF6	CP	FAM133A	MSMB	CCL21	DPYSL5
TAT	IGHV1-46	VSNL1	KLK1	SCGN	FGFR2	GJD2	FAM5C	WISP2
GABRP	IGHV1-2	NKX6-2	BCAN	SLC10A2	MEP1A	MTND4P12	IGLON5	IGLV1-44
SPP2	NLRP2	LPPR1	F11	MT1H	CTA-85E5.1	COL17A1	IGHG2	DPP6
IGKV3-20	ENSG0000(	HLA-DQA2	KNDC1	PTHLH	CSAG1	COL11A1	RPRM	GSTM1
ARHGAP40	RETNLB	GATA4	LHFPL4	ENSG0000(	HRG	DUOXA2	SHD	DPT
MS4A1	PI3	PAX3	ATP6V1G3	LINC00645	IGHG3	SCGN	MUC15	LEFTY2
TFF1	KLK10	HOXA4	CCKAR	BMPR1B	NEU4	HOXC10	PTPN20A	PART1
TRIM50	CST1	RP11-197K	ATP2B2	GCGR	CYP2B7P1	SYT8	PCP4	GFRA1
PCSK1N	UGT2A3	HAND2	LINC00543	APOB	IGHG2	ANXA10	C9orf135	WFDC2
ENSG0000(	MTRNR2L1	HMGA2	REN	NR0B2	FAM99A	PIGR	MT3	PAGE5
GLYAT	SMOC1	GALNT13	KIAA1644	RP11-477N	SLC34A2	CXCL13	RASAL1	PTCHD1
UGT1A9	CAPN6	RPH3A	DGKI	MT3	ESRP1	TRIM29	CNGA3	GATA5
CTD-2015G	TFF2	FAM163B	C11orf53	TFAP2B	HAO2	UGT2A3	RP11-700H	PDPN
IGLV1-40	MAGEA6	IGHV3-23	KRT19	RP11-397G	H19	CFC1	UGT3A2	PLP1
KLK6	SLC13A2	LHFPL3	COX6CP1	MSLN	ADH1C	TMED11P	ODAM	MAGEL2
DPYS	SFRP4	IGLV2-11	FBN3	GRHL2	AKR1B15	SCGB3A1	PENK	IRX1
PPP2R2C	WIF1	MYT1	ADTRP	SLC13A3	SLPI	KCNJ16	GAD1	RP11-615I2
HOXA13	IGLV1-51	GABRA5	WBSCR17	EGF	LECT2	C6orf15	COMP	HAPLN1
ACSM2B	CPS1	CTD-2339F	SDK2	CTXN3	KCNU1	CFTR	CRTAC1	DSC3
REG1B	MMP7	ATCAY	NPY6R	CNTN1	IGKV3-20	DUOX2	APCDD1L	LHX8
MTND4P12	TRIM72	DLX6	COLEC12	EHF	IGKV4-1	SLC9A4	FAM19A3	HMCN2
VGLL1	LRRC26	ZNF488	BPIFA2	NAT8	CLRN3	CLPSL1	GPR139	IGJ
SLC6A11	ELF5	EGFR	ENSG0000(	C1QL1	PAEP	SPRR1A	EDN3	FGF5
LINC00494	PLA2G12B	TRH	PSG5	PAPPA2	CDHR2	NEUROD1	TMEFF2	ENSG0000(
AGXT2	ENSG0000(	IGLV3-19	FAM135B	DCN	WNK2	LCN2	KCNK2	TNNT1
IGHM	ZIC5	AC017048.	ERP27	ENSG0000(	DMKN	CEACAM6	CYP26A1	EEF1A2
IGHG4	KRT6B	RP4-765C7	SLC4A9	DNER	RP11-414K	TNS4	ADH1B	RPRM
IGHG3	RP11-438N	IGHV1-18	RPSAP53	LGALS4	USP9Y	AQP5	TEX26	PGR

KCNJ13	IGLV1-47	IGFBPL1	SLC24A2	GSTA1	FGF19	FGF19	DPP10	ADH1C
IGLV2-23	IGHGP	LGR6	UGT1A6	WBSCR17	CLDN4	TTTY14	SLC18A3	COL10A1
IGHV1-46	ENSG0000(	IGKV3-15	CTD-2335A	SLC13A2	PITX1	RP11-697M	RNA45S5	CRTAC1
IGLV3-10	H19	SYNPR	LPPR1	EGFR-AS1	TMEM92	UGT1A10	SIX3	IGHV5-51
LBP	XPNPEP2	GALR1	SLC26A4	CSDC2	SPP2	KRT14	KLK4	PLIN4
SLC6A14	BMP7	CHST8	SLC26A4-A	UPK1B	LIN28B	KLK10	IGLV2-23	EDN3
CES1	RP4-706A1	SV2B	RIMS1	ADH1B	FGF21	ALPP	CGA	IGFBPL1
CALCA	ATOH1	TMEM125	C7	IGHG4	SYT13	KLK3	LRRC38	SBSPON
SFRP5	IGHV3-33	CA3	IGHG2	AP000439.	F9	AC104135.	ISL2	NPTX2
CYP4A11	PIWIL1	AC005550.	ATP13A5	RP1-163G9	CSMD1	RP4-765C7	SCGN	CSDC2
CEACAM6	IGHV3-74	CRYM	TBX15	TSPAN8	CST1	KRT17	IGHG4	B4GALNT4
PRODH2	B4GALNT4	IGHV4-39	SLC6A17	CXCL5	HAL	PHGR1	FAM166B	SFRP2
SAA4	ONECUT3	GAD2	RP11-690G	ZMAT4	IGKV3-11	SLC13A5	SLC1A6	MMP3
TM4SF4	TACSTD2	AC108142.	GJD2	LPPR1	MAGEB2	SULT1C2	GIP	MAOB
SERPINB5	AC016683.	NEUROD1	DMRT2	KCNJ10	GP2	IGLV8-61	LINC00648	SFRP1
AMBP	SLC28A2	DLL3	RP11-127I2	SCNN1B	SEZ6L2	TCN1	NMUR2	MME
SLC38A4	MMP1	CTD-2081K	SFTPB	XPNPEP2	PPP2R2C	CST1	GRM7	GJB2
IGKV1-5	IGLV1-44	RP11-189B	LTK	ASPG	DKK4	PAH	DGCR5	SERTM1
THRSP	IGHV3-11	MAB21L2	WFDC2	IGLON5	IGLV3-19	NLRP2	NPAS4	SCRG1
PCK1	ENSG0000(	KCNV1	TESPA1	IGKC	NQO1	SLC3A1	CXCL14	NIPAL4
PAX2	GUCA2A	TFAP2B	CEL	ENPP3	IGLC3	UGT2B15	NEUROG2	EMILIN3
FGL1	IGLV3-1	SCNN1B	SLC26A9	F2	ENSG0000(	IGLV10-54	MAL2	FIGF
CD79A	WDR72	CHODL	DBC1	HPD	GPC3	TMPRSS15	KISS1R	SOX2
APOB	MTTP	LBP	ATP6V1C2	EPS8L3	RP11-115C	SCG3	OR2L13	SCUBE1
AKR1D1	KLK7	PAK7	FOXD1	GABRA2	NPTX2	NKX2-2	ERBB3	ENSG0000(
IGF2BP1	IGKV3-15	PCDHA4	CDH22	NPHS1	CFHR5	HOXB13	CELF5	GREM2
MAB21L2	IGHV3-21	FEZF1	ALDH1A3	LINC00462	MUC6	PRAP1	IGHA2	SSX1
SERPINA7	HS6ST2	PCDHB5	CTA-929C8	MIOX	CFTR	SLC6A14	VIP	ASTN1
ADH1B	IGF2BP3	FBN2	GABRB3	CWH43	RP11-293B	DHRS9	PDYN	SGCA
AC092165.	CXCL14	IGHG4	BNC1	CREB3L3	IGKV1-5	FGB	IGHA1	CDH3
IGHV3-15	FREM2	JPH3	LUM	PROM2	BCL2L10	ENSG0000(	CYP11B2	CACNG4
ONECUT3	SLC9A3	RNU4-2	CGA	LINC00671	CNTFR	CCL19	TLX1	C3
HOXA10	LCN2	AC016683.	ALDH1A2	IGHG3	SUSD4	AKR1B10	DMP1	IGLV3-1
EPHA7	MMP3	EYA1	UMOD	SLC9A2	IP6K3	SLC26A9	IGKV1-5	NTF3
TSPAN8	CD177	RP11-438E	COX7A1	NRK	C6orf223	IGHV1-18	IGKV3-20	BMP5
GRHL2	MUC4	SFRP1	IGHG3	CYP2J2	LUM	SIK1	HAPLN1	MMP1
OTC	IGHV1-24	TMEM132(	DIO2-AS1	B4GALNT2	RP11-81H3	HOXC11	CHL1	COL4A5
SPP1	RP11-742N	ENSG0000(	RNF128	IGLV3-21	GTSF1	AMY2B	AC104135.	CTSG
CYP2B6	ENSG0000(	PCDHGA9	RP11-420K	L1CAM	GABRB3	KLK5	HPSE2	CTD-2195M
CFHR5	IGHV3-49	HOXC-AS5	CHD5	RP4-765C7	SMEK3P	NMUR2	RP13-49I15	LPHN3
CPS1	ALDOB	IGKV3-11	RP11-520P	SPON1	TRIM50	FEZF1-AS1	PRKG2	EPHA7
APOF	TNNC2	MAL2	TNS4	DCAF12L1	IGLV3-21	ST8SIA3	SLITRK5	HOXC13
CFTR	GRM8	HOXD10	FXYD3	DES	ENSG0000(	IGHV1-2	NCAN	KCNMB1
TTTY14	PRAP1	CTD-2554C	ZNF676	IGHG2	IGLV1-40	TFF3	GABRG2	WT1-AS
AADAC	KRT6A	SEMA3E	DAPL1	IGHV4-39	PCK1	S100A2	AOX1	PTPRT
PI3	WFDC2	NOS2	ATP2C2	ENSG0000(	MT1H	CLDN2	HOXA9	FGF10
TTYH1	UCA1	SYT13	DPP4	SH3GL2	ENSG0000(	GPR87	SHOX2	ENSG0000(

C8A	SLC13A3	VGF	DNER	ACE2	TUBB4A	C6	ATP2B2	MAGEA3
HSD17B6	AQP5	HOXB3	GLDC	PCSK1N	C7	IGHV1-69	UGT8	FOXG1
ENSG0000(	SLCO1B3	SLC17A6	DRD2	SLC36A2	MOGAT2	WNT7A	FEV	CHRM2
DNER	IGLV7-46	BCAS1	FABP6	DPEP1	TTC36	LINC00443	KRT80	RIMS4
IGLL5	GJB5	HOXA9	CNTNAP5	IGLC3	IGLV2-23	GSTA2	LL0XNC01-	IGLV2-11
IGHA2	IGHV4-34	IGLV3-25	IGFBP1	IGLV3-19	AC098973.	NTSR1	NOS1	SBSN
WIF1	NKD1	VSTM2B	RP11-711K	ENSG0000(	FADS6	LINC00643	ASB4	STXBP5L
GJB4	KLK11	CA9	KCNIP1	NDUFA4L2	MFSD2A	ERN2	NDST3	LRRN1
CYP2E1	MPV17L	LINC00599	RP11-85G2	TUBA3D	PPAP2C	SOX21	FABP6	TPSB2
TRIM29	LYZ	PNOC	AC007879.	ENSG0000(	AVPR1A	APOC3	EPHA8	BPIFB4
HFE2	TTTY15	HOXA13	MUC20	PCP4	IGLC2	DUSP27	SLITRK6	GPR133
ENSG0000(	NXPH4	SLC32A1	PRSS12	PRAP1	SERPINA7	CA9	SV2C	GPR158
FTCD	FZD10	CPNE6	ENSG0000(	LIX1	CNDP1	GKN1	IGLC2	FAM19A5
AP000688.	NXPE1	SOX1	AC066593.	IGHV1-24	HOXA13	APOA2	MTND1P23	ZNF536
IGLV3-25	IGKV1-17	C7orf57	MMP7	FABP1	BEX1	SLC30A2	GFRA3	IGLV6-57
SLC26A9	GP2	LINC00643	HS6ST3	CLIC6	CCL25	IGFL1	KCNJ5	ADAMTS19
C12orf36	SLC6A14	GJB1	SLC6A2	RP11-706O	CXCL5	CTD-2147F	RP4-765C7	PTGDS
LHFPL4	RP4-604A2	MAGEE2	SORCS2	SAA2-SAA4	BEX2	CXCL17	AC016683.	ITGA8
RP11-697M	IGLV10-54	HOXB-AS5	CDH16	CLDN16	STMN2	KRT6B	IGLV3-21	IGLV1-47
TRHDE-AS1	CKB	SCGN	TDGF1	TNFAIP6	DUOXA2	HOTTIP	OPCML	PDZRN4
POU3F3	IGKV1-16	GABRB3	RORB	RP11-627G	SCUBE1	CLDN10	MYH14	PI15
IGLV3-1	IGHV4-31	ELFN2	SLC26A7	HHLA2	CCL21	MMP7	EGR4	CPXM1
CFHR3	AC104135.	SLPI	DIRAS2	IGHM	NKD1	SYT4	RASSF6	HHIP
APOH	SLC44A5	ENSG0000(	IGHG4	TOX3	UGT1A9	IGLV3-10	NGEF	IGHA2
DMBT1	CACNG4	HOXB2	LMO3	HAO2	C8orf47	PRSS21	IGKV3-11	LBP
CHGA	WNT11	SLC44A5	PAX2	SOSTDC1	PCSK1N	LTF	MYLK3	PCSK1N
CACNG4	CHST4	IL13RA2	ASB5	SLC44A5	FREM2	MIR7-3HG	IGLC3	TNNI1
EREG	SPDEF	ACTL6B	CNGB1	VIL1	IGLL5	AC005550.	IGLV2-14	PENK
UGT2B7	CES1	NPPA	BMPR1B	ENSG0000(	FNDC5	BTNL8	RP11-420K	FRMD6-AS2
AC104135.	PCSK1N	SYT4	ADH1C	HS6ST2	PIGR	TUSC5	SERTM1	SCN2B
APCDD1L	IGHV1-69	RP11-676J1	RAB3C	ELF5	RP11-734I1	ENSG0000(	TLX3	CA9
DARC	SAA1	NPY2R	KCNH2	COL23A1	WFDC2	C12orf36	KIAA1644	TBX5
ENSG0000(	CYP2B6	PACSIN1	TPRG1	MCCD1	CACNG4	ALPPL2	DCHS2	SORCS3
TCN1	RP11-343H	SNAP91	RP11-379F	IGHA2	IGHV1-18	FFAR1	PROK2	GDNF
MYEOV	TMSB4Y	CDKN2B	KRTAP5-8	LUM	RP11-774D	IGHG1	SLC5A7	TPSAB1
SLC5A1	TM4SF20	GRID2	C6	IGKV3-20	SULT4A1	MMP1	EGLN3	SMYD1
FREM2	CELP	KRT5	EN1	IGHA1	IGLV1-44	IGHM	LHCGR	CHRDL1
IGLC2	RP11-115D	RP11-745C	PTN	IGLV2-23	NLGN4Y	IGHG3	PTCHD1	DACT2
CHST4	SERPINB5	FBN3	LAD1	CDH4	SLC1A2	IGLV3-19	EPHA7	IGHV1-24
PKLR	SNORA73B	NCMAP	SYT14	TDGF1	IGHGP	WDR72	IGHM	BCHE
COL17A1	IGF2BP1	EFCAB1	SIX1	WT1	IGHV4-59	SERPINA6	RET	LGR5
ERN2	RNU4-1	UBXN10	CYP3A4	ACSM2B	IGHV3-23	PRAME	SALL4	SERPINB2
RGS4	GABRP	CAPSL	EBF2	SLC6A20	SLC22A7	FCER2	AC002480.	SLITRK3
CTD-2297D	IL8	FEZF1-AS1	SFRP2	UNC5D	ACSM1	IGLV3-21	PLP1	SPON1
SLC13A2	GATA4	PRDM13	PRSS1	IGKV3-11	LGALS4	KRT4	RP11-247C	IGKV3-15
ENSG0000(	ACE2	RASSF10	SHISA2	IGLL5	TENM2	GPHA2	CLCNKA	ACAN
SULT1C4	RP11-431J2	ALDH1A3	CTD-2566J3	MAT1A	CD5L	IGHV5-51	IGLV1-40	IGHV3-15

C3P1	SDR16C5	CAPN6	ADH6	RP11-438N	CNTNAP4	CRH	MCF2	SLPI
CREB3L3	CA8	SEC61G	PCK1	KCNS1	DACT2	RP11-320N	H19	LUZP2
PRSS3	FEZF1-AS1	FAM81B	TNFRSF11E	IGLV3-1	SLC44A5	ANXA8	SCUBE1	SEMA3E
SDR16C5	CHRDL2	RP11-436F	GJB1	CXCL6	RP11-169F	UCA1	GRIA3	KRT17
IGJ	ADH1B	TMEFF2	IRX6	AKR1B10	TMC5	APOH	MAPK4	MYO3A
AC147651.	IGKV1-6	TRIM67	RP11-11N9	CDHR1	FABP1	DKK1	CNTFR	CHRNA1
IGHGP	FREM1	SVOP	AC010423.	TMEM130	SOAT2	IGHV4-39	RARRES2	ABCA9
ITGB6	EVX1	SEMA3D	RP11-8P11	IGKV4-1	CHL1	IGHA2	CCBE1	SORCS1
IGHV3-11	FOLR1	CALY	PCDH7	PIK3C2G	PTGFR	TNNT1	VSNL1	ENSG0000(
IGLV3-19	POU6F2	MEOX2	HCAR1	SLC22A2	ALPI	PAX5	ELFN2	SIM1
F13B	AC064834.	DMRTA2	RP3-395M2	CHIT1	BHMT	TMEM179	FAM5B	RBFOX3
ABCG8	KLK12	CPNE4	S100A14	ERBB4	AC104809.	SLCO1B3	NDUFA4L2	KCNB2
KRT23	KRT17	RIT2	RP11-326C	CYP4F2	CA12	LGALS4	GSG1L	MMP9
SNAP91	IGFBP2	IGHV5-51	CTD-2626G	IGLV1-40	PEG3	RP11-528G	MLXIPL	TRHDE
C8B	SOX2	SLC17A8	PKHD1	SLC22A7	B3GNT3	CACNG6	FOXD3	MASP1
DSC3	NTRK2	RP4-555D2	CDC20B	IGKV1-5	GYS2	FAM3D	C4orf6	DUOX2
TFPI2	GSTM1	SHH	CTD-2314G	SLC22A11	SLC12A1	FABP2	NNAT	ROBO2
AC073218.	SLC4A4	UGT8	ESPN	RP11-513G	IGHV4-39	ENSG0000(	LIN28B	PTPRZ1
FGA	PRSS33	COL1A1	SYT10	SYT13	AKR1D1	SLC17A4	RIT2	IGHV4-59
ANKRD1	PRSS21	FAM183A	C6orf223	MAP7D2	FCAMR	PIK3C2G	TNXA	PLA2G2D
KRT16	IGHV3-73	NETO1	DCAF12L1	LTF	IGLV1-47	IGKV3-20	DRD5	LINC00578
NPY	MARCO	HOXA2	AC104135.	BMP7	CCL19	RFX6	SLC6A5	ADRA1A
IGLC3	HAVCR1	LPPR3	SHISA3	ACSM2A	CXCL13	CPA6	ISLR2	MYLK
UGT1A6	SLC35D3	MMP9	KBTBD12	RP11-1C1.6	FAM19A5	CELF3	TMEM45B	C5orf46
ENSG0000(	NPC1L1	TSHR	SPINK5	SLC9A3	ZIC4	SPINK1	IGKV4-1	SLC24A3
UPB1	DHRS9	HOXA7	IGKC	VTCN1	GFRA1	IGLV1-44	MCOLN3	APCDD1L
CYP4A22	MALAT1	CXCL14	SIK1	ENSG0000(	EPS8L3	PI16	RP6-170F5.	EPHA5
IGLV1-47	THBS4	USH1C	SUCNR1	SPINK1	PAGE2	TMPRSS4	RP11-14O1	RP11-531A
IGHV5-51	GAL	MAP3K19	SEZ6L	LRP2	TUBA3C	IGHV3-11	C7	ATP1A2
IGHV1-18	RP11-297P	MSTN	TINAG	IGHGP	SULT1C2	IGLV4-60	ENSG0000(	GRIN2A
ENSG0000(	RP11-11N5	CARNS1	CYP4F11	AC104135.	ACE2	IGKV1-27	IL6	GPC3
RP11-325F	AKR1B10	IGLV1-44	MT3	RP5-884M(	ABCB11	CST6	ENSG0000(	RAMP1
IGHV4-59	PTPRO	GPR128	C19orf77	IGHV1-18	MYO18B	IGHA1	PRMT8	COL9A3
ZIC2	PAH	C8orf22	PIGR	IGHV5-51	GSTA1	PCSK1N	DLX1	VSNL1
SAA2-SAA4	RP11-362F	SRRM4	ENSG0000(	IGLC2	IGKV3-15	SCARA5	MYH6	EN1
ENSG0000(	HOXD13	GDA	IGHA1	IGLV1-44	RP11-397G	BEX1	COBL	HOTAIR
DUOXA2	FAM155B	GABRA3	RP11-672L	IGLV8-61	AQP9	IGLV3-9	IGHGP	FEZF1-AS1
PON1	MEP1A	SULT4A1	PCDH9	DDC	CYP3A7	IGLV3-1	SORCS3	C1QL4
UGT2B11	TMEM178E	LYVE1	SPATA17	IGHV1-2	CTB-43E15	ELF5	SYT6	CDH4
AC079466.	RAMP1	CNPY1	F5	ENSG0000(	CYP4F22	ERP27	C1QL1	ZIC1
TACSTD2	IGHV4-61	GABRG1	RP11-527D	CA10	ENSG0000(	CCK	NOG	ADCY2
ENSG0000(	TM4SF4	TEKT1	SLC35F3	RP11-380J	CXCL1	LEP	ZNF804A	SLC22A3
IGHV4-31	SLC14A1	AC004540.	ADAMTSL1	FABP6	RP11-556E	CHP2	CARTPT	STRA6
ACOT12	MYH11	LINC00486	ALDOB	C5orf27	CNNM1	RP1-60O19	CNTN6	SGCG
SLC22A1	SLC6A20	NKX2-5	RP11-2E17	RALYL	SLCO4C1	PI3	IGHV5-51	IGHV1-46
DUOX2	IGLJ2	IGLV3-1	DCAF12L2	FREM1	FDCSP	TRPV6	GRIA1	PTPRQ
RP11-510M	IGKV2-24	TMEM235	ENSG0000(	TMPRSS4	PRAMEF10	HABP2	MAGEL2	HS6ST3

SEMA3E	MAGEB17	CR1	IP6K3	MTTP	WI2-2610K	ENSG0000(	CBLN4	PGM5-AS1
IGLV2-11	IGHV3-72	PCDHGB1	RAB3B	IGFN1	FETUB	SLC38A3	CADM3	SYNDIG1
SLCO1B3	PCP4	OR4N2	HYAL4	CPA4	PROM1	ATOH1	MT1G	IGLV3-10
SLC3A1	ANPEP	NPTX1	HMGA2	SLC18A3	PAPPA2	CALY	RSPO4	RP11-865I6
PTH2R	AP003774.	EDN3	TPSB2	IRX6	CLDN2	CHIT1	TRPC7	C5orf38
F7	NOS2	WDR38	PCDHA11	IGLV2-14	RP11-242J7	IGLV1-40	PCDH19	NRXN1
LCN2	IGKV1-27	RPRM	HPGD	FBXL21	CHST9	EREG	TRH	FGF16
CP	EDAR	CCL7	LAMB4	FOSB	MT1E	IGLV3-25	ACTL8	DSG2
PTGDS	LINC00261	LRTM2	B4GALNT2	TMEM92	IGLV2-14	ENSG0000(	RP11-92A5	RP11-328N
DEFB1	QPRT	RAB3C	SPINK7	COMP	NRCAM	KCNH6	IGJ	TCEAL5
UGT3A1	MOGAT2	KRT75	IGHM	ANGPTL3	NRG1	FCRL1	BTBD17	COL22A1
FXYD3	IGHV3-48	STOML3	AC069213.	SLC15A1	FSTL5	SCTR	KCNK10	MDFI
ACE2	KRT20	CHRNA4	CLEC2L	C1orf116	CLDN10	IGKC	C4orf50	KRT75
GPR128	RBP2	AFF2	IGLC3	LIPH	ENSG0000(	INSM1	BCHE	GABRA3
UGT8	ECHDC3	GFRA1	CLEC4GP1	RP11-807H	GLS2	A4GNT	SHISA9	AC104135.
ENSG0000(	SLC15A1	HOXD11	ZNF536	PRR15L	CHST4	FAM159B	HOXA-AS4	MAGEA6
ABCB1	TRIM54	SLC1A6	ERVFRD-1	C1QL4	POF1B	APOA1	DES	HS3ST3A1
SLC39A5	GYG2P1	PAX1	ZNF209P	CXCL13	RP11-320N	SLC26A3	NOV	FEZF1
PSG4	GABRA2	RBFOX1	ARSE	TMEM52B	RTP3	RP11-109L1	IGHV3-23	RP11-210N
UGT1A1	PLA2G3	MIR219-2	OXGR1	CTC-327F1(	SLC13A5	MUC15	SSTR5-AS1	SYNPO2
IGKV3-15	ASCL2	LRRC15	COL11A1	SLC30A2	NPY6R	HEPACAM2	WFDC2	CNTFR
KRT6C	PRAME	CD209	IGHA2	ENSG0000(	BTNL8	IGHV2-26	NPPC	ST6GALNA(
HAO2	DRD2	VWC2	COCH	GREM1	DCDC2	IGLV2-14	SSTR1	HMX1
LPAR3	FER1L6	LHX9	TMC5	ANXA13	TRHDE	SERPINB5	GLDN	IRX2
PROM2	TNNT1	FA2H	RP11-280O	FAM83B	PRODH	HOXB9	PRLR	ANGPTL1
CPN1	DKK4	HOXB5	APOB	PADI1	ENSG0000(	IGHG2	CALCB	ADCY8
RP11-89K2	SHISA9	MMP1	SPON2	IGLV3-25	MSI1	LINC00675	SLC1A2	VWC2
CYP24A1	SCARNA5	ENSG0000(	RP11-43F1	DACT2	RP4-765C7	SLC13A2	CDH7	RP6-24A23
CTD-2377D	CXCL9	IGLV6-57	ELMOD1	CDHR5	SLC3A1	MIA	KCNJ9	IGHV3-33
FOLR1	CYP4F2	GSTM5	RP11-513O	TEX11	SLC16A9	CYP24A1	MS4A8B	HS6ST2
FCRL5	C17orf77	GJB2	PTH2R	MMP7	MUC3A	PRSS3	RYR2	FABP4
IGLV1-44	RP11-1220	SCRT1	PCSK1N	PAEP	EFEMP1	LINC00483	ERBB4	IGF2-AS
SCTR	IGHV3-53	RP11-713C	LGI1	RGS7	CNTN3	IGHV1-24	ZCCHC12	SHISA6
SCGN	IGLV3-9	ENSG0000(	CYP4B1	PKLR	UPP2	RP11-294O	IGSF1	AP000688.
SPIB	CHRDL1	SLITRK1	ZG16B	MAPK4	ENSG0000(	FGFBP1	NPTX1	GALNT13
DAO	PADI3	GSX2	PPP4R4	C2orf54	IGHV5-51	SLC39A5	IGLV3-19	POSTN
C19orf77	C6orf15	SHISA6	AHNAK2	A1CF	IGHV3-15	SVOP	PAX2	ITGBL1
APCS	CCL21	CCL20	C2CD4A	ANGPTL4	AQP8	RPSAP53	MEG3	KCNA1
ITIH2	PROM1	C10orf105	CHL1-AS2	AGR2	ARSF	IGJ	FAM153A	ALX1
PAX5	DCDC2	CHRM1	PITX1	NAT8L	CYP2C8	SLC6A19	MC4R	NELL1
LAMA1	PLIN4	ECEL1	THNSL2	OGN	AMN	SULT1E1	MRGPRE	IGKV1-9
NOTUM	HLA-DQA2	PCDHB17	SRRM3	DOC2A	SEZ6	UCN3	FAM70A	SHISA2
HS6ST2	SOX1	COL8A1	CTD-2243E	SLC17A1	TENM1	PPP1R1A	LRP1B	SYT4
KRT6A	IGLV4-60	ZNF300P1	ST8SIA6	TRIM54	ELFN2	CXorf61	CNTNAP2	CPA3
IGHD	SCNN1B	MME	C9orf84	F11	CYP2A13	IGKV1-5	ANKRD20A	IGF2BP1
C19orf80	LGR6	IGHV3-15	CTD-2124B	RP11-284H	OTC	IGLV2-11	DACH2	LRFN5
PIK3C2G	CTC-575D1	CDH18	PPP1R14C	ENSG0000(	CAPN6	MEP1A	SHISA7	SOSTDC1

IGLV3-27	AGR3	RP5-1119A	EEF1A2	PLA2G12B	IGLV3-1	IGLV5-45	IGLL5	BARX1
CNTNAP5	B3GALT5	AGXT2L1	BICC1	COL11A1	SPINT1	BLK	SDR16C5	ENSG0000(
CFHR4	CHIT1	FRAS1	SPESP1	RP11-513O	MT1M	NPIPL2	ASIC2	GDF10
TMEM72	SLC1A7	SHD	FER1L6	HLA-DQA2	FOXQ1	IGKV4-1	SOX9	ADCY5
KRT5	NTS	SCN7A	ZNF99	TYRP1	EPO	ADH1B	VSX1	BTC
AC144833.	C10orf112	NEUROD2	KLHL34	SLC4A9	APOF	MARCO	DCC	PRELP
IGHV3-48	TRIM7	CD70	FUT9	IGHV3-23	ENSG0000(	IGLV3-27	BHMT	MUM1L1
IYD	HPN	EREG	RP11-2N1.:	SPINK13	SULT2A1	IGKV1-9	CYP21A1P	CNTN4
CYS1	PKLR	ENSG0000(	SLC30A2	PRODH2	HEATR7B1	FXYD2	NPTX2	DCAF12L1
KIF1A	IGLV7-43	C6orf141	TMSB4Y	SLC2A2	ECEL1	MUC4	AC109309.:	EYA1
IGKV1-6	MAGEA11	HOXA10	GLB1L3	CYP4F3	SLC6A19	MYEOV	BCL11A	STMN2
LEMD1	SLC38A5	GS1-211B7	SGCD	NKAIN4	C3P1	NXF3	AC079776.	IGSF1
SULT1C2	SBSPON	ZNF560	FAM189A1	IGHV1-46	ENSG0000(	CAPN9	PIANP	PITX2
PROM1	BEX2	SFRP5	FOLR1	MYO3B	ANXA13	SEZ6L	PLCH2	KERA
TNS4	TDGF1	MMP12	SLC6A20	IGKV3-15	COL22A1	ENSG0000(	DUSP2	SLITRK5
CPB2	L1CAM	PABPC5	KCNU1	RPS6KA6	CFHR3	IGHV3-13	PCDH15	ENSG0000(
RP11-116D	GREM1	TACR1	SMTNL1	CD5L	AHSG	KLK11	KCND2	IGHV1-2
MTTP	B3GNT7	TNR	MTND1P23	ENPP7	COMP	PLA2G2F	RSPO1	IGHV3-21
DIO1	LINC00668	PITX1	C2orf72	AGXT2	IGHM	COL10A1	MDGA2	ZNF560
FAM99A	IDO1	RP11-328N	CDH3	IGLV1-47	UGT1A2P	C4BPA	ST8SIA2	CYP11A1
IGHV1-2	SHISA6	MNX1	MST1L	TCEAL2	UGT1A3	FCRLA	SCARA5	FOXL2
CD19	HOXB8	TRDN	CTB-52I2.8	LGI4	PNCK	IGHV4-59	STAC2	CXCL13
SERPINA10	CCL25	NNMT	CLDN10	CNTN3	FRAS1	IGHGP	CALB2	HSPB7
APOA4	EN2	C7	TDRD5	KCNJ13	NAT2	CEACAM18	FOSB	DIO3
VWA2	BEST2	MFAP5	SLC38A8	C19orf21	VNN1	IGHV3-49	C10orf85	KCNQ2
SYT13	PEG10	IGHV3-33	CA8	CCL19	NNMT	IGHV3-23	SH3GL2	NTNG1
LTF	SLC6A4	SPOCK3	ENSG0000(	EPN3	TRHDE-AS1	IGLL5	IGLV2-11	IGHV3-11
PGLYRP2	KRT40	NEUROD4	NTNG1	IGHD	UGT2A1	IGKV2D-29	ELMOD1	ROS1
RP11-115D	PPP2R2C	CHRNA9	ABCA13	SCNN1A	MFAP4	IGLV4-69	ACVR1C	SCG2
AGXT2L1	COL9A3	HOXC9	EYA4	TGFBI	DSCR8	PAEP	GYG2	FBN3
RP11-242J7	IGHV2-26	ASIC4	PHF21B	TF	DQX1	IGHV3-33	NELL2	DLX6
FXYD2	COL11A1	IGKV1-9	FRMPD4	IGHV4-59	UGT1A1	LINC00668	FGFR2	RNF212
IGHV3-21	VNN1	EN1	CHIA	LINC00617	KCNB1	PCSK1	NAT8L	HHATL
HOXB9	IGKV2D-29	CACNA1B	ENSG0000(	IGLV6-57	CFHR1	IGLV2-23	HK2	DMRT2
RALYL	SPINK1	PPP1R1B	CTD-2023N	POF1B	UGT2B15	IGHV4-31	NRXN1	SFRP5
VSIG1	GPR143	SHISA7	TFAP2B	IGLV2-11	TMPRSS3	CREB3L3	CRYBA2	PPP1R1B
GABRB3	EGF	RP5-1177N	ZNF98	DNAH11	RP11-328J2	IGHV3-15	LRRC55	MYH14
ENSG0000(	IGLV5-45	STAC	MAB21L1	ATP12A	PODXL2	UNC80	ACTG2	ZIC2
NPTX2	RNY1	IGHV4-59	RBBP8NL	ENSG0000(	RP11-438N	FEZF1	RPSAP53	DMKN
U1	ADTRP	GABRA4	SLITRK4	ENSG0000(	SIK1	IGKV3-11	CHST9	SOX11
CASR	SLC30A2	CCDC164	RP11-627G	B3GALT5	PAGE5	IGLV1-36	SYNPR	PAGE2
SH3RF2	RP11-3L10.	DAPL1	MASP1	LYPD6B	ENSG0000(	PTPRN	WNT10A	RP11-526J3
CXCL6	NXF3	SLC13A5	MAG	ENSG0000(	IGHA1	ANGPTL7	FAM163B	CNR1
GDA	ISX	CCL13	FAM190A	CCL20	GBA3	IGHV3-21	IGLV1-44	BEX1
DPCR1	SCARNA7	AREG	ENSG0000(	IGKV1-9	RP11-443P	FIBCD1	GRID2	PRSS12
F5	PTPRD	ATP10B	LINC00588	CADM3	B4GALNT4	IGLV6-57	ABCC8	FAT3
TNNI3	UNC93A	CNTN3	MMRN1	C16orf11	PADI1	MAFA	CCL19	TTTY14

ACTBP12	FAM189A1	TFPI2	NRG3	CLEC18C	IGLV3-25	CDH17	PON3	CPZ
TCL1A	PSCA	ENSG0000(	AC090044.	TMEM61	ACTN2	LINC00617	SLC27A6	KNDC1
ANXA13	PHGR1	ENPP5	MRAP2	DAO	ANKFN1	NOL4	TPH1	SCN7A
C6	SYNE4	IGHV3-21	ESRRB	C14orf180	DSG1	AQP12B	IGHV3-15	KCNK3
C19orf33	FOSB	CELF5	SLC12A1	PKP3	DCN	SULT1B1	USH2A	STAC2
GRM5	HOXC11	RP11-80F2:	SERPINA4	ANO5	RP11-403I1	ARX	RP11-863P	MAGEA12
ENSG0000(	CWH43	MEG3	C6orf222	C12orf56	GABBR2	FABP4	GADL1	KCNK2
ENTPD3	THBS2	IGKV1-27	ENSG0000(	CTD-2008P	SFN	PPP1R1B	LGR5	PCYT1B
ATP13A4	MT1E	RP1-310O1	ELAVL2	IGLV3-10	TSPAN8	IVL	SPAG6	RELN
IGHV3-53	CNTNAP2	EBF3	ZFHX4	IGHV3-21	KRT20	CARTPT	BANK1	NPY
CTD-2005D	IGHJ3	SYT1	CECR2	ZFY-AS1	IGLV2-11	NR1I2	SLC6A2	CDH1
GYS2	DMRTA2	CNTNAP4	PCDHA12	VNN1	RP11-323N	CCL21	NR1H4	FNDC1
UGT1A4	KLK8	SLITRK4	GRIA1	UCHL1	RGSL1	LRRTM3	IGHV1-24	C12orf28
GALNT14	CXCL13	SULF1	SLC4A4	TTTY14	AC018865.	AC013275.	AREG	DLX6-AS1
MT1G	RP11-329L(	PCDHGA10	11-Mar	APOH	IGHV1-2	CRISP2	NEUROD1	C16orf89
RP11-1C1.6	FGF19	BAI1	SSTR5	SLC39A5	CDH15	ONECUT1	IGHV3-33	DAB1
CHRND	SPOCK1	MEDAG	LRP1B	ADH1C	CFHR4	PLIN4	AC110619.	ZFP57
IGHV3-33	FEZF1	LINC00645	CEACAM7	BEX1	FAM151A	IGLV1-47	TM4SF4	KCND3
TDRD5	SLC39A5	C5orf49	CCL21	FCAMR	TERT	ENSG0000(	KCNG3	HOXD13
CDH17	NRN1	FAM180A	LRFN2	ALB	ASCL1	GAST	IGHV1-18	CNTNAP2
FAM83E	CAPN9	TMEM196	SNCAIP	PITX2	CCL20	NKX6-2	COL19A1	IGLV4-69
AC073479.	CPA6	HCN1	BTBD11	IGHV4-34	ADH1B	GJB5	SLITRK3	CCL13
GBA3	ENSG0000(	KSR2	CCDC178	CALCA	RP11-372E	ITGB6	TMEM229/	HOXD11
PLA2G12B	GPR128	LINC00507	PLEKHD1	ENSG0000(	HP	ARHGAP40	IGHD	FGFR2
PEG10	IGLV2-18	ELAVL2	TPSAB1	IGHV3-15	KIF1A	APOBEC1	SPP1	DCAF12L2
RP11-397G	APOE	NTSR2	PCDHA10	DGCR5	IGHV3-21	IGHV1-46	ANKRD2	DRD2
CALB2	APCDD1	SST	LINC00659	TMEM72	PRG4	IGLC7	FAM153C	KANK4
KRT20	CXCL11	DAO	CTD-2081K	IGHV3-49	VTCN1	SCEL	CD5L	WNT10B
IGHV1-24	CILP	OTP	MGST1	IGKV1-16	IGHV3-11	LY6K	GSTA3	C3orf72
ENSG0000(	KBTBD12	CELF3	CSDC2	IGJ	GPX2	CALHM3	CDCP1	RP11-401P
KCNH6	HOTTIP	NKAIN4	GCGR	RP11-326C	COX6A2	GPRC5A	COL4A6	DIRAS1
CALCB	RP5-1052M	PCDHGB6	ANKRD34B	IGHV3-11	KCND3	WISP3	ATP2B3	LMX1B
EN1	DIO3OS	VGLL2	ENSG0000(	CDHR2	CYP11A1	SFTA2	IGHV4-39	PNCK
PLA2G2A	KCNJ3	L1CAM	PSG9	ENSG0000(	HS6ST2	IGLC3	TDRD5	IGHD
SLC44A5	IGLC7	FOSB	PRAMEL	HAVCR1	CNTN1	IGKV1-17	EFNB3	KB-1448A5
SLC38A3	KIAA1324L	MRC1	C1orf173	IGLV4-69	ENSG0000(	VWA5B2	LPPR5	CCDC8
GPRC5A	GCNT3	NPTX2	SHD	GLYAT	RFPL4B	CD79A	BMP5	CDH2
RP11-674N	VIP	HOXA-AS2	AC109586.	KRT7	EGFR-AS1	APOB	IGLV1-47	NKX6-1
FAM3B	CA2	ZSCAN1	DCN	NTNG1	PAGE1	ENSG0000(	PRSS12	WSCD2
MUC13	SOSTDC1	CPXM2	IGKV4-1	EEF1A2	GDNF	PLIN1	ENSG0000(	IL17B
DACT2	FABP2	WSCD2	ACVR1C	SIK1	FOXP2	ZFP57	ENSG0000(	PCSK1
BTNL8	CYP4X1	GABRB2	CHI3L1	UGT2A3	ELOVL7	IHH	RMST	TAGLN
UGT2B15	TPSG1	RASSF9	IGKV3-20	WISP2	DPT	SLC34A2	ADCY8	LMX1A
RP11-704M	CADPS	CBLN2	RP11-500B	AC006227.	FER1L6	DQX1	RP11-807H	SPOCD1
BHMT2	DUSP4	RIMS2	RBFOX1	LAD1	TTTY14	SLC7A14	PTPRO	IGKV1-6
PKP3	GLRA2	C1QL2	TMPRSS11I	C12orf39	PPP1R1A	GKN2	ADRB1	NLGN4X
HPGD	FOXD1	ENSG0000(	EGFR-AS1	GPRC5A	LRP2	VIP	PITX1	CILP2

ANXA8	OGN	CELF4	CALN1	SLITRK2	ACADL	IGHV3-73	TCEAL6	BNC1
WFDC2	ALPPL2	FGFBP2	SOX8	SLC38A4	RP11-495P	RP11-211G	CDH4	RSPO2
HAMP	TESC	NPY	CHIT1	TMEM179	TGM3	SLC16A12	SPINK4	SIX2
ENSG0000(	CXCL10	BTC	KLK11	PKP1	CTD-3110P	AC011306.	SPHKAP	PPP1R1A
HULC	IGHD	EMILIN3	IGLV2-11	TACSTD2	NR1I2	ENSG0000(	SLC30A3	NTRK2
MYOM3	PCK1	TMEM132I	CTA-392E5	TCL6	NPFFR2	RP11-48O2	MUC12	DIO3OS
WNT7A	ENSG0000(	CDH19	TRPM6	FAM151A	LRCOL1	CXCL6	TMEM190	GLIS1
OCA2	hsa-mir-67.	PDGFRA	IGLC2	PCDH10	RP5-1154L:	CA4	WBSCR17	GALNT5
IGLV8-61	FBXO2	KCNJ3	TMPRSS4	RANBP3L	SEMA3E	NTS	ANO4	FMO3
CD5L	PRELP	RP11-208G	AQP5	OLIG1	FXYD1	C11orf86	RP11-46C2	PEG3
SYT8	FABP6	RP11-343H	GSTA3	MST1L	SLC39A5	GPR128	HTR3A	PDLIM3
AL773572.:	O3FAR1	RP11-357H	RP5-1043L:	LGALS12	FXYD3	IGHV4-34	NAT16	TNMD
GLYATL1	ABCA12	HOXD9	FGF7	MFAP4	GUCY2C	SCNN1G	C22orf42	SCUBE3
FCER2	C5orf38	RP11-92A5	RP11-178A	IGF2	HOXA10	GUCA1C	DAPL1	SHISA3
ZIC5	MMP12	RP11-148L:	KCNMB2	SLITRK5	BBOX1	LRRC31	CST1	FGF7
PLA2G2D	RP11-750B	CHI3L2	IGKV3-11	OPCML	APOA1	SLC2A2	CBLN2	PLD5
RP11-211G	ANXA10	FNDC1	IGLV2-14	FAM153C	EVPL	TRIM31	SFRP2	CCL19
RP11-522B	SSTR5-AS1	PLA2G5	MMP1	SLC7A13	UNC93A	CASR	SV2B	MYBPC1
CYP4F3	RNA45S5	SNCB	LAMA1	DPYS	SERPINC1	IGKV1-16	CTD-2194D	IGHV4-34
HABP2	ACTG2	MET	SSTR5-AS1	FAM3B	RDH16	AC006262.	ALOX12B	CECR7
MOGAT2	DPP10	ENSG0000(	RP11-680F:	IGHV3-33	ALDOB	IGLV1-51	BAI1	TRIM63
ANXA10	SERPINA1	CXXC11	ABO	IGKV1-17	IGHV1-46	PADI3	IGHV4-59	CSAG1
ATP10B	SLC28A3	IL6	PPP1R1B	RP3-523K2	CTD-3098H	IGHV2-70	LINC00643	ANO5
IGKV1-17	SLC22A11	CBLN1	TMEM132(	KLK6	IGF2BP3	ALPI	HPCAL4	RYR2
GJB5	SAMD5	SLC7A3	MMP26	TMEM207	ENSG0000(	IGLC2	TNNT2	FAM189A1
IGHV3-49	PROM2	LILRA4	PRKG2	BBOX1	WNT4	RP11-1220	RSPO2	MYL1
EEF1A2	HABP2	ACAN	TMEM30B	FOXI2	RP3-340I3.	AC073218.	AC244102.	IL11
NEB	KIAA0226L	CECR7	ENSG0000(	TM4SF5	MUC15	CGB	SLC47A1	CDH15
GUCY1B2	HLA-L	OGN	CAMK2B	FBN3	IGSF9	FAM129C	ZMAT4	CMA1
MMP7	C7	CBLN4	MTUS2	AFM	RAB3C	IGHV1-58	MNX1	MOG
HOXC10	RPS6KA6	THNSL2	CYP3A7	GALNT5	AGXT2L1	IGHV3-64	IGLV3-25	MSI1
IGFBP1	SLFN13	NKX2-1	PGR	CLDN2	RAB25	SSTR5-AS1	GDNF	HRASLS5
PAQR9	IGLV3-27	GBX2	ENSG0000(	SOWAHA	IGFALS	CHST9	FRMPD3	NKD2
MUM1L1	RP11-436H	HOXA11	BEX1	LOX	PRKY	RP3-340N1	MT1F	ALDH1A3
C5orf38	ACTBP2	WT1	U47924.27	FRMD1	RP11-20D1	CDH22	CHRDL1	CXCL9
RP11-127I2	TBX18	MDGA2	PART1	ST8SIA6	PROL1	IGKV2-24	RP11-260A	MYH2
RBBP8NL	FN1	MOXD1	MCCD1	OR2I1P	IGF2-AS	GATA5	RP11-707A	PDGFRA
HLA-DQA2	CTD-3035D	KCNT1	EHF	PTPRN	RP11-81H1	TMEM132I	IGKV1-9	AC108142.
RP11-554A	MYEF2	PTPN5	PRR15	CTC-327F1(	CYP2C9	F11	PPP1R1A	FAM101A
TMPRSS3	ABCB1	GLRA2	VWDE	GRIK3	PI3	SOX21-AS1	GREM1	AQP4
BLK	RNU5A-1	GREM1	RELN	ENSG0000(	HTR1D	SLC4A4	IGKV3-15	TMOD1
COL11A1	SERPINA10	PRKG2	MTND4P24	GUCA2B	ZIC5	ENSG0000(	MIR770	ST8SIA2
SLC22A9	LGR5	DACT2	PACSIN1	IGLV10-54	GLRB	RP11-424G	ENSG0000(	SERPINB5
PDZK1IP1	IGKV3D-20	RNU4-1	FRG2C	CHST9	RP1-27K12	TMEM63C	PCP4L1	MEGF10
IGKV1-9	PKP1	ENSG0000(	RP11-371I1	IGLV1-51	AC068535.	MYO1A	REN	PI3
GUCA2A	AQP9	HES5	TRGC2	STK33	IGJ	IRX2	SPON1	FAM180B
SULT4A1	ENSG0000(	ALPK2	TCL6	ENSG0000(	REG1B	SERPINB4	TMSB4Y	GSTM5

C9orf152	SOX14	RBFOX3	VAT1L	APCDD1L	MUM1L1	IGHV3-48	LY6D	COL6A6
CLDN18	PHYHIPL	AQP9	IGHV5-51	EPB41L4B	ENPP5	GPR119	PART1	MYH7
PPP1R14C	MAT1A	CDKN2B-AS	FAM3B	LINC00473	CTC-505O3	IGKV2-29	CRHR1	IL20RA
IBSP	F5	PCDHGB2	GREM1	SMOC1	COCH	KIF1A	CALY	ABCA8
FCRL2	MT1G	PI16	KCNG1	FGF7	MAP7D2	ATP10B	GLT25D2	LYPD6B
IGHV4-34	MAGEA12	WISP1	C16orf11	REG3G	VIL1	SH3GL2	SLC9A2	SLITRK2
IGFL1	DMKN	COL21A1	PTGES	STAP1	TDGF1	IGHV3-72	SLC22A3	ENSG0000(
APOC1P1	HOXB9	CAMK2A	ODAM	SORCS2	RP11-436I9	COMP	ATP4A	IGF2BP2
ADAMTS16	RNU5B-1	HRH3	CTC-504A5	CNTNAP5	UGT2B10	ALDH3A1	AGRP	MXRA5P1
SLC6A20	C4BPA	PCDHA11	CECR7	RP11-706O	TDO2	APOBEC2	DHCR24	EPYC
CCL21	TFF3	SLC7A10	TERT	TMEM82	FLNC	KCNJ15	FAM150B	RP4-555D2
FAM135B	PNLIPRP2	RNF128	CADPS	RP11-626P	FOLH1B	GPR110	UNC13C	CYP4B1
SLC28A3	PLAC8	ERMN	PRSS16	GAD1	C1QL1	CD19	NXPH1	SPOCK3
GC	RASGEF1A	BTBD17	RPL13AP17	RP11-526F	RASEF	FABP6	GRIK3	FUT9
AC018804.	SATB2-AS1	DPP10	OR2I1P	GBA3	RP11-6B4.1	SFN	KCNH8	MEST
GCGR	AMH	ATP1A3	GRM1	ALDH8A1	MCCD1	GJB6	RP11-750B	CDH18
POPDC3	NKX2-1	FXYD7	RP11-1260	GUCY1B2	HPR	SERPINB2	CACNA1A	GAP43
G6PC	AC018738.	AC095067.	TIMD4	SLC6A13	CDH16	IGF2BP3	LINC00617	GRIA2
IGHV2-26	ASPN	KCNJ16	SFRP4	RP11-742N	HMGA2	CIDEA	MOXD1	PAX1
SHISA9	ENSG0000(	TCF23	LRRC14B	CKMT2	B3GAT1	SERPINA4	RP11-401P	GDF6
KCNK9	ALPK3	CHD5	CALCR	BMP5	RP11-495P	IGFL2	GPC3	HLA-DQA2
SCARA5	PLA2G4A	NOS1	RP11-834C	FUT6	GBP7	IGHV3-74	AC092165.	RBP4
AKR1B10	HSPB6	NKAIN1	CPEB1	DEFB1	IGLV3-10	IGKV3-15	RP11-738O	ISL1
SLC34A2	HIST1H1E	CXCL3	C14orf105	BHMT	NPW	KBTBD12	GRM4	MYH1
PTGFR	RBP1	SLC6A7	PPM1E	SPOCK1	IGHV1-24	LHFPL4	CTD-2195N	TRHDE-AS1
TGM3	AC016708.	SOX3	FAM150A	RNF186	IGLV6-57	ENSG0000(	RXRG	TNXB
PVRL4	IGLV9-49	GALNT5	IGLV3-19	CHGB	GNMT	IGKV6-21	ENSG0000(	ALPK2
AJAP1	OTC	CLCA4	ABP1	F5	FOXN4	S100A14	AC005082.	ADAMDEC1
FOXJ1	PIPOX	MXRA5P1	HABP2	ADAM18	IGKV1-9	PKHD1	PCDHA4	SLN
IGHV3-74	SLC19A3	PRRG3	CXCL14	AC016708.	RP11-294O	LINC00261	FABP4	TPTEP1
GPR115	WISP3	SPON1	SCGB2A1	PTGER1	GABRA2	LRRC15	FGF9	CCBE1
FAM83A	FNDC1	AZGP1	CTD-2193G	SLC5A10	SYT9	IGHV3-53	HOXC10	ENSG0000(
FCRLA	IL33	RP11-424G	RP4-799P1	ESRRB	PAQR5	PRSS33	CALN1	IGKV1-16
FGF21	HUNK	WNK2	SLC16A12	NUPR1L	SIM1	IGKV3D-20	CACNA1E	CLEC4G
COL9A3	CTB-118N6	DLX2	INSRR	GRIN2A	IL8	IGLV7-43	PCDH8	NCAM2
SLC30A2	PLCB4	IDO1	FXYD2	FABP4	SLC30A2	PAPPA2	LINC00461	DARC
IGKV1-27	RP11-372E	TMEM132E	STEAP2	GDA	RP11-30J2(	IGLV7-46	AL122127.:	SERPINB7
AOX1	PTGS2	COL3A1	CRABP1	ENSG0000(	LOXL4	EGF	CHRNA2	TMEFF2
IGLV4-69	CNN1	CUX2	CNTNAP2	HOXB9	ZG16	ZBED2	NKAIN4	ANO4
KCNJ16	VSTM2L	VWA3A	CNR1	FREM2	RP11-157P	ZG16	EPHA6	MOXD1
VSTM2L	CTD-2377D	CADM3	EMX1	PDGFRA	PGLYRP2	UGT2B7	AP001046.	PAX9
SEBOX	TMIGD1	KCNG1	UGT2B7	PPP1R14D	ADAMTS16	GYG2P1	CBLN1	ADAM23
SERPINB2	SLC6A19	HOXD8	CNTFR	RPSAP53	SLC2A5	FUT9	LRRTM3	MMP12
CD1A	POSTN	BARX1	GSTM5	SERPINA4	GLT1D1	CRCT1	KCNG1	CILP
PGC	NPIPL2	KIAA1644	GLRB	LSAMP	KCNJ3	NCCRP1	CES1	KLK4
NLGN1	ENTPD8	ABCA13	CTD-2083E	IGLV3-9	CACNA1H	RP11-89K2	HOXB8	CLVS2
B4GALNT2	RPSAP53	HOXC-AS1	RP3-416H2	FAM153A	C1orf106	IYD	KCND3	NOTUM

TFR2	CTD-2666L	HAPLN2	C1orf116	OVCH2	SLC28A1	SLC8A2	RP11-375B	TNFAIP6
PRKG2	SCEL	IGHV1-46	ASCL4	RP11-165D	RP11-152P	XXbac-B44	PAX8	GLB1L2
ABP1	C11orf86	PLEKHG4B	CYP26C1	SLC16A9	AQP6	MUC21	CECR7	NPAS4
CYP2C8	NKAIN2	FAM123C	RYR2	CYP2B6	RP4-763G1	ZIC2	MAGEE2	HR
VAT1L	ISM2	GRIN2A	LEF1-AS1	IGHV1-69	S100A14	ARL14	MAOB	EPPK1
WDR72	ENSG0000(	RP11-32K4	C12orf77	CLRN3	GRIA3	IGHV3-43	CLMP	TENM1
AQP9	SOX8	SPHKAP	RP11-255M	AP000330.	COL11A1	SDR16C5	RBFOX3	SLITRK6
RP11-522B	GAS1	ENSG0000(	AC011298.	FMO1	SULT1E1	MAB21L2	IP6K3	LRRC4C
KCNK2	IGHV4-28	SSTR1	CRLF1	ENSG0000(	ATRNL1	B4GALNT2	RP11-320G	GRIK3
RP11-1151	CHST5	MAPK15	COBL	HNF4A	SLITRK3	RP11-742N	LUM	LHX9
MME	ALDH1A2	AP000688.	ENSG0000(	PRKG2	ASPHD1	CILP	PTGDS	SPRR2F
TINAG	PCDHGA10	ENSG0000(	SYTL5	IGLV7-46	SYT1	TINAG	DLX2	MYO18B
PHACTR3	IFI44L	DMRT2	HOXA11-AS	HSF4	RP11-371I1	CYP2C9	PTHLH	TYRP1
MFSD6L	SLC18A1	TMEM130	TMEM178B	MUC13	SLC13A2	ODAM	COX6CP1	RASEF
CXCL14	DLX6-AS1	DDIT4L	CPA3	TUBA3E	RP11-328K	SERPINA10	TUBBP5	EMID2
GAD2	BRSK2	SLC6A17	ISM2	HAPLN1	CYP39A1	RP13-870H	SOX11	VIPR2
UGT3A2	ERAP2	CAMKV	EMX2OS	RP5-1120P	TBX4	KIRREL2	IGHV1-46	ALX4
PSCA	RP11-326C	MYBPH	PCOLCE2	SLC22A13	IGLV4-69	CDKN2A	CDH9	METTL24
C2CD4A	CYP1B1	FGF10	ENSG0000(	IGLC7	ENSG0000(	ROS1	SLC38A11	SAA1
SCG3	MZB1	SLC8A2	SLC5A1	RAB42	AC004862.	CHRDL1	TRHDE	CNTN3
TRHDE	RBP4	CSMD3	IGKV1-5	RIC3	MAFA	SEBOX	HMX2	PTPRN
MMP12	SLC26A2	CALB2	CRYAB	RP11-317M	APOA2	VGLL1	GALNT9	EYA4
INS	KRT5	VAT1L	LINC00507	HTR6	PRSS16	DPP6	HSD3BP2	PPP2R2C
AKR7A3	APOD	ENSG0000(	NOS1	NEGR1	IGLV8-61	IGHV2-5	FSTL4	IGHV3-74
LAMP5	DLX3	IGLV1-51	AC005077.	GP2	AC073218.	MOGAT2	ZCCHC16	MORN5
KIAA0125	SCARNA10	DPT	USH1C	AZGP1	SLC1A7	RP11-325F	ANKRD34C	OGDHL
SEC14L4	MTND2P28	HMCN2	SLC39A2	RN7SK	GAL3ST1	AGR2	ARC	SH3GL3
TNNT1	C3	PPP1R1A	ADRB1	IGKV1-6	MTND4P12	HOXA11	FAM222A-A	RIMBP2
ENSG0000(	NOX1	RP11-469N	RP11-167H	PRND	MPPED1	IGLV9-49	DDIT4L	PLEKHG4B
SLC14A1	KIAA1549L	LY6H	TRIM72	FAM150B	GABRA3	SERPINA5	WIF1	SOX8
B3GALT2	TAC1	RP11-471J1	ASCL1	ENSG0000(	CTSE	RP1-310O1	MAFA	IGHV3-49
HOXC9	HYAL1	SLC6A15	FLG	CRTAC1	RP11-261N	ORM1	OGN	VGLL3
ATP2B2	SERPINA6	TFAP2A	RP11-469N	PTGER3	VSTM2L	HAS1	NTNG1	HIF3A
MT1H	CR2	PCDHA6	UBD	MMP9	FOXJ1	RP11-101E	RP11-98L5.	DBC1
CA8	OXGR1	RP11-275H	DUOX2	IYD	EGF	LBP	CYP26C1	NPTX1
RTP3	NTSR1	UNC13C	HOXC-AS3	CDH9	ARG1	AC021218.	PKIB	IGKV1-17
HSD17B13	ARSE	TENM2	RP11-510D	SHISA3	TFF2	MMP13	SDR42E1	COL9A1
STRA6	ADAMTS15	OTOS	RP11-154C	ENSG0000(	COL4A5	MXRA5P1	VSTM2B	KIAA2022
S100A1	SPON1	SEMA3A	IGHV3-23	PTPN20A	HHIPL2	DEFA5	CCL28	REN
CDO1	KLHL13	KIAA2022	IGLV2-23	KCNK9	RND2	CTD-2015G	HTR5A	GYG2
CLDN10	IGHV3-13	ARHGAP36	IGLV3-25	PSAT1	C1orf64	PIWIL1	PLAC2	MAPK10
CTD-2587H	ENSG0000(	CES1	CRIP3	FER1L4	PRR15L	AC011298.	MKX	WFDC1
REG3A	CLC	SCN9A	ENSG0000(	MUM1L1	TMPRSS15	MLK7-AS1	ADORA1	OCA2
AC013275.	C11orf92	AQP5	CHRDL1	C3	SYT8	IGHV4-61	FERMT1	ALDH1L1
FMO1	CTD-2566J1	CHRDL2	SLC38A4	CUBN	TFF1	ADCYAP1	RP11-728G	CSRP3
SIK1	CTNNA2	TAC3	ADH1B	LMX1B	ISLR	HOXA10	NTRK1	KIAA1644
SLC22A31	FRMD1	FAM110C	CREG2	IL1R2	GHRHR	HOTAIR	B4GALNT4	RORB

EN2	REEP1	CYP4F12	KCNG3	CCBE1	C2orf54	ACTL6B	SLC16A12	FREM2
BAAT	AC092165.:	GMNC	NR1H4	LINC00278	DIO3OS	LINC00671	PACSIN1	CXCL1
IGKV3D-20	ST6GAL2	LRFN5	SCNN1B	SYT10	ALDH1L1	UPK3B	SALL1	DEFB1
HHLA2	ABCC2	SH3GL2	UNC13C	BASP1P1	SCGN	FEV	PTPRD	SORBS1
IGHV3-43	CARD11	NELL1	TMEM246	DHRS2	CNTNAP2	CIDEC	PRLH	SCN3A
CHST9	STRA6	TCTEX1D1	TWIST1	RASL11B	AC005550.	C1orf110	RP11-438B	FOXF2
CRISP3	GCG	C9orf24	TMPRSS13	TRHDE-AS1	ACTL8	ZNF736P9Y	MGARP	GATA4
CDH19	MUC16	MCTP2	NXPH2	INPP5J	ZIC2	NPHS1	SNTG1	SNAP25
TMEM151A	CD79A	FEV	AC024560.	CYSLTR2	KCNJ16	HMGA2	IGLV3-1	FAM167A
RP4-765C7	SYNPO2	PENK	XK	UGT1A10	RP11-279F	EN2	HHIPL2	PGM5
ENSG0000(	FABP4	LHFPL4	HOXA9	SLC14A2	MBL2	PITX1	AVPR1A	SSTR1
IGLV3-9	TG	C12orf39	OR1L8	CHL1	IGKV1-17	AC244230.	NR0B1	PLXNA4
IGLV1-51	RP11-673C	NHLH1	GHR	CA4	SSTR5	LRRTM1	C21orf88	KBTBD12
DCDC2	HIST1H1B	CDH7	GPR110	NR2E1	RP11-587P	ACTG2	MRAP	WNK2
RP11-469A	TMEM211	GAL	RP11-865B	MOGAT3	MAGEB17	CACNG4	SYTL5	FOXP2
FAM3D	KRT75	PTGFR	KRBOX1	REG1B	OCA2	CCL20	LUZP2	ADAMTS8
SULT1E1	CTD-2377D	NKAIN2	FAM110C	IGKV1-27	LDLRAD1	MSLNL	C1QL3	RP11-429E
PODXL2	SMOC2	COL28A1	HAPLN1	GPC3	PYCR1	CHST4	SLITRK2	VAT1L
CLDN6	SEMG1	HOXA3	AKR7A3	GPR37	ASNSP1	FAM123C	MCTP2	TMEM59L
CHIT1	SYN3	COL23A1	ZNF208	SOST	ENSG0000(	C1orf127	AL022344.4	PLXNB3
PCDHA11	RP11-267A	NXPH1	LINGO2	SYT9	COX6CP1	MEP1B	ATP8A2	SH3GL2
CD207	HOXC6	OTX1	SPP1	LCN2	CAMK2B	GRM4	RBFOX1	ENSG0000(
AC016683.	AC018865.	NDST3	SCN7A	S100A1	CXCL14	IGFBP1	TRIM29	CAP2
SIGLEC15	D86994.2	SKOR2	LRRC37A4F	ELFN2	POSTN	GREM1	GABRA2	RP4-765C7
ACMSD	OR2I1P	RP11-958N	DPYSL5	CLEC18A	CAPN13	IGKV1-6	CAPN13	TRIM55
SLC13A1	ODAM	MPPED1	KIF1A	AC092653.	IGHV3-49	RP11-539E	PCDH7	ABCC8
ASPG	EMB	OTOG	HTR3B	MYH14	GDA	EPS8L3	MYO3A	DNAH11
IGKV2-24	FCGR3B	ATP6V0A4	KCNK1	TRIM71	PTGES	SERPINB7	ILDR2	MYBPH
HAVCR1	OGDHL	CAMSAP3	LY6K	SERPINA6	PDGFRA	RP11-1L12.	ENSG0000(	BMPR1B
CRHR1	ERP27	TUBB4A	RP11-742N	IL6	ETV4	PLA2G2D	SIX3-AS1	PTGES
NEURL3	SOD3	LINC00617	RGS7	AQP9	IGKV1-16	RIMBP2	BCAT1	GRIK2
KCNJ15	CPLX2	IGLV4-69	PDE3B	SLC28A1	GATA5	FAM150B	LY6H	LINC00645
RP1-27K12	ALDH1A1	IGHV3-11	FOXQ1	CLMP	TUBBP5	PCK1	RP6-109B7	ANGPTL7
CA4	SAA2	LINC00648	STAC2	NPC1L1	SAA4	SLC9A3	VWDE	RP11-400K
ABO	MYEOV	DARC	IGHGP	MGARP	DNER	PNCK	RXFP3	PTH2R
SPAG17	POU5F1B	WT1-AS	HOXA13	NCAM1	CYP4F2	AGR3	DLGAP2	TBX5-AS1
GYG2P1	SLC38A4	RN7SK	C5orf49	KIAA2022	GPR158	LGALS7B	HCN4	TMEM119
GFRA1	VENTX	PTPRT	CPA6	KCNK2	FAM134B	KIAA0125	IGHV3-11	RSPO3
SCNN1A	TRPV6	GALNT9	ALDH3B2	ESRRG	MCOLN3	ENSG0000(	IL8	SYT14
TFF3	TMEM252	NGB	STAB2	CTD-2335A	NAA11	HP	PKHD1L1	ASXL3
PKHD1	BTNL8	AC244102.	OGN	IGKV2-24	AC104135.	C6orf222	IGKV1-17	AR
ENSG0000(	SYNM	PVALB	CTD-2298J:	CES3	GPC5	OTC	RP11-500B	TPSD1
SERPINF2	AC016739.	IGF2	MMEL1	IGHV3-48	RAB3B	ENSG0000(	COCH	CNNM1
CLDN2	NMU	FAM216B	AP001626.	IGLV7-43	CLGN	KRT23	RBP3	IGHV4-31
ENSG0000(	EPYC	HPR	RTP2	IGF2BP3	RP11-434D	GJB4	IGHV4-34	PCOLCE2
CAPN8	AF064858.:	ONECUT2	SEZ6L2	KRT5	OGN	TM4SF5	AC009410.	GABRB3
IGLV9-49	PRKAA2	ARMC4	LINC00189	MTND1P23	CDH22	FCRL5	CTB-36O1.:	PSG5

RASSF9	CFD	SHISA2	ATP10B	NAPSA	CBLN4	DDC	PCDHA13	LSAMP
POU2AF1	ZBTB7C	IGLV8-61	CPA2	RP11-213H	DIO2	NKX6-3	POMC	HTRA3
MS4A8B	NEURL	FGF13	ENSG0000(	ENSG0000(	BICC1	SOSTDC1	PTGS1	IGLV3-9
SLC17A2	LRP4	ELAVL4	IGLV3-21	FXYD3	RP11-1220	CTD-2566J:	SYT7	CD300LG
CHST8	RP11-325F:	GPR26	ROBO2	AC003984.	MMP12	CNR2	IGHV3-21	SOX9
EMX1	CDH16	MMP3	LRRC15	ANKRD2	IGHA2	CAMK2B	TPSB2	PCLO
PHYHIPL	APOB	LIF	AC013472.:	DDN	MAPK4	KLK2	DOK5	ADAMTS16
VSIG2	IGFL2	HPSE2	IGJ	RP11-766F	UGT1A10	KRT7	SCN2B	HOXC10
GPR110	RP11-46C2	NRG1	HOXC10	CR2	KRT5	MYO7B	NDNF	FBXL22
PSG8	BARX2	RALYL	C2orf54	FIBCD1	C2CD4A	VSIG2	ASCL1	C6orf132
ABCC2	UGT2B15	RP11-438N	RP11-313J2	ADAMDEC1	CYP2B6	ST6GALNA(	AHNAK2	PTX3
GBP7	CCL24	SCNN1G	PCDHGA2	IGHV2-26	HFE2	HHLA2	PCDH10	ENTPD2
RP11-404P	CDHR2	ALOX15B	GPR27	NKD2	IGLV1-51	DARC	ALDH1L1	FAM43B
UGT2B17	F13A1	IGKV1-16	HOXA-AS4	TMSB4Y	FERMT1	GSTA1	PTPRT	RP11-424G
CNGB1	HCAR1	C5orf46	LAMA2	AC079630.	RP11-119D	WNT11	TSPAN19	IGKV1-27
ADH6	PPP1R9A	TMEM229/	CYP24A1	PART1	TFDP3	CYP2C18	TENM2	IP6K3
IGHV1-69	WT1	EMID2	TUBB3	RP11-133F:	FNDC1	VGF	KCNK17	MEOX2
PAK7	PADI2	WNT16	KIAA1239	COL4A6	RP11-701P	TCEAL2	ACTN2	MAB21L1
SLC27A2	FRAS1	RPS6KA6	NELL1	SLC23A3	GCNT3	RP5-884M(	STX1B	MSX2
TMPRSS6	IGHV1-58	LMO1	POF1B	TPSG1	AFM	NR0B1	CNTN2	FOXQ1
C6orf222	NKD2	VEPH1	FBXL16	GRIK5	EPPK1	NMU	HAP1	KIF5A
NPC1L1	PAX5	NEU4	LRRC52	SUSD4	C1orf186	PDZD3	PTGFR	PPP1R12B
UBE2U	POU2AF1	THBS1	AC013275.	CYP17A1	KCNK9	CGB8	AC007405.	LHX2
TMPRSS13	RP11-401P	KCNQ2	RP11-142G	GPD1	PCLO	PROM1	NLGN4X	NFASC
ITIH3	GALNT8	WDR69	SCN9A	ENSG0000(	PON1	TRHDE-AS1	BCAN	PCSK2
AHNAK2	CALB2	FLG-AS1	PROM1	RP6-191P2	KANK4	FOXJ1	IGKV1-6	CALN1
RP11-443P	UGT2B7	SEZ6L	SCG5	AP000688.:	ENSG0000(	C1orf81	IGKV1-16	FMOD
RP11-64D2	VAV3	TMPRSS7	PRAME	GDF6	CALCA	CTRL	HMX1	CASQ1
SLC22A10	RP11-706O	DPEP1	KIAA1324L	HHIP	IGKV1-6	ENSG0000(	RP11-402J(	BHLHE22
INHBE	MS4A8B	ENSG0000(	ASPG	EPHA7	GPR133	SIX3	RD3	SPEG
IGLV6-57	GABRB3	RP11-742B	MUC4	PI3	FAM169B	KRT81	DGKB	SAMD11
TTPA	IGHV3-43	GABRA2	CES1	SLN	RP11-946L:	UGT2B17	ENSG0000(	C10orf85
MYO1A	ADAM12	ENSG0000(	SNAP25	PTGDS	RP11-526J:	DPP10	IGLV6-57	GNAO1
RIMS2	KIAA1324	DSCAML1	LIPH	MZB1	MT1X	GRIA2	TLX2	RP11-344E
FCRL3	DKK1	PCP4L1	ENSG0000(	GLB1L3	IL20RA	SMOC1	FAM153B	PRND
RP11-798K	TNFRSF19	COL6A2	LCN2	RP11-211G	IGHV4-31	GJB3	FOXE1	AIM2
FETUB	COL9A1	GPR12	TENM2	SALL3	LYPD8	SAA1	ZDHHC22	F10
IGHV3-73	MS4A1	SEZ6	IGLV1-47	B4GALNT4	MASP2	C6orf58	SHISA8	TSPAN8
FAT3	RASSF10	ATP8A2	KCNJ10	ENSG0000(	ARSEP1	OGN	RP11-438N	MZB1
TUBBP5	MSI1	DMRTA1	IL20RA	SLC47A1	ADRA1A	IGLV2-18	CCK	HOXA13
PKP1	ADIPOQ	LGI3	SELE	IGHV4-31	DEFB132	SLC9A2	RP11-23P1	RBFOX1
IL6	NLGN4Y	SLC22A3	GSTM3	DIO1	IGHV3-74	HBB	XKR4	PAX7
KCNH8	KRT16	CKMT1B	RP11-666A	C11orf86	SERPINA11	KIAA1324	PABPC1L2E	UGT8
AC018865.:	MME	CLEC2L	RP11-362F	NNMT	APOA5	CP	UST	IGHV1-69
AGMO	KIF26A	KLHDC7A	LINC00494	TRHDE	PART1	TRIM54	GAP43	DUSP27
CDHR5	IGLV1-36	UNC5D	EMX2	IGHV3-74	SLC7A10	BCAS1	VGF	LGI1
TMEM179	ID1	IGLV1-47	CALCB	DPP6	PKP3	CAPN8	ARMC4	MKX

ZIC1	C2orf54	S100A8	GRIP2	PADI3	RP11-401P	TM4SF4	MMP1	SDK2
AGR3	SP5	SGCD	PDGFRA	SLC17A2	APOC1P1	IFNE	KCNA4	THBS4
PLEKHG4B	HSD17B2	SERTM1	ASB11	RP11-154C	ILDR2	MOGAT3	LBX1	UCHL1
ADRA2A	ARHGDIG	LUM	ADCY1	SEMA3D	RP11-59E1	PHACTR3	CPNE6	C2orf40
C1QL1	CD109	ZFR2	RP11-751H	FOXP2	TTR	GALNT5	IGLV8-61	CRLF1
POU6F2	HBB	HOXB6	IGHV4-39	VAT1L	ADCY8	DEFB1	PABPC1L2A	SNAP91
CACNG6	TMEM63C	PPP2R2C	ICAM5	ENSG0000(	CDH12	MT1G	TPSAB1	WFIKKN2
DIO3	F7	LINC00475	TACSTD2	NEFM	PKHD1	VPREB3	UPK3BL	GAL3ST3
IGLC7	MFAP5	ST8SIA5	SULT4A1	APOC1	GULP1	AQP12A	PAMR1	ACTN2
CALN1	FCRL5	LRP2	ELSPBP1	PITX1	NPSR1-AS1	RP11-255B	RP11-260A	RXRG
IRX3	SPATC1L	ST18	ASIC1	HS6ST3	RP11-495P	ALDOB	GRP	CLEC3A
SP8	HLA-DRB5	CXCL11	ADCY5	CYP11A1	IGF2BP2	SPOCK3	GDNF-AS1	DUOXA2
KRT83	CFTR	AGAP2	LEF1	IGHV3-53	PNMA3	CDHR2	LMX1B	RAB3B
AC099552.	MUC1	OTX2	IGSF9	RAB3B	HHIP	EVX1	TGFBR3L	CLDN11
SCEL	PTGIS	IP6K3	AC108142.	SLC5A2	MT1F	CYP2B6	AC022182.	TCERG1L
COL10A1	TRPM6	RHOD	RP11-734K	AC067959.	HOTTIP	CLIC3	DLL3	PTGIS
ONECUT2	TMPRSS13	GPD1	MLPH	IGDCC4	UCHL1	PVRL4	TSPAN11	ZIC4
FUT3	CTD-2192J	BHLHE22	MT1L	TNNT1	GRID1	TRIM15	LRFN2	FREM1
A1BG	RPL37AP8	MGAT4C	IGKV3-15	CXCR2P1	CSAG2	FCRL2	ABCC3	CACNA1G
IRX2	GUCA2B	IGKV2-24	IGLV1-40	GYG2	DGCR5	ENSG0000(	FGF12	BHMT2
SST	ORM1	SLC5A11	GNGT1	ARSF	IGHV3-33	HPN	LGI1	FBLN2
HIST1H2AI	GABRB2	WNT7B	RIMS4	ADCY2	LAMC2	FSTL5	HCG4P5	KCNJ3
CAPN6	TFAP2A	SHANK2	TRAV30	HPCAL4	PLEKHB1	TMEM40	EMX1	EGFL6
RDH16	LRRN1	SLC25A48	KLK4	UNC93A	APOC3	RP11-706O	LINGO2	AC053503.
ITIH1	IGHV4-55	PLP1	RP11-1267	B3GNT3	CELF5	LINC00661	FREM2	IRS4
IGF2BP3	TNFSF9	CLVS2	STAC	CLNK	BCHE	IGKV1-8	RP4-555D2	CBLN2
OVOL1	TBX10	CYP4F3	IGHV1-18	IL1RL1	NECAB2	MYH7	LRFN5	ALDH1A2
KCNJ3	IL1B	GSDMA	ENSG0000(	IBSP	SLC29A4	PKP1	GPM6A	CDO1
OGN	IGFALS	CTD-2316B	ENSG0000(	LRRC2	TMEM82	CABP7	AP000783.	SYTL5
PIGR	ENSG0000(	ENSG0000(	FSTL4	ENSG0000(	KRT80	FCGR3B	MFAP5	AK5
DPEP1	TMEM236	ZNF385B	CACNA1I	SLC16A12	KIAA1244	PRSS8	LIF	FLRT3
SLCO1A2	C2CD4B	FUT9	RP11-438D	LINC00460	IFI27	SFRP2	DNAH11	LINGO2
CALB1	PTGDR	WDR16	PDZK1IP1	SMIM5	ADH1A	HS6ST3	B3GALT5	IGLV1-51
CADM3	ENSG0000(	LECT1	TCEAL2	TNNI1	CCDC64B	ENSG0000(	HTN3	FSTL5
DSG1	RP11-93B1	KCNH8	AR	GLP2R	RP11-213H	AADAC	MKRN3	DRD1
C6orf223	LTK	FGF5	TRIM50	RP11-424G	MT1A	LEMD1	RNA28S5	GSG1L
CLDN19	SFTA2	LRRTM4	DPP6	RP13-452N	KRT6A	MMP11	RORB	IGLV8-61
FCRL1	DARC	LBX1	CACNA1D	IGLV4-60	ENSG0000(	FER1L4	CPNE4	CTD-2314G
IGKV1-16	RP11-89K2	PCDHA3	CD5L	C6	NEBL	IGHV1-3	C8orf34	RP11-844P
SCGB1D2	RP11-161M	CYP4F29P	SV2B	RP11-697M	SSUH2	KCNMB2	FIBCD1	MEG9
FAM153B	FBLN2	PCDH11Y	PCDHB5	IRX2	SAMD5	G6PC	IGFN1	HOXC11
BARX2	ENSG0000(	KCND2	VIPR2	KCNK3	TNFRSF19	RP11-438N	CES5A	CA12
FST	ITGBL1	SUSD5	RP11-20D1	IGLV1-36	ASPDH	KCNK3	DTX1	RP11-867G
H19	PRSS1	IGHV3-74	RP11-116O	WNT7B	WNK4	EDN3	IGHV3-74	MTND4P12
CHGB	SULT1B1	TRIM29	MAPK4	C21orf62	RPSAP53	CCL17	DMKN	LAMA2
SERPINA6	MMP9	OPCML	HOXA11	BCAS1	GPR37	UGT1A1	IGKV2-24	UBE2QL1
ALDH8A1	ST6GALNA(	KCNF1	PAPPA2	RP11-115N	MRAP2	TMEM196	C8orf47	PRKAA2

KLK1	ENSG0000(	C19orf59	DARC	EMID2	SLC5A1	INA	LPHN3	OMD
TMSB4Y	AQP3	SLC8A3	RP11-958N	PKHD1	ITGB8	ANXA13	THSD7B	SIX3
C6orf141	UBD	C1QL4	MIOX	GLYATL1	CYP4A11	RP11-326C	AC002398.	RP11-400N
PAH	FER1L4	MAL	KRT80	TFAP2C	GLDN	LGALS7	ENSG0000(	NKAIN4
RP11-357H	GIF	CDH22	FOSB	TENM3	DMBT1	SERPINA3	IGHV3-53	ENSG0000(
PRSS1	KCNV1	TKTL1	AMTN	KSR2	MZB1	ADH1C	CA4	MKRN3
ADORA1	DPT	RASAL1	RARRES2	KLK7	CEACAM6	MTND4P2(	VIPR2	RP11-454K
ENSG0000(	GPD1	ENTPD2	ZFP57	RP11-798K	PIK3C2G	CLCA2	RP11-178A	DLX1
UNC80	IGHV3-66	FMOD	ENSG0000(	IGLV2-18	CYP4A22	IGHV4-28	NR4A3	IGHV3-53
SLC7A4	SCAND3	EVA1A	SLC22A3	GPC5	CCDC162P	TACSTD2	IGLV4-69	NKX3-2
BANK1	OLR1	SLC10A4	LINC00668	SLC26A9	ITIH5	COX6B2	IBSP	IGHV2-26
PCDHA10	FTLP3	TREM1	LGI3	MAL2	SLC5A12	SYT5	SYT16	RP11-54O7
CTD-2008P	HIST1H4C	LINC00403	SFN	HOXB13	VSIG1	CYP2S1	PDZK1	CYP19A1
PCDHB5	AIFM3	SLC7A14	FABP4	C1orf95	RP11-219C	SIGLEC14	PCDHA6	MXRA5
CA12	GGT6	PKP2	NAT8L	AOX1	LHFPL4	SLC6A17	ABCG8	CPB1
RP11-525K	PYY	CLGN	IGLL5	KIF25-AS1	AGXT	VTCN1	QRFPR	ANPEP
SPDEF	BST2	C11orf9	SYT4	ATP2B2	CLEC2L	PROM2	RNF43	OPCML
ENSG0000(	AHNAK2	IGLV7-46	RP11-152C	IGKV3D-20	PDIA2	MUC3A	TCEAL5	KRT19
CLRN3	VANGL2	KLK7	DDIT4L	OLFM4	POPDC3	SLC38A4	ADAMTS19	DIRAS2
PADI1	FOXA1	AHNAK2	AIF1L	IGKV2D-29	COL10A1	IGKV6D-21	COL28A1	CD177
IGSF1	HOXD10	CREG2	CTNNA2	STK32A	FAM99B	TAGLN3	IGDCC3	CYP24A1
IGHV3-66	FAUP1	CAMP	RP11-495P	FRMD7	ZPLD1	IGKV2D-40	ENSG0000(	RP11-256I9
WNT10A	CTD-2147F	FAM196A	LRP2	SORCS1	OXT	RP11-526J3	WISP2	JPH3
MCOLN3	NRXN3	OLFM3	IGHV1-46	TUBB4A	XAGE5	IGLV5-37	IGHV1-2	SLC7A4
ZPLD1	THNSL2	RGS22	CTD-2247C	LRRC15	ILDR1	CXCL14	SEMA3B	KRT5
NRAP	IGKV1-8	INSM1	IGLV3-1	ANXA3	ENSG0000(	C19orf33	ENTPD3	CHI3L2
NEU4	SSTR5	KISS1R	RP11-495P	APOC3	MYRIP	CYMP	FAM123A	RPSAP53
PF4V1	EDN3	HS3ST3A1	SLC16A11	PRSS22	KCNJ4	IGKV1-12	IL1RAPL2	ENSG0000(
CDHR2	ZDHHC8P1	OSR1	PCDHGA3	MMP1	AKR7A3	AMTN	FOLR1	NOG
IGHV3-72	IGLJ3	FAM153B	GALNT9	IQSEC3	RP11-114G	BAAT	CDH8	IRX6
C7	SYT1	PCDHB3	KLB	PTPRH	PRAMEF4	IGHV3-66	APOC1	ANKRD1
YBX2	RAB27B	MKX	MOXD1	SLC38A5	CHIT1	SPIB	EMILIN3	LRRC2
AKR1C4	SNORD17	PCDHGA12	ABCA8	COL10A1	RP11-757G	HRASLS2	AQP10	PKP1
SPTSSB	SNHG14	EGFR-AS1	CWH43	IGLV9-49	C6orf132	PLP1	KIRREL2	EPHA3
GJB6	TENM3	POU3F4	SLC1A2	PPP1R1B	CUX2	SPDEF	OPRK1	CHODL
CRYAB	RP11-269C	CHST9	CTB-1I21.1	EFNA5	PFN1P11	CCL24	DBH	FOXF1
SORCS1	PHGDH	SNAP25	AC012499.	MGAM	CIDEC	SFRP1	FAM131C	CSMD1
C5orf46	J01415.6	SRY	TUBBP5	BAAT	GUCA2B	ENSG0000(	RP11-299L	PHACTR3
HOXB8	IGHV2-5	OPRK1	MYO1H	ERVMER34	SNAP25	GABRA2	RP11-863P	CD70
RP11-438N	GPC3	IGKV1-6	CTD-2554C	ENSG0000(	SYT3	POU6F2	ARPP21	MEDAG
MZB1	SLC7A4	FRMPD4	C21orf33	FOXJ1	UPB1	CD177	SHH	OLFM4
WNT7B	RPS19P1	RP11-313J2	IHH	ARPP21	CYP19A1	AC066593.	FAM131B	CHD5
UROC1	HOXA13	ADAMDEC1	COL9A2	NOG	GS1-179L1	HLA-DQA2	ACAN	NGEF
IGHV1-3	ANXA13	C1QL3	GLOD5	RP11-556I1	PRR15	GATA4	CDH23	CACNA1E
RP11-19E1	SLC38A11	DLX1	C1QL1	OXGR1	ACSM2A	AC131097.	AC005062.	AC141928.
AP1M2	SFRP1	RP11-809C	DCDC2	TRPC2	FAM178B	ALDH1L1	CTB-52I2.8	ZFHX4-AS1
ATP8A2	PCDH19	GLYATL2	WISP2	GLRB	LRP1B	SLC28A2	PTPRR	NLGN1

CRIP3	HCG4P5	TOX3	ANKRD22	ACPP	PRSS12	DSC3	DKK1	EN2
ABCB4	TNC	CYP1B1	ENSG0000(	ISLR	IGKV1-27	VSTM2A	RP11-586D	RP11-197K
ISX	MLF1	CYP27B1	ENSG0000(	ENSG0000(	TTTY15	SCG5	SOX2	CDKN2A
RP11-93B1	GBP5	ZMAT4	ENSG0000(	CLGN	HMCN2	C22orf42	PRND	CPXM2
GCG	BTNL3	GPR1	TMEM35	CXCL1	C1orf116	MUC13	LCN1	TFAP2C
UGT2A3	PLN	PTPRN	CCDC68	TFAP2A	TMEM163	BHLHA15	LAMP5	CFD
APOC2	DACT2	COL25A1	BIRC7	CTSE	UGT2B11	GGT6	DMRT2	MAT1A
RP11-707A	PGM5	MMD2	PCSK1	PPP2R2C	ADCY1	PAX9	IGLV1-51	CLSTN2
GDNF	RP11-627G	ZDHHC22	SLC9A4	CIDEC	EFNA5	SEZ6	GABBR2	AC079776.
C4BPB	ENSG0000(	MEPE	BARX2	CYP4F11	LHX3	HOXA11-AS	STEAP4	RP4-663N1
LINC00261	SULT1C2	HPCAL4	UGT2B28	PTPRD	SLC15A1	GPA33	RP11-124O	RIC3
AGTR1	RP11-400N	C11orf87	NALCN	COL22A1	MTND4P2(	CHRDL2	RP11-531A	FERMT1
OR2I1P	GAD1	ASIC2	NEURL	SLC2A5	RBP1	KLHDC7B	MARCO	SHANK2
SPRR3	ABCA3	ENSG0000(	LYPD6B	ATRNL1	FCN2	CPHL1P	IGHV3-49	SMPX
TMC5	HCAR2	PDLIM4	RGS8	IGHV2-70	PNMA5	SPRR2A	MFI2	MGST1
IDO1	ENSG0000(	DLGAP1-AS	C12orf56	HS3ST2	ENSG0000(	TSPAN8	SYNDIG1L	FIBCD1
RAB3B	ALB	CARTPT	RP13-452N	GAL3ST3	PTPRD	ANKRD1	SLC44A5	RP11-354E
NPIPL2	ZNF43	RP11-649A	PYDC1	VWA5B1	FMO1	PENK	ANGPTL7	MIR143HG
FSTL4	SYT13	B3GAT2	SPSB4	TINAG	AOX1	TNNI2	MUM1L1	DUOXA1
FRMPD1	MRC1	HOXC8	FLG-AS1	IRX1	IGHV1-69	TMSB4Y	C11orf87	IL13RA2
RP11-1267	CHGB	RARRES2	EPHA6	KNDC1	SLC39A4	NR1H4	MMRN1	PKP2
SPRR1A	SPRR1B	COL5A1	TFPI2	ANGPTL1	G6PC	AC078941.	NRGN	PPL
AMN	CTTNBP2	CLDN11	CTB-36O1.7	NOL4	DCAF8L2	RXRG	RP11-742N	GPR1
CHEK2P2	PRKCG	PTX3	RP11-146N	LONRF2	MARCO	CDH10	AC093375.	HOXA11
KCNJ6	FOXP2	ADAMTSL1	INSC	HSPB7	HULC	LAMB3	AMHR2	PTGES3L
GNG4	REN	KCTD4	CLDN8	PLA2G2A	DUSP9	CYP4F24P	SFRP1	RP4-792G4
DMGDH	CHRM2	CAMK1G	SLC22A10	SOX11	TRPV6	NPY	IGF2BP2	PCDH11X
NDNF	CAPN13	MIR7-3HG	IGHV3-15	ROR2	KCNC2	PRKCG	CXCL2	ENAM
FMO3	C1orf95	RASEF	GGT6	FLRT3	FAM201A	RP11-259G	AQP5	CACNA1I
IGLV7-46	AC144521.	NDN	C1orf64	RP11-411K	DIRAS2	PTX3	IGF2	C12orf54
COMP	RGL3	SORCS1	WTAPP1	LRRC19	APCS	RTL1	RP11-1018	SUSD5
OIT3	COL8A1	HOPX	SEC14L6	FOXA1	YBX2	KLK12	SCN7A	CKMT2
HMGA2	AZGP1	CNTNAP5	MFSD6L	ANKS4B	UCA1	FUT3	RALYL	KLHL30
S100A14	MLPH	CLVS1	THSD7B	RP11-127I2	SPOCK1	GCNT3	SLC35F3	RIMS1
CTD-2195N	RTN4RL1	CCL2	ART5	TNFRSF9	IGFBP1	RELN	KRT19	APOD
C2orf54	EDA	OSTN	AP000472.	PI16	ITGBL1	TRIM50	MT1A	XIRP2
RELN	PTPRU	C20orf166-	HCG4P5	EPHA10	ENSG0000(	ENSG0000(	DRP2	STK33
IGLV1-36	CD163	CTD-2554C	NUPR1L	MCOLN3	CDS1	EPHA7	TRIM55	DUSP9
CAPN13	CECR2	EPHA7	CRYM	PCOLCE2	ZNF385D	FOSB	CAMKV	PLCXD3
RP11-678G	DMRT2	CXCL10	RTN4RL2	ZNF385B	ENSG0000(	SCG2	LINC00645	LINC00517
FCN2	MMP13	RET	DEFB132	RP11-549B	RP13-143G	UPK3BL	KNCN	DLX5
GABRG3	RP11-413E	TFAP2C	TMEM45B	KRT13	TESC	SLC4A10	DPT	ST6GAL2
NPTX1	MSR1	EPHB1	PRSS29P	DNAJB13	EPHA10	IGHV3-7	LRRC10B	C1QTNF7
OR12D1P	CLDN8	PNMT	TMEM174	C1orf168	AHNAK2	TFPI2	PKHD1	EXTL1
PRR15	EYA1	IGSF1	RP11-469H	RP11-60A8	HAND2	AMH	RP4-792G4	C14orf180
RP3-324N1	GPNMB	ESPNL	AC073218.	DIRAS1	EVC	PDZK1IP1	PDGFRA	LINC00473
TRPV6	CPA3	RP11-615I2	MYRIP	CTD-2195N	SLC26A3	ENSG0000(	SLCO5A1	CALCA

PLCXD3	RASAL1	HILS1	RP11-991C	LRRN4CL	ADORA2BP	CSF3	VDR	F2RL2
SLC15A1	MASP1	F5	CHRDL2	RP11-325F	IGHV3-73	GABRG2	TSLP	LY6K
CYP4F2	PRODH	IGKV1-17	SNTG1	TRIM15	MDGA2	FAM135B	NTSR1	MYBPC2
TBC1D27	LY6D	RP11-357K	IL6	IGHV3-73	UBXN10	RUNDC3A	POU6F2	NCAM1
TMEM27	SIK1	ESM1	HCAR3	IGF2BP1	RP11-308B	AC079466.	RNF212	ZMAT4
MYO7B	CXCL17	LCNL1	CELF5	CRABP1	HOXC10	TH	AGTR1	TNNT2
IRX6	TRNP1	IGLV3-10	C1orf233	GALNT9	PPP1R3G	CTD-2547H	GALNT8	GPR64
SLC22A3	FCGR3A	C11orf88	GRIK1	CXorf22	THBS2	D86994.2	HS3ST5	NELL2
PCDHA12	CBFA2T3	PCLO	LINC00113	IGLV3-27	ACSM5	CDHR5	RP11-552E	NKX2-2
TM4SF5	GLT25D2	RP11-298D	VAX2	USH1C	DPEP1	IGF2BP1	RP4-610C1	FDCSP
CYP2C9	IGHV2-70	TF	ERBB4	CKMT1B	BCAS1	GJB2	CGNL1	CACNA1B
CLGN	HLA-DRB6	AC116614.	IL8	GNGT1	C19orf80	UGT1A6	LRRC15	ENSG0000(
LYPD6B	MYOM3	SNX31	VWA5B1	PRELP	PLA2G2D	KCNJ3	STMN2	SCAND3
HTR3A	CCDC60	KCNH5	SLC19A3	SCIN	APCDD1	C1orf106	DSP	AC145291.
IGLV4-60	IGKV2-29	LILRB5	DRAXIN	RP11-145M	CCNO	BARX1	POU3F2	HRK
IGLV7-43	CPE	CD300E	OLIG1	CRB2	IGSF1	C19orf59	USP32P1	PRRG3
INHBC	MIR143HG	FLG	PADI2	CFTR	GGT6	FOLR1	TMEM171	PTGER3
ELOVL2	SPRR1A	RP3-460G2	CD22	GABRB3	PLCH2	LRRC19	TSPEAR	CYP4F12
AGT	ENSG0000(	FOXJ1	CTNND2	PLA2G2D	ACSL4	ENSG0000(	HMX3	POU3F3
A1CF	GAS6-AS1	SH3GL3	PTCHD2	ARG2	LYZ	IGHE	COL11A1	PPARGC1A
C8G	CCL19	GRM5	TRPM3	LAMC2	HOXD-AS1	UPK2	RP11-89K2	MYH8
ASGR1	TRIM29	VAX1	IGF2BP3	RP11-386B	ENSG0000(	IGFL3	WSCD2	ELAVL3
GLT25D2	CHI3L1	SPOCD1	GPC4	PCBP3	ZCCHC16	BIRC7	PRSS50	ADCYAP1
ANKRD2	MMP11	COL14A1	SLC34A2	CALCR	RXRG	XPNPEP2	IGLV3-10	CLIC6
ANGPTL1	PI15	CCDC108	RP11-46C2	SFTPB	IGHV4-34	RNA45S5	SLITRK4	NDST3
CHRDL1	CSF3	HS6ST3	GPR98	CDCA2	LINC00659	CALB2	SPTSSB	DPP10
CLDN10-AS	INSM1	MXRA5	SIAH3	CTC-575D1	RP11-618I1	VNN1	PCDHGC4	LIPC
IGHV3-64	RPL13P12	GABRE	ABCG8	WNK4	KIAA1239	CACNA1B	RGS22	RP11-766F
TNFRSF13B	SULF1	ANGPTL4	RP11-413E	GABRD	LAD1	CTD-2008P	ENSG0000(	CTD-2335A
DHRS2	ENSG0000(	TMEM246	SPOCK1	SLC4A3	SYT7	LYZ	GJD2	XK
INHA	CCDC80	NEUROD6	IGHV3-33	KRT6A	RP11-467L	SERTM1	SLC5A8	SYT10
SSTR5	AXDND1	KRBOX1	SCUBE2	CTD-2377D	LHX9	ENSG0000(	RP11-2E17	IGSF9B
MEG3	PALM3	SLC6A11	TMEM215	RP11-119D	PAGE2B	RP11-392O	ENSG0000(	TSPAN2
LRRIQ1	SLC3A1	AC002454.	HNF1A-AS1	AC021218.	RP11-70C1	ALDH3B2	MAL	IGKV2-24
NAT8	PTPN13	MYBPC1	KALP	TNNT2	U91319.1	HCG4P5	GIPR	LAMP5
RP11-48O2	C2CD4A	SEC14L5	MINOS1P3	EYA1	DDX53	AGMO	SKOR1	RP11-14O2
DIO3OS	STC2	IL20RA	GPR87	HSD11B2	AL133445.1	AXDND1	DLK1	FOXD3
HSD17B2	GREM2	S100A9	AC006159.	UGT1A6	UGT2B7	FCN1	LYPD6	DPYSL4
C11orf53	DNASE1L3	IL1R2	ROR2	RP1-27K12	GNAO1	KCNQ2	KCNG4	TEX15
TFAP2C	TINAG	FAM19A1	GPR37	PGF	MYEOV	IGKV2-30	CXCL13	KRT16
AC066593.	ARHGAP4	PPP1R17	RAB27B	UBD	CABYR	ACE2	SAMD5	ABCA6
RP11-424G	CPNE7	VIT	SYT17	SIGLEC8	DIO1	RP11-706O	CYP27C1	CDH22
HOXC13	PPP1R14C	TMPRSS3	AOX1	HLA-G	FBN3	C12orf39	GALNT14	KCNJ4
HTR1D	HSPB7	PLCXD3	CDA	D86994.2	RP11-549B	RALYL	REG3G	CXCL6
ABCG5	C10orf82	BPIFB2	RP11-404E	RP11-81H1	GREM1	CYP3A4	LPPR3	NOS1
PLA2G4F	NR0B2	NAT16	POSTN	SCN2A	PRAP1	CLRN3	MINOS1P3	KLHDC8A
CPNE4	IL6	ENSG0000(	GRID2	TMEM252	AC021218.	IGF1	FAM19A5	C10orf71

PNMAL1	XKR9	IGHV1-2	RP11-1042	AC073115.	LYPD6B	CTNND2	OLR1	IRF6
ST6GALNA(	COL17A1	ENSG0000(	APCDD1L	TRPM3	FEZF1-AS1	VWA5B1	NOX5	FMO2
EVX1	RPS11P5	RP3-340N1	NTM	FAXC	CDH19	PART1	RP11-10B2	PIANP
SELE	ADRA2A	PCSK6	MAFA	THRSP	PPP1R1B	C21orf62	PTN	LRRN4CL
ZIC4	HLA-DQB1	RSPO2	ADAMTS9-,	VGLL1	DPYS	AZGP1	NTRK3	RP11-438N
TFCP2L1	HOXB13	RXRG	RP11-855O	FUT9	TRIM31	C7	RP4-610C1	TOX3
ADCY8	LRRC19	CLIC6	SLPI	RP11-219E	HLA-DQA2	CRABP2	ENSG0000(	ADAMTS9-,
TCL6	GZMB	FOXA1	CTB-43E15	GZMK	PRSS22	IGKV5-2	RP11-8L2.1	FOXE1
PRSS29P	TCP11	NCAN	AC009166.	C19orf80	RP11-89K2	VIL1	C21orf33	CTD-3049M
ARSI	SERPINB2	CDH15	GATA3	TMEM30B	CLVS2	MT1H	GABRA3	IGHV3-73
FERMT1	CALCA	CNGA3	GSDMC	TMPRSS3	EMX1	CST2	INSC	HDC
HOXC6	C11orf9	CACNG5	AC019100.	KLRG2	SFRP2	STXBP5L	PHACTR3	TIMD4
AFF2	LGALS2	NHLH2	GPR39	WFDC2	CTSL2	BMP5	CAMK2A	C11orf87
AZGP1P1	SYTL5	PRSS35	RP11-750B	NTRK2	NLGN4X	ST18	KCNH7	C1orf110
PDX1	RP11-4K3_	PCDHB2	MCIN	TMEM27	HSD17B6	GNMT	SYT2	KCNG1
IL8	SCARA5	AK5	RP11-44F1	AIF1L	ENPP7	TPSD1	HS3ST4	NTSR1
IGLV5-45	IGHV3-64	FST	LOXHD1	SLC34A3	FA2H	RP11-420K	CLUL1	MS4A2
DPT	ATP12A	AKAP14	AC010524.	PI15	ANKRD1	DLGAP1-AS	KCNJ4	PRUNE2
GREM2	IGKV1D-8	CACNG2	FBXO40	RP5-1172A	TMEM45B	PRSS29P	LRTM2	PCBP3
IGKV1-8	ART3	C4BPA	PTH1R	FDCSP	RP11-1036	CLDN3	UNC5D	GSTA1
RP11-706O	TPSB2	RP11-286B	RP11-44F1	IGHV3-13	KCNH2	NTF4	CPNE7	ASPN
VSX1	ZNF334	NDNF	ENSG0000(	KLK4	KCNF1	SELE	CREG2	ASPA
IL20RA	FDCSP	PDX1	CA4	RP11-124N	FAM155B	MINOS1P3	SOSTDC1	FMO1
PCSK1	RP11-697M	RP1-177G6	BHMT2	CCDC146	PAK3	TNFRSF13C	MYOC	RP11-863P
IGHV4-61	DDX43	GABBR2	NCAM2	MOXD1	HRASLS2	FCRL3	GPR83	RPSAP52
TMEM130	OCA2	TMEM151A	SYT13	SLCO1A2	SPATA21	PM20D1	ESPNL	NTN1
MYO3B	PHACTR3	GABRG3	CTA-929C8	DPT	SNTG1	TNFRSF13B	LINC00599	REEP1
RAB3C	FAM3D	BCAN	GLIS3	LINC00675	PDPN	AC016708.	HOXC-AS2	CACNG7
ZNF536	FADS2	MMP19	LRRC2	SLC7A9	SLC1A1	HBA2	AZGP1	HS3ST2
C19orf21	SCARNA13	COL1A2	OVOL2	WFDC5	LONRF2	HSD17B2	RP11-84E2	LRRC4B
IGFBPL1	RARRES1	AC093726.	RP11-299H	SYT8	SH3GL3	RP11-776H	HOXB-AS3	IDO1
ENSG0000(	ENSG0000(	MLIP	SERPINF2	ADAMTS9-,	RP11-115J1	DUSP26	TSTD1	WNT11
CTD-2147F	CREB3L1	FCGR2B	HTR3A	ACTC1	AP000688.	CHRM2	ENSG0000(	LRRN3
RP11-776H	RASD1	SLC26A4-A	GRHL2	AQP4	SHBG	WNT2	DCN	PAK3
ACTBL2	PLA2G2D	TPSAB1	CPLX1	EPHA6	NKAIN2	NRXN1	CCDC33	MSLN
MUC1	AREG	RP5-1121A	FAM181B	RP11-219G	SMPX	ABP1	NPIPL2	HEPH
CNR1	TMEM132(	LGR5	RP11-380D	SYT7	SHD	BRS3	ZIC3	ATP2B2
SCN9A	EMX1	IGHV4-34	RP11-564C	DMKN	ENSG0000(	RP3-417L2(	RP11-958N	MMP7
U91319.1	KIF19	RP11-844P	CCDC64B	CGREF1	VCAN	LGR6	AC016708.	CXCR2P1
ITIH5	GPX1P1	KRT16	HOXA7	NRAP	HKDC1	NSG2	PCDHA11	DUSP26
BMPR1B	NPR3	ENSG0000(	ENSG0000(	SFN	RP11-742N	MAT1A	RASGEF1C	CDH8
NR0B2	ZNF813	ALDH1L1-A	VN1R85P	GABRP	GYG2P1	CDK5R2	AC009498.	PTGFR
TMEM171	PF4	FAM92B	CLDN3	IGLV5-45	SLC22A10	SLC17A6	RAB37	KIF26B
ENAM	CCL20	ARHGDIG	GSTA1	MAB21L3	TEX11	CYP2W1	RP11-286N	MB
RIC3	DIO3	LHX5	SMTNL2	RNU4-2	CEACAM7	UBD	KCNK1	KCND2
DLX6-AS1	DPP4	BEND4	CLGN	CKMT1A	GLYATL1	MS4A8B	THEM5	CNKSR2
IGHV4-28	RP5-857K2	ENSG0000(	ENSG0000(	RP11-396O	LINC00648	BPIFB2	ADRA2A	SPESP1

CYP2A7	GPR110	CNTNAP2	CAPN13	CLEC18B	SORCS2	RETN	SEMA3C	CA4
RP11-223I1	RPL39L	AL009178.:	BMP3	CYP8B1	HUNK	OGDHL	RP11-713C	CTNNA2
Metazoa_S	EPHB1	IGF2BP2	ENSG0000(	PSG4	GPLD1	TNNI3	SLC35D3	PPP1R14C
RP11-372E	RNF182	SPATA18	COL4A6	SCG2	FTCD	IL20RB	BMP7	PAPPA2
IGKV2D-29	GDF10	COL9A3	HLA-DQA2	GMNC	MSC	CALN1	HOXA10	NDNF
C5	F10	WFIKKN2	SLC8A1	ABCC2	TRPM8	CYP26A1	STK19P	RIPK4
LIN28A	PLA2G2F	COL10A1	ENSG0000(	FAM153B	ARNT2	ZIC5	RP11-706O	CCKBR
SFTA2	ROBO2	ERBB3	C1DP1	ELMOD1	PI15	PAX7	USH1C	CXCL5
CDX2	KCNMA1	SPAG17	AC009299.	RP11-674N	AXIN2	RP11-356M	IL1A	C4B
LYPD6	SIX2	TGM5	RP11-109J4	CRABP2	BTNL3	CLDN6	RBP4	COL19A1
RP11-462L8	HHLA2	HOXA11-AS	ALDH1A1	STMN2	RSPO2	RP11-59J5.	PLEK2	YBX2
PDZD3	SLC26A9	ARSF	ACTC1	CRHBP	ABCG8	SLC38A5	TRHDE-AS1	DLGAP1
CTC-327F1(	MT1M	PRKCG	FOXF1-AS1	TRIM29	RHCG	SLC6A20	TMEM132E	KIAA1549L
HSD11B1	SCARNA6	HS3ST4	AC118345.	MFAP5	LINC00470	USP32P1	TFCP2L1	PLAC9
RP11-1220	NINL	HOXB-AS1	PCDHGB4	KCNH6	HABP2	RTBDN	IL13RA2	RXFP1
SEMA3D	TRPA1	HCG22	HOXA10	IGHV4-61	FABP4	SYT16	ENSG0000(	GABRA2
IGSF11	IL1RN	ARSI	FGF12	DBC1	ENSG0000(	TUBBP5	KCTD8	SEMA3A
TMEM40	C4orf48	LRRC61	SEMA3C	ENSG0000(	CES1P1	NRG3	RP11-403I1	PTPN20A
GRP	AP001065.	RP11-731J8	AQP4	DHDH	SSTR1	DMBX1	POU3F1	KRT34
PSAPL1	LINC00654	PCDHA2	AC017048.	NPY6R	ENSG0000(	ZG16B	RP11-120I2	BAI1
FGF9	SELENBP1	CTHRC1	RP13-439H	TREH	UGT1A6	LCT	TOX	GALNT9
TENM3	MT3	TPBG	RP11-745L1	IGHV3-66	KRT14	GPC5-AS1	FOXI2	PYGM
CNR2	PCDHA4	ATP2B3	IGHV1-2	GPR143	LYPD1	CDH19	NTNG2	VANGL2
TRPA1	FPR1	HECW1	BEND6	UPB1	CHRDL1	SAA2	PRKCG	ARSE
TNR	RNA28S5	CCKBR	FIBIN	RP11-1L12.	ARL14	ANPEP	MOCOS	NRG1
PROZ	ENSG0000(	CD5L	SH3RF2	SVOPL	LDHC	AC016745.	RTL1	DLX3
ARMC3	RP11-568J2	KLRK1	ZNF728	MYL3	ALOX15B	OLR1	B3GALT1	EBF2
GALNT5	TFPI2	RASL11B	EGFL6	LRRN2	LINC00511	C5orf38	MEOX2	MAGEC2
CXCL9	CES3	WNT2	HOXC9	PRODH	FBXL16	AMN	HTR4	RASL12
ENSG0000(	FAM83A	LRRIQ1	NKAIN4	LINC00494	PLGLA	SLC44A5	C2CD4B	ESYT3
CXCL1	HIST1H1D	LINC00320	F7	ALDH1L1	VEPH1	C1QL1	NEUROD2	PIK3C2G
TNFRSF11B	AC012501.	CCDC33	CTD-2369P	FA2H	DYDC2	ENAM	AFF2	L1CAM
RP11-30K9	ST3GAL4	GOLT1A	USP32P1	FCRL5	AC010524.	IL6	ADRA1D	NDP
ECHDC3	BCAS1	TMSB4Y	MAPT	USP32P1	RP11-300J1	UGT2B11	TPD52L1	FSTL4
KCNH3	ENSG0000(	RP11-1191.	RP11-846F4	AC010084.	FHOD3	POU2AF1	NECAB2	RP11-120J1
BMP7	MAOB	TFCP2L1	ENSG0000(	ENSG0000(	BMP7	AC016683.	IL1RL1	GALNT8
ABCC8	ANGPTL1	KCNS1	TGFA	IGKV6-21	PRKAA2	AHNAK2	WDR72	BAI3
C4BPA	ADRA2C	FAM225B	HOXA6	COL1A1	RP11-150O	ENSG0000(	KLK2	PCSK9
ABCB11	RPL13P2	RP11-107I1	ADAMDEC1	BSPRY	FAM110C	RHOV	CTC-378H2	SLITRK4
KCNH2	CASQ2	IGLV7-43	DNASE1L3	TMEM45B	UBE2QL1	NR0B2	DLGAP1	GABRB2
RNASE7	SRPX2	KIF25-AS1	SERPINE1	RP11-319F.	CRISP3	MFAP5	FSIP2	RP11-16M8
ADARB2	MLK7-AS1	HHIP	MGAM	TPSB2	HOXB13	IRX1	CA10	CHGB
DIO2	IHH	KCNK1	SLCO4C1	NMRK2	ANXA10	SHH	PPP1R1C	RASL11B
SLC9A2	FLJ22184	AC007682.	ADAMTS3	C10orf126	FUT6	ZFY-AS1	CST2	AC093850.
LMX1B	FAP	RP11-439H	PRLR	RP11-496I9	SLC16A12	ENSG0000(	SOCS3	CLEC3B
FAM81B	QPCT	APCDD1L-A	AC004988.	ALDH1A2	MNX1	OR2I1P	FGF11	GPR37
ENSG0000(	SNORA23	HTR5A	DSCAML1	LA16c-329I	ANKRD35	MTTP	TMEM163	RP11-399O

F12	RNU5E-1	ENSG0000(	RP11-166D	IGKV1-8	RP11-262H	ENSG0000(	ENSG0000(	P2RX1
FLRT2	RPL3P7	PCDHGB3	SPARCL1	ALOX15B	STEAP2	MIR31HG	RP11-745L:	EMX2
CBLN2	ENSG0000(	KRT17	ACAN	RP11-2E17	FBXW10	RP11-44F2	C3	PLAC1
COX6CP1	COL1A1	GRM7	KCNK15	RP11-314M	RP11-396O	CCKBR	ESM1	ITIH3
TNFRSF13C	TREM1	DTHD1	TENM1	TSPAN1	ENHO	C4BPB	TNR	SLC27A6
SIX2	ENSG0000(	AC140481.	IGHV4-59	C5orf38	CYP2D6	PCP4	EPCAM	LBX1
FCAMR	NEU4	GRIA4	CHRNA3	CXCL14	DYNC1I1	P2RX5	RP11-142I2	ARHGEF4
GABRA5	SERPIND1	SP9	BAALC	CHRNA4	EMID2	CXCL9	GYG2P1	CBLN4
OXTR	IL11	IGLON5	RP11-389K	IGHV3-72	WNT7B	MMP3	RNA5SP11:	NSG1
CNTN3	OSM	PRAME	RP11-59N2	TRPV6	NTRK2	ADH4	FGFBP2	RP11-114G
CKMT1B	KRT7	DYDC2	RP11-380I1	THPO	WDR72	SLC14A1	FMO2	PSG4
GDF2	RP11-23P1	AC092484.	STMN2	CHI3L1	FOLR1	KCNN4	ABTB2	MYOD1
OGDHL	HLA-DQB2	AP000696.	ENSG0000(	ARHGAP40	PNMAL1	SCN7A	MZB1	NNAT
CTD-3080P	IGHV3-20	GATA6	ENSG0000(	CES1	SCARA5	HAPLN1	SLC2A12	AOX1
CRTAC1	CCL13	RP11-259G	RP11-167H	RNF212	KLK4	ENSG0000(	F12	CNTNAP3

10 cancer types

SKCM
TYRP1
KRT6A
RPS4Y1
KRT16
KRT14
KRT5
KRT6C
MAGEC2
KRT6B
DDX3Y
KRT1
MAGEA4
XIST
MAGEC1
KDM5D
PAEP
CTAG2
EDN3
SPRR1B
S100A7
PAGE5
NELL1
MAGEA6
TXLNG2P
OCA2
MAGEA12
EIF1AY
MAGEA3
MAGEA10
CALML5
KRT17
HHATL
PMEL
CALML3
MAGEA1
UTY
USP9Y
IGLV3-19
RP11-80F22.9
IGKV4-1
MAGEB2
ENSG00000270816
CASP14
CSAG1
CHST9

IGHV1-18
TRPM1
SFN
GJB6
IVL
ALDH3B2
ZFY
KRTDAP
SPRR2E
IGHM
IGLV2-14
IGHG1
TRIM29
RP11-429E11.2
DSG1
VGF
IGLV3-25
IGKC
ABCB5
IGLV1-40
DSC3
DCT
IGHV3-21
MAPK4
IGHV3-23
DSG3
IGLV3-21
IGKV3-11
LY6D
IGLV3-10
EYA1
SPRR1A
RP11-527H14.2
IGHV1-2
IGHG4
IGKV3-20
IGLV2-23
PRKG2
DSCR8
IGHV5-51
SPRR2A
IGLL5
PSPHP1
IGKV1-5
MAGEA11
SERPINB5
IGLV1-47

PI3
SOSTDC1
PRKY
IGHV4-39
CR2
PMP2
FABP7
PKP1
IGLV3-1
IGLV6-57
TTTY15
IGLV2-11
ENSG00000280411
SBSN
CCL21
SFRP1
IGHG3
IGHV3-15
KRT13
ENSG00000270550
IGLC3
IGLV1-44
LRRTM4
COL9A1
IGLV4-69
IGKV1-9
KLK6
MTRNR2L1
IL13RA2
MUC15
IGHV4-59
GATA4
GABRA3
LHFPL3-AS1
KLK5
IGKV3-15
BAAT
KLK7
FDCSP
IGHV3-73
IGLC2
IGHV1-24
LINC00439
IGHG2
TFAP2B
RP11-98L5.2
IGKV1-17

SPRR2G
IGHV3-11
PRDM7
HOXB13
SPRR2D
LRP2
IGHV3-33
OLIG2
IGHGP
ASB11
RP11-369C8.1
CMTM5
CDH1
IGLV8-61
TF
IGHV1-69
IGKV1-16
SERPINB3
MMP13
CSMD1
ITIH6
IGHV1-46
IGKV1-27
KRTAP19-1
HAPLN1
AC145110.1
CNDP1
IGHV3-74
IGHV3-49
ENSG00000278196
RP4-718J7.4
AC073236.3
SSX1
TYR
BMP7
PCSK2
SOX1
IGHA1
SALL1
FAM178B
ANGPTL7
FSTL5
S100A7A
RP11-399D6.2
ZNF536
IGHV3-48
SCRG1

POU3F3
GRIK3
MLANA
LINC00520
IGKV1-6
PLA2G2D
LGALS7B
ADCY2
NRG3
IGHD
AC018730.1
IGKV2-24
NKX2-5
GFRA3
GPR143
COL22A1
COMP
UGT2B7
CTD-3049M7.1
ENSG00000278910
COL20A1
IGHV1-58
COL11A1
IGJ
S100A14
NMRK2
MRGPRX3
COL17A1
IGHV4-31
SOX2
SLC7A4
KIF1A
ENSG00000274576
KRT4
MS4A1
RP11-738O11.13
AC009499.1
LCN2
MUC7
LOXL4
CTD-2527I21.11
OLIG1
MAG
MEGF10
IGHV2-26
FGFBP1
CA8

PRIMA1
CTCFL
DPP10
SLC38A8
RP11-151D14.1
IGKV2D-29
LGI3
IGHV3-53
BCAN
LINC00504
NPTX2
FAM69C
CST1
DSP
MLIP
FAM133A
RP11-669N7.2
CST2
KRT15
CPSF1P1
KLK10
ASB4
INSC
ENSG00000277586
MMP8
HORMAD1
C7
LOR
RP11-63E9.1
GJB2
IGLV7-43
CRTAC1
IGLV3-9
SLPI
CXCL14
SYTL5
HOXC12
IGLV1-51
AC002511.1
ANO4
MZB1
FLG2
FMN2
RP11-30J20.1
PRSS21
CHIT1
TCN1

RP11-317N12.1
IGKV2D-40
SEMA3E
CCL19
KLK11
SPRR3
SPESP1
BAGE2
RXRG
GAPDHS
IGLC7
IGLV9-49
SLITRK6
LIN28B
NLGN4Y
DPP6
SERPINB4
IGLV5-45
LIN28A
EVPL
MPZ
KRT75
ADH1B
UCN2
HOXD13
IGKV1-8
RDH8
IGLV3-27
COL2A1
MKRN9P
CPN1
RP11-59E19.4
IGHV4-34
FREM2
DNAH9
CSAG2
HTN1
DSG2
LY6K
PAGE2
ROBO2
RP4-765C7.2
SERPINA5
ENTHD1
DNER
GRIK2
KPRP

PKLR
IGHV2-5
NCAM1
LINC00668
SLC5A4
MAT1A
S100A8
NAA11
TRIM63
GDNF
SERPINB13
LCE3D
PRSS33
KLK8
TDRD9
SLC45A2
PI16
PSG4
ABCA8
FCRL5
MAGEB1
RP11-197K6.1
IGHA2
RP4-529N6.1
EYA4
IGF2BP1
ZFP57
TFPI2
MIR205HG
IGLV4-60
RP11-1220K2.2
CLDN1
PKP3
SFRP2
PPP1R3C
IGHV4-61
CHRM1
LINC00488
MMP1
RP11-366L20.2
GABRG3
DLK1
MMP3
IGLV1-36
ARPP21
FAM83C
PDZK1IP1

IGKV6-21
AC095067.1
CLCA2
FXYD3
FAM83A
GS1-179L18.1
KRT78
IP6K3
MYH14
RP11-886D15.1
DMKN
LINC00470
FREM1
IL36RN
A2ML1
C10orf99
COL19A1
ZIC1
RAB25
IGLV7-46
RHCG
ERVMER61-1
LRRTM1
KRT2
BAALC
IGHV2-70
CNTFR
DSC1
MCHR1
GRIA2
ITIH5
IGHV3-13
MTND4P12
SYNPR
ENSG00000276775
IGHV3-66
AC012512.1
OLFM3
TMEM132D
CHRDL1
IGHV3-72
HPGD
IGKV3D-20
AP000688.8
LGALS7
PLA2G2A
ZNF280A

SPINK5
MME
HMGA2
FLRT3
CD79A
PAX5
CXCL13
RP11-449L23.2
C1QTNF3
TMEM163
RP11-2E17.1
PNLIPRP3
RP11-103J17.2
SHISA2
AL035610.1
ZNF560
KIT
RLBP1
GABRB3
RPSAP53
C20orf26
LRRC15
SERPINB2
PLA2G4E
NCCRP1
PLA2G4F
LCE2A
AGT
LINC00355
GABRG2
MAL2
CSAG3
TRIM48
SLC6A17
TCL1A
AKR1B10
SFRP5
IGHV4-28
IGFL1
PAPL
NKX2-2
CDH7
KLK13
RP11-114M1.2
ITGA10
SEC14L4
TEX15

AC074389.9
TUBA3C
FAM19A5
IGHV3-43
AGMO
RTN4RL1
ATP6V0D2
AC020907.1
IGKV5-2
EEF1A2
BEX1
LY6G6C
IGKV2-30
SCIN
SERPINB7
CRABP1
MAL
ST8SIA2
HES2
NEFM
IGHV1-3
EPHA5
PASD1
SCEL
KIAA0125
RNF128
NR0B1
SULT4A1
PI15
COL11A2
WFDC1
HS6ST2
GABRA2
GLB1L2
WFDC5
OR7A5
CA14
NTNG1
NSG1
ENSG00000279516
KCNJ13
HCN1
CDKN2A
CRCT1
VAX1
ENSG00000274956
LINC00589

POU2AF1
BIRC7
LINC00648
HLA-DQA2
RP3-527G5.1
ENSG00000275830
PCDHGA12
IGLV2-18
FCER2
PITX2
LINC00681
CD19
MGST1
AGXT2L1
AC092165.4
CTD-2380F24.1
CLDN14
RP11-46C20.1
PLA1A
FAM135B
RP11-522D2.1
PKHD1
MMP16
RELN
COL4A5
FAM163A
CILP
MMP12
IRX2
ENSG00000271579
PPP1R14C
SH3GL2
RP11-230G5.2
HOXB9
CHL1
GABRA5
MKRN3
ATP6V0A4
C2orf54
RP11-152P17.2
GAP43
SULT1C2
CCNYL2
ADIPOQ
L1CAM
FAM70A
CXCL9

NTS
WDR72
RP11-488I20.9
RP11-488I20.8
CEACAM6
CWH43
CYTL1
PPP2R2C
GSTM1
SLC16A6
DNASE2B
C5orf38
ILDR2
CD5L
PRSS3
CDH2
ENSG00000278530
RIMS2
GDNF-AS1
DKK1
GSG1L
SLC24A5
CXCL5
AZGP1
FCRL1
CDH3
ANO3
IGLV10-54
TMPRSS13
KCNS1
SLC30A8
RP11-3L21.2
SLAMF9
LCE1C
MUCL1
COBL
GJB3
TTTY14
ENSG00000275620
SYT6
XX-CR54.1
IGKV1D-16
ENSG00000272398
MYOC
PHKA1
CERS3
IGKV3D-15

FIBCD1
TCERG1L
GTSF1
SERPINA3
DAPL1
MAOB
DNAH2
FERMT1
IGHV3-20
RP11-129K20.2
IGHV6-1
ANXA8
KRT80
SFRP4
LMX1B
RSPO4
RP11-51M18.1
MAEL
VEPH1
SEMA3A
CA6
RNF182
RP11-706O15.3
VIT
TGM3
PCSK1N
TRPM8
CADM3
PRSS8
MCOLN3
SLURP1
ANKRD30B
KCNJ10
AC013402.2
TACSTD2
PAGE1
MGP
RP11-244B22.2
PLCH1-AS1
RP11-1038A11.3
TDRD12
KRT23
TNS4
PTN
PLCB4
OR7C1
OGDHL

BMPR1B
ENSG00000273706
IGHV3-64
PPARGC1A
DUOXA1
TFF3
SCUBE2
TNFRSF17
VAT1L
ADRA2C
SPRR2B
IRX6
CXCR2P1
ANO5
MT1A
ST8SIA5
ENSG00000278266
ENSG00000270090
PTGDS
RP11-401O9.3
ENSG00000273388
MAP7D2
AC104655.3
CRNN
HTR2C
RP11-404F10.2
LINC00189
RP11-308B16.1
RP11-107I14.1
PRRT4
BNC1
RP11-94H18.1
POF1B
SDR16C5
IL1RAPL1
WNT7B
ERVFRD-1
IRX1
RP11-400N13.3
ELOVL2
MORC1
DDX43
DMRTA1
NALCN
NEFH
LDHC
IGKV3D-11

IGHV4-55
APOD
ENSG00000276566
IDO1
TFAP2C
CNTN6
TMEM40
AC010967.2
TTYH1
SDK2
FABP4
ISL1
RNF175
OR2I1P
KRT19
ENSG00000276399
SLC26A4-AS1
NIPAL4
PCSK1
TSPAN10
ZNF750
RP11-84E24.3
RAMP1
LMO3
PDLIM4
PLA2G4D
LCE3E
PRDM13
PITX1
RPH3A
ZNF812
AACSP1
PTPRZ1
IGHV3-7
PCSK9
PCLO
WISP2
SEMA3D
TTC39A
RP11-697M17.1
SLCO1A2
ABCC2
MYBPC1
ADAMDEC1
WFDC12
FAM83F
IGHE

FEZF1-AS1
HRASLS5
AP001065.2
TRIM58
TNNT1
MSI1
BCHE
TTR
BPIFB1
AC010145.3
ANGPT1
NPY6R
CTNND2
CST6
RP11-366F6.2
AQP5
CEACAM5
HIF3A
IGKV1-12
GAL
AC011294.3
TKTL1
RP11-342C23.4
CYP2J2
IGHJ3
UBD
WNK2
AC073218.2
EREG
ATP1B2
IGKV1D-8
AC004988.1
PRKAA2
LCE1A
LPAR3
IL22RA1
DPEP3
FCRL3
SLC35F1
HRASLS
HTN3
S100A2
FLG
HP
DEFB1
CKMT1B
TUBB4A

CADPS
CXorf48
LRRC4B
RP11-138J23.1
SOX11
MYEOV
CBLC
TTLL6
CNTN4
ANXA3
CNTN1
AC141928.1
ZIC2
MEOX2
RP11-252C15.1
CNTN3
CTNNA2
LONRF2
TMEM171
LINGO2
NPY1R
C10orf126
PSAPL1
RP11-276H19.4
GFRA1
RP11-424G14.1
NPPC
RP1-90G24.6
CLDN11
CNR1
TRHDE
RIMS4
PTPN20A
DSCR4
SLC9A3
MARCO
KRT79
FAM155B
RP11-1070N10.5
NKX6-1
NR2E1
HOXB-AS5
ALB
DLL3
LBX1
EPYC
CXCL10

GPR27
NAT16
TRIM71
GNGT1
RP11-203P23.2
GAL3ST1
PCDHB5
NGEF
ENSG00000273259
MYO5B
SFTPC
VCX3A
NKX2-4
TAGLN3
IGKV1-39
MUM1L1
RP11-307C19.2
LAD1
F5
ITGB8
ALOX12B
NTSR1
GDF15
REG3G
SIK1
SLC24A4
AC005281.2
IGKV2-29
RP11-299H22.3
CHI3L1
S100A9
SLC5A12
C2orf70
SORCS1
SCN9A
F2RL2
CHAD
IGLV1-41
BANK1
PNLDC1
COL9A3
PLEKHS1
SCG3
TMPRSS4
SLC6A10P
CTD-2081K17.1
DHRS2

DCX
FCRL2
DLX6-AS1
ART3
SLC6A11
DLGAP1
NRXN1
AC093787.1
LGI4
PRSS35
GGT6
RP11-371I1.2
SLC6A14
C21orf90
SCML4
DES
SHISA3
C1orf106
GRHL2
D86994.2
ENSG00000280061
RP5-884M6.1
ENSG00000278630
RP11-599J14.2
CHRM3
LRRN4CL
SUSD5
HAND2
OVOL1
BLK
PPL
SDR9C7
AC244230.1
GALNTL6
RP11-267A15.1
RP11-129M6.1
PLAC2
CXCL1
TRIM51CP
NKAIN4
EHF
DPYSL5
RPTN
RP11-574H6.1
AC108142.1
SNCB
RP11-396O20.2

GZMK
WNK4
BBOX1
RP1-90G24.10
FCAMR
KCNN2
GAGE2A
KCNQ5
TPSB2
CIDEA
SAA1
IGKV3-7
UCHL1
RP11-180C1.1
AC018865.8
B4GALNT4
C6orf141
LINC00326
LRAT
ZNF503-AS1
BAI1
FOXN1
IRF6
RP11-93B14.5
FCRLA
AIM2
RAB3B
FAM123A
ENSG00000279184
GALNT5
CYSLTR2
TMEM246
S100A1
AP001065.15
CD8A
AC066593.1
HOXC13
PROM2
CTD-2314G24.2
LAMC2
CD8B
LIF
LCE1F
KLHDC8A
HS3ST5
PPP1R1B
AARD

RP3-523K23.2
TNFRSF13B
MAGEL2
KCTD14
LCE1B
TSPAN8
SLITRK5
RP11-128P17.2
OGN
SOX8
DMRT2
LCE2B
ATP1A2
TMPRSS11D
KLC3

Supplementary Table 2. Gene set enrichment analysis results of top 1,000 most variable genes used for

CHOL

Gene Set Name
GO_HUMORAL_IMMUNE_RESPONSE
GO_COMPLEMENT_ACTIVATION
GO_IMMUNOGLOBULIN_COMPLEX
GO_HUMORAL_IMMUNE_RESPONSE_MEDIATED_BY_CIRCULATING_IMMUNOGLOBULIN
GO_B_CELL_MEDIATED_IMMUNITY
GO_ANTIGEN_BINDING
GO_REGULATION_OF_HUMORAL_IMMUNE_RESPONSE
GO_IMPORT_INTO_CELL
GO_SIGNALING_RECEPTOR_BINDING
GO_ADAPTIVE_IMMUNE_RESPONSE

COAD

Gene Set Name
GO_IMMUNOGLOBULIN_COMPLEX
GO_HUMORAL_IMMUNE_RESPONSE
GO_HUMORAL_IMMUNE_RESPONSE_MEDIATED_BY_CIRCULATING_IMMUNOGLOBULIN
GO_COMPLEMENT_ACTIVATION
GO_ANTIGEN_BINDING
GO_B_CELL_MEDIATED_IMMUNITY
GO_SIGNALING_RECEPTOR_BINDING
GO_IMMUNOGLOBULIN_COMPLEX_CIRCULATING
GO_REGULATION_OF_IMMUNE_SYSTEM_PROCESS
GO_ADAPTIVE_IMMUNE_RESPONSE

GBM

Gene Set Name
GO_CELL_CELL_SIGNALING
GO_NEUROGENESIS
GO_CENTRAL_NERVOUS_SYSTEM_DEVELOPMENT
GO_INTRINSIC_COMPONENT_OF_PLASMA_MEMBRANE
GO_SYNAPSE
GO_IMMUNOGLOBULIN_COMPLEX
GO_HUMORAL_IMMUNE_RESPONSE
GO_NEURON_DIFFERENTIATION
GO_SYNAPTIC_SIGNALING
GO_EXTRACELLULAR_MATRIX

KICH

Gene Set Name
GO_INTRINSIC_COMPONENT_OF_PLASMA_MEMBRANE
GO_ION_TRANSPORT
GO_PLASMA_MEMBRANE_REGION
GO_SYNAPSE
GO_NEURON_DIFFERENTIATION

GO_CELL_CELL_SIGNALING
GO_CELL_SURFACE
GO_NEUROGENESIS
GO_IMPORT_INTO_CELL
GO_SIGNALING_RECEPTOR_BINDING

KIRP
Gene Set Name
GO_IMMUNOGLOBULIN_COMPLEX
GO_HUMORAL_IMMUNE_RESPONSE_MEDIATED_BY_CIRCULATING_IMMUNOGLOBULIN
GO_COMPLEMENT_ACTIVATION
GO_HUMORAL_IMMUNE_RESPONSE
GO_ANTIGEN_BINDING
GO_B_CELL_MEDIATED_IMMUNITY
GO_IMPORT_INTO_CELL
GO_REGULATION_OF_HUMORAL_IMMUNE_RESPONSE
GO_ADAPTIVE_IMMUNE_RESPONSE
GO_ADAPTIVE_IMMUNE_RESPONSE_BASED_ON_SOMATIC_RECOMBINATION_OF_IMMUNE_RECEPTO

LIHC
Gene Set Name
GO_HUMORAL_IMMUNE_RESPONSE
GO_IMPORT_INTO_CELL
GO_COMPLEMENT_ACTIVATION
GO_HUMORAL_IMMUNE_RESPONSE_MEDIATED_BY_CIRCULATING_IMMUNOGLOBULIN
GO_IMMUNOGLOBULIN_COMPLEX
GO_ENDOCYTOSIS
GO_ANTIGEN_BINDING
GO_B_CELL_MEDIATED_IMMUNITY
GO_SIGNALING_RECEPTOR_BINDING
GO_SMALL_MOLECULE_METABOLIC_PROCESS

PAAD
Gene Set Name
GO_IMMUNOGLOBULIN_COMPLEX
GO_HUMORAL_IMMUNE_RESPONSE
GO_HUMORAL_IMMUNE_RESPONSE_MEDIATED_BY_CIRCULATING_IMMUNOGLOBULIN
GO_COMPLEMENT_ACTIVATION
GO_B_CELL_MEDIATED_IMMUNITY
GO_ANTIGEN_BINDING
GO_ADAPTIVE_IMMUNE_RESPONSE
GO_SIGNALING_RECEPTOR_BINDING
GO_IMMUNOGLOBULIN_COMPLEX_CIRCULATING
GO_LYMPHOCYTE_MEDIATED_IMMUNITY

PCPG
Gene Set Name

GO_CELL_CELL_SIGNALING
GO_SYNAPSE
GO_INTRINSIC_COMPONENT_OF_PLASMA_MEMBRANE
GO_SIGNALING_RECEPTOR_BINDING
GO_NEUROGENESIS
GO_SYNAPTIC_SIGNALING
GO_NEURON_DIFFERENTIATION
GO_NEURON_PROJECTION
GO_IMMUNOGLOBULIN_COMPLEX
GO_SYNAPTIC_MEMBRANE

SARC
Gene Set Name
GO_NEURON_DIFFERENTIATION
GO_NEUROGENESIS
GO_IMMUNOGLOBULIN_COMPLEX
GO_CELL_CELL_SIGNALING
GO_ANIMAL_ORGAN_MORPHOGENESIS
GO_SIGNALING_RECEPTOR_BINDING
GO_EXTRACELLULAR_MATRIX
GO_HUMORAL_IMMUNE_RESPONSE_MEDIATED_BY_CIRCULATING_IMMUNOGLOBULIN
GO_COMPLEMENT_ACTIVATION
GO_LOCOMOTION

SKCM
Gene Set Name
GO_IMMUNOGLOBULIN_COMPLEX
GO_HUMORAL_IMMUNE_RESPONSE
GO_HUMORAL_IMMUNE_RESPONSE_MEDIATED_BY_CIRCULATING_IMMUNOGLOBULIN
GO_COMPLEMENT_ACTIVATION
GO_ANTIGEN_BINDING
GO_B_CELL_MEDIATED_IMMUNITY
GO_IMMUNOGLOBULIN_COMPLEX_CIRCULATING
GO_IMMUNOGLOBULIN_RECEPTOR_BINDING
GO_ADAPTIVE_IMMUNE_RESPONSE
GO_PHAGOCYTOSIS_RECOGNITION

unsupervised hierarchical clustering in 10 cancer types

Number of Genes in Gene Set (K)	Number of Genes in Overlap (k)	p-value	FDR q-value
352	121	1.83E-105	1.87E-101
170	87	2.20E-94	1.12E-90
144	81	1.73E-92	5.89E-89
147	80	9.92E-90	2.53E-86
219	81	1.80E-73	3.67E-70
155	68	6.53E-68	1.11E-64
133	64	2.95E-67	4.29E-64
810	126	2.21E-64	2.82E-61
1611	170	1.33E-61	1.50E-58
663	112	5.68E-61	5.79E-58

Number of Genes in Gene Set (K)	Number of Genes in Overlap (k)	p-value	FDR q-value
144	89	7.07E-107	7.20E-103
352	108	8.12E-88	4.14E-84
147	74	1.59E-79	5.40E-76
170	75	5.25E-75	1.34E-71
155	69	2.18E-69	4.44E-66
219	75	4.73E-65	8.04E-62
1611	168	5.87E-60	8.55E-57
69	47	7.99E-60	1.02E-56
1670	169	1.90E-58	2.15E-55
663	108	4.90E-57	5.00E-54

Number of Genes in Gene Set (K)	Number of Genes in Overlap (k)	p-value	FDR q-value
1665	175	1.79E-63	1.82E-59
1625	162	2.35E-55	1.20E-51
995	124	1.80E-52	6.12E-49
1744	162	3.43E-51	8.73E-48
1332	139	1.44E-49	2.93E-46
144	53	2.86E-48	4.87E-45
352	74	2.14E-47	3.11E-44
1365	134	9.82E-45	1.25E-41
733	98	4.28E-44	4.85E-41
531	83	1.29E-42	1.20E-39

Number of Genes in Gene Set (K)	Number of Genes in Overlap (k)	p-value	FDR q-value
1744	149	8.75E-47	8.92E-43
1692	125	9.28E-33	4.73E-29
1190	103	1.41E-32	4.81E-29
1332	108	1.09E-31	2.78E-28
1365	108	8.90E-31	1.81E-27

1665	120	1.93E-30	3.28E-27
902	86	3.12E-30	4.07E-27
1625	118	3.20E-30	4.07E-27
810	81	6.38E-30	7.22E-27
1611	113	1.21E-27	1.23E-24

Number of Genes in Gene Set (K)	Number of Genes in Overlap (k)	p-value	FDR q-value
144	80	1.11E-91	1.13E-87
147	67	4.13E-69	2.11E-65
170	69	8.36E-67	2.84E-63
352	90	2.00E-66	5.10E-63
155	64	1.35E-62	2.75E-59
219	69	6.87E-58	1.17E-54
810	111	4.46E-52	6.49E-49
133	53	5.46E-51	6.96E-48
663	100	9.05E-51	1.02E-47
352	75	3.40E-49	3.46E-46

Number of Genes in Gene Set (K)	Number of Genes in Overlap (k)	p-value	FDR q-value
352	84	3.55E-59	3.62E-55
810	114	1.61E-54	8.20E-51
170	60	1.02E-53	3.46E-50
147	56	2.16E-52	5.50E-49
144	55	1.51E-51	3.07E-48
661	92	1.50E-43	2.54E-40
155	50	1.10E-42	1.49E-39
219	57	1.17E-42	1.49E-39
1611	137	1.07E-39	1.21E-36
1962	152	2.48E-39	2.52E-36

Number of Genes in Gene Set (K)	Number of Genes in Overlap (k)	p-value	FDR q-value
144	97	4.26E-122	4.34E-118
352	121	3.14E-105	1.60E-101
147	83	8.41E-95	2.86E-91
170	83	1.28E-87	3.27E-84
219	85	4.68E-79	9.54E-76
155	72	5.15E-74	8.75E-71
663	122	1.04E-70	1.51E-67
1611	179	3.11E-68	3.96E-65
69	50	8.85E-66	1.00E-62
351	90	2.32E-65	2.36E-62

Number of Genes in Gene Set (K)	Number of Genes in Overlap (k)	p-value	FDR q-value

1665	197	8.02E-82	8.18E-78
1332	173	1.21E-77	6.19E-74
1744	187	2.56E-70	8.71E-67
1611	163	2.74E-57	6.99E-54
1625	162	4.94E-56	8.49E-53
733	111	5.00E-56	8.49E-53
1365	147	7.27E-55	1.06E-51
1317	140	1.74E-51	2.21E-48
144	54	6.18E-50	7.00E-47
438	82	1.08E-48	1.10E-45

Number of Genes in Gene Set (K)	Number of Genes in Overlap (k)	p-value	FDR q-value
1365	154	9.05E-58	4.45E-54
1625	168	9.29E-58	4.45E-54
144	60	1.31E-57	4.45E-54
1665	168	2.96E-56	7.53E-53
1071	134	2.32E-55	4.72E-52
1611	161	3.03E-53	5.15E-50
531	94	6.42E-52	9.35E-49
147	54	2.19E-48	2.79E-45
170	56	4.53E-47	4.77E-44
1984	170	4.68E-47	4.77E-44

Number of Genes in Gene Set (K)	Number of Genes in Overlap (k)	p-value	FDR q-value
144	101	1.17E-131	1.19E-127
352	113	4.95E-96	2.52E-92
147	81	1.54E-92	5.22E-89
170	84	1.58E-90	4.02E-87
155	76	1.39E-81	2.83E-78
219	83	2.06E-77	3.51E-74
69	52	1.01E-70	1.47E-67
73	51	1.77E-66	2.26E-63
663	115	2.51E-65	2.84E-62
81	52	1.06E-64	1.08E-61

Supplementary Table 3. Significantly amplified and deleted loci across 7 cancer types

COAD				GBM				
Subtype A		Subtype B		Subtype A		Subtype B		Subty
Amp	Del	Amp	Del	Amp	Del	Amp	Del	Amp
17q12	16p13.3	17q12	16p13.3	12q14.1	9p21.3	7p11.2	9p21.3	7q31.2
13q12.2	6q26	8p11.21	16q23.1	4q12	1p32.3	4q12	10q23.31	2q32.1
8q24.21	4q22.1	8q24.21	20p12.1	7p11.2	12q14.1	12q14.1	1p36.23	10p14
11p15.5	20p12.1	12p13.32	1p33	1q32.1	6q26	1q32.1	6q27	
20q11.21	3p14.2	13q12.13	1p36.11	6p21.1	15q14	7q21.2	14q13.1	
8p11.21	16q23.1	20q13.12	3p14.2	6q21	10q23.31	12p13.32	3q29	
12p13.32	1p33	11p15.5	5q12.1	12p13.32	13q14.2	12q15	10q26.3	
20q13.12	5q12.1	20p11.21	4q35.1		17p13.1	1p36.21	1p32.3	
6p21.1	5q22.2	19q13.12	10q23.31		4q31.23	3q26.33	22q13.31	
19q13.11	6p25.3	18q11.2	6q26		1p36.31	2p24.3	13q14.2	
13q22.1	1p36.11	13q22.1	4q22.1		10q26.3	4p16.3	4q34.3	
8p11.23	18q21.2	5p13.1	18q21.2		10q21.1		16q24.2	
12p11.22	4q34.3	16q12.1	15q21.1		15q21.3		1q44	
8p11.23	3q26.31	6p21.1	6p25.3		14q13.1		13q21.2	
17p11.2	10q21.1	20p12.2	5q21.2		16q23.1		15q15.1	
20p11.21	10q23.31	17q23.3	21q11.2		11p15.4		6p24.1	
20p12.1	18q12.2	7p11.2	18q12.3		14q32.2		10p15.3	
1p33	15q12		8p23.1				19q13.41	
20p13	22q13.32		3q26.31				11p15.5	
8q21.13	17p12		17p12				2q37.2	
	15q22.33		1p31.1				10p13	
	8p22		22q13.32				5q13.1	
	8p23.2		15q11.2				5q34	
	8q11.1		2q22.3				17q11.2	
	7q31.1		2p21					
	10q25.2		17q24.3					
	19p13.3		9p21.3					
	21q21.1		10q21.1					
	15q15.2		2q37.1					
	13q14.13							
	4p16.3							
	17q24.3							
	2q22.1							
	1q42.2							

KIRP			PAAD				PC	
ype A	Subtype B		Subtype A		Subtype B		Subtype A	
Del	Amp	Del	Amp	Del	Amp	Del	Amp	Del
1p36.32		9p21.3			18q11.2	9p21.3	4q31.1	17q11.2
4q34.3		2q37.2			8q24.21	18q21.2	17q21.31	1p12
11q22.3		1p36.22			19q13.2	1p36.11	1q21.3	22q13.32
16q24.1		11q22.3			9p13.3	17q22		3p24.1
9p21.3		14q32.33			7q22.1	17p12		1p36.32
2q36.3		2q22.1			1p12	5q14.2		3q26.31
19p13.3		5q15			17q12	22q13.32		11p15.4
22q13.32		3p14.2			12q15	6p25.3		4q35.2
6p12.3		6q27			12p12.1	22q13.31		17p13.1
14q11.2		15q21.1			8p11.23	12q24.32		9p23
2q22.1		4q31.23			17p11.2	14q11.2		6q16.1
10q21.1		8p23.1			4p16.3	7q36.1		5q35.2
21q22.3					6p21.31	4q35.1		4q22.1
					20p11.23	19p13.3		16q21
					8p11.21	19p13.2		1q44
					12p13.33	11p15.4		3q13.11
					3q26.2	16q23.1		17q21.31
					2q36.2	15q14		
						10p15.3		
						19q13.33		
						21q11.2		
						1p36.32		
						13q14.2		
						6q26		
						10q23.31		
						2q37.3		
						9q22.33		

PG		SARC				SKCM		
Subtype B		Subtype A		Subtype B		Subtype A		Subty
Amp	Del	Amp	Del	Amp	Del	Amp	Del	Amp
		12q15	9p21.3	12q15	13q14.2	1p12	9p21.3	3p13
		1q24.3	4q34.3	1q24.3	2q37.3	11q14.1	10q23.31	11q13.3
		5p15.33	17q11.2	3p12.1	9p21.3		11q24.3	5p15.33
		19q13.11	3q13.31	1p32.1	17p13.1		4q34.3	22q13.2
		22q11.22	3q11.1	6q25.1	10p15.3		1p36.31	1p12
		8q24.21	21q11.2	19q12	4q35.2		5p15.33	12q15
				13q34	8p23.3		2q37.3	1q44
				17p11.2	1p36.32		12q24.33	12q14.1
				5p15.2	19q13.43		6q27	8q24.3
				11q22.2	19p13.3		19p13.3	6p24.3
				1q21.3	11q25		6q13	6q12
				19p13.2	7q36.3		15q13.2	15q26.1
				2p11.2	11p15.5		5q35.3	7p22.3
				15q26.3	17q11.2		10q26.3	4q12
				17q24.3	17p13.1		15q15.1	17q25.3
				8p11.22	17q25.3		12q21.31	7q34
				7q31.2	9q34.3		13q14.2	15q24.3
				21q21.1	10q23.31		5q32	5q35.3
				5q35.3	2p25.3		16q23.3	13q12.3
				6p21.1	1q44		1p22.1	1q21.3
				9p24.1	10q26.3		1q42.13	7q36.1
				14q13.2	18q23		6q23.2	1p36.11
					6q14.1		9p24.3	9p13.3
					21q22.3		17p13.3	9p24.2
					9p24.3			
					2q37.3			
					6p25.3			
					22q13.32			
					16q12.1			
					11q22.3			
					3p21.31			
					5p15.33			
					1p32.3			
					12q12			
					14q24.1			
					3q29			
					12p13.1			
					5q35.3			
					20q13.33			
					3q25.2			
					13q34			
					15q13.2			
					8q24.3			

18p11.32
12q24.33

ype B
Del
9p21.3
10q23.31
11q23.3
4q34.3
2q37.3
1p22.1
1p36.31
5q12.1
10p15.3
5q32
10q26.3
6q26
3q13.31
15q14
16p13.3
15q13.3
15q21.1
19p13.3
9p23
13q34
8p23.3
16q24.2
10q21.1
5p15.31
14q23.3
14q31.1
16q23.1
3q26.31
4p16.3
22q13.32
13q12.11
12q24.33
16q11.2
3p26.1
17p13.3
12q13.12
6q21
17q21.31

Supplementary Table 4. Significantly amplified and deleted genes across 7 cancer types

COAD				GBM				
Subtype A		Subtype B		Subtype A		Subtype B		Subty
Amp	Del	Amp	Del	Amp	Del	Amp	Del	Amp
IKZF3	RBFOX1	ERBB2	RBFOX1	CDK4	CDKN2A	[EGFR]	CDKN2A	CAPZA2
CDX2	PARK2	ANK1	MAF	PDGFRA	CDKN2C	PDGFRA	PTEN	FSIP2
[LOC72767	FAM190A	MYC	FLRT3	[EGFR]	SLC16A7	CDK4	ERRFI1	hsa-mir-54
hsa-mir-48	FLRT3	CCND2	BEND5	MDM4	QKI	MDM4	QKI	CAV1
C20orf160	FHIT	[USP12]	hsa-mir-19	POLH	hsa-mir-14	CDK6	NPAS3	PHYH
PLAT	WWOX	ADA	FHIT	QRSL1	PTEN	CCND2	hsa-mir-31	CAV2
FGF6	BEND5	hsa-mir-48	hsa-mir-58	NDUFA9	hsa-mir-12	CPM	CYP2E1	PRPF18
ADA	hsa-mir-58	GINS1	ING2	CYP27B1	ATP1B2	PRDM2	CDKN2C	MET
BYSL	APC	ZNF527	PTEN	PIK3C2B	NR3C2	[SOX2-OT]	hsa-mir-32	CDC123
ZNF507	FOXC1	[GATA6]	PARK2	MAD2L1BP	hsa-mir-42	DDX1	RB1	ST7
[KLF5]	hsa-mir-19	[KLF5]	FAM190A	LOC100422	CYP2E1	FGFR3	LINC00290	USP6NL
WHSC1L1	SMAD4	hsa-mir-12	SMAD4	DYRK4	hsa-mir-60	GSX2	BANP	TFEC
PTHLH	hsa-mir-13	[LOC38827	B2M	METTL1	hsa-mir-21	TSPAN31	AHCTF1	OPTN
[ZNF703]	NAALADL2	hsa-mir-58	FOXC1	LRRN2	NPAS3	PIK3C2B	hsa-mir-31	TES
C17orf103	hsa-mir-60	PLCB4	RAB9BP1	GTPBP2	hsa-mir-19	C12orf5	hsa-mir-62	CELF2
GINS1	PTEN	hsa-mir-63	RBM11	AKAP3	hsa-mir-48	MDM2	EDN1	ST7-AS1
PCSK2	hsa-mir-92	EGFR	hsa-mir-92	TSPAN31	hsa-mir-20	PDPN	ZMYND11	NUDT5
ELAVL4	hsa-mir-12	GRB7	hsa-mir-54	PLEKHA6	CDKN2B	MYCN	CD33	ST7-AS2
hsa-mir-12	hsa-mir-32	FGF6	NAALADL2	MRPS18A	USP15	LETM1	hsa-mir-42	SEPHS1
FABP4	DNAH9	HNF4A	hsa-mir-74	RAD51AP1	CAHM	MARCH9	CXCR7	ST7-OT4
ZPBP2	SMAD3	IGF2	PTGFR	OS9	hsa-mir-12	MYCNOS	hsa-mir-54	UPF2
PDX1	hsa-mir-42	NINL	hsa-mir-32	PPP1R15B	KLLN	TACC3	CCNB1	SEC61A2
IGF2	hsa-mir-59	ZNF569	hsa-mir-62	XPO5	hsa-mir-75	AGAP2	hsa-mir-34	MCM10
XKR7	FNTA	hsa-mir-58	ACVR2A	GALNT8	SHBG		EVI2A	DHTKD1
AP3M2	LRRN3	BYSL	ZFP36L2	MARCH9	ZBTB48		C9orf53	FRMD4A
FGF23	hsa-mir-42	PLCB1	SOX9	LOC127841	DUX4		KLLN	CAMK1D
HNF4A	hsa-mir-43	CYB561	CDKN2A	RSPH9	MIR605		PDE10A	ECHDC3
CCND3	hsa-mir-54	HPVC1	hsa-mir-60	C12orf4	hsa-mir-628		CPN2	CCDC3
DPY19L3	hsa-mir-62	IKZF3	hsa-mir-56	AGAP2	hsa-mir-3182		DUX4	SFTA1P
PPFIBP1	hsa-mir-12	FGF23	WWOX	LOC10013	hsa-mir-675		FAF1	LOC219731
NINL	hsa-mir-94	WISP2	MACROD2		hsa-mir-4309		FAM19A5	UCMA
SNRPB2	SOX9	INS	hsa-mir-3115		C9orf53		LPAR6	BEND7
AGBL4	hsa-mir-12	ABHD12	PDE4D		FAM19A2		SCCPDH	LOC254312
AVP	hsa-mir-15	ZNF570	IRF2		hsa-mir-627		hsa-mir-12	C10orf47
FABP5	MACROD2	C6	ATAD1		hsa-mir-15a		hsa-mir-12	LOC28307
POLR1D	PDE4D	RUNX2	TRIM69		TP53		GCNT2	LOC43995
INS	FLJ11235	LAMP5	GMDS		TNFRSF9		TUBB8	MIR4480
C12orf4	GMDS	ACE	ABCC13		DUX2		ETFB	
MYBL2	CD52	SEC61G	hsa-mir-4318		hsa-mir-1266		hsa-mir-302e	
SLC29A1	ME2	MIEN1	hsa-mir-596		hsa-mir-1972-2		IQCA1	
LOC40068	MGC45800	DYRK4	MIR4789		hsa-mir-4298		CAMK1D	
MED21	MIR4789	SERINC3	DNAH9		hsa-mir-1247		CDK7	
ABHD12	CSTF2T	TH	IFI44		CDKN2B-AS1		hsa-mir-1229	

KIF16B	PAPSS2	C7	ACR	hsa-mir-4310	EVI2B
GNRH2	hsa-mir-43	CCND3	hsa-mir-4310	hsa-mir-3168	C6orf118
IL7	hsa-mir-31	PAK7	ORC4	RPL22	GP5
GSX1	FAM19A5	MAP3K3	HAAO	MTG1	DUX2
TH	ZNF18	LANCL2	LOC100499467	hsa-mir-147b	LOC284933
C12orf5	hsa-mir-42	MIR4728	CDKN2B	hsa-mir-140	TFB2M
SGK2	CLN8	RAD51AP1	PRKG1	hsa-mir-210	KLF5
GUCA1A	SGK196	PKIG	hsa-mir-1471	hsa-mir-656	hsa-mir-147b
STK38L	IMMP2L	IGF2-AS1	MACROD2-AS1	hsa-mir-626	HIVEP1
OTOR	HABP2	C9	hsa-mir-4253	hsa-mir-621	C10orf108
IDH3B	hsa-mir-19	CDC5L	PART1	KCNAB2	NKG7
IMPA1	hsa-mir-12	ANKRD5	STOX2	SYCE1	hsa-mir-483
LNX2	hsa-mir-43	MRC2	CFL1P1	hsa-mir-1282	ASB18
IGF2-AS1	hsa-mir-75	VOPP1	LOC647946	hsa-mir-1538	LOC283070
SERINC3	hsa-mir-57	PRMT8	ANGPT2	AP2A2	GTF2H2
GUCA1B	LOC10049	C20orf111	MAP2K4	hsa-mir-370	hsa-mir-4281
FGFR1OP2	CCNT2	MIR483	IFI44L	hsa-mir-1233-2	NF1
MACROD2	hsa-mir-11	DAB2	ARSA	hsa-mir-4305	LOC285796
ITPA	MACROD2-	DNAH8	hsa-mir-626	TNFRSF25	HES1
PMP2	PART1	LOC100131	MBD5	PAOX	MTG1
ATP5EP2	FGR	DCAF7	MTA3	hsa-mir-627	LOC339685
MIR483	ELAC1	VSTM2A	MTAP	hsa-mir-328	SMYD3
PKIG	LINC00290	PARP11	CSTF2T	RHOG	DACH1
MEA1	MIR605	JPH2	hsa-mir-1244-1	hsa-mir-345	hsa-mir-1282
TM7SF3	ATAD1	INS-IGF2	hsa-mir-1256	hsa-mir-1233-1	MAK
MACROD2-	MIR4318	FGF10	TRAPPC11	ALOX5AP	KLK10
OXT	hsa-mir-31	SLC29A1	KLLN	PER3	hsa-mir-675
PKIA	LOC28493	TLK2	MIR4318	SPRNP1	MIR4480
PRHOXNB	hsa-mir-54	LOC28587	DEFA1	hsa-mir-4310	CD180
INS-IGF2	MYOM2	C12orf4	ZNF18	AARS	hsa-mir-1271
IFT52	HGSNAT	GDAP1L1	ELTD1	ART1	OMG
PEX6	EIF3IP1	MIR4686	CHKB	hsa-mir-342	DKFZp451B082
CCDC91	NRAP	FYB	hsa-mir-1233-2	hsa-mir-211	LSG1
PTPRA	hsa-mir-31	GLO1	THADA	ATP7B	SYCE1
TPD52	hsa-let-7c	DDX42	C9orf53	VAMP3	FLJ46257
MIR4686	hsa-mir-62	FKBP9L	MIR605	FRG2B	LOC149134
C20orf111	hsa-mir-15	C12orf5	ALPI	hsa-mir-626	LMO7
POLH	ADD1	LOC79015	hsa-mir-1290	AP1G1	hsa-mir-627
ASUN	DARS	GDNF	ENPP6	ASCL2	NEDD9
SNRPB	ACTN2	GLP1R	DEFA3	hsa-mir-3173	KLK11
STMN2	IFI6	TANC2	MIR744	ACTC1	hsa-mir-4298
JPH2	MIR1305	EFCAB4B	CPT1B	BRCA2	NAIP
PPP2R5D	CFL1P1	TTPAL	hsa-mir-1233-1	KLHL21	hsa-mir-585
ARNTL2	NBEAP1	GHR	PLEKHH2	SPRN	MIR4733
TGM3	MIR3201	GUCA1A	CDKN2B-AS1	hsa-mir-1233-2	CAHM
HEY1	hsa-mir-32	TACO1	LOC100506939	AFG3L1P	ATP13A3
GDAP1L1	DLGAP2	TOX2	ALPP	CARS	PAOX

PTK7	POTEA	HMGCS1	ALPL	hsa-mir-1260	MIR3201
KLHDC5	TCF7L2	GUCA1B	RWDD4	APBA2	CNST
ProSAPiP1	hsa-mir-13	CCDC47	DEFA4	RCBTB2	PCDH9
MRPS28	CXADR	FITM2	TYMP	UTS2	hsa-mir-4310
LOC79015	BUB1B	IL7R	hsa-mir-211	LOC619207	TFAP2A
PRPH2	hsa-mir-31	HSP90AB1	OXER1	hsa-mir-1233-1	KLK8
MRPS35	ADRA2C	LIMD2	ALPPL2	AGRP	hsa-mir-210
NOP56	HNMT	R3HDML	C1QA	CD81	PIK3R1
ZC2HC1A	AGT	LIFR	LOC728175	hsa-mir-625	hsa-mir-218-2
TTPAL	SFN	MDFI	DEFA5	B2M	TMEM44
SRF	KLLN	KCNH6	MAPK11	CPB2	SPRNP1
C12orf70	IPW	MIR3646	hsa-mir-1268	PARK7	MIR4535
UBOX5	MIR4535	OXCT1	LOC728819	DUX4L7	LOC255654
PAG1	hsa-mir-54	MEA1	CHRND	hsa-mir-211	ATXN8OS
TOX2	ARHGEF10	STRADA	C1QB	APRT	hsa-mir-626
TBCC	TECTB	LOC10050	DEFA6	CD151	GCM2
REP15	AES	PRKAA1	MAPK12	hsa-mir-548h-1	KLK13
C20orf194	NCAM2	MOCS1	hsa-mir-3118-6	BUB1B	ADM
SNX16	CAPN3	MARCH10	LOC100129726	ELF1	RAD17
FITM2	hsa-mir-62	PTGER4	CHRNG	ACOT7	hsa-mir-103-1-as
VEGFA	ATP5I	NFKBIE	C1QC	DUX4L6	LRRC15
C12orf71	LCT	LOC72968	DEFB1	ACTC1	FRG2B
SNORD57	LYST	RPL37	SBF1	ZFHX3	UCHL3
ZBTB10	GPR3	NFYA	hsa-mir-3118-4	CDKN1C	hsa-mir-1233-2
R3HDML	NDN	MIR548W	HTR2B	hsa-mir-4308	TMEM14C
MED20	hsa-mir-38	SEPP1	CAPZB	CAPN3	SIGLEC7
MANSC4	KBTBD11	PEX6	DEFB4A	ESD	AP2A2
SNORD56	ACSL5	SKP2	PPP6R2	CAMTA1	SMN1
ZFAND1	AMH	PGC	ACTC1	DUX4L5	hsa-mir-146a
GTSF1L	TMPRSS15	SLC1A3	SP110	ADAM10	FAM43A
POLR1C	CKMT1B	POLH	CASP9	C16orf3	SPRN
CPXM1	hsa-mir-43	ZNF131	CLN8	CTSD	TBC1D4
CHMP4C	CRMP1	PPP2R5D	ZBED4	hsa-mir-3172	hsa-mir-1233-1
MIR3646	MCM6	OSMR	APBA2	CHRM5	ELOVL2
MAD2L1BP	GNG4	PTK7	INPP5D	FOXO1	SIGLEC9
EBF4	HMGN2	NUP155	RUNX3	ICMT	AMPD3
SLC10A5	SNRPN	PRPH2	MYOM2	DUX4L3	SMN2
LOC10050	NAT1	PAIP1	SCO2	APBA2	hsa-mir-3142
CUL7	FBXO25	SRF	NBEAP1	CA5A	XXYLT1
FASTKD5	ZDHHC6	MRPS30	KCNJ13	DRD4	LOC619207
ZNF704	ATP5D	TBCC	CDA	hsa-mir-624	PIBF1
C6orf108	BTG3	NNT	DLGAP2	CHRNA7	ACTC1
VPS16	EPB42	VEGFA	RABL2B	FLT1	PAK1IP1
FABP9	ATP7B	TTC33	BUB1B	CHD5	KLK14
CNPY3	CTBP1	TFEB	NCL	DUX4L2	APBB1
FAM113A	MGAT5	NIPBL	CDC42	AQP9	TAF9
FABP12	KCNK1	SUPT3H	MFHAS1	CA7	hsa-mir-1303

FRS3	RPS6KA1	FBXO4	MLC1	DUSP8	FLJ34208
MRPS26	UBE3A	KCNK5	CAPN3	hsa-mir-3171	DUX4L7
SLC22A7	NAT2	WDR70	NEU2	CKMT1B	KLF12
DDRGK1	CSMD1	NCR2	CD52	MLNR	B2M
CAPN11	VTI1A	CCL28	ARHGEF10	ERRFI1	TMEM14B
ZNF343	AZU1	MED20	MAPK8IP2	MIR3944	KLK12
CUL9	USP25	C5orf28	CHRM5	B2M	RHOG
SLC4A11	GANC	POLR1C	NPPC	CALB2	SERF1A
UBR2	RCBTB2	C5orf42	RCC1	HBB	hsa-mir-3141
TMC2	DGKQ	MAD2L1BP	KBTBD11	hsa-mir-4307	LOC100131551
KIAA0240	CXCR4	PARP8	PLXNB2	EPB42	DUX4L6
C20orf141	LGALS8	CUL7	CHRNA7	GTF2F2	DIS3
ZNF318	SLC9A1	SPEF2	PDE6D	HES2	BUB1B
STK35	MKRN3	RCAN2	CLCNKA	BUB1B	ADTRP
YIPF3	ADRA1A	CARD6	SPAG11B	CBFA2T3	CTU1
TGM6	C8orf42	C6orf108	BRD1	HBBP1	ART1
USP49	LOC14318	LMBRD2	GABRA5	hsa-mir-208b	SMA4
SNORA51	HCN2	CNPY3	PSMD1	GANC	hsa-mir-1294
GNMT	CHODL-AS1	EMB	CLCNKB	GUCY1B2	LOC100507391
SNORD86	GCHFR	FRS3	FBXO25	DNAJC11	DUX4L5
PRICKLE4	CPB2	HEATR7B2	FAM19A5	CAPN3	PCDH17
SNORD110	EVC	SLC22A7	GABRB3	CBFB	CAPN3
MRPL2	KYNU	EGFLAM	PTMA	HBD	C6orf228
SNORD119	MTR	APOBEC2	CNR2	hsa-mir-1201	CLDND2
GTPBP2	ARID1A	NADKD1	AGPAT5	GATM	ASCL2
LOC10013	PAR5	CAPN11	NCAPH2	HMGB1	SMA5
MRPS18A	ASAH1	UGT3A1	GABRG3	PLEKHG5	hsa-mir-378
TMEM239	ERICH1	ENPP4	SNORD20	CHRM5	DUX4L3
MRPS10	GUCY2GP	CAPSL	DDOST	CDH1	KLHL1
MIR1292	BSG	CUL9	DEFB103B	HBE1	CHRM5
TMEM63B	LINC00308	LOC15368	MOV10L1	SERPINA3	SYCP2L
TRERF1	ITPKA	UBR2	GANC	GCHFR	VSIG10L
XPO5	ELF1	UGT3A2	SAG	HTR2A	CARS
MRPL14	FGFR3	DAAM2	E2F2	NOL9	SLC30A5
DLK2	ZEB2	NIM1	CSMD1	CHRNA7	hsa-mir-145
TTBK1	NID1	KIAA0240	MIOX	CDH3	DUX4L2
RRP36	NR0B2	RANBP3L	GCHFR	HBG1	PCDH20
ABCC10	SNURF	ZNF318	SP100	ACTN1	CHRNA7
KLC4	ATP6V1B2	RICTOR	ECE1	PDIA3	LINC00518
TJAP1	ZNF596	YIPF3	MCPH1	KPNA3	KLK9
KLHDC3	MIR4295	C5orf51	PANX2	TAS1R1	CCKBR
TAF8	CDC34	USP49	IPW	CKMT1B	CENPH
PTCRA	C21orf91	PLCXD3	SPP2	CDH5	hsa-mir-584
C6orf223	IVD	GNMT	EPHA2	HBG2	MIR3944
RSPH9	ESD	HCN1	PPP1R3B	ACYP1	BORA
LRRC73	GAK	PRICKLE4	ALG12	ITPKA	CKMT1B
RPL7L1	NXPH2	C5orf34	ITPKA	LCP1	C6orf52

CRIP3	TARBP1	MRPL2	DGKD	ESPN	SIGLECP3
C6orf226	FCN3	C5orf39	EPB41	CYP19A1	CD81
ATP6V0CP3	CYFIP1	CLIC5	ERI1	CDH11	MRPS36
C6orf132	BMP1	SNORD72	CRELD2	HRAS	ADRA1B
LOC100132	LOC286083	TREM2	IVD	AKT1	TDRD3
TOMM6	CIRBP	LOC646719	GPR55	IVD	CYP19A1
	CHODL	TREM1	EPHA8	MAB21L1	ERVFRD-1
	LTK	LOC648987	CLDN23	THAP3	C19orf75
	FOXO1	GTPBP2	ADM2	DUT	CD151
	GRK4	MIR580	LTK	CDH13	MARVELD2
	RAB3GAP1	MRPS18A	TRIP12	IGF2	ADRB2
	TBCE	CCDC152	EPHB2	ANG	DIAPH3
	MAP3K6	MRPS10	DEFB104A	LTK	DUT
	C15orf2	LOC100132	TRABD	SMAD9	TMEM170B
	POLR3D	TMEM63B	MEIS2	PHF13	IGLON5
	OR4F21	MIR3650	ECEL1	EPB42	CDKN1C
	CNN2	SAYSD1	EXTL1	CDH15	CCDC125
	C21orf91-C	LOC100506	SGK223	INS	ANXA6
	MAP1A	TRERF1	SELO	APEX1	KCTD12
	MLNR	EGFLAM-AS	TRPM1	MAP1A	EPB42
	HTT	LRFN2	EIF4E2	NEK3	LOC100130275
	UBXN4	AARS2	EYA3	NPHP4	TPP1
	GPR137B	XPO5	C8orf42	FBN1	MAST4
	WASF2	ENPP5	HDAC10	CDH16	ATOX1
	MAGEL2	ZFAND3	NDN	IRF7	PRR20A
	BNIP3L	MRPL14	ARL4C	ARF6	FBN1
	RPL23AP53	DLK2	FGR	MEIS2	LOC100506409
	CSNK1G2	TREML2	ERICH1	PCDH8	CNGA4
	D21S2088E	KCNK16	SHANK3	GPR153	LOC647859
	PLCB2	TTBK1	OCA2	FGF7	BNIP1
	GTF2F2	RRP36	NMUR1	COX4I1	COMMD6
	HGFAC	KCNK17	FUCA1	KCNQ1	FGF7
	R3HDM1	ABCC10	ZNF596	ARG2	CTSD
	TSNAX	KLC4	TUBGCP6	MFAP1	GUSBP3
	CNKSR1	TJAP1	PLCB2	UBL3	CAMK2A
	ATP10A	BTBD9	SP140	RNF207	OR7E156P
	CHRNA2	FOXP4	IFI6	GABPB1	GABPB1
	MIR596	KLHDC3	DEFT1P	CTRB1	DRD4
	CFD	TAF8	LOC90834	LSP1	GTF2H2B
	LINC00317	PTCRA	RAD51	ARHGAP5	CANX
	RAD51	TCTE1	SH3BP4	TRPM1	LINC00550
	GUCY1B2	SPATS1	GALE	RB1	GALK2
	IDUA	C6orf223	DEFB105A	HES3	DUSP8
	LRP1B	RSPH9	LMF2	GALK2	GTF2H2C
	GNPAT	LRRC73	RYR3	CTRL	CCNG1
	NUDC	TREML2P1	NGEF	MUC2	LINC00347
	NIPA2	LOC221442	SFN	BCL2L2	GANC

CLU	C6orf130	DEFB106A	PLCB2	EIF4G2
LOC100287	KIF6	KLHDC7B	RFC3	SERF1B
ARID3A	TSPO2	SCG5	MIR4252	CD74
LINC00320	UNC5CL	SNORD82	GANC	MZT1
SPINT1	TREML4	GPR3	CYBA	GATM
HTR2A	RPL7L1	DEFB107A	MUC6	HBB
LETM1	TREML1	LOC284933	BDKRB1	GTF2H2D
ARHGAP15	TREML3	SNRPN	RAD51	CDX1
GGPS1	SLC35B2	GIGYF2	RFXAP	CTAGE11P
WDTC1	FLJ41649	HMGN2	MIR4689	GCHFR
SNORD107	CRIP3	FLJ10661	GATM	HBBP1
DPYSL2	C6orf226	RPL23AP82	DHODH	GUSBP9
EFNA2	TMEM151E	SPINT1	NAP1L4	CLTB
LINC00478	ATP6V0CP3	CAB39	BDKRB2	PRR20B
SRP14	C6orf132	HMGCL	RYR3	PDIA3
KPNA3	MIR586	LOC286083	SLC7A1	HBD
LRPAP1	TDRG1	C22orf34	MIR4417	LOC100170939
GTDC1	LOC100131	SRP14	GCHFR	CSF1R
TOMM20	LOC100132	UGT1A10	NQO1	PRR20C
STX12	TOMM6	HSPG2	NUP98	HDC
TUBGCP5	MIR4647	LOC349196	BMP4	HBE1
EGR3	MIR4641	CHKB-CPT1B	SCG5	LOC100272216
ELANE	MIR4642	THBS1	TPT1	CSNK1A1
MIRLET7C		UGT1A8	PDIA3	PRR20D
THBS1		HTR1D	DYNC1LI2	ONECUT1
LCP1		XKR5	SLC22A18	HBG1
MSX1		IL17REL	ZFP36L1	OCLN
YSK4		TJP1	SORD	NKX2-5
CAPN9		UGT1A7	TRPC4	PRR20E
AHDC1		HTR6	HDC	ITPKA
NIPA1		DEFB103A	DPEP1	HBG2
EPB49		FAM116B	SLC22A18AS	DBN1
GAMT		TYRO3	CALM1	MIR1297
MIR125B2		UGT1A6	SPINT1	IVD
TP53BP1		ID3	TNFSF11	HPX
MAB21L1		OR4F21	ONECUT1	DIAPH1
MYL5		PIM3	E2F4	MIR3169
THSD7B		UBE3A	POLR2L	LTK
COG2		UGT1A5	SERPINA6	HRAS
ZNF593		STMN1	SRP14	DOCK2
PAR1		FAM90A13	SUCLA2	MAP1A
EPHX2		ODF3B	ITPKA	IGF2
GNA11		MKRN3	FANCA	DPYSL3
MIR99A		UGT1A9	PSMD13	MEIS2
TYRO3		MFAP2	ENTPD5	ILK
SMAD9		FAM90A5	THBS1	DRD1
PDE6B		SYCE3	DLEU2	MFAP1

TMEM163	PAR5	IVD	INS
RBM34	UGT1A4	FOXF1	SLC26A2
GPN2	NBL1	RNH1	MYO5A
LOC283683	FAM90A7	CDKN3	IRF7
EXTL3	LOC100128946	TJP1	DUSP1
GNA15	JMJD7-PLA2G4B	TSC22D1	NEDD4
C21orf37	UGT1A1	LIPC	KCNQ1
JMJD7-PLA2G4B	OPRD1	FOXL1	EBF1
NEK3	FAM90A8	MRPL23	PLCB2
PPP2R2C	LOC100144603	CEBPE	LMO1
ZRANB3	SNAP23	TP53BP1	F12
DISC2	UGT1A3	CCNA1	MAPK6
AIM1L	PAFAH2	LTK	LSP1
OR4N4	FAM90A18	FOXC2	FABP6
PTK2B	MIR3201	RPLP2	RAB27A
GNG7	HERC2	CFL2	MUC2
SNAP23	PID1	TYRO3	FAT2
PCDH8	PAX7	DCLK1	RAD51
RGS12	FAM90A9	MAP1A	MUC6
LYPD1	MIR4535	GALNS	FGF1
DISC1	SNURF	RRM1	RYR3
PIGV	ATG16L1	FOXN3	NAP1L4
HERC2P3	PLA2G2A	SLC30A4	FGFR4
FGL1	FAM90A10	KL	SCG5
MKNK2	AQR	MEIS2	NUP98
TGM5	USP40	GAS8	FOXI1
RB1	PLA2G5	SCT	SLC12A1
RNF4	DEFA10P	CHGA	SLC22A18
ACMSD	ARHGAP11A	EIF3J	FLT4
ARID4B	HJURP	ITM2B	SORD
GPATCH3	PPP1R8	MFAP1	SLC22A18AS
GOLGA6L1	DEFB107B	GCSH	GABRA1
GFRA2	SLC12A6	TRIM21	SPINT1
GPX4	COPS7B	CKB	POLR2L
PPIP5K1	PTAFR	JMJD7-PLA2G4B	GABRA6
RFXAP	DEFB104B	MTRF1	SRP14
SH3BP2	RASGRP1	TRPM1	PSMD13
SPOPL	TRPM8	GLG1	GABRB2
EGLN1	RAP1GAP	STIM1	TCF12
CEP85	DEFB106B	CMA1	RNH1
GOLGA8IP	GPR176	SNAP23	GABRG2
GNRH1	ARMC9	UTP14C	THBS1
GZMM	RHCE	MYO1E	MRPL23
LCMT2	DEFB105B	HAS3	GABRP
TPT1	CHP	TALDO1	TP53BP1
WFS1	EFHD1	LTB4R	RPL27A
NCKAP5	RHD	HERC2	GLRA1

KIAA1383	DEFB109P1B	FRY	TYRO3
LIN28A	OIP5	MYO5A	RPLP2
SNORD108	ITM2C	HP	GM2A
LOXL2	RPA2	TH	SLC30A4
GADD45B	RPL23AP53	CRIP1	RRM1
RASGRP1	BAHD1	SLC28A2	GRK6
TRPC4	DNER	LPAR6	EIF3J
WHSC1	RPL11	NEDD4	SCT
DKFZp686O1327	FAM90A14	HPR	GPX3
HEATR1	FAN1	TSPAN4	JMJD7-PLA2G4B
DHDDS	B3GNT7	CRIP2	SMPD1
SNORD109A	RPS6KA1	TGM5	GRIA1
LPL	SPAG11A	SLC25A15	SNAP23
NFIC	MAPKBP1	PLCB2	TRIM21
GPR176	SP140L	HSBP1	NR3C1
TNFSF11	RSC1A1	TNNI2	USP8
WHSC2	MIR596	CTSG	ST5
MIR128-1	GOLGA8A	PPIP5K1	GRM6
ERO1LB	DIS3L2	LHFP	SLC28A2
SH3BGRL3	SDHB	MAPK6	STIM1
SNORD109B	DEFA1B	HSD11B2	HK3
MSR1	RTF1	TNNT3	CCPG1
OAZ1	SPATA3	DAD1	TAF10
CHP	SLC9A1	AQR	HMMR
SUCLA2	FAM90A20	TRIM13	COPS2
ZNF141	CYFIP1	RAB27A	TALDO1
LOC647012	FBXO36	HSD17B2	HNRNPAB
SIPA1L2	TAF12	TRPC2	TGM5
TMEM222	FAM90A19	COCH	TEAD1
SNORD115-1	MGA	ARHGAP11A	HNRNPH1
NEFM	SLC16A14	USPL1	PIGB
PALM	TCEA3	RAD51	TH
OIP5	FAM66B	HSF4	HRH2
DLEU2	VPS39	PHLDA2	PPIP5K1
SLBP	LOC151475	DIO2	TSPAN4
PABPC1P2	TCEB3	LCMT2	NDST1
ARV1	ZNF705G	MRPS31	AQR
ZDHHC18	FAM189A1	RYR3	TNNI2
WHAMMP3	LINC00471	IRF8	HTR4
NEFL	ZBTB17	ZNF195	SECISBP2L
POLR2E	FAM66E	DIO3	TNNT3
BAHD1	C15orf2	SLC12A6	IL12B
TSC22D1	LOC151484	DLEU1	ARHGAP11A
NOP14	SLC30A2	SCG5	TRPC2
LOC100129961	LOC100132396	KARS	ITK
TTC13	TMEM87A	RASSF7	LCMT2
SYTL1	MSL3P1	DLST	PHLDA2

POTEB	LUZP1	RASGRP1	KCNMB1
NKX3-1	LOC100287015	N4BP2L2	SLC12A6
POLRMT	RPAP1	SLC12A1	TUB
MAPKBP1	C2orf57	LCAT	LCP2
CCNA1	ARID1A	IFITM1	BCL2L10
FAM193A	DEFT1P2	DYNC1H1	WEE1
ZEB2-AS1	DKFZP434L187	SERF2	LTC4S
PCNXL2	TIGD1	OLFM4	RASGRP1
UBXN11	SNHG3	SORD	ZNF143
SNORD64	DEFB4B	MAF	MFAP3
PCM1	GREM1	BRSK2	SERF2
PRTN3	C2orf72	EIF2S1	ZNF195
RTF1	NR0B2	GPR176	MGAT1
DCLK1	MIR548I3	POSTN	GNB5
C4orf6	RPUSD2	SPINT1	ZNF214
MIR3679	ECEL1P2	MC1R	MSX2
C1orf124	FCN3	TSPAN32	ARPP19
FAM46B	MIR4659A	EIF5	ZNF215
PAR4	EHD4	CHP	NPM1
PDGFRL	LOC348761	HSPH1	SLC27A2
PTBP1	AKR7A2	SRP14	RASSF7
MGA	MIR4660	CHST6	PCDH1
ITM2B	NDUFAF1	TSSC4	GPR176
MFSD10	C2orf82	ELK2AP	PPFIBP2
LOC100507600	ALDH4A1	OIP5	PCDHGC3
NTPCR	MIR4659B	SUGT1	CHP
C1orf172	NUSAP1	TCF12	IFITM1
PAR-SN	DNAJB3	MVD	PDE6A
PNOC	EIF4G3	TRIM22	OIP5
RPS15	LOC100652791	EML1	OR6A2
VPS39	TMEM85	BAHD1	PDGFRB
MTRF1	PRSS56	LECT1	BAHD1
MAEA	MAP3K6	THBS1	DCHS1
KIAA1804	SPTBN5	NFATC3	POU4F3
FAM76A	SNORA75	IFITM3	CEP152
LOC348120	C1orf38	ERH	EIF3F
PPP2R2A	KLF13	FAN1	PPP2R2B
SGTA	SCARNA6	WBP4	MAPKBP1
CCNDBP1	CROCC	TJP1	BRSK2
UTP14C	MAGEL2	CHMP1A	MAPK9
PCGF3	SCARNA5	DEAF1	GOLGA8A
C1orf198	ZBTB40	ESR2	MICAL2
ZNF683	DLL4	MAPKBP1	PROP1
GOLGA8E	LOC100286922	AKAP11	RTF1
PPP3CC	CELA3A	TP53BP1	CTR9
STK11	INO80	PLCG2	RARS
TMEM87A	MIR1471	IFITM2	MGA

LPAR6	WASF2	ESRRB	TRIM66
SPON2	PPP1R14D	GOLGA8A	RPS14
C1orf131	MIR1244-1	EXOSC8	DMXL2
CATSPER4	HNRNPR	TYRO3	TSPAN32
OR4M2	MTMR10	PSKH1	SGCD
SFTPC	MIR1244-3	OR7E12P	VPS39
TCF3	SRRM1	FKBP3	TSSC4
RPAP1	ZNF770	RTF1	SLC6A7
SLC25A15	MIR1244-2	FNDC3A	AP4E1
TACC3	CNKSR1	SLC30A4	MRVI1
EDARADD	HAUS2	PSMB10	SLC34A1
TRNP1	MIR4777	KCNQ1OT1	CCNDBP1
OR4N3P	GMEB1	FOXG1	TRIM22
SLC7A2	FAM82A2	MGA	SLIT3
THOP1	NUDC	PDS5B	EID1
RPUSD2	DNAJC17	EIF3J	IFITM3
LHFP	SRSF10	PSMD7	SNCB
MXD4	NOP10	PKP3	C15orf63
SLC35F3	RCAN3	FNTB	DEAF1
CD164L2	NDNL2	VPS39	SPARC
HERC2P2	MST1P2	KIAA0564	TMEM87A
SLC18A1	C15orf24	JMJD7-PLA2G4B	IPO7
TLE2	MST1P9	RPL13	SPINK1
TUBGCP4	PAK6	SIRT3	RPAP1
TRIM13	PADI2	FOS	IFITM2
CPLX1	CASC5	FAM189A1	STK10
B3GALNT2	LYPLA2	ZC3H13	PYGO1
LOC644961	AVEN	SNAP23	TRIM3
NF1P2	DNAJC8	RRAD	TAF7
STC1	ATP10A	OR52A1	PLDN
MADCAM1	AKR7A3	FUT8	OR7E12P
EHD4	GJD2	CCNDBP1	ZNF354A
MRPS31	SPEN	SPG20	GREM1
SLC26A1	VPS18	ALDH1A2	LYVE1
EXOC8	KDM1A	ST3GAL2	TCOF1
SCARNA1	ZFP106	PGAP2	RPUSD2
LOC503519	WDTC1	GALC	KCNQ1OT1
FZD3	CHAC1	C15orf63	TTC1
SF3A2	KIAA0090	LRCH1	TUBGCP4
NDUFAF1	C15orf29	USP8	PKP3
DLEU1	PLEKHM2	SLC9A5	STC2
D4S234E	NIPA2	C11orf21	SCG3
IRF2BP2	OTUD3	GCH1	SWAP70
MIR1976	C15orf41	TMEM87A	PCDHGB4
CHEK2P2	DNAJC16	MTUS2	TMOD3
TUSC3	ZFYVE19	CCNB2	DENND5A
PPAP2C	UBR4	SLC12A4	ADAM19

NUSAP1	DISP2	TRPM5	TMOD2
OLFM4	ATP13A2	GMFB	RRP8
STX18	CHRFAM7A	RPAP1	FGF18
FAM89A	CELA3B	INTS6	EHD4
LOC646214	ULK4P3	SLC28A2	SIRT3
ADAM7	PADI4	SNTB2	HDAC3
S1PR4	ULK4P1	UBQLN3	DUOX2
SPTBN5	TMEM50A	GPR33	OR52A1
POSTN	ARHGAP11B	DKFZP434L187	SQSTM1
FGFRL1	STX12	CKAP2	MYEF2
C1orf31	ATPBD4	CCPG1	ARFIP2
CXADRP2	CLIC4	SPG7	ATP6V0E1
TNFRSF10D	C15orf57	IGF2-AS1	NDUFAF1
AP3D1	SYF2	GPX2	OR5E1P
DLL4	C15orf23	PLDN	PTTG1
SUGT1	C1orf144	NUFIP1	RSL24D1
CYTL1	BMF	COPS2	OR10A3
TRIM67	LDLRAP1	TAT	PDLIM7
REREP3	SNORD107	BET1L	NUSAP1
TNFRSF10C	PLA2G2D	GSTZ1	FXC1
MED16	CHST14	GREM1	CNOT8
INO80	RNU11	NBEA	TMEM85
LECT1	TUBGCP5	TGM5	DKK3
PIGG	HSPB7	TERF2	HAND1
SNRPD2P2	NIPA1	CEND1	SPTBN5
LOC653061	AHDC1	GTF2A1	RBMXL2
TNFRSF10B	PLA2G4E	RPUSD2	MED7
FSTL3	SMPDL3B	PCDH17	CTDSPL2
PPP1R14D	PAR1	PIGB	PGAP2
WBP4	LINC00339	TK2	ADAMTS2
STK32B	LOC145845	TRIM34	DUOX1
SNORA14B	PADI1	BRF1	C11orf21
SNORD116-19	TMCO5A	TUBGCP4	RNF14
TNFRSF10A	PLA2G2E	C13orf15	DLL4
APC2	CSNK1A1P1	PPIP5K1	TRPM5
HAUS2	HP1BP3	ZNF19	CLINT1
AKAP11	OTUD7A	CDHR5	INO80
LYAR	CELA2B	GZMH	UBQLN3
LOC100287814	SPRED1	EHD4	PCDHGA8
GOLGA6L6	ZNF593	MED4	ZNF280D
FGF17	PGBD4	AQR	RNF141
ABCA7	MRTO4	ZNF23	MAML1
FAM82A2	EXD1	TOLLIP	TRPM7
EXOSC8	YTHDF2	GZMB	IGF2-AS1
KIAA1530	FSIP1	DUOX2	KIAA0141
LGALS8-AS1	PADI3	DNAJC15	PPP1R14D
LOC727924	RHOV	SECISBP2L	BET1L

DOK2	WNT4	GAN	JAKMIP2
HMG20B	LPCAT4	TRIM68	ZNF770
DNAJC17	FBXO42	HIF1A	CEND1
FNDC3A	PLA2G4F	NDUFAF1	GFPT2
ZFYVE28	RNF186	ALG5	FLJ10038
MIR1182	LRRC57	ARHGAP11A	CYB5R2
GOLGA8C	GPN2	SLC7A5	GNPDA1
MTMR7	C15orf55	PIDD	HAUS2
UQCR11	FBLIM1	FOXA1	TRIM34
PAK6	LOC283683	NUSAP1	G3BP1
KIAA0564	MED18	VPS36	FAM82A2
TNIP2	OR4N4	LCMT2	CDHR5
MIR1537	PQLC2	CDK10	TNIP1
PWRN1	LOC283710	KCNQ1DN	DNAJC17
ENTPD4	TRNAU1AP	HNRNPC	TOLLIP
SBNO2	FAM98B	TMEM85	GNB2L1
CASC5	AIM1L	PHF11	MNS1
ZC3H13	PLA2G4D	SLC12A6	USP47
HAUS3	XKR8	TRADD	SLU7
LINC00184	HERC2P3	MMP26	NOP10
PWRN2	ARHGEF10L	HSPA2	TRIM68
PHYHIP	GOLGA6L1	SPTBN5	RGS14
HMHA1	TMEM57	POMP	MYO5C
STARD9	GOLGA8G	BCL2L10	PIDD
SPG20	CAMK2N1	MBTPS1	CPLX2
ABCA11P	GOLGA8IP	CHRNA10	FAM214A
TSNAX-DISC1	ASAP3	HSP90AA1	KCNQ1DN
SNORD116-1	SNORD108	CTDSPL2	C5orf4
SORBS3	PNRC2	UFM1	C15orf24
SHC2	SNORD109A	RASGRP1	PARVA
VPS18	PIGV	CES2	FAM114A2
LRCH1	SNORD109B	PNPLA2	PAK6
MFSD7	NBPF1	IFI27	MMP26
LOC100506795	SNORD115-1	KLF13	TCERG1
SNORD116-2	NECAP2	SOHLH2	DTWD1
NPM2	WHAMMP3	SERF2	AKIP1
TIMM13	RCC2	NAE1	BTNL3
ZFP106	POTEB	PHRF1	CASC5
INTS6	FAM54B	ITPK1	C11orf16
TMEM175	FMN1	DUOX1	LMAN2
LOC100506810	C1orf63	ENOX1	AVEN
SNORD116-3	SNORD64	GNB5	TMEM9B
PNMA2	PITHD1	NOL3	SPINK5
DAZAP1	PAR4	SIGIRR	GJD2
CHAC1	MAN1C1	JAG2	NRIP3
CKAP2	PAR-SN	DLL4	SOX30
TMEM128	NIPAL3	RCBTB1	STARD9

MIR4753	LOC348120	ARPP19	ASCL3
SNORD116-4	SEPN1	TAF1C	MGAT4B
ADAM28	MRPL42P5	RIC8A	VPS18
FGF22	KIF17	KLC1	CHRNA10
TMEM62	C15orf52	INO80	B4GALT7
NUFIP1	GRHL3	NUDT15	SQRDL
C4orf42	GOLGA8E	SLC27A2	PNPLA2
MIR4671	IL22RA1	SLC7A6	SYNPO
SNORD116-5	OR4M2	EPS8L2	ZFP106
LZTS1	GPATCH3	KTN1	PHRF1
SLC39A3	OR4N3P	PPP1R14D	RNF44
ZFYVE19	PLA2G2F	KIAA1704	RFX7
NBEA	HERC2P2	GPR176	SCUBE2
TMEM129	CEP85	USP10	ABLIM3
MIR4427	C15orf53	CHID1	SPATA5L1
SNORD116-6	PINK1	LGALS3	ZBED5
XPO7	C15orf54	MTMR10	HMGXB3
THEG	PHACTR4	FAM48A	CHAC1
DISP2	MIR211	CHP	SIGIRR
C13orf15	C1orf135	ATP6V0D1	TBC1D9B
EVC2	NF1P2	OR51G1	C15orf29
SNORD116-7	RSG1	LTBP2	RIC8A
TRIM35	HERC2P9	ZNF770	ARHGAP26
FZR1	MUL1	THSD1	ATP8B4
C15orf57	WHAMMP2	OIP5	MRPL17
MED4	LIN28A	BCAR1	ATP10B
OTOP1	GOLGA8B	OR51B4	WDR76
SNORD116-8	AGMAT	MARK3	EPS8L2
RHOBTB2	EIF2AK4	HAUS2	N4BP3
LSM7	FAM110D	CYSLTR2	TMEM62
C15orf23	LOC503519	BAHD1	STK33
DNAJC15	DHDDS	C16orf7	FAF2
ZNF595	C15orf62	OR51B2	SEMA6D
SNORD116-9	ZNF436	MAX	CHID1
PSD3	C15orf56	FAM82A2	WWC1
MBD3	TAS1R2	SPRYD7	SPG11
BMF	PHGR1	FAN1	OR51G1
ALG5	ACTL8	KIAA0513	FBXW11
JAKMIP1	CHEK2P2	OR52N1	ELL3
SNORD116-10	SH3BGRL3	MGAT2	OR51B4
SLC39A14	LOC646214	DNAJC17	LARP1
MIER2	SESN2	COG6	C15orf48
CHST14	CXADRP2	CEP152	OR51B2
VPS36	TMEM222	PIEZO1	ZNF346
FAM53A	LOC646278	SLC25A22	C15orf41
SNORD116-11	USP48	CTAGE5	OR52N1
FGF20	REREP3	NOP10	GEMIN5

PCSK4	NBPF3	NAA16	SPPL2A
TGM7	LOC653061	MAPKBP1	RIC3
PHF11	ZDHHC18	DHX38	PCDHGA12
ZBTB49	LOC653075	ATHL1	ZFYVE19
SNORD116-12	DDI2	ATXN3	SLC25A22
ADAMDEC1	MIR626	NDNL2	CCDC69
C19orf24	TRIM63	RNASEH2B	CGNL1
CATSPER2	MIR627	GOLGA8A	ATHL1
UFM1	CROCCP2	IST1	TSPAN17
ZNF721	ANP32AP1	OR51G2	FRMD5
SNORD116-13	SYTL1	MAP3K9	OR51G2
CNOT7	SNORD116-19	C15orf24	OR4F3
PLEKHJ1	IGSF21	DHRS12	DISP2
PLA2G4E	GOLGA6L6	RTF1	OR51E2
SOHLH2	SNHG12	ATP2C2	HAVCR1
ZNF718	LOC727924	OR51E2	ULK4P3
SNORD116-14	C1orf201	MMP14	PTDSS2
ZDHHC2	OIP5-AS1	PAK6	CYFIP2
BTBD2	UBXN11	KIAA0226L	ULK4P1
TMCO5A	GOLGA8C	MGA	MOB2
ENOX1	ATPIF1	CLEC3A	PRELID1
CRIPAK	PWRN1	PTDSS2	ATPBD4
SNORD116-15	CROCCP3	ALDH6A1	SBF2
SLC25A37	PWRN2	CASC5	IL17B
RNF126	RAB42	PROSER1	C15orf57
ZSCAN29	SNORD116-1	DMXL2	FAM160A2
RCBTB1	FAM46B	CHST4	MAT2B
LOC285484	SNORD116-2	MOB2	C15orf23
SNORD116-16	C1orf172	MNAT1	MICALCL
SCARA3	SNORD116-3	AVEN	MRPL22
NCLN	IFFO2	CDADC1	BMF
TTBK2	SNORD116-4	VPS39	TRIM5
NUDT15	MYOM3	MPHOSPH6	TLX3
DOK7	SNORD116-5	TRIM5	SHF
SNORD116-17	KLHDC7A	MTHFD1	SYT8
KCTD9	SNORD116-6	GJD2	KCNIP1
SPPL2B	VWA5B1	CAB39L	DUOXA1
CDAN1	SNORD116-7	FAM189A1	PRKCDBP
KIAA1704	UBXN10	NUTF2	ZNF354C
FLJ35424	SNORD116-8	SYT8	CHST14
SNORD116-18	ARHGEF19	MYH6	ODF3
PIWIL2	SNORD116-9	STARD9	RPL26L1
WDR18	C1orf213	CCDC70	CASC4
STRC	SNORD116-10	AP4E1	OSBPL5
FAM48A	PDIK1L	COX4NB	C5orf45
RNF212	SNORD116-11	ODF3	TGM7
SNORD116-20	C1orf64	MYH7	LRRC56

ELP3	SNORD116-12	VPS18	DCTN4
REXO1	IL28RA	COG3	CATSPER2
SPRED1	SNORD116-13	CCNDBP1	MRGPRE
THSD1	FAM43B	TUBB3	PCDH12
C4orf10	SNORD116-14	OSBPL5	LEO1
SNORD116-21	PAQR7	NDUFB1	ART5
INTS10	SNORD116-15	SQRDL	DDX41
ZNF77	FAM76A	SETDB2	PLA2G4E
ADAL	SNORD116-16	EID1	TRIM78P
CYSLTR2	AKR7L	CFDP1	NOP16
NAT8L	SNORD116-17	LRRC56	TRIM69
SNORD116-22	TMCO4	NEDD8	TRIM6
CCDC25	SNORD116-18	ZFP106	LARS
CELF5	ZNF683	KATNAL1	C15orf43
EXD1	SNORD116-20	C15orf63	OR52E2
SPRYD7	MDS2	CTCF	HMP19
C4orf44	SNORD116-21	MRGPRE	LOC145663
SNORD116-23	LOC284632	NFATC4	OR52J3
INTS9	SNORD116-22	SPATA5L1	UIMC1
TLE6	SLC25A34	KBTBD7	LOC145783
FSIP1	SNORD116-23	TMEM87A	OR51L1
COG6	ESPNP	NFAT5	RAB24
FAM86EP	SNORD116-24	ART5	FLJ27352
SNORD116-24	LOC339505	NFKBIA	OR51A7
CSGALNACT1	SNORD116-25	CHAC1	RBM27
LPPR3	FAM131C	EBPL	LOC145845
RHOV	SNORD115-2	RPAP1	OR51S1
NAA16	PADI6	WWP2	NEURL1B
POLN	SNORD116-26	TRIM78P	TMCO5A
SNORD116-25	SPATA21	NOVA1	OR51F2
HR	SNORD116-27	C15orf29	FAM193B
ZNF556	CATSPER4	C13orf33	ZSCAN29
UBR1	SNORD115-3	DKFZP434L187	OR52R1
RNASEH2B	TMEM82	PRDM7	TMED9
C4orf48	SNORD115-4	TRIM6	TTBK2
SNORD115-2	TRNP1	PNP	OR52M1
PBK	SNORD115-5	WDR76	CDHR2
OR4F17	CD164L2	KBTBD6	CDAN1
PLA2G4F	SNORD115-6	PYGO1	OR52K2
DHRS12	PLA2G2C	DDX19B	CCDC99
LOC402160	SNORD115-7	OR52E2	STRC
SNORD116-26	SH2D5	NRL	OR5P2
ZNF395	SNORD115-8	TMEM62	THG1L
FAM108A1	C1orf130	STARD13	DYX1C1
LRRC57	SNORD115-9	PLDN	OR5P3
KIAA0226L	LDLRAD2	GABARAPL2	GALNT10
ZNF876P	SNORD115-10	OR52J3	CSNK1A1P1

SNORD116-27	UQCRHL	SIX6	OR2D3
BIN3	SNORD115-11	SPG11	NHP2
DOHH	MINOS1	N4BP2L1	OTUD7A
FAM98B	SNORD115-12	GREM1	OR2D2
PROSER1	LOC644961	MON1B	RBM22
ZNF732	SNORD115-13	OR51L1	SPRED1
SNORD115-3	LOC646471	OTX2	OR52W1
MTUS1	SNORD115-14	ELL3	RNF130
KLF16	LOC653566	EPSTI1	PGBD4
PLA2G4D	SNORD115-15	RPUSD2	OR56A4
CDADC1	SCARNA1	TCF25	PCDHGC5
SCARNA22	SNORD115-16	OR51A7	ADAL
SNORD115-4	SNORA44	OXA1L	OR56A1
KIAA1967	SNORD115-17	C15orf48	PCDHGC4
DOT1L	SNORA61	ARL11	EXD1
MRPL42P5	SNORD115-18	TUBGCP4	SYT9
CAB39L	SNORA16A	PHLPP2	PCDHGB7
MIR943	SNORD115-19	OR51S1	FSIP1
SNORD115-5	SNORD99	PAX9	OR52B4
SH2D4A	SNORD115-20	C15orf41	PCDHGB6
KISS1R	LOC729059	WDFY2	RHOV
C15orf52	SNORD115-21	SCG3	C11orf40
CCDC70	FLJ37453	ZCCHC14	PCDHGB5
LOC100129917	SNORD115-22	OR51F2	C15orf33
SNORD115-6	MIR1976	SERPINA5	OR52I2
PDLIM2	SNORD115-23	ZFYVE19	PCDHGB3
LMNB2	MIR3115	CG030	UBR1
C15orf53	SNORD115-25	TMOD3	OR51E1
COG3	MIR4253	KIAA0182	PCDHGB2
LOC100130872	SNORD115-26	OR52R1	PATL2
SNORD115-7	MIR3917	PCK2	UBQLNL
EBF2	SNORD115-29	FRMD5	PCDHGB1
MUM1	MIR3675	LINC00284	LPCAT4
C15orf54	SNORD115-30	TMOD2	LOC143666
SETDB2	LOC100506730	ATMIN	PCDHGA11
LOC100133461	SNORD115-31	OR52M1	PLA2G4F
SNORD115-8	LOC100506801	PGF	OR10A5
FAM160B2	SNORD115-32	DISP2	PCDHGA10
MIDN	LOC100506963	CSNK1A1L	LRRC57
EIF2AK4	SNORD115-33	EHD4	OR2AG1
KBTBD7	C1orf151-NBL1	COTL1	PCDHGA9
TMED11P	SNORD115-34	OR52K2	LYSMD2
SNORD115-9	MIR4695	SERPINA1	DNHD1
NUDT18	SNORD115-35	CHRFAM7A	PCDHGA7
R3HDM4	MIR4684	RXFP2	C15orf55
CATSPER2P1	SNORD115-36	DUOX2	SCGB1C1
EBPL	MIR378F	MLYCD	PCDHGA6

LOC100507266
SNORD115-10
DOCK5
C19orf6
CKMT1A
KBTBD6
MIR378D1
SNORD115-11
FLJ14107
TPGS1
C15orf62
EPSTI1
MIR4800
SNORD115-12
REEP4
REEP6
C15orf56
ARL11
HTT-AS1
SNORD115-13
STMN4
IZUMO4
PHGR1
WDFY2
SNORD115-14
CHMP7
SCAMP4
MIR626
LINC00284
SNORD115-15
VPS37A
ADAT3
MIR627
CSNK1A1L
SNORD115-16
NKX2-6
ZNF554
OIP5-AS1
PRR20A
SNORD115-17
SGCZ
GRIN3B
LOC100131089
FAM216B
SNORD115-18
PEBP4
ZNF57
SNORD115-37
RCAN3AS
SNORD115-38
SNORD115-39
SNORD115-40
SNORD115-41
SNORD115-42
SNORD115-43
SNORD115-44
SNORD116-28
SNORD116-29
SNORD115-48
SNORD115-24
SNORD115-27
SNORD115-28
SNORD115-45
SNORD115-47
LOC100128714
LOC100131089
ANKRD63
HERC2P7
GOLGA8F
GOLGA8DP
JMJD7
PLA2G4B
ULK4P2
LOC100288615
LOC100288637
LOC100289656
MIR1233-1
MIR1233-2
MIR4310
MIR3942
LOC100505648
LOC100507466
MIR4509-1
MIR4509-2
MIR4508
MIR4510
MIR4509-3
MIR4715
TMCO5B
OR56A4
SERPINA4
ULK4P3
TEX26
MYEF2
SF3B3
OR56A1
PIGH
ULK4P1
PRR20A
NDUFAF1
CES3
OR52B4
PNN
ARHGAP11B
FAM216B
RSL24D1
ADAT1
C11orf40
POLE2
ATPBD4
LACC1
NUSAP1
CHST5
OR52I2
PPM1A
C15orf57
LINC00330
TMEM85
EDC4
OR51E1
PPP2R5C
C15orf23
HNRNPA1L2
SPTBN5
PLA2G15
UBQLNL
PPP2R5E
BMF
ST13P4
CTDSPL2
COG4
LOC143666
PRKCH
SHF
B3GALTL
KLF13
WDR72
C11orf42
PCDHGA5
HMGN2P46
NLRP6
PCDHGA4
SLC24A5
NS3BP
PCDHGA3
PRTG
OR56B4
PCDHGA2
LOC283663
LOC255512
PCDHGA1
FAM98B
OR52B2
PCDHGB8P
PLA2G4D
C11orf35
NMUR2
GLDN
OR51F1
KIAA1191
FMN1
OR51B5
ERGIC1
MRPL42P5
KRT8P41
CLK4
USP50
LOC283104
ODZ2
TEX9
CSNK2A1P
CNOT6
C15orf52
OR51V1
KCTD16
TNFAIP8L3
H19
HMHB1
SHC4
EFCAB4A
C5orf54
CTXN2
TMEM80

ANKRD63	PLEKHG4	NSD1
LACC1	SCGB1C1	C15orf53
SNORD115-19	PRKD1	OR10A4
CDCA2	DUOXA1	GMCL1P1
JSRP1	DGKH	C15orf54
JMJD7	DUOX1	OLFML1
LINC00330	LRRC29	ARAP3
SNORD115-20	NLRP6	DUOXA2
ESCO2	LGMN	LOC283299
MOB3A	CHST14	RMND5B
PLA2G4B	CCDC122	GOLGA8B
HNRNPA1L2	DLL4	C11orf36
SNORD115-21	CPNE7	RANBP17
FBXO16	NS3BP	EIF2AK4
MFSD12	PSEN1	NLRP10
MIR4310	CASC4	PCYOX1L
ST13P4	STOML3	CATSPER2P1
SNORD115-22	INO80	NLRP14
C8orf48	VPS4A	CCNJL
GIPC3	OR56B4	UNC13C
LOC100505648	PSMA3	ANO9
DGKH	TGM7	SH3TC2
SNORD115-23	EEF1DP3	CKMT1A
R3HCC1	FAM63B	LOC338651
C19orf21	IL17C	PANK3
CCDC122	LOC255512	SERINC4
SNORD115-25	PSMA6	B4GALNT4
LGI3	CATSPER2	SAP30L
PLK5	FAM194B	C15orf62
STOML3	RNF111	OR52L1
SNORD115-26	NOB1	BTNL8
LOC254896	OR52B2	C15orf56
C2CD4C	PSMB5	OR2AG2
FAM194B	PLA2G4E	DOK3
SNORD115-29	SPERT	PHGR1
LOC286059	ZNF280D	OR52B6
CIRBP-AS1	TMEM208	ZFP2
SPERT	C11orf35	LOC645212
SNORD115-30	PSMC1	OR10A2
EFHA2	TRIM69	RUFY1
C19orf25	DLEU7	MIR626
DLEU7	TRPM7	OVCH2
SNORD115-31	FHOD1	CPEB4
LOC286114	OR51F1	MIR627
ATP8B3	PSMC6	PDDC1
FAM124A	C15orf43	PRR7
SNORD115-32	FAM124A	MIR628

SCARA5
DIRAS1
TPTE2P3
SNORD115-33
LOC389641
ZNF555
CTAGE10P
SNORD115-34
C8orf80
C19orf26
SLC25A30
SNORD115-35
MIR320A
CSNK1G2-AS1
SUGT1P3
SNORD115-36
MIR383
C19orf77
SIAH3
SNORD115-37
C8orf58
ODF3L2
KCNRG
SNORD115-38
LOC100128993
ADAMTSL5
LINC00282
SNORD115-39
MIR4287
TMPRSS9
FREM2
SNORD115-40
MIR4288
NDUFS7
NEK5
SNORD115-41
MIR3622A
C19orf35
THSD1P1
SNORD115-42
MIR3622B
WASH5P
KCTD4
SNORD115-43
LOC100507156
ONECUT3
NHLRC3
PPP1R14D
ANKRD11
OR51B5
PSME1
LOC145663
TPTE2P3
MTMR10
ZDHHC1
OR51V1
PSME2
LOC145845
CTAGE10P
ZNF770
OSGIN1
H19
PTGDR
TMCO5A
SLC25A30
FLJ10038
PARD6A
EFCAB4A
PTGER2
ZSCAN29
PAN3
HAUS2
CKLF
TMEM80
ABCD4
TTBK2
SUGT1P3
FAM82A2
NIP7
C11orf36
PYGL
CDAN1
SIAH3
DNAJC17
FAM96B
ANO9
RABGGTA
STRC
KCNRG
MNS1
GINS2
LOC338651
RAD51B
CSNK1A1P1
GALNTL4
NDFIP1
ANP32AP1
MRGPRG
OR4F16
LOC728758
KRTAP5-1
FBXO38
OIP5-AS1
KRTAP5-3
YIPF5
MIR147B
KRTAP5-4
TRIM7
LOC100129387
IFITM5
TIGD6
LOC100131089
FAM99A
SPRY4
ANKRD63
OR56B1
MXD3
LOC100132724
GVINP1
THOC3
JMJD7
OR52K1
SPINK7
PLA2G4B
OR52I1
TRIM52
ULK4P2
OR51D1
HAVCR2
LOC100288615
OR52A5
AGXT2L2
MIR1233-1
OR51B6
C5orf62
MIR1266
OR51M1
FCHSD1
MIR1282
OR51Q1
UNC5A

SNORD115-44	LINC00282	LOC100306975
MEX3D	NOP10	OR51I1
SERP2	TPPP3	TRIM41
SNORD116-28	B4GALNT4	MIR1233-2
FLJ45445	MOK	OR51I2
SERTM1	OTUD7A	BOD1
SNORD116-29	SLC46A3	MIR4310
PRSS57	MYO5C	OR52D1
LINC00547	TRAPPC2L	COL23A1
SNORD115-48	OR52L1	MIR3942
FAM138F	ARID4A	OR52H1
LINC00548	SPRED1	TIMD4
SNORD115-24	FREM2	LOC100505648
LINGO3	NDNL2	OR52N4
MIR15A	WWOX	ZNF300
SNORD115-27	OR52B6	LOC100507466
FAM138A	RNASE1	OR52N5
MIR16-1	PGBD4	MYOZ3
SNORD115-28	NEK5	SERF2-C15ORF63
C19orf71	FAM214A	OR52N2
ALG11	BCMO1	UBTD2
SNORD115-45	PDDC1	DYX1C1-CCPG1
LOC100288123	RNASE2	OR52E6
TSC22D1-AS1	ADAL	SCGB3A1
SNORD115-47	THSD1P1	MIR4510
MIR1909	C15orf24	OR52E8
TTL	TERF2IP	SFXN1
LOC100128714	MRGPRG	MIR4716
MIR1227	RNASE3	OR52E4
SERPINE3	EXD1	GPRIN1
HERC2P7	KCTD4	MIR4713
MIR3187	PAK6	OR56A3
SNORA31	PRMT7	PWWP2A
GOLGA8DP	KRTAP5-1	MIR4712
MIR4321	RNASE4	OR56A5
MIR621	FSIP1	C1QTNF2
MIR4509-1	NHLRC3	TMCO5B
MIR4745	DTWD1	OR10A6
CCDC169	NECAB2	SCGB3A2
MIR4509-2	KRTAP5-3	MYZAP
PRR20B	RNASE6	OR51T1
MIR4508	RHOV	ZNF354B
PRR20C	SERP2	OR51A4
MIR4509-3	CASC5	C5orf47
PRR20D	KLHDC4	OR51A2
MIR4715	KRTAP5-4	PPARGC1B
PRR20E	RPL36AL	IFITM10

TPT1-AS1
MIR1297
MIR548F5
MIR759
MIR320D1
MIR4305
MIR3613
OR7E37P
SPG20OS
LOC100507240
LOC100509894
CCDC169-SOHLH2
MIR4703
LOC100616668

UBR1
SERTM1
AVEN
HYDIN
IFITM5
RPS29
PATL2
LINC00547
GJD2
DEF8
FAM99A
RTN1
LPCAT4
LINC00548
STARD9
DUS2L
OR56B1
SALL2
PLA2G4F
MIR15A
VPS18
CHTF8
OR52K1
SEL1L
LRRC57
MIR16-1
SQRDL
TXNL4B
OR52I1
SRSF5
C15orf55
LOC440131
ZFP106
BANP
OR51D1
SIX1
HMGN2P46
ALG11
RFX7
PDPR
OR52A5
SLC8A3
LOC283710
TSC22D1-AS1
SPATA5L1
RFWD3
OR51B6

C5orf58
MIR210
OR2Y1
KRTAP5-5
AFAP1L1
KRTAP5-2
GRPEL2
KRTAP5-6
LSM11
TMEM41B
GPR151
LOC440028
ZNF300P1
TRIM6-TRIM34
NUDCD2
MIR483
UBLCP1
SNORA3
SLC36A2
SNORA52
SPINK13
LOC644656
C5orf41
RASSF10
LOC153469
LOC650368
BTNL9
LOC653486
PPP1R2P3
SNORA23
FAM71B
SNORA45
PRELID2
SNORA54
SH3RF2
SNORD97
PLAC8L1
INS-IGF2
RNF145
MUC5B
HIGD2A
LOC729013
FAM153B
MIR675
LOC202181
MRVI1-AS1
STK32A

SLC10A1	FAM99B
FAM98B	SLC36A1
ZAR1L	LOC100133161
CHAC1	EIF4E1B
LRRC36	MRPL23-AS1
OR51M1	RASGEF1C
SNAPC1	MIR4298
PLA2G4D	LOC257358
TTL	MIR4299
C15orf29	EFCAB9
DDX19A	MTRNR2L8
OR51Q1	SH3PXD2B
SOS2	MIR210HG
GOLGA8G	LOC285593
SERPINE3	LOC100506305
SLTM	FAM153A
FBXL8	MIR4686
OR51I1	ARL10
SPTB	MIR4485
FMN1	RELL2
SNORA31	MIR4687
ATP8B4	LOC285626
SMPD3	LOC285627
OR51I2	LOC285629
SRP54	SLC36A3
MRPL42P5	KIF4B
MIR621	OR2V2
WDR76	ZNF454
ZNF821	C5orf60
OR52D1	LOC340037
SSTR1	ARSI
C15orf52	PFN3
CCDC169	ZNF879
TMEM62	IRGM
ZDHHC7	FBLL1
OR52H1	NIPAL4
STYX	C5orf25
C15orf53	FLJ38109
PRR20B	C5orf46
SEMA6D	ARHGEF37
VAC14	SPINK6
OR52N4	MIR103A1
TEP1	MIR143
C15orf54	MIR145
PRR20C	MIR146A
SPG11	MIR218-2
FTSJD1	SPINK14

OR52N5	FNDC9
TGFB3	FLJ16171
DUOXA2	MIR340
PRR20D	SNORD95
ELL3	SNORD96A
DDX28	LOC643201
OR52N2	GRXCR2
TGM1	SPINK9
MIR211	ZFP62
PRR20E	CBY3
POLR2M	FAM153C
TSNAXIP1	SNORA74B
OR52E6	MIR585
NKX2-1	MIR143HG
HERC2P9	LOC728554
LINC00426	LOC729080
C15orf48	AACSP1
CENPN	LOC729678
OR52E8	OR4F29
TNFAIP2	FAM196B
WHAMMP2	LOC100132062
TPT1-AS1	LOC100132287
C15orf41	LOC100133331
C16orf61	C5orf52
OR52E4	LOC100268168
TRAF3	MIR1229
GOLGA8B	MIR103B1
PAN3-AS1	MIR3142
SPPL2A	MIR3141
THAP11	MIR4281
OR56A3	MIR3912
TSHR	LOC100505658
EIF2AK4	LOC100507387
MIR1297	MIR4634
ZFYVE19	MIR4638
JPH3	MIR378E
OR56A5	LOC100859930
VRK1	
CATSPER2P1	
MIR548F5	
CGNL1	
PDP2	
OR51T1	
WARS	
CKMT1A	
MIR759	
FRMD5	

RANBP10
OR51A4
XRCC3
SERINC4
MIR320D1
DISP2
VAT1L
OR51A2
YY1
C15orf62
MIR4305
CHRFAM7A
KIAA1609
IFITM10
ZBTB25
C15orf56
MIR3613
ULK4P3
WFDC1
MIR210
PABPN1
PHGR1
OR7E37P
ULK4P1
PDF
KRTAP5-5
DPF3
LOC645212
TEX26-AS1
ARHGAP11B
DPEP2
KRTAP5-2
GPR68
LOC646278
SPG20OS
ATPBD4
DPEP3
KRTAP5-6
TCL1A
LOC653075
LOC100507240
C15orf57
MTHFSD
TRIM6-TRIM34
GPR65
MIR626
LOC100509894

C15orf23
ACD
MIR483
GEMIN2
MIR627
CCDC169-SOHLH2
BMF
DBNDD1
SNORA52
NUMB
ANP32AP1
MIR4703
SHF
FA2H
LOC650368
ADAM21
LOC728758
LOC100616668
DUOXA1
FAM65A
LOC653486
ADAM20
OIP5-AS1
LDHAL6B
TMEM231
SNORA54
ADAM6
MIR147B
CHST14
TMCO7
INS-IGF2
DLK1
LOC100131089
CASC4
WDR59
MUC5B
CCNK
ANKRD63
TGM7
ELMO3
MIR675
CDKL1
GOLGA8F
CATSPER2
KLHL36
FAM99B
DCAF5

JMJD7
LEO1
FBXO31
LOC100133161
ALKBH1
PLA2G4B
PLA2G4E
ESRP2
MRPL23-AS1
EIF2B2
ULK4P2
TRIM69
CENPT
MIR4298
AP1G2
LOC100288615
C15orf43
C16orf70
MIR210HG
SLC7A7
LOC100288637
LOC145663
CYB5B
MIR4686
MTA1
LOC100289656
FAM81A
CMIP
MIR4687
NEMF
MIR1233-1
GCOM1
GFOD2
PNMA1
MIR1282
LOC145783
CDT1
RPS6KA5
MIR1233-2
FLJ27352
MAP1LC3B
TRIP11
MIR4310
LOC145845
PMFBP1
CPNE6
MIR3942

TMCO5A
DYNLRB2
NRXN3
LOC100505648
ZSCAN29
HSDL1
AKAP6
LOC100507466
TTBK2
CRISPLD2
AKAP5
SERF2-C15ORF63
CDAN1
C16orf48
SPTLC2
MIR4509-1
STRC
SLC7A6OS
BAG5
MIR4509-2
DYX1C1
COG8
C14orf2
MIR4510
CSNK1A1P1
SPIRE2
CDC42BPB
MIR4509-3
OTUD7A
ZNF469
TCL1B
TMCO5B
SPRED1
B3GNT9
RGS6
PGBD4
CIRH1A
KIAA0391
ADAL
ZNRF1
KIAA0247
EXD1
CNTNAP4
KIAA0586
FSIP1
MARVELD3
DLGAP5

RHOV
MTSS1L
KIAA0125
C15orf33
CENPBD1
KIAA0317
UBR1
ZNF276
TOX4
PATL2
KCNG4
TECPR2
LPCAT4
SDR42E1
GOLGA5
PLA2G4F
CMTM1
REC8
LRRC57
PKD1L2
MED6
LYSMD2
RNF166
PARP2
C15orf55
EXOSC6
CNIH
WDR72
C16orf46
DHRS2
HMGN2P46
DNAAF1
GPHN
SLC24A5
NRN1L
EFS
PRTG
CMTM3
IRF9
LOC283663
SPATA2L
PRMT5
LOC283710
C16orf55
SEC23A
FAM98B
ZC3H18

VTI1B
PLA2G4D
CDYL2
FBLN5
GOLGA8G
TMEM170A
BATF
GLDN
SLC38A8
TM9SF1
FMN1
ZFP90
SIVA1
MRPL42P5
RLTPR
NPC2
USP50
KCTD19
AHSA1
TEX9
CMTM4
EXOC5
C15orf52
CMTM2
CGRRF1
TNFAIP8L3
BEAN1
CYP46A1
SHC4
SLC22A31
EDDM3A
CTXN2
IL34
DHRS4
C15orf53
TMED6
PAPOLA
C15orf54
FLJ30679
ACOT2
DUOXA2
LOC146513
TMED10
MIR211
ZFPM1
FERMT2
HERC2P9

ADAD2
RIPK3
WHAMMP2
ZFP1
PTPN21
GOLGA8B
ADAMTS18
AP4S1
EIF2AK4
MGC23284
C14orf1
CATSPER2P1
LINC00311
WDHD1
UNC13C
LDHD
BAZ1A
CKMT1A
FUK
MAP4K5
SERINC4
MLKL
SUPT16H
C15orf62
ZNF778
NID2
C15orf56
ACSF3
VASH1
PHGR1
CCDC79
ATG14
LOC645212
CES4A
ZBTB1
LOC646278
EXOC3L1
SNW1
LOC653075
LINC00304
ACIN1
HSP90AB4P
LOC283867
PCNX
MIR626
HTA
DAAM1

MIR627
LOC283922
SAMD4A
MIR628
NUDT7
TTLL5
ANP32AP1
PDXDC2P
FAM179B
LOC728758
CLEC18C
RCOR1
OIP5-AS1
SNAI3
SYNE2
MIR147B
FAM92B
PACS2
LOC100129387
ATXN1L
SCFD1
LOC100131089
PKD1L3
KHNYN
ANKRD63
CLEC18A
ANGEL1
GOLGA8F
CTU2
PPP1R13B
LOC100132724
C16orf86
DICER1
JMJD7
PABPN1L
SLC7A8
PLA2G4B
LOC400548
ZFYVE26
ULK4P2
LOC400550
TTC9
LOC100288615
LOC400558
KLHDC2
LOC100288637
C16orf74

FLRT2
LOC100289656
MIR140
HECTD1
MIR1233-1
CTRB2
HEATR5A
MIR1266
MIR328
NGDN
MIR1282
CLEC18B
LRP10
LOC100306975
SNORD68
PLEKHG3
MIR2116
KIAA0895L
SIPA1L1
MIR1233-2
SNORD71
DCAF4
MIR4310
SNORD111
KIF26A
MIR3942
LOC727710
C14orf109
LOC100505648
LOC729513
NKX2-8
LOC100507466
LOC732275
TINF2
SERF2-C15ORF63
SNORD111B
PLEK2
DYX1C1-CCPG1
LOC100128881
TIMM9
MIR4509-1
LOC100129617
OR10G3
MIR4509-2
LOC100130015
OR10G2
MIR4510

LOC100130894
OR4E2
MIR4716
SYCE1L
TCL6
MIR4713
LOC100287036
MLH3
MIR4509-3
MIR1538
ATP5S
MIR4712
MIR1972-1
KCNH5
TMCO5B
MIR1910
CIDEB
MYZAP
SNORA70D
PRO1768
MIR3182
CHMP4A
MIR1972-2
STXBP6
LOC100505865
GPR132
LOC100506083
POMT2
LOC100506172
STRN3
C16orf95
SLC39A2
CKLF-CMTM1
ERO1L
MIR4720
COQ6
MIR4722
FAM158A
MIR4719
ATL1
FCF1
RDH11
SERPINA10
NIN
GLRX5
ZNF219
COX16

GMPR2
SLC22A17
DACT1
ATP6V1D
EVL
C14orf129
JKAMP
CINP
MBIP
DHRS7
C14orf166
ASB2
SIX4
ZFYVE1
CPSF2
KCNK10
GNG2
KLHL28
C14orf101
HAUS4
PPP2R3C
PRPF39
C14orf119
FBXO34
CDCA4
C14orf102
RNF31
ATG2B
RBM23
UBR7
DNAAF2
C14orf105
EXD2
VRTN
MIS18BP1
SYNJ2BP
SLC39A9
MEG3
C14orf167
G2E3
FLVCR2
OSGEP
C14orf118
SMEK1
ARHGEF40
BTBD7
MUDENG

TDP1
ZNF839
SPATA7
EAPP
ACTR10
YLPM1
METTL3
LTB4R2
KCNK13
C14orf162
SDR39U1
C14orf132
DDX24
RPGRIP1
ADCK1
TMEM63C
PELI2
RHOJ
NDRG2
GALNTL1
PLEKHH1
NYNRIN
TXNDC16
TRMT5
UNC79
HOMEZ
BEGAIN
CHD8
FANCM
PPP4R4
CCNB1IP1
ZNF410
NGB
RBM25
SNX6
SAV1
C14orf93
ABHD4
C14orf133
NPAS3
SMOC1
MOAP1
DIO3OS
EDDM3B
IRF2BPL
MPP5
CDH24

INF2
C14orf135
GPR135
METTL17
IL25
GNPNAT1
BCL11B
OTUB2
ZFYVE21
MEG8
THTPA
OR4K5
OR11H2
WDR25
OR4K1
METTL21D
LINC00341
ZC2HC1C
C14orf169
IPO4
CLMN
CATSPERB
ZC3H14
RIN3
L2HGDH
C14orf159
C14orf45
NUBPL
DCAF11
TMEM121
DDHD1
OR4K15
SGPP1
TMX1
AMN
SLIRP
DNAL1
RPS6KL1
SYT16
IFI27L2
FSCB
SETD3
BRMS1L
APOPT1
HHIPL1
JPH4
C14orf142

RNASE7
INSM2
ARHGAP5-AS1
RAB2B
AJUBA
STON2
ZFHX2
KIAA1737
RPPH1
SLC25A21
PAPLN
FAM181A
BTBD6
EFCAB11
LRRC16B
PPP1R3E
TMEM55B
CHURC1
C14orf43
LIN52
NEK9
EXOC3L4
WDR20
TTC5
MAPK1IP1L
EGLN3
C14orf126
IFT43
WDR89
C14orf149
AHNAK2
TRIM9
TRMT61A
DHRS1
CMTM5
MIA2
TDRD9
ANKRD9
AK7
IFI27L1
C14orf28
TRAPPC6B
C14orf79
PLD4
ADSSL1
RNASE11
TPPP2

RNASE8
MRPL52
PSMB11
OR4K14
OR4L1
OR11H6
LRR1
KLHDC1
FRMD6
SOCS4
NAA30
GPHB5
NOXRED1
JDP2
ISCA2
ACOT4
TTC8
TC2N
SLC24A4
SLC25A29
DEGS2
KLHL33
RPL10L
LINC00239
LOC145216
RDH12
ADAM21P1
GSC
SERPINA12
PRIMA1
MIPOL1
PPP1R36
SLC38A6
C14orf37
FRMD6-AS1
ABHD12B
LOC145474
PTGR2
FAM161B
C14orf166B
ISM2
CEP128
MDP1
TTC7B
LRFN5
FAM71D
TMEM229B

C14orf49
CLEC14A
FITM1
REM2
TMEM30B
MDGA2
SAMD15
C14orf21
EML5
SPTSSA
MGC23270
ADCY4
C14orf183
RALGAPA1
SFTA3
FBXO33
NUDT14
LINC00521
SERPINA11
IGBP1P1
LOC283547
C14orf182
LOC283553
GPR137C
PROX2
ZDHHC22
TMED8
C14orf178
LOC283585
LOC283587
FAM181A-AS1
SNHG10
C14orf177
SLC25A47
LINC00523
LOC283624
TSSK4
FAM177A1
KIAA0284
C14orf80
DHRS4L2
C14orf55
C14orf39
CCDC85C
ITPK1-AS1
SNORD8
SNORD56B

SERPINA9
LINC00226
LINC00221
RNASE10
VSX2
OR6S1
SLC35F4
COX8C
ASPG
RAB15
FLJ31306
TBPL2
C14orf23
TOMM20L
SERPINA13
C14orf64
RTL1
TMEM179
OR4N2
OR4K2
OR4K13
OR4K17
OR4N5
OR11G2
OR11H4
RNASE9
OR5AU1
HEATR4
FLJ22447
PLEKHD1
LOC400236
LOC400238
DICER1-AS1
C14orf180
POTEG
MIR127
MIR134
MIR136
MIR154
MIR203
MIR208A
MIR299
C14orf165
OR11H12
RNASE13
LINC00238
CCDC88C

OR4Q3
OR4M1
TEX21P
MIR323A
MIR337
MIR345
MIR376C
MIR369
RNASE12
MIR376A1
MIR377
MIR379
MIR380
MIR381
MIR382
MIR433
MIR431
MIR329-1
MIR329-2
MIR323B
MIR409
MIR412
MIR410
MIR376B
MIR485
MIR493
MIR432
MIR494
MIR495
MIR496
MIR487A
ACOT1
ACOT6
POTEM
LOC642426
ECRP
C14orf176
CBLN3
LOC645431
RPL13AP3
LINC00520
FLJ43390
SYNDIG1L
TEX22
MIR539
MIR376A2
MIR487B

SCARNA13
SNORA28
SNORA79
SNORD9
MIR411
MIR624
MIR654
MIR655
MIR656
DHRS4L1
C14orf38
LOC731223
SNORD113-1
SNORD113-2
SNORD113-4
SNORD113-5
SNORD113-6
SNORD113-7
SNORD113-9
SNORD114-1
SNORD114-2
SNORD114-3
SNORD114-4
SNORD114-5
SNORD114-6
SNORD114-7
SNORD114-8
SNORD114-9
SNORD114-10
SNORD114-11
SNORD114-12
SNORD114-13
SNORD114-14
SNORD114-15
SNORD114-16
SNORD114-17
SNORD114-18
SNORD114-19
SNORD114-20
SNORD114-21
SNORD114-22
SNORD114-23
SNORD114-24
SNORD114-25
SNORD114-26
SNORD114-27
SNORD114-28

SNORD114-29
SNORD114-30
SNORD114-31
MIR758
MIR668
MIR770
SNORD127
SNORD126
SNORA11B
MIR300
MIR541
MIR665
MIR543
MIR208B
MIR889
ZBTB42
KTN1-AS1
LOC100129345
LOC100129794
LOC100131366
LOC100288846
LOC100289511
MIR1247
MIR1185-1
MIR1185-2
MIR1260A
MIR1197
OTX2OS1
MIR1193
MIR4309
MIR3173
MIR4308
MIR4307
MIR548Y
LOC100505967
LOC100506071
LOC100506321
LOC100506433
LOC100507043
NEDD8-MDP1
BCL2L2-PABPN1
SYNJ2BP-COX16
CHURC1-FNTB
MIR4505
MIR3545
MIR4708
MIR4709

MIR4503
MIR4710
MIR548AI
MIR4707
MIR4706
MIR2392
LOC100628307
HIF1A-AS2

KIRP			PAAD				PC
ype A	Subtype B		Subtype A		Subtype B		Subtype A	
Del	Amp	Del	Amp	Del	Amp	Del	Amp	Del
hsa-mir-551a		CDKN2A			[GATA6]	CDKN2A	MAML3	NF1
LINC00290		hsa-mir-3133			[PCAT1]	SMAD4	SLC4A1	MAN1A2
hsa-mir-4301		hsa-mir-1976			RPS16	SFN	S100A1	hsa-mir-32
hsa-mir-1910		DDX10			RUSC2	hsa-mir-14	UBTF	RBMS3
CDKN2A		CRIP1			TRRAP	hsa-mir-74	S100A2	hsa-mir-55
hsa-mir-3133		LRP1B			NOTCH2	XRCC4	RUNDC3A	NAALADL2
hsa-mir-637		hsa-mir-1303			ERBB2	ACR	S100A3	hsa-mir-42
hsa-mir-3201		hsa-mir-4273			hsa-mir-12	FOXF2	S100A4	hsa-mir-13
hsa-mir-133b		hsa-mir-1913			BCAT1	ARHGAP8	S100A5	ATP1B2
hsa-mir-3171		hsa-mir-147b			BAG4	TMEM132	S100A6	PTPRD
LRP1B		hsa-mir-548g			SLC47A1	ANG	S100A13	FUT9
hsa-mir-605		hsa-mir-1234			hsa-mir-57	hsa-mir-59	CHTOP	hsa-mir-34
ADARB1		CDKN2B			SLC26A8	hsa-mir-13	S100A14	MMRN1
hsa-mir-4251		hsa-mir-149			PLK1S1	hsa-mir-43	S100A16	hsa-mir-32
hsa-mir-34c		hsa-mir-3115			ZMAT4	SMARCA4		hsa-mir-31
hsa-mir-3182		EXPH5			hsa-mir-14	hsa-mir-302e		hsa-mir-12
CDKN2B		CRIP2			MECOM	CLEC3A		hsa-mir-21
hsa-mir-149		hsa-mir-3141			COL4A4	hsa-mir-147b		OMG
DAPK3		hsa-mir-1324			SUPT5H	ADARB2		FAM19A5
ACR		hsa-mir-1202			UNC13B	hsa-mir-1274b		hsa-mir-42
CRISP1		hsa-mir-1282			ARPC1B	hsa-mir-548x		MIR4789
hsa-mir-4307		hsa-mir-3139			GRB7	AJAP1		hsa-mir-30
LOC647012		hsa-mir-939			CPM	LPAR6		hsa-mir-42
PRKG1		MTAP			LRMP	PARK2		CD68
PTTG1IP		hsa-mir-4269			DDHD2	LIPF		C9orf123
hsa-mir-429		hsa-mir-4253			SNORA59B	hsa-mir-3133		hsa-mir-12
ACAT1		KDELC2			ATP5I	COL15A1		FAM190A
AFG3L1P		ELK2AP			C1R	C9orf53		hsa-mir-13
MTAP		hsa-mir-1294			CLDN11	ARID1A		hsa-mir-31
hsa-mir-4269		hsa-mir-1284			ACSL3	LPO		hsa-mir-54
EEF2		hsa-mir-1273c			ZFP36	MAP2K4		hsa-mir-29
ARSA		hsa-mir-627			ATP8B5P	SCARNA18		MIR4733
GCLC		LRBA			ARPC1A	ARSA		LOC284933
hsa-mir-208b		hsa-mir-661			NEUROD2	FOXC1		hsa-mir-42
CSTF2T		C9orf53			IFNG	PRR5		hsa-mir-48
COL6A1		AGXT			C12orf77	FLJ37505		hsa-mir-54
hsa-mir-1302-2		hsa-mir-1256			SNORA59A	HNRNPC		EFNB3
APOA1		BRF1			CTBP1	hsa-mir-153-2		hsa-mir-42
APRT		hsa-mir-378			C1S	hsa-mir-4276		hsa-mir-31
C9orf53		hsa-mir-3136			PRKCI	hsa-mir-1909		hsa-mir-15
hsa-mir-562		hsa-mir-3145			IRS1	C19orf52		hsa-mir-54
SNORD37		hsa-mir-4310			GMFG	hsa-mir-483		hsa-mir-63
CHKB		CLGN			FAM166B	WWOX		MIR3201

GSTA1	hsa-mir-937	SMURF1	hsa-mir-1282	hsa-mir-13[illegible]
hsa-mir-1201	CDKN2B-AS1	PNMT	KLF6	hsa-mir-67[illegible]
MIR605	KIF1A	LYZ	hsa-mir-935	hsa-mir-19[illegible]
COL6A2	hsa-mir-1290	DGKQ	hsa-mir-125b-2	EIF4A1
CDK11B	JAG2	CACNA1C	MIR4417	hsa-mir-12[illegible]
APOA4	hsa-mir-145	SKIL	RNLS	ADCY7
C16orf3	hsa-mir-4272	PAX3	hsa-mir-149	hsa-mir-11[illegible]
CDKN2B-AS1	hsa-mir-548a-2	PAF1	TGFBR1	hsa-mir-19[illegible]
hsa-mir-1471	hsa-mir-626	MYH16	PIGV	hsa-mir-36[illegible]
PIAS4	EDNRA	TCAP	MPO	MIR4535
CPT1B	hsa-mir-1302-7	MDM2	MIR744	CDK11B
GSTA2	BOK	FGFR3	CHKB	hsa-mir-42[illegible]
ANG	hsa-mir-1273d	CCND2	FOXQ1	hsa-mir-31[illegible]
PRMT2	ADAM6	TERC	PRR5-ARHGAP8	SHBG
DFFB	hsa-mir-584	SERPINE2	MIR3612	hsa-mir-58[illegible]
APOC3	hsa-mir-135a-1	SAMD4B	RNASE1	AGRP
CA5A	hsa-mir-588	TMEM130	hsa-mir-671	ACTA1
hsa-mir-1244-1	hsa-mir-1233-2	STARD3	hsa-mir-548t	hsa-mir-56[illegible]
MIR637	ELF2	CNOT2	hsa-mir-3187	ACACA
TYMP	hsa-mir-151	GAK	hsa-mir-675	DFFB
GSTA3	COL6A3	CD4	hsa-mir-627	hsa-mir-21[illegible]
APEX1	hsa-mir-34a	SEC62	IDI1	AGA
ITGB2	MTA1	SCG2	hsa-mir-373	SOX15
DVL1	hsa-mir-874	MED29	hsa-let-7c	hsa-mir-21[illegible]
ARCN1	hsa-mir-566	KPNA7	ANKRD22	AMFR
CBFA2T3	hsa-mir-3144	IKZF3	hsa-mir-4269	ACTN2
hsa-mir-4268	hsa-mir-1233-1	PTPRB	GALNT12	hsa-mir-56[illegible]
MAPK11	GAB1	IDUA	ZDHHC18	ACCN1
GSTA4	hsa-mir-30d	CD9	SEPT4	DVL1
BCL2L2	DTYMK	GPR160	CPT1B	ADM
LSS	hsa-mir-4252	CUL3	RNASE2	SLC25A4
MEGF6	KIAA0125	PLEKHG2	hsa-mir-1975	TP53
ATM	hsa-mir-886	MIR3609	hsa-mir-1979	hsa-mir-10[illegible]
CDH13	hsa-mir-4271	PPP1R1B	hsa-mir-1302-11	BBS2
hsa-mir-3132	hsa-mir-548b	RAP1B	hsa-mir-4298	ADSS
MAPK12	hsa-mir-211	LETM1	hsa-mir-4310	hsa-mir-42[illegible]
HCRTR2	GYPA	CD27	PFKP	ACLY
CEBPE	hsa-mir-1208	MYNN	hsa-mir-1283-2	MEGF6
PCNT	GBX2	FARSB	hsa-mir-3118-5	AP2A2
GABRD	hsa-mir-551a	MIR4530	LIPJ	ANXA2P1
FXYD2	PACS2	MIEN1	hsa-mir-562	FXR2
CDH15	hsa-mir-1289-2	YEATS4	ALG2	hsa-mir-14[illegible]
hsa-mir-153-1	hsa-mir-191	MYL5	SRSF1	CA7
SBF1	hsa-mir-587	CHD4	TYMP	AGT
IL17A	hsa-mir-1268	SLC7A14	RNASE3	hsa-mir-13[illegible]
CMA1	GYPB	KCNE4	hsa-mir-548f-4	AP2B1
S100B	hsa-mir-1207	PGAP3	AGA	GABRD

GNB1	GPC1	CCT2	AMH	AMPD3
CXCR5	hsa-mir-4251	PDE6B	hsa-mir-210	CASP3
COX4I1	GPR132	ATN1	hsa-mir-626	MPDU1
hsa-mir-3131	hsa-mir-1244-2	LRRC31	PITRM1	hsa-mir-31[illegible]
PPP6R2	hsa-mir-711	DOCK10	hsa-mir-643	CBFB
MCM3	hsa-mir-2113	MIR4728	hsa-mir-3156-3	ARF1
LTB4R	hsa-mir-3118-6	FRS2	LIPM	hsa-mir-12[illegible]
SLC19A1	GYPE	WHSC1	hsa-mir-1471	AOC2
PEX10	hsa-mir-1205	ENO2	ANKS6	GNB1
CASP1	GPR35	PHC3	SUPT4H1	APBB1
CYBA	hsa-mir-429	WDFY1	MAPK11	CLCN3
hsa-mir-26b	TMEM121	CPSF6	RNASE4	SNORA67
ZBED4	hsa-mir-548f-3	ZNF141	hsa-mir-490	hsa-mir-13[illegible]
MUT	hsa-mir-2115	FGF6	SLC25A4	CBLN1
CTSG	hsa-mir-4282	ARPM1	ATP5D	CHML
MCM3AP	hsa-mir-3118-4	NYAP2	AP2A2	hsa-mir-31[illegible]
PRKCZ	IL15	KCNMB4	hsa-mir-1233-2	ARL4D
CASP4	hsa-mir-1204	SLBP	ZMYND11	PEX10
DPEP1	HDLBP	FKBP4	hsa-mir-125a	RHOG
hsa-mir-548f-2	hsa-mir-1302-2	LRRC34	BAGE	CPE
SCO2	BTBD6	MRPL44	LIPK	WRAP53
PGK2	hsa-mir-548p	IL22	hsa-mir-1244-1	hsa-mir-31[illegible]
DAD1	hsa-mir-1226	MAEA	VEZF1	CDH5
FTCD	hsa-mir-30a	FOXM1	MAPK12	LYST
SCNN1D	ACTC1	LRRIQ4	RNASE6	hsa-mir-42[illegible]
CASP5	SMAD1	FAM124B	hsa-mir-29b-1	ATP6V0A1
FANCA	hsa-mir-548d-1	SLC35E3	CASP3	PRKCZ
hsa-mir-1302-4	NDUFA10	PCGF3	AZU1	ART1
RABL2B	ALPL	GAPDH	APBB1	CTSO
PKHD1	NUDT14	SAMD7	hsa-mir-1233-1	SAT2
FOXG1	hsa-mir-583	RHBDD1	WDR37	hsa-mir-12[illegible]
DIP2A	hsa-mir-564	IL26	hsa-mir-150	CDH8
SKI	hsa-mir-30c-2	SPON2	CXADR	CHRM3
CD3D	APBA2	GNB3	LIPN	hsa-mir-13[illegible]
FOXF1	MGST2	LOC10012[illegible]	hsa-mir-4268	BRCA1
hsa-mir-2355	hsa-mir-2053	MOGAT1	EPX	SCNN1D
MLC1	SEPT2	MDM1	SBF1	ASCL2
RHAG	RERE	TACC3	SALL2	DCTD
GZMH	C14orf80	KCNA1	hsa-mir-335	SNORA48
POFUT2	ADRB2	AP1S3	CLCN3	hsa-mir-37[illegible]
TP73	hsa-mir-138-1	NUP107	HCN2	CDH11
CD3E	hsa-mir-548u	CPLX1	RHOG	FH
FOXL1	B2M	KCNA5	hsa-mir-211	ACY1
hsa-mir-3130-4	NR3C2	SGPP2	DIP2C	CACNB1
MAPK8IP2	hsa-mir-548a-3	RAB3IP	hsa-mir-4324	SKI
TFAP2B	PDCD1	SLC26A1	NCAM2	CARS
GZMB	C1QA	KCNA6	hsa-mir-3132	ETFDH

PCBP3	LINC00226	CCDC140	MTMR4	SNORD10
TNFRSF4	ANXA6	BEST3	PPP6R2	hsa-mir-145
CD3G	ACVR2B	FGFRL1	TOX4	CDH16
FOXC2	hsa-mir-133b	LAG3	hsa-mir-183	GALNT2
AAMP	NBEAP1	LOC646736	CPE	ADCY5
PLXNB2	NDUFC1	LRRC10	BSG	CDC6
CRISP2	hsa-mir-3151	PIGG	ART1	TP73
HNRNPC	PPP1R7	LTBR	hsa-mir-1268	CCKBR
C21orf58	C1QB	MIR4439	LARP4B	F11
MMP23B	LINC00221	MIR1279	hsa-mir-220c	hsa-mir-584
CRYAB	APC	KIAA1530	TMPRSS15	CES1
GALNS	ACY1	NDUFA9	hsa-mir-153-1	GNG4
ACADL	hsa-mir-586	SNORA70G	BZRAP1	ADPRH
BRD1	BUB1B	ABCA11P	ZBED4	CCR7
GCM1	POU4F2	NINJ2	EDDM3A	TNFRSF4
MMP14	hsa-mir-1273	MIR3913-2	hsa-mir-129-1	CD81
YBEY	SPP2	MFSD7	DCTD	ACSL1
MMP23A	C1QC	NOP2	CDC34	hsa-mir-874
DDX6	TEX22	MIR3913-1	ASCL2	CETP
GAS8	ATOX1	TMEM175	hsa-mir-3118-6	GUK1
AGXT	ALAS1	NTF3	GTPBP4	ALAS1
FAM19A5	hsa-mir-1275	LOC100507	hsa-mir-3191	CNP
TRAM2	CAPN3	C4orf42	HSPA13	MMP23B
MYH6	ABCE1	PTMS	hsa-mir-3131	CD151
COL18A1	hsa-mir-875	TMEM129	OR4D1	FAT1
TNFRSF14	PER2	PTPN6	SCO2	hsa-mir-886
DDX10	CA6	ZNF595	SUPT16H	CNGB1
GCSH	ALDH7A1	PEX5	hsa-mir-593	HNRNPU
ALPI	AMT	FAM53A	ETFDH	ALCAM
NCAPH2	hsa-mir-219-1	RAD52	CIRBP	CSF3
CRISP3	CHRM5	ZNF721	CARS	MMP23A
MYH7	RPS3A	KDM5A	hsa-mir-3118-4	CDKN1C
C21orf56	hsa-mir-3150	ZNF718	IDI2-AS1	FGA
TNFRSF18	LRRFIP1	SCNN1A	hsa-mir-320e	hsa-mir-128
DLAT	CAPZB	CRIPAK	TPTE	CSNK2A2
HSBP1	CAMK4	SLC6A12	hsa-mir-26b	KCNK1
ALPP	APEH	RNF212	RNF43	ARF4
MOV10L1	hsa-mir-1236	SLC6A13	RABL2B	DHX8
ICK	CHRNA7	ZNF876P	OR10G3	TNFRSF14
NEDD8	UCP1	VAMP1	hsa-mir-592	TPP1
C21orf67	hsa-mir-3149	ZNF732	F11	FGB
ISG15	HDAC4	TEAD4	CNN2	hsa-mir-124
DRD2	CASP9	LOC100129	CCKBR	CTRL
HSD17B2	CAMK2A	TNFRSF1A	ACTC1	LGALS8
ALPPL2	ARF4	LOC100130	IDI2	ATP6V1A
MIOX	hsa-mir-877	TPI1	A1BG	DUSP3
CD2AP	CKMT1B	TMED11P	NRIP1	TNFRSF18

NFATC4	SMARCA5	TULP3	hsa-mir-548f-2	CNGA4
FAM207A	hsa-mir-2052	VWF	MKS1	FGG
PLCH2	FARP2	FGF23	MLC1	hsa-mir-548
FDX1	RUNX3	USP5	OR10G2	CYLD
IRF8	CAMLG	MLF2	ABP1	MTR
ATIC	RHOA	DYRK4	ACSL1	C3orf51
PANX2	hsa-mir-3143	CD163	CSNK1G2	ERBB2
FBXO9	DUT	CLSTN3	CD81	ISG15
NOVA1	INPP4B	NCAPD2	APBA2	CTSD
MCM3AP-AS1	hsa-mir-124-2	LPCAT3	LOC282980	FRG1
CEP104	ARL4C	LRRC23	AP2A1	hsa-mir-548
GRIA4	TNFRSF8	EMG1	BTG3	DYNC1LI2
MAF	CAST	LEPREL2	hsa-mir-1302-4	NID1
KIF1A	C3orf51	AKAP3	MSX2P1	CACNA1D
ALG12	hsa-mir-548a-1	RAD51AP1	MAPK8IP2	ETV4
TNFRSF21	EPB42	TSPAN9	OR4E2	PLCH2
PNP	SNORD73A	PHB2	AKR1B1	DRD4
LINC00315	hsa-mir-486	ERC1	FAT1	GK3P
SLC35E2	RAMP1	IFFO1	CFD	hsa-mir-58[illegible]
GUCY1A2	CDA	GALNT8	CD151	E2F4
MC1R	CD14	GPR162	B2M	RAB4A
BARD1	CACNA1D	COPS7A	LOC338588	CASR
CRELD2	ABCF1	ING4	KLK3	EZH1
TINAG	FBN1	MRPL51	USP25	CEP104
NRL	ANAPC10	C1RL	hsa-mir-2355	DUSP8
LINC00162	hsa-mir-3148	TAPBPL	HSF5	GLRB
RER1	STK25	PLEKHG6	PLXNB2	hsa-mir-22[illegible]
HSPB2	CDK11B	ITFG2	SLC39A2	GNAO1
MVD	CD74	PRMT8	ARF5	RGS7
BCS1L	SLC25A20	PARP11	FRG1	CBLB
ADM2	ACAT2	ANO2	ARID3A	GAST
TMEM14A	GABRA5	C12orf4	CDKN1C	SLC35E2
OXA1L	MAB21L2	C12orf5	NBEAP1	EIF4G2
COL18A1-AS1	hsa-mir-4288	LPAR5	TUBB8	GPM6A
NOC2L	COPS8	WNK1	BAX	hsa-mir-9-2
HTR3A	CDC42	ADIPOR2	RBM11	GOT2
CHMP1A	CDC25C	WNT5B	hsa-mir-3130-4	RYR2
BMPR2	CAMP	CDCA3	OR4D2	CD80
TRABD	CRISP1	C12orf32	BRD1	FZD2
HMGCLL1	GABRB3	NRIP2	ZNF219	RER1
PCK2	LSM6	RBP5	OPN1SW	HBB
LOC642852	hsa-mir-4287	CCDC77	GK3P	GRIA2
OR4F3	CAPN10	ACRBP	EFNA2	hsa-mir-428
IL10RA	CD52	SPSB2	TPP1	HSD11B2
PLCG2	CDO1	EFCAB4B	BUB1B	TARBP1
BOK	CCK	CACNA2D4	LINC00200	CD86
SELO	AGER	C12orf57	BCAT2	G6PC

CENPQ	GABRG3	FBXL14	CHODL-AS1	NOC2L
PSMB5	TBC1D9	ZNF384	AAMP	HBBP1
LINC00163	hsa-mir-548h-4	DSTNP2	DYNLL2	GUCY1A3
ARHGEF16	PASK	C12orf53	NCAPH2	hsa-mir-58
IL18	RCC1	DCP1B	ARHGEF40	HSF4
RPL13	CDX1	MATL2963	BPGM	TBCE
CASP8	ENTPD3	CD163L1	GLRB	CD47
HDAC10	AIF1	RPL13P5	ELANE	KAT2A
LRRC1	GANC	B4GALNT3	CNGA4	OR4F3
PSME1	SLC7A11	LOC28344	CAPN3	HBD
SSR4P1	hsa-mir-320a	ACSM4	LOC399708	GUCY1B3
SSU72	ATG4B	MIR141	C5AR1	hsa-mir-44
MLL	CLCN6	MIR200C	C21orf15	KIFC3
SPG7	CHD1	IQSEC3	ACADL	GPR137B
CASP10	CDC25A	LOC57453	C17orf47	CISH
SHANK3	AIM1	LRTM2	MOV10L1	GFAP
ELOVL5	GATM	SCARNA12	METTL3	ARHGEF16
PSME2	CCRN4L	SCARNA11	BRAF	HBE1
LOC100129027	hsa-mir-548v	FAM138D	GPM6A	HMGB2
WRAP73	SH3BP4	LOC67865	GAMT	hsa-mir-58
NCAM1	CLCNKA	SCARNA10	CTSD	LCAT
GAN	AP3S1	LOC10027	CHRM5	TSNAX
CD28	CISH	LOC10028	C10orf108	COL8A1
TUBGCP6	AMD1	LOC10029	CA11	CCR10
TFAP2D	GCHFR	MIR3649	LINC00308	SSU72
RABGGTA	ZNF330	LOC10050	AGXT	HBG1
LOC100505746	hsa-mir-383	LOC10065	MIR142	HPGD
SDF4	SNED1		MIOX	ADRA1B
NNMT	CLCNKB		RPGRIP1	MMP2
SLC7A5	CSF1R		CALD1	ZNF124
CHRND	CCR1		GRIA2	CPOX
LOC90834	ARG1		GNG7	GRB7
PAQR8	PDIA3		DRD4	WRAP73
RNASE1	PCDH18		CHRNA7	HBG2
MXRA8	hsa-mir-598		ADARB2-AS1	HSP90AA4
NPAT	TRAF3IP1		CALM3	ADRB2
CDK10	CNR2		C21orf91	MMP15
CHRNG	CSF2		ALPI	HIST3H3
LMF2	CCR3		LOC100506779	CSTA
MLIP	ATP6V1G2		PANX2	GRN
RNASE2	IPW		NDRG2	SDF4
CPSF3L	OTUD4		CALU	HPX
PAFAH1B2	hsa-mir-1322		HMGB2	ING2
MBTPS1	ANO7		MKNK2	ANXA6
COL4A3	CORT		DUSP8	MT1A
KLHDC7B	CSNK1A1		CKMT1B	GNPAT
IL17F	CCR5		LOC100216001	DNASE1L3

RNASE3	BAI3	CD33	HCRT
C1orf159	ITPKA	SAMSN1	MXRA8
POU2AF1	MAML3	ALPP	HRAS
TAF1C	hsa-mir-4286	MIR4736	IRF2
COL4A4	PRLH	ALG12	APC
LOC284933	DDOST	CHD8	MT1B
EFHC1	CSNK1G3	CASP2	KMO
RNASE4	CCR8	HPGD	DOCK3
AURKAIP1	BAK1	GPX4	HSD17B1
PPP2R1B	IVD	HBB	CPSF3L
USP10	TMEM184C	EPB42	IGF2
COL6A3	hsa-mir-124-1	LOC100507034	KLKB1
RPL23AP82	THAP4	SIGLEC6	TRIM23
C6orf141	DFFA	BAGE5	MT1E
RNASE6	CTNNA1	ALPPL2	EXO1
MRPL20	CCBP2	CRELD2	DRD3
PTS	BCKDHB	EDDM3B	IFI35
C16orf7	LTK	CDK5	C1orf159
CPS1	RNF150	HSP90AA4P	ILK
C22orf34	hsa-mir-597	GZMM	MTNR1A
GSTA5	ANKMY1	HBBP1	ARSB
SALL2	DFFB	GABRA5	MT1F
ATAD3A	DMXL1	CD37	GGPS1
RDX	COL7A1	BAGE4	DUSP7
KIAA0513	CFB	ATIC	IGFBP4
CREB1	MAP1A	ADM2	AURKAIP1
CHKB-CPT1B	PCDH10	METTL17	INS
OPN5	hsa-mir-548i-3	CHRM2	NEK1
TEP1	SCLY	ING2	ATOX1
PANK4	DVL1	GADD45B	MT1G
SCN2B	DIAPH1	HBD	TOMM20
PIEZO1	CTNNB1	GABRB3	EPHA3
CRYBA2	PRDM1	CGB	ITGA2B
IL17REL	MEIS2	BAGE3	MRPL20
GPR115	SCOC	KIF1A	IRF7
TGM1	hsa-mir-596	TRABD	NPY1R
TP73-AS1	ASB1	RNASE7	ALDH7A1
SCN4B	E2F2	CLCN1	MT1H
ATP2C2	DPYSL3	IRF2	URB2
CRYGA	CX3CR1	OAZ1	FHIT
FAM116B	BMP5	HBE1	JUP
FAM83B	MFAP1	GABRG3	ATAD3A
PABPN1	HHIP	AP2S1	KCNQ1
LRRC47	NAT1	BAGE2	NPY2R
SDHD	UGT1A10	BARD1	BHMT
CLEC3A	ECE1	SELO	MT1JP
CRYGB	SLC26A2	RAB2B	CEP170

PIM3	CYP8B1	CPA1	FLNB
GPR111	BMP6	KLKB1	KRT9
AP1G2	TRPM1	PALM	PANK4
HES4	ARHGAP10	HBG1	LMO1
SLN	NAT2	GANC	NPY5R
MPHOSPH6	UGT1A8	CRX	BNIP1
CRYGC	MEGF6	CHODL	MT1M
ODF3B	HBEGF	BCS1L	AKT3
DEFB110	DAG1	HDAC10	GAP43
SLC7A7	DST	TPPP2	KRT10
PRDM16	NDN	CPA2	AJAP1
TAGLN	NAA15	MTNR1A	LSP1
COX4NB	ADCY8	POLR2E	PET112
CRYGD	UGT1A7	HBG2	BTF3
SYCE3	EPHA2	GATM	MT1L
DEFB112	EBF1	DBP	ZNF238
CPNE6	DNASE1L3	ANKRD30BP2	GATA2
VWA1	BTN1A1	BMPR2	KRT12
UPK2	OCA2	SHANK3	TP73-AS1
TUBB3	SETD7	RNASE8	MUC2
CTLA4	ADRA1A	DPP6	PLRG1
LOC100128946	UGT1A6	NEK1	CAMK4
DEFB113	ENO1	POLRMT	MT1X
TOX4	EFNA5	HPX	SPHAR
NADK	DOCK3	GCHFR	GBE1
ZBTB16	BYSL	EMP3	KRT13
PRDM7	PLCB2	LIPI	LRRC47
CYP27A1	RAB33B	BOK	MUC6
LOC100144603	ADRB3	TUBGCP6	PPID
DEFB114	UGT1A5	LOC283624	CAMK2A
REC8	EPB41	EN2	MT2A
MMEL1	EGR1	NPY1R	CAPN9
CUL5	DUSP7	PRTN3	GOLGB1
MON1B	C2	HRAS	KRT14
DES	RAD51	PDIA3	HES4
MIR3201	TTC29	ETFB	NAP1L4
GLYATL3	ANGPT1	ABCC13	RPS3A
PARP2	UGT1A9	CD28	CAMLG
OR4F5	EPHA8	LOC90834	MT3
ZNF259	ETF1	SNORD8	SDCCAG8
TCF25	CELSR3	EPHA1	GP9
DTYMK	C4A	NPY5R	KRT15
MIR4535	RYR3	PTBP1	PRDM16
GFRAL	SLC10A7	IGF2	NUP98
DHRS2	ANGPT2	IPW	MSMO1
LINC00115	UGT1A4	FCAR	CANX
PCSK7	EPHB2	C21orf91-OT1	NFATC3

ZCCHC14	FAT2	CHRND	COG2
EEF1B2	FHIT	LMF2	GPR15
KLHL31	C4B	OR6S1	KRT16
EFS	SCG5	EPHB6	VWA1
MORN1	USP38	PPID	SLC22A18
HTR3B	ANK1	RPS15	SFRP2
KIAA0182	UGT1A1	ILK	CAST
EPHA4	EXTL1	ITPKA	POLR2C
DEFB133	FBN2	FCGRT	RBM34
IRF9	FLNB	D21S2088E	GPR27
GLTPD1	DDR1	CHRNG	KRT17
ZW10	SLC12A1	KLHDC7B	NADK
ATMIN	C4orf49	OR5AU1	SLC22A18A
ERBB4	ANXA13	EZH2	TDO2
MIR206	UGT1A3	MSMO1	CCNB1
PRMT5	EYA3	SGTA	PSKH1
OR4F16	FER	INS	ABCB10
UBE4A	GNAI2	IVD	GRM2
COTL1	RUNX2	FKBP1AP1	KRT19
ACSL3	SNRPN	POTED	MMEL1
PTCHD4	PRMT10	COL4A3	POLR2L
TM9SF1	ASAH1	RPL23AP82	TLL1
CCNL2	USP40	RNASE13	CCNG1
RBM7	FGR	FLNC	PSMB10
MLYCD	FGF1	TLL1	OPN3
FN1	GNAT1	STK11	GSK3B
MIR133B	CCNC	IRF7	KRT31
EDDM3A	SORD	LTK	OR4F5
TAS1R3	PABPC4L	FLT3LG	PSMD13
MPZL2	ASPH	LINC00317	TLR2
CPNE7	HJURP	COL4A4	CCNH
GBX2	MTOR	C22orf34	RBL2
RPS16P5	GDF9	ECRP	TRIM58
DHRS4	XCR1	GBX1	GTF2E1
ATAD3B	CCND3	TLR3	KRT32
ATP5L	SPINT1	TCF3	LINC00115
IL17C	ZNF827	KCNQ1	RNH1
GPC1	ATP6V1B2	MAP1A	TLR3
LOC730101	HES6	FPR1	CD14
RIPK3	FUCA1	LINC00320	RRAD
PLEKHN1	GFRA3	COL6A3	AHCTF1
TREH	GPR27	CHKB-CPT1B	HCLS1
ANKRD11	CDC5L	C14orf176	KRT33A
GPR1	SRP14	GRM8	MORN1
GSTA7P	DCLK2	VEGFC	MRPL23
SUPT16H	ATP6V1C1	THOP1	VEGFC
C1orf170	CXCR7	LMO1	CD74

CEP164	IFI6	MEIS2	SALL1
OSGIN1	GLRA1	FPR2	OR1C1
GPR35	GPX1	LOC388813	HGD
ACIN1	CDKN1A	CPS1	KRT33B
KIAA1751	THBS1	IL17REL	GLTPD1
EXPH5	FREM3	SNORD9	RPL27A
GINS2	BAI1	MNX1	GLRA3
HDLBP	RNPEPL1	GLRA3	CDC25C
KHNYN	GABRD	MADCAM1	CCL17
LOC115110	GLRX	LSP1	OR2M4
PHLDB1	GRM2	MFAP1	HTR1F
TRAPPC2L	CDSN	FPR3	KRT34
AGFG1	TJP1	LINC00478	OR4F16
SLC7A8	MMAA	CREB1	RPLP2
ACAP3	BLK	FAM116B	SORBS2
SIK2	GAL3ST2	HTR5A	CDK7
WWOX	GALE	SORBS2	CCL22
DNAJB2	GM2A	SF3A2	OR2L2
NGDN	HYAL1	MUC2	ITGB5
UBE2J2	CGA	TRPM1	KRT35
SIK3	TP53BP1	FTL	CCNL2
BCMO1	ELMOD2	ANKRD20A11P	RRM1
HTR2B	BMP1	CRYBA2	SAP30
LRP10	RAB17	PIM3	CDO1
PUSL1	GNB1	IMPDH1	CX3CL1
BACE1	GPX3	SAP30	OR2L1P
NECAB2	IMPDH2	PPAP2C	ITIH1
IDH1	CLIC1	MUC6	LASP1
TINF2	TYRO3	NDN	TAS1R3
B3GALT6	GUSBP5	FUT1	SCT
CADM1	POLR3D	MIRLET7C	SNORD73A
KLHDC4	TRPM8	CRYGA	CDX1
SP110	SFN	ODF3B	SLC6A2
OR10G3	GRIA1	INSIG1	OR2T1
TPRG1L	ITIH1	HAND2	ITIH3
REXO2	CLPS	AP3D1	LHX1
DEF8	UBE3A	NAP1L4	ATAD3B
IGFBP2	LOC641364	OCA2	SMPD1
OR10G2	BNIP3L	FUT2	LRAT
FAM213B	MLPH	MIR125B2	CETN3
TIMM8B	GPR3	CRYGB	SLC9A5
BANP	NR3C1	SYCE3	TAF5L
IGFBP5	ITIH3	IRF5	ITIH4
OR4E2	CCR6	RAPGEF2	LIG3
ACTRT2	MKRN3	MED16	PLEKHN1
SIDT2	LOC641365	NUP98	TRIM21
ZDHHC7	OSGIN2	PLCB2	HAND2

IHH	IQCA1	GPR32	CHD1
CIDEB	ZBTB48	MIR99A	SLC12A3
MIB2	HARS	CRYGC	DISC2
FXYD6	ITIH4	LOC100128946	KPNA1
CENPN	CNR1	KCNH2	NBR1
CXCR1	SLC30A4	MFAP3L	C1orf170
CHMP4A	LOC646576	FSTL3	ST5
SAMD11	CA1	SLC22A18	RAPGEF2
C11orf71	C2orf54	RAD51	CKMT2
C16orf61	HMGN2	GRIN2D	SLC12A4
CXCR2	HINT1	TEKT4P2	DISC1
STXBP6	LAMB2	CRYGD	LSAMP
LOC148413	COL9A1	LOC100144603	ADAM11
SLC35F2	PAR5	KEL	KIAA1751
JPH3	C4orf51	ADAM29	STIM1
CXCR2P1	CA2	APC2	MFAP3L
SLC39A2	ILKAP	SLC22A18AS	ERCC8
CCDC27	HMGCL	RYR3	TK2
RAB39A	HSD17B4	ARHGAP35	PPPDE1
VAT1L	RPSA	MIR3156-3	MCM2
INHA	COL10A1	CTLA4	MEOX1
FAM158A	EIF3J	MIR4535	LOC11511C
CALML6	LOC100129858	LEP	TAF10
BTG4	CA3	ANXA10	ADAM29
KIAA1609	ING5	ABCA7	AP3S1
INPP5D	HSPG2	POLR2L	TRADD
ZNF219	HSPA4	SCG5	SCCPDH
C1orf86	LTF	GYS1	MITF
FAM55D	COL11A2	MIR3687	MLLT6
WFDC1	JMJD7-PLA2G4B	CYP27A1	ACAP3
IRS1	LOC100505545	MEST	TALDO1
GMPR2	CA8	KLHL2	ANXA10
ATAD3C	MGC16025	UQCR11	CLTB
TTC12	HTR1D	PSMD13	CES2
MTHFSD	HSPA9	SNRPN	TRIM17
KCNJ13	MAP4	HAS1	CD200
SLC22A17	COL12A1	MIR3648	MPP2
LOC254099	SNAP23	DES	UBE2J2
C11orf57	MIR4799	MKLN1	TH
DBNDD1	CALB1	SCRG1	KLHL2
MAP2	AGAP1	SBNO2	COX7C
HAUS4	HTR6	RNH1	NAE1
TTLL10	NDST1	SORD	ARID4B
ELMOD1	MITF	HRC	MYLK
KLHL36	COL19A1	C21orf37	MPP3
MYL1	HERC2	DTYMK	PUSL1
C14orf119	RUNX1T1	NDUFB2	TSPAN4

FAM41C	TWIST2	DUX4	SCRG1
TEX12	ID3	HMHA1	CRHBP
FBXO31	HTR4	MRPL23	NOL3
NCL	MOBP	SPINT1	EGLN1
RNF31	COX7A2	PRMT1	NDUFB4
C1orf174	SNURF	EEF1B2	MYO1D
TMPRSS4	CDH17	NOS3	B3GALT6
CMIP	NEU4	PALLD	TNNI2
NDUFA10	TNFRSF9	SHC2	DUX4
RBM23	IK	RPL27A	HAPLN1
KLHL17	MST1	SRP14	SLC7A6
IFT46	ATF6B	IL11	KIAA1383
CDT1	SLC28A2	EPHA4	CNTN3
NDUFS1	CEBPD	CNOT4	NAGLU
C14orf167	MTERFD2	ANP32C	TPRG1L
TMEM240	STMN1	TIMM13	TNNT3
DSCAML1	IL3	RPLP2	PALLD
MAP1LC3B	MST1R	THBS1	CSF1R
SEPT2	MAPK14	IRF3	ATP6V0D1
OSGEP	TGM5	ERBB4	KIF26B
TMEM52	CHRNA2	NRF1	PDHB
ARHGAP20	UBE2F	FAM149A	NEUROD2
DYNLRB2	MATN1	DAZAP1	FAM213B
NEU2	IL4	RRM1	TRPC2
ARHGEF40	MYD88	TJP1	KIAA0922
AGRN	CSNK2B	KCNA7	CSF2
USP28	PPIP5K1	ACSL3	GPR56
HSDL1	CHRNB3	PAX4	HEATR1
NPPC	OTOS	FBXO8	PLXNA1
METTL3	MFAP2	FGF22	PEX12
FAM132A	IL5	SCT	ACTRT2
CARD18	MYL3	TP53BP1	PHLDA2
CRISPLD2	CTGF	KCNC3	TRIM2
PAX3	AQR	FN1	CSNK1A1
LTB4R2	CLU	PIP	N4BP1
HES5	MYEOV2	DUX2	ZNF692
AASDHPPT	MTHFR	SLC39A3	POU1F1
SPIRE2	IL9	SMPD1	PNMT
PDCD1	NKTR	TYRO3	MIB2
SDR39U1	CYP21A2	KCNJ14	TUB
LOC388588	ARHGAP11A	GBX2	ANP32C
C11orf1	COX6C	PODXL	CSNK1G3
ZNF469	OR6B3	PDLIM3	NUP93
PDE6D	NBL1	THEG	NUP133
RPGRIP1	IL13	TRIM21	PRKCD
RNF223	CNTN3	UBE3A	MED1
DYNC2H1	CYP21A1P	KIR2DL1	SAMD11

CNTNAP4	LCMT2	GPC1	WEE1
SERPINE2	CRH	PRSS1	FAM149A
NDRG2	LOC150935	SPOCK3	VCAN
MIR200A	NPPA	LSM7	HERPUD1
ALG9	IRF1	ST5	ERO1LB
CENPBD1	PDHB	MKRN3	PROS1
PPP1R7	DAXX	KIR2DL3	PPY
NYNRIN	SLC12A6	GPR1	LOC148413
MIR200B	CTSB	PRSS2	ZNF143
PDGFD	LOC151171	AADAT	FBXO8
ZNF276	NPPB	MBD3	NKX2-5
PSMD1	ITK	STIM1	NUTF2
HOMEZ	PFKFB4	SLC30A4	FMN2
FLJ42875	DNAH8	KIR2DL4	PTPRG
TMPRSS5	RASGRP1	GPR35	PSMB3
KCNG4	CYC1	TAS2R38	CCDC27
PTH2R	LOC151174	FAM198B	ZNF195
CHD8	OPRD1	MIER2	DUX2
ANKRD65	KCNN2	TAF10	CTNNA1
BCO2	PLXNB1	PAR5	IRX5
SDR42E1	DOM3Z	KIR2DS4	ZNF695
PTMA	SERF2	HDLBP	RHO
CCNB1IP1	CYP7A1	PTN	PYY
MIR429	MSL3P1	GALNT7	CALML6
TMPRSS13	PAFAH2	PCSK4	ZNF214
PKD1L2	LECT2	TALDO1	ARFIP1
PTPRN	PRKAR2A	EIF3J	DBN1
C14orf93	DSP	KIR3DL1	KATNB1
FAM138F	GPR176	AGFG1	GJC2
KIAA1826	CYP11B1	PTPRN2	ROBO1
RNF166	LOC200772	CLDN22	PSMD3
SNORD20	PAX7	C19orf24	C1orf86
ABHD4	LMNB1	TH	ZNF215
LOC643837	PRKCD	JMJD7-PLA2G4B	PDLIM3
BUD13	E2F3	KIR3DL2	DMXL1
C16orf46	CHP	DNAJB2	CTCF
RPE	CYP11B2	RARRES2	SIPA1L2
EDDM3B	CXXC11	C4orf27	ROBO2
TMEM88B	PEX10	PLEKHJ1	PSMD11
TMEM25	LNPEP	TSPAN4	ATAD3C
DNAAF1	PTH1R	SNAP23	RASSF7
RPL37A	EDN1	KLK1	SPOCK3
CDH24	OIP5	HTR2B	DHFR
C1orf233	ADAM3A	RHEB	NUDT21
DIXDC1	DUSP28	MARCH1	ZP4
SPATA2L	PEX14	BTBD2	RPL24
SAG	LOX	TNNI2	RAB5C

METTL17	PTPRG	HERC2	LOC254099
FAM138A	EEF1A1	KLK2	PPFIBP2
ZC3H12C	BAHD1	IDH1	AADAT
C16orf55	DECR1	SHH	DIAPH1
CCL20	ESPNL	NEIL3	CNOT1
IL25	PGD	RNF126	RHOU
WASH7P	SMAD5	TNNT3	RPL29
ALKBH8	QARS	SNURF	RAD51D
ZC3H18	SLC29A1	LAIR1	TTLL10
SLC4A3	FAN1	SP110	IFITM1
THTPA	DEFA1	SLC4A2	FAM198B
MIR551A	RBM44	TMEM144	DOCK2
FDXACB1	PIK3CD	SPPL2B	ZNF423
CDYL2	MAN2A1	TRPC2	TFB2M
SLC11A1	SNORA62	SLC28A2	RPN1
OR4K5	EPB41L2	LAIR2	RARA
CDK11A	MAPKBP1	IGFBP2	FAM41C
C11orf52	DEFA3	SMARCD3	OR6A2
SLC38A8	AQP12A	C4orf43	ACCN5
SP100	PLA2G2A	WDR18	DPYSL3
OR11H2	MCC	PHLDA2	RPGRIP1L
SLC35E2B	ROBO1	TGM5	GREM2
CARD16	EPHA7	LHB	ATXN7
SLC22A31	GOLGA8A	IGFBP5	RPL19
SPP2	DEFA4	SMO	LOC284661
OR4K1	KLHL30	UFSP2	DCHS1
LOC728716	PLA2G5	REXO1	GALNT7
APOA5	MFAP3	TUB	DRD1
FLJ30679	ROBO2	PPIP5K1	CES3
TNP1	EYA4	LIG1	SMYD3
IPO4	RTF1	IHH	SLC15A2
LOC729737	DEFA5	AKR1D1	RPL27
C11orf93	OR6B2	DDX60	C1orf174
LOC146513	PLOD1	LPPR3	EIF3F
TNS1	NDUFA2	ZNF195	CLDN22
DCAF11	RPL29	AQR	SLC26A2
OR4F29	ESR1	LIM2	ARL2BP
PIH1D2	CYFIP1	CXCR1	ARV1
ZFPM1	DEFA6	SSBP1	NEK4
TUBA4A	ASB18	CDKN2AIP	CCL1
OR4K15	EXOSC10	ZNF556	KLHL17
LOC100129534	NEUROG1	ZNF214	BRSK2
FAM55A	ATXN7	ARHGAP11A	DKFZP434I[illegible]
ADAD2	F13A1	MYBPC2	HBEGF
SUMO1	MGA	CXCR2	EDC4
JPH4	DEFB1	TBXAS1	C1orf35
LOC100130417	FLJ43879	ODZ3	TKT

FAM55B	PPP1R8	OR4F17	CCL2
ADAMTS18	NPY6R	ZNF215	TMEM240
VIL1	SCN5A	LCMT2	CTR9
RNASE7	FABP7	NDUFA3	DCHS2
LOC100132062	VPS39	CXCR2P1	DUSP1
AMICA1	DEFB4A	UBE2H	PLA2G15
MGC23284	MIR149	LRP2BP	TTC13
WNT6	PRKCZ	FAM108A1	TMF1
RAB2B	PAM	RASSF7	CCL3
LOC100132287	SCN10A	SLC12A6	TMEM52
KBTBD3	FANCE	NKG7	TRIM66
LINC00311	FAM189A1	INHA	C4orf27
XRCC5	DPYS	VIPR2	EBF1
AJUBA	DNAJB3	PDGFC	PLEKHG4
LOC100133331	PTAFR	KLF16	PGBD5
CWF19L2	PCDH1	PPFIBP2	TNNC1
ZNF778	SEMA3F	RASGRP1	CCL3L1
ZNF142	FKBP5	CNOT3	AGRN
ZFHX2	CCNDBP1	INPP5D	TSPAN32
LOC100133445	DPYSL2	XRCC2	MARCH1
KDELC2	LOC643387	FSTL5	EFNA5
ACSF3	RAP1GAP	DOT1L	DKFZP434H
FZD5	PCDHGC3	IFITM1	ZNF669
RPPH1	SMARCC1	SERF2	TRH
LOC100133612	FOXO3	NPAS1	CCL4
LAYN	C15orf2	IRS1	FAM132A
LINC00304	DUSP4	ZYX	TSSC4
SCG2	PRR21	STOX2	NEIL3
LRRC16B	RHCE	KISS1R	EGR1
DDX11L1	PCSK1	OR6A2	LRRC29
TTC36	NEK4	GPR176	ZNF672
NUDT7	FRK	NTF4	UPK1B
FZD7	C15orf63	KCNJ13	CCL5
PPP1R3E	E2F5	ARHGEF5	HES5
TTC34	AQP12B	KIAA1430	MRVI1
C11orf65	RHD	LMNB2	FBXW7
SNAI3	PDE6A	DCHS1	ETF1
CUL3	TCTA	CHP	TOX3
TMEM55B	FUCA2	NUCB1	PCNXL2
LOC100288069	TMEM87A	MAP2	UMPS
MPZL3	EEF1D	ZNF212	CCL7
FAM92B	D2HGDH	FNIP2	LOC388588
DGKD	RPA2	MUM1	TRIM22
TTC5	PDGFRB	EIF3F	TMEM144
MIR4251	TDGF1	OIP5	F2R
ANKK1	FYN	PEG3	CCDC113
CTU2	RPAP1	MYL1	SH3BP5L

STK16	EGR3	ZNF282	WNT5A
DHRS1	LOC728323	SH3RF1	CCL8
RNF214	RPL11	MIDN	RNF223
PABPN1L	PFDN1	BRSK2	IFITM3
KLF7	TGM4	BAHD1	C4orf43
CMTM5	GABBR1	POLD1	F2RL1
LOC283143	DKFZP434L187	NCL	TMEM208
LOC400548	EIF4EBP1	CUL1	OR2G3
ADAM23	PP14571	RXFP1	CNBP
RNASE11	RPL22	R3HDM4	CCL11
BCL9L	PGGT1B	TRIM66	MIR200A
LOC400550	TKT	FAN1	DEAF1
NRP2	GABRR1	PPP2R1A	UFSP2
TPPP2	PLDN	NDUFA10	F2RL2
FOXR1	EPB49	TRIM24	C16orf80
LOC400558	LOC100286922	SPCS3	OR2G2
CFLAR	RPS6KA1	C19orf6	ZNF80
RNASE8	PITX1	TSPAN32	CCL13
C11orf53	TMF1	MAPKBP1	MIR200B
C16orf74	GABRR2	PRKCG	IPO7
PER2	GREM1	NDUFS1	DDX60
MRPL52	EPHX2	MGAM	F12
C11orf34	BOK-AS1	TRAPPC11	FHOD1
SNORD68	RSC1A1	TPGS1	OR2C3
CDK5R2	POU4F3	TSSC4	ZNF148
PSMB11	CLEC3B	GOLGA8A	CCL14
C11orf87	GCNT2	PRRG2	FLJ42875
LOC727710	RPUSD2	SEPT2	IFITM2
RQCD1	CLN8	DGKI	CDKN2AIP
OR4K14	MIR4269	MLF1IP	FABP6
C11orf92	SCNN1D	REEP6	BRD7
LOC732275	PPIC	TRIM22	TRIM11
LRRFIP1	TNNC1	RTF1	MAPKAPK3
OR4L1	GJA1	KLK7	CCL15
C11orf88	TUBGCP4	NEU2	ANKRD65
LOC100128881	EXT1	ATP6V1F	TRIM3
GPR55	UBE2F-SCLY	NBLA00301	ODZ3
OR11H6	SDHB	IZUMO4	FAT2
MIR34B	PPP2CA	IFITM3	ZDHHC1
LOC100129617	UBA7	CYFIP1	C1orf124
TRIP12	GCLC	KLK6	SLMAP
KLHL33	EHD4	NPPC	CCL16
MIR34C	EXTL3	ACCN3	MIR429
LOC100130015	MIR2467	WWC2	OR7E12P
ECEL1	SRSF4	SCAMP4	LRP2BP
MDP1	PPP2R2B	DEAF1	FBN2
DDI1	USP4	MGA	PARD6A

SYCE1L	GLO1	KLK10	OBSCN
EIF4E2	DUOX2	PAX3	MANF
FITM1	EYA1	PDIA4	CCL18
CARD17	MIR4440	CEP44	FAM138F
LOC100287036	SKI	ADAT3	LYVE1
TTLL4	PURA	IFITM2	PDGFC
REM2	UQCRC1	VPS39	FER
LOC643733	GLP1R	PTGIR	PLLP
MIR1910	MYEF2	PDCD1	NTPCR
HDAC4	FABP4	UBE3C	RAB7A
C14orf21	MIR4786	SNX25	CCL23
LOC643923	SLC2A5	ZNF554	LOC643837
MIR3182	RPS14	TRIM3	KCNQ1OT1
FARP2	VIPR1	FAM189A1	FSTL5
ADCY4	GMPR	PTPRH	FGF1
CLDN25	NDUFAF1	PDE6D	CKLF
C16orf95	FABP5	FAM131B	EFCAB2
FARSB	MIR4441	TKTL2	ACOX2
LOC283624	SLC9A1	GRIN3B	SH3GL1P1
RPL23AP64	SGCD	OR7E12P	TMEM88B
MIR4720	WNT5A	CCNDBP1	PKP3
ABCB6	GNL1	RPL18	STOX2
TSSK4	NUSAP1	SERPINE2	FGFR4
LOC100132078	PTK2B	FAM115A	FAM96B
MIR4722	SRM	CBR4	KIAA1804
ARPC2	SKP1	ZNF57	BAP1
DHRS4L2	ZNF35	KCNQ1OT1	SLC4A1
LOC100288346	GPLD1	C15orf2	C1orf233
MIR4719	TMEM85	RPL28	SWAP70
ARL4C	FDFT1	PPP1R7	KIAA1430
SNORD8	TAF12	DNAJB6	FOXD1
BACE1-AS	SLC6A7	MGC45800	TPPP3
ABI2	IFRD2	JSRP1	ZNF496
RNASE10	GPR6	PKP3	CGGBP1
MIR4301	SPTBN5	C15orf63	SMARCE1
RAMP1	FGFR1	RPS5	FAM138A
OR6S1	TCEA3	PSMD1	DENND5A
CASP12	SLC12A2	ABCF2	FNIP2
SPEG	MAPKAPK3	DDX60L	FOXI1
C14orf23	GPR31	MOB3A	CES1P1
LOC100526771	CTDSPL2	DENND5A	C1orf198
LANCL1	FGL1	TMEM87A	RUVBL1
OR4N2	TCEB3	RPS9	STAT3
HSPB2-C11orf52	SLC22A4	PTH2R	WASH7P
NMUR1	SEMA3B	FASTK	RRP8
OR4K2	GPX5	NAF1	SH3RF1
FXYD6-FXYD2	KLF13	C19orf21	FLT4

STK25	FNTA	RRP8	PRMT7
OR4K13	TNFRSF1B	RPAP1	HIST3H2A
MIR4491	SLC22A5	RPS11	CADPS
COPS8	SLMAP	PTMA	STAT5A
OR4K17	GRIK2	ABCB8	MIR551A
MIR4492	DUOX1	WDR17	SIRT3
CAPN10	ADAM2	PLK5	RXFP1
OR4N5	TP73	SIRT3	GABRA1
MIR4693	SNX2	DKFZP434L187	DUS2L
SP140	MANF	RRAS	ZNF670
OR11G2	GRM1	PTPRN	B4GALT4
LOC100652768	MAGEL2	PAXIP1	STAT5B
IKZF2	GATA4	ZFP42	CDK11A
OR11H4	TNFRSF4	C2CD4C	OR52A1
FASTKD2	SPARC	OR52A1	SPCS3
RNASE9	ACOX2	PLDN	GABRA6
PASK	GRM4	CLEC11A	LPCAT2
OR5AU1	DLL4	SNORD20	NLRP3
ATG4B	GEM	KLHDC10	SNX4
POTEG	ZBTB17	SPATA4	TADA2A
OBSL1	SPINK1	CIRBP-AS1	SLC35E2B
MIR208A	BAP1	ARFIP2	ARFIP2
DNPEP	GSTA1	GREM1	TRAPPC11
C14orf165	INO80	SEPW1	GABRB2
SH3BP4	GFRA2	RPE	HEATR3
OR11H12	SLC30A2	SSPO	FAM36A
KCNE4	SPOCK1	ENPP6	SUCLG2
RNASE13	HYAL3	C19orf25	HNF1B
NGEF	GSTA2	OR5E1P	LOC728716
OR4Q3	PPP1R14D	RPUSD2	OR5E1P
SNORD82	GLI4	SLC1A5	MLF1IP
OR4M1	LUZP1	RPL37A	GABRG2
PNKD	SRP19	NUP205	SLC38A7
RNASE12	CADPS	ASB5	C1orf96
SNED1	GSTA3	ATP8B3	HESX1
POTEM	MTMR10	OR10A3	MLX
GIGYF2	GML	TUBGCP4	LOC729737
LOC642426	PRDM2	SLC8A2	OR10A3
TRAF3IP1	TAF7	SAG	NBLA00301
ECRP	HYAL2	AHCYL2	GABRP
ABCA12	GSTA4	C4orf38	OGFOD1
C14orf176	ZNF770	DIRAS1	OR2M5
SNORD51	GNRH1	FXC1	NR1I2
CBLN3	LAPTM5	EHD4	THRA
CNPPD1	TCF7	SNRNP70	OR4F29
SNORD9	SUCLG2	CCL20	FXC1
STK36	GTF2H4	TNPO3	MAP9

DHRS4L1	HAUS2	C4orf39	GDF9
CPS1-IT1	NPBWR1	ZNF555	LRRC36
SNORD126	ARID1A	RBMXL2	OR2M3
ICOS	TCOF1	DUOX2	MBD4
MIR208B	HESX1	SPIB	TOP2A
GMPPA	GUCA1A	SLC4A3	LOC100129
MIR4307	FAM82A2	CLEC5A	RBMXL2
SMARCAL1	GPR20	C4orf45	WWC2
LOC100505967	SNHG3	C19orf26	GFRA3
ANO7	TGFBI	PGAP2	FBXL8
NEDD8-MDP1	BSN	NDUFAF1	OR2T12
PRLH	GUCA1B	AURKC	H1FX
BCL2L2-PABPN1	DNAJC17	SLC11A1	DNAJC7
THAP4	GPT	MKRN1	LOC100130
MIR4707	NR0B2	TRIM60	PGAP2
ANKMY1	UBE2B	CSNK1G2-AS1	CEP44
SCLY	LIMD1	C11orf21	GLRA1
NOP58	HIST1H1C	NUSAP1	DOK4
ASB1	NOP10	SULT2B1	OR14C36
CAB39	GRINA	SP100	KALRN
PRKAG3	MMP23B	CNTNAP2	TUBG1
UGT1A10	UBE2D2	C4orf46	LOC100132
UGT1A8	CCRL2	ODF3L2	C11orf21
UGT1A7	HIST1H1D	TRPM5	SNX25
UGT1A6	NDNL2	TMEM85	GLRX
UGT1A5	GSR	SULT2A1	DDX28
UGT1A9	MMP23A	SPP2	OR2T34
UGT1A4	VDAC1	GIMAP2	UBA3
UGT1A1	UBA3	TMEM192	UBTF
UGT1A3	HIST1H1E	ADAMTSL5	LOC100132
FEV	C15orf24	UBQLN3	TRPM5
INO80D	GTF2E2	SPTBN5	MND1
PID1	KCNAB2	SYT5	GM2A
ATG16L1	WNT8A	TNP1	TSNAXIP1
ANKZF1	RPL14	OR2F1	OR2T10
USP40	HIST1H1B	RWDD4	RRP9
HJURP	PAK6	TMPRSS9	PCGF2
STRADB	HAS2	IGF2-AS1	LOC100133
HES6	FCN3	CTDSPL2	UBQLN3
ACCN4	ST8SIA4	TNNI3	TKTL2
DOCK10	SEC22C	TNS1	GRK6
MREG	HIST1H1T	SLC13A4	COQ9
WDR12	CASC5	CCDC111	OR2T4
PECR	NRG1	NDUFS7	MAGI1
C2orf83	AKR7A2	BET1L	ZNF207
MFF	REEP5	KLF13	LOC100133
CXCR7	RRP9	TNNT1	RNF141

RNPEPL1	HIST1H2AE	TUBA4A	CBR4
CYP20A1	AVEN	COPG2	GPX3
MARCH4	HNF4G	TRIML2	CIAPIN1
WDFY1	ALDH4A1	C19orf35	OR2T11
NYAP2	NME5	CEND1	STXBP5L
ALS2	MAGI1	DUOX1	TAF15
ZDBF2	HIST1H2AD	TULP2	LOC100133
USP37	ATP10A	SUMO1	IGF2-AS1
CTDSP1	HSF1	TPK1	FHDC1
MPP4	EIF4G3	CCDC110	GRIA1
GAL3ST2	PDLIM4	WASH5P	THAP11
TMBIM1	CACNA2D2	CYB5R2	OR2B11
RAB17	HIST1H2BD	MAGEL2	GUCA1C
RNF25	GJD2	NR1H2	RND2
COPS7B	IKBKB	VIL1	DDX11L1
RAPH1	TNFRSF25	SND1	BET1L
CDK15	EIF4EBP3	CYP4V2	MGC45800
TMEM237	SLC22A14	ONECUT3	NR3C1
NBEAL1	HIST1H2BB	TRIM34	PDP2
CFLAR-AS1	STARD9	DLL4	WDR64
MRPL44	IL7	ZNF8	IQCB1
TRAK2	TNFRSF14	WNT6	PIP4K2B
TRPM8	PCDHGB4	SND1-IT1	TTC34
ATG9A	SLC22A13	LOC285441	CEND1
MLPH	HIST1H1A	MEX3D	DDX60L
FAM134A	VPS18	CDHR5	GRM6
GLB1L	IMPA1	INO80	ZNF319
SPAG16	TNFRSF18	ZNF17	C1orf131
CHPF	CDC23	XRCC5	DZIP3
IQCA1	VPRBP	DENND2A	CNTNAP1
ALS2CR8	HCRTR2	LOC285501	LOC100288
NHEJ1	SQRDL	FLJ45445	CYB5R2
FAM124B	IDO1	TOLLIP	NAF1
TM4SF20	PER3	PPP1R14D	GTF2H2
C2orf54	ADAM19	ZNF28	RANBP10
TUBA4B	IP6K1	ZNF142	EDARADD
ARMC9	HDAC2	ZNF777	VPRBP
EFHD1	ZFP106	LOC339975	TCAP
SPHKAP	EIF3E	PRSS57	MIR4251
WNT10A	MAP3K6	TRIM68	TRIM34
SLC19A3	HDAC3	MTMR10	WDR17
ILKAP	PSMD6	MZF1	GZMA
ITM2C	HFE	FZD5	NOD2
RHBDD1	SPATA5L1	TMEM176B	MRPL55
ING5	KCNQ3	TRIML1	PSMD6
PLCD4	DHRS3	FAM138F	AOC3
MGC16025	P4HA2	PIDD	MIR4417

TMEM169	EXOG	ZNF770	CDHR5
DNER	HIVEP1	ZNF132	ZFP42
B3GNT7	CHAC1	SCG2	GZMK
SP140L	KCNS2	HIPK2	DPEP2
STK11IP	VAMP3	LOC340017	HIST3H2BB
MOGAT1	ATG12	LINGO3	TOMM70A
AGAP1	XYLB	KCNQ1DN	BECN1
TWIST2	HIVEP2	HAUS2	TOLLIP
PARD3B	C15orf29	ZNF134	SPATA4
DIS3L2	LOXL2	FZD7	HARS
NEU4	C1orf38	WDR91	DPEP3
ICA1L	NREP	ANKRD37	SLC35F3
AP1S3	OXSR1	FAM138A	PARP3
SGPP2	HLA-A	MMP26	KRT38
ALS2CR12	WDR76	FAM82A2	TRIM68
SPATA3	LPL	ZNF135	ENPP6
TMEM198	H6PD	CUL3	HEXB
ZFAND2B	CNOT8	REPIN1	PAPD5
CPO	PARP3	LOC389247	B3GALNT2
MDH1B	HLA-B	LOC100288123	COX17
FBXO36	TMEM62	AKIP1	KRT37
MTERFD2	LY6E	DNAJC17	PIDD
UBE2F	ISG15	ZNF137P	ASB5
OTOS	RAB9BP1	DGKD	HINT1
MYEOV2	ZNF197	HILPDA	AKTIP
OR6B3	HLA-C	TRIM61	C1orf150
FAM117B	SEMA6D	MIR1909	CD96
LOC150935	LY6H	C11orf16	KRT36
PKI55	PLCH2	NOP10	KCNQ1DN
C2orf67	HAND1	ZNF154	SH3D19
LOC151171	RBM6	STK16	HK3
LOC151174	HLA-DMA	FSCN3	GINS3
METTL21A	SPG11	HELT	LOC148824
CCNYL1	LYN	MIR1227	TFG
ALS2CR11	SDC3	TMEM9B	CDK5R1
CCDC140	MED7	NDNL2	MMP26
SLC23A3	RBM5	ZNF175	C4orf38
LOC151300	HLA-DMB	KLF7	HMGCR
GPBAR1	ELL3	ATP6V0A4	NDRG4
SLC16A14	MATN2	LOC401164	LOC149134
LOC151475	CROCC	MIR3187	ST3GAL6
LINC00471	MYOT	NRIP3	HAP1
LOC151484	NME6	C15orf24	AKIP1
MSL3P1	HLA-DOA	TEAD2	C4orf39
WDR69	NIPA2	ADAM23	HMMR
C2orf57	MCM4	TAS2R3	ACD
PIKFYVE	PUM1	FAM92A3	EXOC8

LOC200726	NRG2	MIR4321	ARL6IP5
TIGD1	EIF1B	ASCL3	ZNHIT3
LOC200772	HLA-DOB	PAK6	C11orf16
CCDC108	C15orf48	PPFIA3	C4orf45
C2orf72	MMP16	NRP2	HNRNPAB
CXXC11	CEP104	TAS2R4	FTO
UNC80	CXCL14	HSP90AA6P	RNF187
RUFY4	TRAIP	MIR4745	POLQ
DUSP28	HLA-DPA1	CHRNA10	EFTUD2
ESPNL	C15orf41	CASC5	TMEM9B
ECEL1P2	MOS	PLA2G4C	TRIM60
LOC348761	KLHL21	PER2	HNRNPH1
C2orf62	H2AFY	CPA4	IRX6
RBM44	ARIH2	C4orf47	CNST
AQP12A	HLA-DPB1	PNPLA2	ALDH1L1
KLHL30	ZFYVE19	AVEN	RPL23
PLEKHM3	MSR1	NAPA	NRIP3
C2orf80	SLC35E2	CDK5R2	RBM46
RESP18	SMAD5-AS1	PRKAG2	HRH2
C2orf82	ARL6IP5	DUX4L4	IRX3
OR6B2	HLA-DPB2	PHRF1	PLD5
DYTN	FRMD5	ATP10A	PDIA5
ASB18	MSRA	SIGLEC5	CCL4L1
FLJ43879	ZBTB40	RQCD1	ASCL3
VWC2L	RNF14	ZC3HC1	C4orf46
MIR149	NPRL2	GALNTL6	HSD17B4
MIR153-1	HLA-DQA1	SCUBE2	FAM65A
MIR26B	DISP2	GJD2	C1orf100
DNAJB3	MYBL1	UBE2M	RPP14
MIR375	MFN2	LRRFIP1	NR1D1
SNORA41	SNCAIP	LUC7L2	CHRNA10
LOC643387	CXCR6	FRG2	TIGD4
PRR21	HLA-DQA2	SIGIRR	HSPA4
LOC646324	CHRFAM7A	STARD9	C16orf57
LOC646736	MYC	CYTH2	IBA57
PRSS56	PTPRU	GPR55	HHLA2
AQP12B	TTC37	MRPS33	MED24
SNORA75	CSPG5	SLED1	PNPLA2
SCARNA6	HLA-DQB1	RIC8A	TMEM154
SCARNA5	ULK4P3	VPS18	HSPA9
SNORD11	NBN	ZNF264	ELMO3
SNORD70	CELA3A	TRIP12	OR2T6
D2HGDH	CLINT1	NUB1	FSTL1
LOC728323	CCR9	FLJ38576	HDAC5
DIRC3	HLA-DQB2	MRPL17	PHRF1
SNORD11B	ULK4P1	SQRDL	TMEM192
LOC100129175	NDUFB9	NCR1	NDST1

PP14571	WASF2	ECEL1	SETD6
LOC100130451	PCDHGA8	TAS2R5	LOC255654
LOC100286922	USP19	DUX4L6	FAM107A
MIR1471	HLA-DRA	EPS8L2	GJC1
MIR1244-1	ARHGAP11B	ZFP106	SCUBE2
MIR548F2	NEFM	NAPSA	RWDD4
LOC100329109	ANGPTL7	EIF4E2	HTR1A
BOK-AS1	PCDHA9	CHPF2	ESRP2
MIR1244-3	SLC38A3	DUX4L5	C1orf101
MIR1244-2	HLA-DRB1	STK33	NISCH
MIR3131	ATPBD4	SPATA5L1	PSME3
MIR4268	NEFL	KLK4	ZBED5
MIR3130-1	HNRNPR	TTLL4	CCDC111
MIR3130-2	MATR3	NCAPG2	HTR4
MIR2355	CYB561D2	DUX4L3	FAM192A
MIR3132	HLA-DRB5	CHID1	PGBD2
MIR4269	C15orf57	CHAC1	FILIP1L
LOC100507443	TONSL	ZNF432	EIF1
UBE2F-SCLY	SRRM1	HDAC4	SIGIRR
MIR4439	KIAA0141	CHCHD3	TRIML2
MIR4776-1	TMEM115	MIR578	IK
MIR4777	HLA-DRB6	OR51G1	CENPT
MIR2467	C15orf23	C15orf29	OR2L13
MIR4775	NKX3-1	DHX34	MGLL
MIR4440	CNKSR1	FARP2	NBR2
MIR4786	JAKMIP2	WDR60	RIC8A
MIR4776-2	RPP14	LINC00290	CCDC110
MIR4441	HLA-E	OR51B4	IL3
	BMF	WDR76	CHD9
	NOV	SAE1	OR14A16
	UBE4B	FARSB	TWF2
	PJA2	RBM28	RAMP2
	FAM107A	LOC728175	MRPL17
	HLA-F	OR51B2	CYP4V2
	SHF	TMEM62	IL4
	ODF1	TRIM28	C16orf70
	MAD2L2	ABCB6	HNRNPU-A[illegible]
	DDX46	TMEM140	COPG
	WDR6	DUX4L2	VAT1
	HLA-G	OR52N1	EPS8L2
	DUOXA1	SPG11	LOC285441
	TNFRSF11B	ZNF256	IL5
	PDPN	ARPC2	GFOD2
	SLC23A1	GIMAP4	VN1R5
	RASSF1	LOC731424	MYH15
	HLA-H	RIC3	LEPREL4
	SNORD107	ELL3	STK33

OPRK1	LILRB2	LOC285501
GMEB1	ARL4C	IL6ST
GNPDA1	GIMAP5	C16orf48
NISCH	CLDN24	LOC339529
HLA-J	SLC25A22	TMCC1
CHST14	NIPA2	CCT6B
PCM1	ZNF211	CHID1
NUDC	ABI2	RNF175
SRA1	TMEM176A	IL9
TREX1	LOC100288255	SLC7A6OS
HLA-L	ATHL1	LOC339535
CASC4	C15orf48	PDZRN3
PDE7A	TRAPPC2P1	C1QL1
MASP2	RAMP1	OR51G1
RAD50	TRPV6	LOC339975
SCN11A	MIR1305	IL12B
HMGA1	OR51G2	NLRC5
TUBGCP5	C15orf41	OR6F1
PDGFRL	ZNF274	PLXND1
SRSF10	SPEG	RUNDC3A
KIF20A	AGK	OR51B4
TUSC2	MIR4276	TRIML1
HSF2	OR51E2	IL13
TGM7	ZFYVE19	CCDC135
ENPP2	ZNF460	OR2W3
UTS2	LANCL1	RAD54L2
G3BP1	METTL2B	STARD3
TWF2	MIR3945	OR51B2
HSPA1A	PTDSS2	LOC340017
CATSPER2	FRMD5	IRF1
PENK	RUVBL2	CAPNS2
RER1	NMUR1	OR2T8
APBB3	TRPV5	FRMD4B
TRAK1	MIR3688-1	DDX52
HSPA1B	MOB2	OR52N1
NIPA1	DISP2	PRSS48
PLAG1	LILRB1	ISL1
RCAN3	STK25	MT4
TNIP1	ZC3HAV1	OR2T3
DHX30	LOC100505989	STAB1
HSPA1L	FAM160A2	DUSP14
PLA2G4E	CHRFAM7A	RIC3
PLAT	KDELR1	ANKRD37
MST1P2	COPS8	ITGA1
SEC24A	AKR1B10	B3GNT9
SACM1L	LOC100506013	OR2T29
HSP90AB1	TRIM5	FAM208A

TRIM69	ULK4P3	SYNRG
PLEC	LILRB5	SLC25A22
MST1P9	CAPN10	LOC389247
C5orf4	ACTR3B	ITGA2
SCAP	LOC100506085	NKD1
HTR1B	SYT8	IRF2BP2
PAR1	ULK4P1	RYBP
PNOC	SLC27A5	CASC3
PADI2	SP140	ATHL1
FAM114A2	KIAA1147	TRIM61
LAMB2P1	LOC100506122	ITK
HTR1E	PRKCDBP	RSPRY1
C15orf43	ARHGAP11B	FAM89A
PMP2	LILRB4	C3orf27
LYPLA2	IKZF2	IKZF3
BRD8	FAM40B	OR51G2
EXOSC7	LOC100506229	HELT
ID4	ODF3	KCNMB1
LOC145663	ATPBD4	CCDC102A
POLB	KLK11	C1orf31
PARK7	FASTKD2	ABHD14A
TCERG1	ESYT2	GPATCH8
PDZRN3	MIR3688-2	OR51E2
IFNGR1	OSBPL5	LOC401164
LOC145845	C15orf57	KCNN2
POLR2K	LILRA1	CMTM1
CTRC	PASK	C1orf229
HNRNPA0	ZNF398	C3orf17
RAD54L2	LRRC56	KCNH4
IGF2R	C15orf23	PTDSS2
TMCO5A	LILRB3	FAM92A3
POU5F1B	ATG4B	KIF2A
ACOT7	KIAA1549	NRN1L
SPINK5	MRGPRE	OR2M1P
FRMD4B	BMF	POC1A
IL17A	LILRA3	SUZ12
ZSCAN29	OBSL1	MOB2
PPP2CB	MLL3	HSP90AA6P
DNAJC8	ART5	TNPO1
SOX30	SHF	CMTM3
STAB1	LILRA2	OR11L1
IMPG1	DNPEP	ABI3BP
TTBK2	EXOC4	FAM215A
PPP2R2A	TRIM78P	SBF2
CLSTN1	DUOXA1	C4orf47
KIF3A	KPTN	LCP2
NBEAL2	SH3BP4	C16orf78

ITPR3	GALNT11	OR2L8
CDAN1	TRIM6	PVRL3
PPP3CC	SNORD107	KRT23
AKR7A3	KLK8	FAM160A2
SYNPO	KCNE4	DUX4L4
FAM208A	LRRC4	LECT2
JARID2	OR52E2	SNX20
STRC	CHST14	OR2AK2
PKIA	PNKP	CHMP2B
SPEN	NGEF	TMEM98
RHOBTB3	LMBR1	TRIM5
KLHL18	OR52J3	GALNTL6
KIFC1	CASC4	LMNB1
CSNK1A1P1	U2AF2	RLTPR
PRKDC	SNORD82	OR2L3
KDM1A	LINC00244	DNAH1
ABLIM3	OR51L1	AATF
LARS2	TUBGCP5	SYT8
KIF25	ATF5	FRG2
OTUD7A	PNKD	LNPEP
PTK2	NOM1	KCTD19
WDTC1	OR51A7	OR2M2
ELL2	TGM7	LRIG1
RYBP	PPP6R1	TUBG2
KPNA5	SNED1	PRKCDBP
SPRED1	CCDC136	SLED1
PVT1	OR51S1	LOX
KIAA0090	CATSPER2	CMTM4
HMGXB3	CARD8	OR2T33
NAT6	GIGYF2	ERC2
LAMA2	PARP12	NKIRAS2
PGBD4	OR51F2	ODF3
PEX2	NIPA1	FLJ38576
KIF1B	ZC3H4	LTC4S
ARHGAP26	TRAF3IP1	CMTM2
ABHD14A	CREB3L2	OR2M7
LAMA4	OR52R1	APPL1
ADAL	PLA2G4E	CCDC56
RAB2A	RPL13A	OSBPL5
PLEKHM2	ABCA12	DUX4L6
FSTL4	LRRC61	CD180
POC1A	OR52M1	BEAN1
LPA	TRIM69	OR2G6
EXD1	SYNGR4	ZBTB20
RAD21	SNORD51	PSMC3IP
OTUD3	C7orf49	LRRC56
SEPT8	OR52K2	DUX4L5

TMEM158	PAR1	SMAD5
LTA	LILRA4	CPNE2
FSIP1	CNPPD1	OR2T2
RP1	GCC1	OR5K1
KAZN	OR5P2	SOCS7
PPIP5K2	C15orf43	MRGPRE
NDUFAF3	ZIM2	DUX4L3
LTB	STK36	MAN2A1
RHOV	ZNF767	GPR114
RPL7	OR5P3	OR2T5
CAMTA1	LOC145663	OR5H1
ACSL6	NUP62	SOST
MYRIP	CPS1-IT1	ART5
MARCKS	TTC26	MIR578
UBR1	OR2D3	MAP1B
RPL8	LOC145845	PRSS54
DNAJC16	HSPBP1	OR14I1
PHF15	ICOS	GNL3
PTPN23	JHDM1D	RAPGEFL1
MAK	OR2D2	TRIM78P
PATL2	TMCO5A	LINC00290
RPL30	PPP1R15A	MCC
UBR4	GMPPA	CES5A
LARP1	TMUB1	OR2T27
DNAH1	OR52W1	FAM162A
MAN1A1	ZSCAN29	SLC25A39
LPCAT4	ZNF324	TRIM6
RPS20	SMARCAL1	LOC728175
ATP13A2	SLC37A3	MEF2C
HARS2	OR56A4	GPR97
HIGD1A	TTBK2	OR2T35
MAS1	KLK5	SEC22A
PLA2G4F	ANO7	HIGD1B
SDC2	KRBA1	OR52E2
TARDBP	OR56A1	DUX4L2
TNFAIP8	CDAN1	MAP3K1
LRIG1	PRKD2	CNEP1R1
MCM3	PRLH	TRIM67
LRRC57	FAM71F1	ATP2C1
SDCBP	SYT9	CDK12
CELA3B	STRC	OR52J3
GEMIN5	ZNF473	FAM160A1
ERC2	THAP4	MFAP3
MDFI	SLC35B4	CCDC79
C15orf55	OR52B4	MAP1LC3C
SFRP1	CSNK1A1P1	FOXP1
ICMT	KLK13	KRT20

PCDHGA12	ANKMY1	OR51L1
APPL1	TMEM209	LOC731424
ME1	C11orf40	MGAT1
HMGN2P46	OTUD7A	CES4A
SFTPC	CCDC9	OR2W5
PADI4	SCLY	ZBTB11
LRRTM2	OR6W1P	NLE1
GNL3	OR52I2	OR51A7
MEA1	SPRED1	CLDN24
SLC24A5	PRPF31	MOCS2
ST3GAL1	NOP58	EXOC3L1
TMEM50A	ADCK2	OR13G1
CCDC69	OR51E1	MORC1
FOXP1	PGBD4	FNDC8
MAP3K4	FGF21	OR51S1
LOC283683	ASB1	LOC100288
SLA	PLXNA4	MSH3
STX12	UBQLNL	LOC283856
PCDHB5	ADAL	DUSP5P
SPCS1	SNORD35A	SPCS1
MAP3K5	CAB39	CWC25
OR4N4	ZC3HAV1L	OR51F2
SLC7A2	LOC143666	MIR1305
CLIC4	EXD1	MSX2
FBXL21	SNORD34	LOC283867
SETD2	PRKAG3	SNRPD2P2
MEP1A	LOC93432	ACAD9
LOC283710	OR10A5	SLFN12
SLC18A1	FSIP1	OR52R1
SYF2	SNORD33	MIR3140
KLHL3	UGT1A10	NAIP
PRSS50	CPA5	MT1DP
MICB	OR2AG1	RPS7P5
FAM98B	RHOV	ASTE1
SLC20A2	SNORD32A	KLHL11
CHD5	UGT1A8	OR52M1
HAVCR1	CEP41	MIR4276
RBM15B	DNHD1	NDUFA2
MLN	UBR1	TEPP
PLA2G4D	SIGLEC7	LOC646627
SNAI2	UGT1A7	KLF15
C1orf144	C7orf29	RHOT1
SNORD63	SCGB1C1	OR52K2
GMPPB	PATL2	MIR3945
MLLT4	BBC3	NDUFS4
HERC2P3	UGT1A6	LOC388276
SNTB1	TSGA13	SNORA14B

LDLRAP1	C11orf42	GTPBP8
SNORA74A	LPCAT4	C17orf79
ARHGEF3	DKKL1	OR5P2
MOCS1	UGT1A5	MIR3688-1
GOLGA6L1	AGAP3	NEUROG1
SPAG1	NLRP6	C16orf86
NOC2L	PLA2G4F	LOC731275
CYFIP2	SIGLEC9	TAGLN3
ABHD5	UGT1A9	UTP6
MOG	C7orf13	OR5P3
GOLGA8G	NS3BP	LOC100505
SQLE	LRRC57	NPM1
FBXO2	SIGLEC8	CES1P2
PKD2L2	UGT1A4	LOC10013C
C3orf18	OR9A4	RBM15B
MSH5	OR56B4	GSDMB
GOLGA8IP	C15orf55	OR2D3
STAR	GPR77	LOC100506
FBXO6	UGT1A1	NPY6R
UQCRQ	OR9A2	MIR138-2
SS18L2	LOC255512	LOC100287
MUT	HMGN2P46	SEC61A1
SNORD108	CHMP2A	ATXN7L3
STC1	UGT1A3	OR2D2
PLA2G2D	C7orf34	LOC100506
AFF4	OR52B2	PAM
SHISA5	LOC283683	MIR328
MYB	DHDH	LGALS8-AS
SNORD109A	FEV	PIK3R4
STK3	TMEM139	PLXDC1
OR4F3	C11orf35	OR52W1
IL17B	OR4N4	LOC100506
ZDHHC3	ZNF544	PCDH1
MYO6	INO80D	LOC643714
SNORD109B	NOBOX	MIR1182
TACC1	OR51F1	PODXL2
RNU11	LOC283710	LYZL6
SLC27A6	UBE2S	OR56A4
ZMYND10	PID1	LOC100506
RPL10A	OR2A14	PCDHGC3
SNORD115-1	OR51B5	LOC643802
TAF2	FAM98B	MIR1537
HSPB7	SLC6A16	ARHGEF3
SNX24	ATG16L1	ARHGAP23
TEX264	OR6B1	OR56A1
NEDD9	KRT8P41	MIR4453
WHAMMP3	PLA2G4D	PCSK1

TCEA1	TFPT	CRNDE
ARHGEF16	ANKZF1	LINC00184
MRPL22	OR2F2	TRAT1
CCDC72	OR51V1	FKBP10
NEU1	HERC2P3	SYT9
POTEB	STRN4	MIR3688-2
TCEB1	USP40	PDE4D
AHDC1	ZNF786	MT1IP
PCDHB1	H19	TSNAX-DISC
ZNF589	GOLGA6L1	IMPG2
NFKBIE	CCDC106	C17orf75
FMN1	HJURP	OR52B4
TERF1	PRSS37	PDE6A
SMPDL3B	EFCAB4A	LOC644649
TMED7	GOLGA8G	MIR3123
HEMK1	EPN1	C3orf18
NFKBIL1	STRADB	WNK4
SNORD64	KLF14	C11orf40
TG	TMEM80	PDGFRB
PRO0611	GOLGA8IP	KIAA0895L
ISOC1	GLTSCR2	MIR3124
IP6K2	HES6	TIMMDC1
NFYA	C7orf45	C17orf53
PAR4	OR10A4	OR52I2
KLF10	SNORD108	PFDN1
LINC00339	GLTSCR1	SNORA46
SAR1B	ACCN4	MIR3620
SFMBT1	SVOPL	PLA1A
NMBR	OLFML1	TMUB2
PAR-SN	SNORD109A	OR51E1
TPD52	EHD2	PGGT1B
SSU72	DOCK10	SNORA50
DCTN4	MTPN	MIR3916
NCKIPSD	LOC283299	TEX264
NOTCH4	SNORD109B	DHX58
LOC348120	KLK14	UBQLNL
TRHR	MREG	PIK3R1
UBIAD1	LRGUK	MIR3935
MZB1	C11orf36	LOC100506
PHF7	SNORD115-1	HEMK1
NT5E	KLK12	MMP28
MRPL42P5	WDR12	LOC143666
TRPS1	ASB10	PITX1
PADI1	NLRP10	LOC100505
PAIP2	WHAMMP3	LOC100506
TLR9	SHANK1	SFMBT1
OPRM1	PECR	DHRS11

C15orf52	PRSS58	OR10A5
TSTA3	NLRP14	PMCHL2
PLA2G2E	POTEB	LOC100505
CDKL3	NOSIP	ZNF670-ZN
LZTFL1	C2orf83	PHF7
PARK2	RNF32	DCAKD
GOLGA8E	ANO9	OR2AG1
TTPA	FMN1	POU4F3
WRAP73	ZNF580	CKLF-CMTN
PCDH12	MFF	MIR4753
P4HTM	TRY6	FBXO40
PBX2	LOC338651	GGNBP2
OR4M2	SNORD64	DNHD1
UBE2V2	HSD17B14	PPIC
SLC45A1	CXCR7	MIR4666A
FAM13B	LOC154761	TLR9
SLC6A20	B4GALNT4	MRM1
PCMT1	PAR4	SCGB1C1
OR4N3P	GP6	PPP2CA
COL14A1	RNPEPL1	MIR4677
HP1BP3	CLEC2L	SEMA5B
FAM53C	OR52L1	PLEKHH3
IL17RD	PAR-SN	C11orf42
PDCD2	VRK3	PPP2R2B
CTXN2	CYP20A1	MIR4671
UQCRB	C7orf55	WDR5B
CELA2B	OR2AG2	DBF4B
REEP2	LOC348120	NLRP6
C3orf75	ZNF581	MAPK9
ENPP1	MARCH4	MIR4427
HERC2P2	LOC154822	PARP14
VDAC3	OR52B6	MYO19
ZNF593	MRPL42P5	NS3BP
PRR16	PTOV1	PROP1
SNRK	WDFY1	IL17RD
ENPP3	TMEM213	COASY
C15orf53	OR10A2	OR56B4
WRN	C15orf52	PURA
MECR	PPP1R12C	GRAMD1C
COMMD10	NYAP2	SRCIN1
QRICH1	GIMAP8	LOC255512
PEX6	OVCH2	RAD17
C15orf54	GOLGA8E	ROPN1
YWHAZ	TRPM4	AARSD1
SDF4	ALS2	OR52B2
LARS	CRYGN	RARS
PXK	PDDC1	SIDT1

PEX7	OR4M2	KRTAP1-3
DUOXA2	ZNF586	C11orf35
ZNF7	ZDBF2	RASA1
MRTO4	ZNF425	PXK
CXXC5	MRGPRG	KRTAP1-1
SLC25A38	OR4N3P	OR51F1
PGC	FAM83E	RASGRF2
MIR211	USP37	RG9MTD1
ZNF16	LOC155060	KRTAP9-9
YTHDF2	KRTAP5-1	OR51B5
RAPGEF6	HERC2P2	RPS14
ULK4	EPS8L1	SLC41A3
PGK2	CTDSP1	KRTAP4-6
NF1P2	ZNF746	KRT8P41
FZD3	KRTAP5-3	RPS23
PADI3	C15orf53	SLC35A5
ERAP1	RASIP1	KRTAP2-1
FEZF2	MPP4	LOC283104
PGM3	ATP6V0E2	SGCD
HERC2P9	KRTAP5-4	TMEM45A
TUSC3	C15orf54	TTC25
ERRFI1	TMEM160	CSNK2A1P
KDM3B	GAL3ST2	SKP1
EBLN2	RBM33	FEZF2
PHF1	IFITM5	KRTAP4-12
WHAMMP2	DUOXA2	OR51V1
UBXN8	PIH1D1	SLC6A7
WNT4	TMBIM1	IFT57
PHAX	MGC27345	KRTAP1-5
ANO10	FAM99A	H19
PIM1	MIR211	SLC12A2
GOLGA8B	C19orf73	EBLN2
KAT6A	RAB17	KRTAP3-1
FBXO42	GALNTL5	EFCAB4A
RBM27	OR56B1	SLC34A1
DALRD3	NF1P2	SHQ1
PKHD1	PNMAL1	KRTAP3-2
EIF2AK4	RNF25	TMEM80
PSCA	GIMAP7	SLC22A4
RNF186	GVINP1	PBRM1
FLJ11235	HERC2P9	KRTAP9-2
SHQ1	TMEM143	OR10A4
PLAGL1	COPS7B	SLC22A5
CATSPER2P1	ZNF467	DPPA4
FZD6	OR52K1	KRTAP9-3
MXRA8	WHAMMP2	OLFML1
PCDHB18	ZNF444	SLIT3

PBRM1	RAPH1	TMEM39A
PLG	ZNF800	KRTAP9-8
LOC503519	OR52I1	LOC283299
NSMAF	GOLGA8B	SMN1
HES2	ZNF331	ZNF654
PCDHB17	CDK15	KRTAP17-1
CHDH	GIMAP1	C11orf36
PLN	OR51D1	SMN2
CKMT1A	EIF2AK4	ABHD10
LY6D	KLK15	PPP1R1B
GPN2	TMEM237	NLRP10
ZCCHC10	LOC202781	SNCB
IL17RB	OR52A5	CHDH
POLH	CATSPER2P1	TBC1D3F
SERINC4	NLRP2	NLRP14
RGS20	NBEAL1	SNX2
FBLIM1	C7orf33	IL17RB
GIN1	OR51B6	MIEN1
CACNA2D3	LOC503519	ANO9
POU3F2	ZNF416	SPARC
C15orf62	MRPL44	IFT122
JRK	FABP5P3	MRPL45
MED18	OR51M1	LOC338651
WDR55	CKMT1A	SPINK1
DCP1A	ZNF446	TBC1D23
POU5F1	TRAK2	VPS25
C15orf56	UBN2	B4GALNT4
TNKS	OR51Q1	SPOCK1
PQLC2	SERINC4	WDR52
ANKHD1	ZNF701	TMEM101
GLT8D1	TRPM8	OR52L1
PPARD	TAS2R39	SRP19
PHGR1	OR51I1	CACNA2D3
EIF3H	C15orf62	RAB11FIP4
CASZ1	ZNF83	OR2AG2
THG1L	ATG9A	STK10
ZNF167	TAS2R40	DCP1A
PPP1R10	OR51I2	GHDC
LOC645212	C15orf56	OR52B6
DGAT1	ZNF415	TAF7
TRNAU1AP	MLPH	GLT8D1
TMCO6	TAS2R41	KRTAP4-4
LMOD3	OR52D1	OR10A2
PPP2R5D	PHGR1	TAF9
CHEK2P2	CABP5	EAF2
GPAA1	FAM134A	TNS4
CPSF3L	CNPY1	OVCH2

TRIM36	OR52H1	TBCA
SEMA3G	LOC645212	LMOD3
PREP	SPHK2	FBXL20
LOC646214	GLB1L	PDDC1
ADAM18	LOC285889	TCF7
C1orf159	OR52N4	CLDND1
GALNT10	CHEK2P2	KRTAP9-4
KIF15	MEIS3	GALNTL4
PRIM2	SPAG16	ZNF354A
CXADRP2	FLJ40852	MUC13
ADAM9	OR52N5	KRTAP4-1
AURKAIP1	LOC646214	MRGPRG
RBM22	SLC17A7	TCOF1
ADAMTS9	CHPF	SEMA3G
PKIB	LOC285965	KRTAP4-5
LOC646278	OR52N2	KRTAP5-1
ADAM7	CXADRP2	NR2F1
MRPL20	NAT14	C3orf37
RIOK2	IQCA1	KRTAP4-3
PCBP4	FAM115C	KRTAP5-3
MAPK13	OR52E6	TGFBI
REREP3	LOC646278	NIT2
RIPK2	VN1R1	KRTAP4-2
AIM1L	ALS2CR8	KRTAP5-4
PCDHGC5	ZNF775	THBS4
ABHD6	OR52E8	POGLUT1
PRL	REREP3	KRTAP3-3
LOC653061	RCN3	IFITM5
TNFRSF10D	NHEJ1	TTC1
TMEM51	LOC285972	BBX
PCDHGC4	OR52E4	KRTAP2-4
LRTM1	LOC653061	FAM99A
PSMB1	ZNF304	UBE2B
LOC653075	FAM124B	ADAMTS9
TNFRSF10C	ATG9B	AOC4
XKR8	OR56A3	OR56B1
PCDHGB7	LOC653075	UBE2D2
C3orf14	TTYH1	PCBP4
PSMB8	TM4SF20	ZNF830
MIR626	TPI1P2	GVINP1
TNFRSF10B	OR56A5	VDAC1
ARHGEF10L	MIR626	PCNP
PCDHGB6	PNMAL2	SLFN11
KIAA1143	C2orf54	OR52K1
PSMB9	FLJ40288	WNT8A
MIR627	OR10A6	SENP7
TNFRSF10A	MIR627	RASL10B

VPS13D	PRR12	OR52I1
PCDHGB5	TUBA4B	XRCC4
HHATL	MESTIT1	ABHD6
PTK7	OR51T1	G6PC3
ANP32AP1	ANP32AP1	OR51D1
FGF17	ZNF471	ST8SIA4
ATAD3A	ARMC9	LRTM1
PCDHGB3	TAS2R60	ASB16
WDR48	OR51A4	OR52A5
PTPRK	SNORD116-19	REEP5
SNORD116-19	USP29	C3orf14
GGH	EFHD1	PGAP3
TMEM57	CTAGE6P	OR51B6
PCDHGB2	OR51A2	SERF1A
SELK	GOLGA6L6	ISY1
RGL2	PLEKHA4	HSPB9
GOLGA6L6	SPHKAP	OR51M1
WISP1	TSPAN33	NME5
PANK4	IFITM10	HEG1
PCDHGB1	LOC727924	ORMDL3
PROK2	ZNF71	OR51Q1
PRPH2	WNT10A	ENC1
LOC727924	AGBL3	KIAA1257
CPNE3	MIR210	TMEM106A
CAMK2N1	LOC728758	OR51I1
PCDHGA11	SCAF1	AP3B1
RNF123	SLC19A3	ARHGAP31
REV3L	OR6V1	LRRC37B
LOC728758	KRTAP5-5	OR51I2
FOXH1	OIP5-AS1	PDLIM4
ASAP3	CACNG8	KIAA1407
PCDHGA10	ILKAP	NT5C3L
KIF9	OR2A12	OR52D1
TRIM27	KRTAP5-2	PPAP2A
OIP5-AS1	GOLGA8C	KIAA1524
CHRNA6	CACNG7	RFFL
PNRC2	ITM2C	OR52H1
PCDHGA9	OR2A1	STC2
CSRNP1	KRTAP5-6	SELK
RHAG	PWRN1	ZPBP2
GOLGA8C	CACNG6	OR52N4
TRPA1	RHBDD1	PDE8B
PIGV	FAM71F2	PROK2
PCDHGA7	TMEM41B	LSM12
GORASP1	PWRN2	OR52N5
RING1	ZNF350	EIF4EBP3
PWRN1	ING5	EEFSEC

DOK2	STRA8	CCDC43
NBPF1	TRIM6-TRIM34	OR52N2
PCDHGA6	SNORD116-1	PCDHGB4
CDCP1	TSKS	NSUN3
BRD2	PLCD4	CNTD1
PWRN2	KLRG2	OR52E6
ASH2L	MIR483	CDC23
NECAP2	SNORD116-2	POPDC2
PCDHGA5	ZNF667	TMEM132E
CCDC71	MGC16025	OR52E8
RNF5	WDR86	ADAM19
SNORD116-1	SNORA3	NFKBIZ
MTMR7	SNORD116-3	SPACA3
DNAJC11	ELSPBP1	OR52E4
PCDHGA4	TMEM169	FGF18
NT5DC2	LOC349160	ATG3
RNY4	SNORA52	GJD3
SNORD116-2	SNORD116-4	OR56A3
CCNE2	LIN7B	HDAC3
RCC2	DNER	CCDC14
PCDHGA3	GSTK1	KRT222
SLC26A6	LOC650368	OR56A5
ROS1	SNORD116-5	SQSTM1
SNORD116-3	ZNF649	NT5DC2
EBAG9	B3GNT7	KRT40
AJAP1	KCP	OR10A6
PCDHGA2	LOC653486	P4HA2
CAMKV	SNORD116-6	OR5H6
RPS6KA2	ZSCAN18	C17orf50
SNORD116-4	SP140L	OR51T1
MYOM2	FLJ43663	HSPB3
FAM54B	SNORA45	OR5H2
PCDHGA1	SNORD116-7	SLFN13
LRRC2	MGC2752	OR51A4
RPS10	STK11IP	ATP6V0E1
SNORD116-5	FAM180A	ZXDC
DLGAP2	SNORA54	SLC35G3
CTNNBIP1	SNORD116-8	OR51A2
PCDHGB8P	TSEN34	ATG12
FYCO1	MOGAT1	ZBED2
RPS12	OR2A25	UNC45B
SNORD116-6	INS-IGF2	IFITM10
MSC	SNORD116-9	PTTG1
C1orf63	MBOAT7	CEP97
PCDHB15	AGAP1	CD300LG
CCDC51	OR2A5	MIR210
RPS18	MUC5B	PDLIM7

SNORD116-7	SNORD116-10	HSPBAP1
MFHAS1	FKRP	KIF18B
AGTRAP	TWIST2	KRTAP5-5
PCDHB14	PRRT4	NREP
THOC7	MIR675	C3orf52
RREB1	SNORD116-11	RUNDC1
SNORD116-8	ZSCAN5A	KRTAP5-2
KCNB2	PARD3B	CNOT8
PITHD1	RAB19	QTRTD1
PCDHB13	FAM99B	LOC147093
WDR82	SNORD116-12	KRTAP5-6
RXRB	LENG1	RAB9BP1
SNORD116-9	DIS3L2	CCDC48
RECQL4	OR2A7	WIPF2
MAN1C1	LOC100133161	TMEM41B
PCDHB12	SNORD116-13	HAND1
RTP3	LILRP2	NEK11
VPS52	NEU4	KRT25
SNORD116-10	OR2A20P	LOC440028
CYP7B1	MRPL23-AS1	MED7
NIPAL3	SNORD116-14	THOC7
PCDHB11	LILRA6	TMEM99
ID2B	ICA1L	TRIM6-TRI
ATXN1	LOC401431	HOMER1
SNORD116-11	MIR4298	NAA50
BAG4	SNORD116-15	SLFN5
SEPN1	ZNF329	MIR483
PCDHB10	AP1S3	MYOT
ATRIP	OR2A42	ABTB1
SRSF3	MIR210HG	NAGS
SNORD116-12	SNORD116-16	SNORA3
ENTPD4	TBC1D17	ADAMTS2
TP73-AS1	SGPP2	WDR82
PCDHB9	FLJ45340	FAM134C
NICN1	MIR4686	SNORA52
SGK1	SNORD116-17	SCAMP1
SNORD116-13	ZNF419	OR5AC2
ARHGEF10	ALS2CR12	RHBDL3
PLEKHG5	MIR129-1	LOC644656
PCDHB8	MIR4687	NRG2
MON1A	SNORD116-18	PARP9
SIM1	ISOC2	KRT28
SNORD116-14	SPATA3	LOC650368
MTFR1	MIR153-2	CXCL14
LRRC47	SNORD116-20	ID2B
PCDHB7	MYH14	KRT24
KBTBD8	TMEM198	LOC653486

SKIV2L	MIR182	H2AFY
SNORD116-15	SNORD116-21	ARL6
LRRC14	ZNF665	ZNF385C
PTCHD2	ZFAND2B	SNORA23
PCDHB6	MIR183	SMAD5-AS
ABHD14B	SNORD116-22	CHCHD6
SLC17A1	ZNF552	RDM1
SNORD116-16	CPO	SNORA45
TTC35	MIR29A	RNF14
KIF17	SNORD116-23	C3orf26
PCDHB4	ZNF671	GAS2L2
C3orf39	MDH1B	SNORA54
SLC22A1	MIR29B1	CARTPT
SNORD116-17	SNORD116-24	KBTBD8
RIMS2	ZNF613	C17orf66
HES4	FBXO36	SNORD97
PCDHB3	MIR96	SNCAIP
UCN2	SNORD116-25	SLC12A8
SLC22A3	ZNF702P	C17orf105
SNORD116-18	MTERFD2	INS-IGF2
ST18	LOC407835	TTC37
GRHL3	SNORD115-2	RETNLB
PCDHB2	ZNF606	FAM171A2
ZNF502	UBE2F	MUC5B
SLC22A2	AKR1B15	CLINT1
SNORD116-20	SNORD116-26	CCDC54
TOX	ZNF614	C17orf104
IL22RA1	OTOS	LOC729013
PCDHAC2	CTAGE15P	PCDHGA8
RFT1	SNORD116-27	ABHD14B
SMPD2	FUZ	PIGW
SNORD116-21	MYEOV2	MIR675
MTSS1	OR2A9P	PCDHA9
MIIP	SNORD115-3	MINA
PCDHAC1	ZNF611	C17orf78
ZBTB47	OR6B3	MRVI1-AS1
SNRPC	OR2A2	ZFYVE16
SNORD116-22	SNORD115-4	GPR128
PTDSS1	MED25	LOC28410
CELA2A	FAM117B	FAM99B
PCDHA13	MIR335	MATR3
ACTR8	SNORD115-5	DIRC2
SOD2	BCL2L12	CISD3
SNORD116-23	LOC150935	LOC100133
PHYHIP	ARHGEF35	MAML1
GPATCH3	SNORD115-6	C3orf15
PCDHA12	GRWD1	GSDMA

SLC25A26	PKI55	MRPL23-AS
SOX4	GIMAP6	KIAA0141
SNORD116-24	SNORD115-7	PHLDB2
RB1CC1	ZNF541	KRT42P
PRDM16	C2orf67	MIR4298
PCDHA11	WEE2	JAKMIP2
ZNF501	SNORD115-8	LOC90246
SRF	SYT3	PTRF
SNORD116-25	LOC151171	MIR4299
ZNF623	MIR490	PJA2
PLA2G2F	SNORD115-9	C3orf25
PCDHA10	AKT1S1	KLHL10
GPR62	LOC151174	MTRNR2L8
SRPK1	ZNF862	DDX46
SNORD115-2	SNORD115-10	BOC
KIAA0196	ZNF528	C17orf65
CEP85	METTL21A	MIR210HG
PCDHA8	LOC645249	GFPT2
FAM3D	SNORD115-11	FAM55C
SSR1	BRSK1	MSL1
SNORD116-26	CCNYL1	MIR4686
KBTBD11	LOC646329	SLC23A1
NMNAT1	SNORD115-12	RFT1
PCDHA7	SNORD35B	KRT27
LYZL4	ALS2CR11	MIR4485
ELOVL4	C7orf73	GNPDA1
SNORD116-27	SNORD115-13	ACTR8
HHLA1	ZNF347	SLFN14
VWA1	CCDC140	MIR4687
PCDHA6	ACTR3C	SRA1
KBTBD5	SNORD115-14	TXNRD3
T	ZNF577	FLJ43826
SNORD115-3	SLC23A3	EDIL3
SORBS3	MIR592	OSBPL11
PINK1	SNORD115-15	STAC2
PCDHA5	SUV420H2	COL4A3BP
TMEM42	LOC151300	SLC25A26
TAF11	MIR593	CCL14-CCL1
SNORD115-4	SNORD115-16	RAD50
TRIB1	C19orf48	LRRC58
PRAMEF1	GPBAR1	KRT26
PCDHA4	MIR595	KIF20A
IQCF1	SNORD115-17	GPR62
MAP3K7	ZBTB45	CCL4L2
SNORD115-5	SLC16A14	G3BP1
HRSP12	LOC728377	CCDC58
PRAMEF2	SNORD115-18	C17orf98

PCDHA3	ZNF587	LHFPL2
GLYCTK	LOC151475	FAM3D
TAP1	LOC728743	LOC388387
SNORD115-6	SNORD115-19	CWC27
NPM2	FIZ1	ZPLD1
NADK	LINC00471	CCDC103
PCDHA2	LOC730441	APBB3
PPM1M	SNORD115-20	CD200R1
TAP2	GALP	ARL5C
SNORD115-7	LOC151484	CCNO
DLC1	LUZP6	DCBLD2
PHACTR4	SNORD115-21	KRT39
PCDHA1	SIGLEC10	TNIP1
C3orf49	MSL3P1	TPRA1
TAPBP	MIR671	C17orf102
SNORD115-8	SNORD115-22	GNB2L1
NDRG1	SIGLEC12	UROC1
C1orf135	WDR69	MIR193A
VTRNA1-3	LOC100124692	NSA2
SNTN	SNORD115-23	NUDT16
TBCC	ZNF628	TBC1D3B
SNORD115-9	C2orf57	FST
PGCP	LOC100128264	COL6A6
EFHD2	SNORD115-25	TBC1D3C
VTRNA1-2	KIR3DX1	SLU7
SYNPR	PIKFYVE	FAM172BP
TBP	CTAGE4	CCL3L3
SNORD115-10	SNORD115-26	POLR3G
SPAG11B	ZNF551	IQCF1
MMEL1	LOC200726	LOC440434
VTRNA1-1	LOC100128822	RGS14
C3orf45	SNORD115-29	GLYCTK
TCF19	ZNF616	GPR179
SNORD115-11	TIGD1	PLK2
LYPLA1	LOC100129148	PPM1M
RSG1	SNORD115-30	FBXO47
NMUR2	ZNF766	IQGAP2
ASB14	LOC200772	C3orf49
TCF21	LOC100130705	ARGFXP2
SNORD115-12	SNORD115-31	SEC24A
NCOA2	ZNF468	SNTN
OR4F5	CCDC108	SNORA21
FEM1C	LOC100130880	CPLX2
FAM19A4	SNORD115-32	SYNPR
TCP1	ZNF160	KRTAP4-11
SNORD115-13	C2orf72	C5orf4
ARFGEF1	LOC100131176	RPL32P3

MUL1	SNORD115-33	TBC1D3G
C5orf15	CTU1	FAM114A2
CCDC12	CXXC11	H1FOO
TCP10	LOC100132707	SNORD7
SNORD115-14	SNORD115-34	BRD8
COLEC10	ZNF835	ASB14
NOL9	UNC80	MIR632
CDC42SE2	LOC100134229	TCERG1
PPP4R2	SNORD115-35	DTX3L
TCP11	ZNF765	KRTAP4-8
SNORD115-15	RUFY4	BTNL3
KHDRBS3	LOC100134713	FAM19A4
LIN28A	SNORD115-36	KRTAP2-2
TRPC7	NLRP12	HNRNPA0
CCDC13	DUSP28	LOC151658
TCTE3	LOC100287482	KRTAP9-1
SNORD115-16	SNORD115-37	LMAN2
DCTN6	MYADM	DPPA2
AGMAT	ESPNL	TBC1D3
ZNF608	MOXD2P	SPINK5
XIRP1	SNORD115-38	CCDC80
PPP1R11	ZNF845	TBC1D3H
SNORD115-17	ECEL1P2	SMA4
PNMA2	ZNF783	BTLA
LINC00115	SNORD115-39	LOC730755
KCTD16	CCDC114	SMA5
PRICKLE2	LOC348761	PPP4R2
DYNLT1	MIR548F3	SNORD124
SNORD115-18	SNORD115-40	SOX30
ADAM28	ACPT	ROPN1B
MORN1	C2orf62	MIR365B
SEMA6A	MIR548I4	ESM1
TTC21A	SNORD115-41	C3orf22
TEAD3	CGB5	LOC10013C
SNORD115-19	RBM44	KIF3A
POP1	MIR548F4	SPICE1
FAM110D	SNORD115-42	LOC100131
PCDHB16	CGB7	ADAMTS6
C3orf67	AQP12A	NUDT16P1
TFAP2A	MIR548T	KRTAP4-9
SNORD115-20	SNORD115-43	MGAT4B
AP3M2	LRRC4B	LOC152225
DHDDS	KLHL30	KRTAP4-7
HMHB1	MTRNR2L6	B4GALT7
KCTD6	SNORD115-44	IGSF11
TFAP2B	LENG9	C17orf96
SNORD115-21	PLEKHM3	SYNPO

COPS5	MIR3907	C3orf30
GPR157	SNORD116-28	LOC10019C
EPB41L4A	CGB8	RHOBTB3
KLHDC8B	C2orf80	PARP15
THBS2	LOC100505483	MIR2117
SNORD115-22	SNORD116-29	RNF44
RBPMS	GNG8	DNAJB8
SPSB1	RESP18	MIR2909
ERAP2	LOC100506585	ABLIM3
LOC201617	SNORD115-48	GPR156
NR2E1	BIRC8	LOC100505
SNORD115-23	C2orf82	ELL2
WWP1	LOC100507421	CHST13
GLTPD1	SNORD115-24	LRRC3C
SIL1	FAM71E1	SV2C
DNAH12	OR6B2	PRICKLE2
TNF	GIMAP1-GIMAP5	KRTAP16-1
SNORD115-25	SNORD115-27	HMGXB3
STMN2	RDH13	KBTBD12
ZNF436	DYTN	LOC100505
ARAP3	MIR4468	TBC1D9B
PDE12	SNORD115-28	ALG1L
TNFAIP3	PTH2	AA06
SNORD115-26	ASB18	ARHGAP26
PTP4A3	SNORD115-45	C3orf67
TAS1R2	ZIM3	SLFN12L
FBXL17	FLJ43879	FSTL4
FAM116A	SNORD115-47	KCTD6
TNXA	SIGLEC11	RAD51L3-R
SNORD115-29	VWC2L	MRPS27
ERLIN2	MIR147B	ARL13B
TAS1R1	CGB1	MIR4728
YTHDC2	MIR149	ATP10B
ZNF620	LOC100128714	DHFRL1
TNXB	CGB2	MIR4726
SNORD115-30	MIR153-1	N4BP3
LZTS1	LOC100131089	GABRR3
OR4F16	LMTK3	MIR4734
GRAMD3	MIR26B	SEPT8
ALS2CL	ANKRD63	PTPLB
TPBG	LENG8	MIR4724
SNORD115-31	DNAJB3	FAF2
PROSC	HERC2P7	LOC201617
ACTL8	KIR3DL3	MIR4727
PCYOX1L	MIR375	PPIP5K2
TMIE	GOLGA8F	DNAH12
TPD52L1	ZNF837	MIR4725

SNORD115-32	SNORA41	WWC1
RNF139	GOLGA8DP	PDE12
CCNL2	CLDND2	FBXW11
SH3TC2	LOC643387	FAM116A
C3orf64	JMJD7	ACSL6
TPMT	ZNF816	TIGIT
SNORD115-33	PRR21	PHF15
ZHX1	PLA2G4B	KIAA2018
SH3BGRL3	ZNF543	LARP1
SAP30L	LOC646324	CADM2
FBXW12	ULK4P2	PPWD1
CRISP2	COX6B2	ZDHHC23
SNORD115-34	LOC646736	HARS2
PUF60	LOC100288615	LOC255025
SESN2	OSCAR	OTP
TXNDC15	PRSS56	NUP210P1
ENTPD3-AS1	LOC100288637	SKIV2L2
TSPYL1	ZNF813	COL6A5
SNORD115-35	AQP12B	ZNF346
ZHX2	LOC100289656	PLCXD2
ESPN	JOSD2	SSBP2
MCTP1	SNORA75	GCET2
ZNF619	MIR1233-1	BHMT2
TTK	IZUMO2	LSAMP-AS3
SNORD115-36	SCARNA6	TNFAIP8
DENND3	MIR1282	C3orf64
TAS1R3	CPT1C	PART1
NDFIP1	SCARNA5	LOC285205
ZNF621	MIR1233-2	GEMIN5
TUBB2A	ALDH16A1	EPHA6
SNORD115-37	SNORD11	PCDHGA12
XPO7	MIR4310	DNAJB8-AS
ATAD3B	NTN5	LRRTM2
FBXO38	SNORD70	C3orf38
CCDC66	MIR3942	FAM169A
TULP1	NLRP13	RABL3
SNORD115-38	D2HGDH	CCDC69
TRIM35	LOC100505648	CCDC66
TMEM222	NLRP8	PCDHB5
YIPF5	LOC728323	SLC9A10
C3orf23	LOC100507466	FBXL21
UTRN	NLRP5	LOC285359
SNORD115-39	DIRC3	KLHL3
ZC3H3	SERF2-C15ORF63	LOC285401
PLEKHN1	ZNF787	TSPAN17
TIGD6	SNORD11B	ILDR1
ZNF660	MIR4509-1	OR4F3

VARS	ZFP28	EIF4E3
SNORD115-40	LOC100129175	HAVCR1
EFR3A	MIR4509-2	RAB43
USP48	VSIG10L	SNORD63
SPRY4	PP14571	LOC339874
FLJ39534	MIR4508	SNORA74A
VEGFA	LOC147646	H1FX-AS1
SNORD115-41	LOC100130451	RNU5E-1
RRS1	MIR4510	LOC344595
NBPF3	LINC00085	RNU5D-1
NUDT12	LOC100286922	TMPRSS7
LOC285401	MIR4509-3	CYFIP2
EZR	ZNF480	CD200R1L
SNORD115-42	MIR1471	PKD2L2
SULF1	MIR4715	LNP1
ZDHHC18	ZNF534	UQCRQ
SLC4A9	MIR1244-1	CCDC37
EIF4E3	TMCO5B	AFF4
VIP	ZNF578	SPATA12
SNORD115-43	MIR548F2	PRELID1
RHOBTB2	ERVV-1	TMEM110
SLC25A33	LOC100329109	IL17B
SLC25A2	LOC147670	IQCF2
CCDC36	BOK-AS1	DIMT1
ZNF76	C19orf18	IQCF5
SNORD115-44	MIR1244-3	MAT2B
ARC	ZNF418	MUSTN1
DDI2	MIR1244-2	SLC27A6
SPATA9	ZNF417	VGLL3
PRSS42	MIR3131	SNX24
ZNF165	ZNF548	IQCF3
SNORD116-28	MIR4268	MRPL22
BOP1	TMEM190	LOC401074
LZIC	MIR3130-1	PCDHB1
TSSK1B	TMC4	FLJ22763
ZNF445	MIR3130-2	DMGDH
TRIM26	LOC147804	FLJ25363
SNORD116-29	MIR2355	TLX3
DDHD2	ZNF524	OR5K2
TRIM63	MIR3132	KCNIP1
PCDHB19P	ZNF784	OR5H14
SPATA12	MIR4269	ZNF354C
ZNF184	CCDC155	OR5H15
SNORD115-48	LOC100507443	TMED7
KIF13B	DACT3	OR5K3
C1orf170	UBE2F-SCLY	ISOC1
PCBD2	NLRP4	OR5K4

C3orf77	MIR4439	RPL26L1
ZNF187	ZNF542	MIRLET7G
SNORD115-24	MIR4776-1	SAR1B
PSD3	ZNF582	MIR135A1
CROCCP2	MIR4777	C5orf45
PSD2	ZNF583	MIR198
C3orf62	MIR2467	DCTN4
ZNF192	CDC42EP5	FAM19A1
SNORD115-27	MIR4775	IPO11
ZFPM2	ZNF836	STX19
SYTL1	MIR4440	MZB1
ANKRD32	ZNF610	IQCF6
TMEM110	MIR4786	PAIP2
ZNF193	ZNF600	C3orf78
SNORD115-28	MIR4776-2	CDKL3
HEY1	ZNF320	LOC440970
IGSF21	MIR4441	PCDH12
C5orf32	ZNF497	ARGFX
PRSS45	ZNF550	GCNT4
ZNF204P	ZNF579	TMEM30C
SNORD115-45	ZNF114	FAM13B
TRAM1	ZNF525	ALG1L2
SNHG12	SPACA4	FAM53C
MEGF10	NLRP7	TXNRD3NB
AMIGO3	C19orf76	REEP2
PTP4A1	ZNF584	COL6A4P2
SNORD115-47	ZSCAN4	PRR16
LEPROTL1	NLRP11	FLJ20518
KIAA1751	TMEM86B	COMMD10
SPINK7	PRR24	LOC653712
ZNF662	ZNF549	POLK
DEK	NAPSB	LINC00488
MIR147B	IL4I1	DDX41
SCRIB	SSC5D	SNORA7B
KIAA2013	ZNF547	NOP16
HAVCR2	ZIK1	SNORD19
CDHR4	ZNF776	LARS
ALDH5A1	ZSCAN1	FAM86DP
LOC100128714	TPRX1	CXXC5
KIAA0146	MAMSTR	SNORD69
THAP3	IZUMO1	HMP19
C5orf62	C19orf63	MIR548A2
FAM212A	MGC45922	UIMC1
PRRC2A	KLK9	MIR548A3
LOC100131089	SIGLECP3	RAPGEF6
SLC39A14	C19orf75	MIR567
C1orf201	ZNF615	ERAP1

TSLP	ZNF841	MIR568
IQCF2	LOC284379	KDM3B
BAG6	VSTM1	GXYLT2
ANKRD63	TMEM150B	PHAX
LRRC6	FAM71E2	FAM86HP
UBXN11	VN1R2	RAB24
FCHSD1	VN1R4	ESRG
IQCF5	NLRP9	PELO
GPANK1	C19orf81	LOC10000[illegible]
HERC2P7	ZNF677	RBM27
LY96	RFPL4A	SNORD19B
C1orf158	ZSCAN5B	NEURL1B
C5orf30	ZSCAN22	LOC100125
MUSTN1	ZNF530	DHX29
DDX39B	C19orf51	C3orf74
GOLGA8F	LILRA5	FLJ11235
SGK3	ZNF773	LOC10012[illegible]
FBXO44	LOC386758	DDX4
LYRM7	ZNF808	ZBTB20-AS[illegible]
IQCF3	ZNF761	FAM193B
ABHD16A	TMEM238	ZNF717
GOLGA8DP	ZNF470	SGTB
RAD54B	ZNF749	LOC100287
ATPIF1	ZNF324B	ARL15
SLC25A46	ZNF805	FRG2C
LOC401074	ZNF321P	PCDHB18
SLC39A7	SIGLEC16	MIR1284
JMJD7	FLJ26850	PCDHB17
DCAF13	ZNF880	MIR1280
CROCCP3	ZNF772	TMED9
CDKN2AIPNL	IGLON5	MIR548I1
MIRLET7G	MIRLET7E	ZCCHC10
HSD17B8	MIR125A	MIR1324
PLA2G4B	MIR150	CDHR2
RNF19A	MIR99B	LOC100302
FHAD1	TARM1	GIN1
TIMD4	MIR371A	MIR548G
MIR135A1	MIR372	WDR55
OR2H2	MIR373	MIR3136
ULK4P2	A1BG-AS1	ANKHD1
GPR124	DPRX	MIR4272
LOC115110	DUXA	CCDC99
ZNF300	ASPDH	MIR4273
MIR138-1	MIR512-1	THG1L
RDBP	MIR512-2	MIR3921
LOC100288615	MIR498	AGGF1
KIAA1429	MIR520E	MIR3938

RAB42	MIR515-1	WDR41
MYOZ3	MIR519E	PVRL3-AS1
MIR191	MIR520F	TMCO6
LST1	MIR515-2	IGSF11-AS1
LOC100288637	MIR519C	TRIM36
C8orf71	MIR520A	MYLK-AS1
FAM46B	MIR526B	GALNT10
PRDM6	MIR519B	IQCF4
FAM19A1	MIR525	NHP2
PLA2G7	MIR523	LOC100506
LOC100289656	MIR518F	RBM22
RGS22	MIR520B	LOC100507
RBP7	MIR518B	RIOK2
FTMT	MIR526A1	LOC100507
FLJ33065	MIR520C	DEPDC1B
TFEB	MIR518C	ADAMTS9-
MIR1233-1	MIR524	BDP1
FBXL6	MIR517A	ABHD14A-
ACAP3	MIR519D	RNF130
FNIP1	MIR521-2	TMEM110-
TMEM89	MIR520D	ERBB2IP
STL	MIR517B	ISY1-RAB43
MIR1282	MIR520G	PCDHGC5
PTTG3P	MIR516B2	MIR4787
UBE2J2	MIR526A2	PCDHGC4
SLC35A4	MIR518E	MIR4795
IQCF6	MIR518A1	PCDHGB7
EPM2A	MIR518D	MIR4796
MIR1233-2	MIR516B1	PCDHGB6
FBXO25	MIR518A2	MIR4444-1
C1orf172	MIR517C	PCDHGB5
EPB41L4A-AS1	MIR520H	MIR4446
C3orf78	MIR521-1	PCDHGB3
HIST1H4I	MIR522	LOC100652
MIR4310	MIR519A1	PCDHGB2
FGF20	MIR527	PCDHGB1
LRRC38	MIR516A1	PCDHGA11
MARCH3	MIR516A2	PCDHGA10
MIR425	MIR519A2	PCDHGA9
HIST1H2AI	KLKP1	PCDHGA7
MIR3942	LOC646508	PCDHGA6
SNORA72	SBK2	PCDHGA5
AADACL3	LOC646862	PCDHGA4
LEAP2	SEC1	PCDHGA3
SNORA6	SNORD23	PCDHGA2
HIST1H2AK	SNORD88A	PCDHGA1
LOC100505648	SNORD88B	PCDHGB8P

SNORD54	SNORD88C	PCDHB15
PUSL1	MIR643	PCDHB14
SCGB3A2	RPL13AP5	PCDHB13
LOC644714	CEACAM18	PCDHB12
HIST1H2AJ	SHISA7	PCDHB11
LOC100507466	FLJ30403	PCDHB10
OPLAH	ZNF814	PCDHB9
B3GALT6	SIGLEC14	PCDHB8
SLCO6A1	MIMT1	PCDHB7
SPINK8	MIR935	PCDHB6
HIST1H2AL	SNAR-G1	PCDHB4
SERF2-C15ORF63	SNAR-F	PCDHB3
PABPC1	SNAR-A1	PCDHB2
IFFO2	SNAR-A2	PCDHAC2
PPARGC1B	SNAR-A12	PCDHAC1
C3orf71	LOC100128252	PCDHA13
HIST1H2AC	LOC100128398	PCDHA12
MIR4509-1	LOC100129083	PCDHA11
KCNV1	SGK110	PCDHA10
TPRG1L	BSPH1	PCDHA8
PRRC1	LOC100131691	PCDHA7
LOC646498	PEG3-AS1	PCDHA6
HIST1H2AB	SNAR-A3	PCDHA5
MIR4509-2	SNAR-A5	PCDHA4
STAU2	SNAR-A7	PCDHA3
FAM213B	SNAR-A11	PCDHA2
ZNF474	SNAR-A9	PCDHA1
FLJ20518	SNAR-A4	VTRNA1-3
HIST1H2AM	SNAR-A6	VTRNA1-2
MIR4508	SNAR-A8	VTRNA1-1
MTBP	SNAR-A13	NMUR2
MYOM3	SNAR-A10	FEM1C
POU5F2	SNAR-B2	C5orf15
SNORD19	SNAR-C2	CDC42SE2
HIST1H2BG	SNAR-C4	TRPC7
MIR4510	SNAR-E	KIAA1191
DKK4	SNAR-C5	ERGIC1
KLHDC7A	SNAR-B1	CLK4
AFAP1L1	SNAR-C1	ODZ2
FAM86DP	SNAR-C3	CNOT6
HIST1H2BL	SNAR-D	NLN
MIR4509-3	SNAR-G2	ZNF608
EIF2C2	SNAR-A14	KCTD16
VWA5B1	ERVV-2	SEMA6A
GRPEL2	ZNF587B	ARRDC3
SNORD69	MIR1283-2	ZSWIM6
HIST1H2BN	MIR1323	PCDHB16

MIR4715	MIR1283-1	ANKRA2
LSM1	MIR3191	HMHB1
UBXN10	MIR3190	C5orf54
LSM11	MIR320E	MCCC2
MIR548A2	MIR4324	EPB41L4A
HIST1H2BM	LOC100505681	CENPK
TMCO5B	LOC100505812	ERAP2
ADAMDEC1	LOC100506012	RGNEF
ARHGEF19	LOC100506033	NSD1
GPR151	LOC100506068	SIL1
MIR564	LOC100507003	GMCL1P1
HIST1H2BF	ZNF865	ARAP3
BHLHE22	ZNF816-ZNF321P	RMND5B
ACTRT2	MIR4754	FBXL17
STARD4	MIR4752	YTHDC2
GXYLT2	MIR371B	RANBP17
HIST1H2BE	MIR4749	SLC30A5
MRPS28	MIR4750	CENPH
MIB2	MIR4751	GPBP1
WDR36		GRAMD3
FAM198A		PCYOX1L
HIST1H2BH		CCNJL
COMMD5		SH3TC2
C1orf127		PANK3
ZNF300P1		PARP8
CCR2		SAP30L
HIST1H2BI		ANKRD55
MRPL13		TXNDC15
SAMD11		MCTP1
SOWAHA		PTCD2
ESRG		BTNL8
HIST1H2BC		DOK3
ATAD2		ELOVL7
LOC148413		C5orf44
SHROOM1		ZFP2
SNORD19B		RUFY1
HIST1H2BO		CPEB4
ASAP1-IT1		PRR7
PHF13		NDFIP1
C5orf24		OR4F16
C3orf74		FBXO38
HIST1H3A		YIPF5
MRPL15		TRIM7
CCDC27		TIGD6
C5orf20		SPRY4
LOC100128640		MXD3
HIST1H3D		NUDT12

CNOT7
C1orf213
SLC36A2
NRADDP
HIST1H3C
CPSF1
PDIK1L
SPINK13
ZNF717
HIST1H3E
PURG
C1orf64
CEP120
LOC100132146
HIST1H3I
LRP12
SLC2A7
SLC25A48
BSN-AS2
HIST1H3G
RRM2B
CALML6
SRFBP1
PRSS46
HIST1H3J
CYHR1
IL28RA
LOC153469
LOC100287879
HIST1H3H
ASAP1
FAM43B
ZMAT2
FRG2C
HIST1H3B
MTERFD1
PAQR7
ARSK
MIR1284
HIST1H4A
PI15
FAM76A
FAM81B
MIR1324
HIST1H4D
FAM135B
TMEM201

SLC4A9
ATG10
SLC25A2
SPATA9
TSSK1B
FAM172A
PCDHB19P
GPR98
PCBD2
UTP15
ZCCHC9
PSD2
ANKRD32
THOC3
ZBED3
GFM2
C5orf32
MEGF10
SPINK7
SPZ1
TRIM52
HAVCR2
AGXT2L2
C5orf62
TSLP
FCHSD1
UNC5A
C5orf30
LYRM7
TRIM41
SLC25A46
BOD1
CDKN2AIPN
COL23A1
TIMD4
NDUFAF2
ZNF300
MYOZ3
UBTD2
MRPS36
ATP6AP1L
SCGB3A1
PRDM6
FTMT
SFXN1
FNIP1
SNX18

CCDC112	SLC35A4
MIR1226	GPRIN1
HIST1H4F	PWWP2A
ZC2HC1A	C1QTNF2
C1orf86	EPB41L4A-[illegible]
PPP1R2P3	MARCH3
MIR711	FCHO2
HIST1H4K	RAB3C
PHF20L1	LYSMD3
C1orf126	LEAP2
FAM71B	SCGB3A2
MIR3136	ZNF354B
HIST1H4J	C5orf35
LACTB2	IL31RA
ATAD3C	EMB
PRELID2	SLCO6A1
MIR4272	C5orf47
HIST1H4C	PPARGC1B
FAM82B	PRRC1
AKR7L	JMY
SH3RF2	C5orf58
MIR4271	ZNF474
HIST1H4H	OR2Y1
ZNF706	POU5F2
LOC254099	AFAP1L1
PLAC8L1	GRPEL2
MIR4273	TMEM171
HIST1H4B	TMEM174
GOLGA7	LSM11
TTLL10	POC5
DCP2	GPR151
MIR3938	STARD4
HIST1H4E	WDR36
VPS28	ZNF300P1
TMCO4	NUDCD2
LIX1	UBLCP1
KRBOX1	ACOT12
HIST1H4L	SOWAHA
ZDHHC2	SHROOM1
ZNF683	C5orf24
ADAMTS19	SREK1
IQCF4	C5orf20
HIST1H4G	SLC38A9
FAM203A	SLC36A2
MDS2	SPINK13
SPATA24	C5orf41
LOC100506994	CEP120

STX7	SLC25A48
KCNK9	TMEM167A
NPHP4	MBLAC2
DNAJC18	TMEM161B
LOC100507062	SRFBP1
CMAHP	LOC153469
SLC25A37	ZMAT2
FAM41C	MARVELD2
C5orf27	BTNL9
ADAMTS9-AS2	ARSK
DHX16	FAM81B
C8orf55	CCDC112
LOC284632	PPP1R2P3
STK32A	FAM71B
ABHD14A-ACY1	PRELID2
SUPT3H	SH3RF2
UBR5	PLAC8L1
LOC284661	RNF145
AQPEP	MIER3
TMEM110-MUSTN1	CDC20B
PEX3	PAPD4
SCARA3	DCP2
SLC25A34	LIX1
SLC36A1	ZNF366
MIR4793	FAM151B
GCM1	S100Z
FAM49B	ADAMTS19
ESPNP	HIGD2A
KIAA0825	SPATA24
MIR4787	DNAJC18
DDO	FAM153B
AZIN1	LOC202181
C1orf174	CCDC125
GPR150	C5orf27
MIR4444-1	GAPT
RNASET2	CMYA5
ATP6V1H	STK32A
KLHL17	AQPEP
DTWD2	SLC36A1
MIR4443	EIF4E1B
KCNK5	RASGEF1C
OTUD6B	ANKRD31
TMEM240	SERINC5
RELL2	LOC257358
LOC100652759	LOC257396
STX11	EFCAB9
TMEM66	SH3PXD2B

TMEM52	LOC285593
SLC36A3	FAM153A
LUST	ARL10
B3GALT4	KIAA0825
CHRAC1	GPR150
LOC339505	DTWD2
KIF4B	RELL2
SNX3	NBPF22P
SNTG1	LOC285626
AADACL4	LOC285627
RGMB	LOC285629
RNGTT	SLC36A3
GDAP1	KIF4B
PRAMEF5	OR2V2
RFESD	C5orf64
CD164	RNF180
EXOSC4	SREK1IP1
HNRNPCL1	ZNF454
CHSY3	C5orf60
SYNGAP1	RGMB
PDP1	RFESD
PRAMEF9	CHSY3
TMEM173	LOC340037
WISP3	TMEM173
CNGB3	FAM170A
PRAMEF10	LOC340073
FAM170A	LOC340074
STK19	ARSI
LY6K	ANKRD34B
FAM131C	PFN3
LOC340073	ZNF879
IER3	IRGM
KCTD9	FBLL1
PADI6	IDAS
LOC340074	ACTBL2
SYNJ2	FAM174A
ESRP1	MTX3
C1orf187	CATSPER3
ARSI	NIPAL4
VNN2	HCN1
WHSC1L1	SLCO4C1
SPATA21	TICAM2
IRGM	DND1
VNN1	MAST4
IMPAD1	C5orf25
AGRN	RNF138P1
FAM174A	FLJ38109

PRPF4B	C5orf48
TMEM70	LOC389332
APITD1	C5orf65
CATSPER3	C5orf46
WASF1	ARHGEF37
PINX1	RGS7BP
CATSPER4	C5orf63
NIPAL4	SPINK6
HIST1H3F	ANKHD1-EI
TRMT12	MIR103A1
GPR153	MIR143
SLCO4C1	MIR145
HIST1H2AG	MIR146A
OXR1	MIR218-2
FAM132A	MIR9-2
TICAM2	SPINK14
HIST1H2BJ	FNDC9
WDYHV1	CRSP8P
HES5	FLJ42709
DND1	C5orf56
RNF8	FLJ16171
PIWIL2	MIR340
LOC388588	IGIP
FLJ38109	GPX8
TAAR5	TIFAB
ELP3	FLJ35946
RNF207	LOC553103
C5orf48	MIR449A
MAP7	CTXN3
THAP1	SNORD95
TMEM82	SNORD96A
LOC389332	ECSCR
TBX18	LOC642366
ARMC1	TMEM232
TRNP1	C5orf43
C5orf65	LOC643201
LATS1	GRXCR2
INTS10	SPINK9
CD164L2	ZFP62
C5orf46	LOC644100
GCM2	FLJ33630
CCDC25	LOC644936
HES3	CCNI2
ARHGEF37	LINC00461
WDR46	CBY3
UBE2W	LOC647859
PRAMEF12	GUSBP3

C5orf63	GTF2H2B
ZBTB22	FAM153C
BRF2	SNORA13
PRAMEF21	SCARNA18
SPINK6	SNORA47
TAAR2	SNORA74B
AGPAT5	MIR449B
PRAMEF8	MIR581
ANKHD1-EIF4EBP3	MIR583
TAAR3	MIR585
LAPTM4B	SNHG4
PRAMEF18	MIR143HG
MIR143	GTF2H2C
CD83	LOC728342
C8orf39	SERF1B
PRAMEF17	LOC728554
MIR145	LOC728723
HMGN3	LOC729080
TMEM55A	AACSP1
PLA2G2C	LOC729678
SPINK14	OR4F29
PPT2	GTF2H2D
SLC39A4	GUSBP9
TMEM200B	VTRNA2-1
FNDC9	MIR874
CDYL	LOC100129
CHD7	LRRC70
PRAMEF4	LOC100131
C5orf56	FAM196B
NCR2	LOC100132
SYBU	LOC100132
PRAMEF13	FAM159B
IGIP	LOC100133
MED23	LOC100133
INTS8	LOC100170
SH2D5	C5orf52
TIFAB	LOC100268
QKI	LOC100272
INTS9	LOC100289
C1orf130	LOC100289
FLJ35946	MIR1289-2
LY86	MIR1229
CSGALNACT1	MIR103B1
RNF223	MIR1244-1
LOC553103	TMED7-TIC
FHL5	NCRUPAR
HR	LOC100303

PRAMEF3
CTXN3
AKAP7
PAG1
LDLRAD2
ECSCR
ATG5
PBK
MIR200A
TMEM232
MED20
ZNF395
MIR200B
GRXCR2
SLC25A27
DEFB103B
MIR34A
SPINK9
TBPL1
BIN3
FLJ42875
LOC644100
EEF1E1
TEX15
PRAMEF11
FLJ33630
BAG2
GSDMC
PRAMEF6
CCNI2
POLR1C
C8orf44
LOC440563
SNORA13
MAD2L1BP
JPH1
UQCRHL
MIR583
WTAP
C8orf4
MINOS1
SNHG4
AKAP12
ENY2
ANKRD65
MIR143HG
MDC1

MIR2277
MIR449C
MIR1244-3
MIR1244-2
MIR4280
MIR3142
MIR3141
MIR4281
MTRNR2L2
MIR3607
MIR3655
MIR3660
MIR3912
MIR3936
MIR3661
LOC100505
LOC100505
LOC100505
LOC100505
OCLN
LOC100507
MIR4804
MIR4633
MIR4634
MIR4461
MIR3977
MIR4460
MIR4638
MIR4803
MIR378E
LOC100859

CPA6
PRAMEF7
LOC728342
TRAM2
SLURP1
MIR429
LOC729080
KIAA0408
SLC45A4
FAM138F
VTRNA2-1
PHACTR2
MTUS1
LOC643837
MIR874
FAM65B
KIAA1456
TMEM88B
LOC100133050
ZSCAN12
ZFAT
C1orf233
C5orf52
BCLAF1
KIAA1967
LOC644961
LOC100289230
CUL7
ZNF250
C1orf200
LOC100289673
ZBTB24
PLEKHA2
PRAMEF19
MIR1289-2
KIAA0319
SH2D4A
PRAMEF20
MIR1244-1
SNAP91
PRDM14
FAM138A
TMED7-TICAM2
FIG4
SNX16
LOC646471
MIR1244-3

NUP153
NECAB1
LOC649330
MIR1244-2
CASP8AP2
PDLIM2
LOC653566
MIR3141
RANBP9
SOX17
PRAMEF22
MIR3655
SLC17A4
CSMD1
PRAMEF15
MIR3936
UST
EBF2
WASH7P
MIR3661
TRIM10
FAM160B2
PRAMEF16
LOC100505658
FLOT1
DEPTOR
SCARNA1
LOC100505678
RCAN2
PYCRL
SNORA44
LOC100505841
SLC17A2
C8orf33
SNORA61
MIR4633
HCG9
ZBTB10
SNORA59B
MIR4461
PRSS16
LYNX1
SNORA59A
MIR4460
CRISP3
MTMR9
SNORA16A

TRDN
DUSP26
SNORD85
CITED2
C8orf51
SNORD99
BTN3A3
DSCC1
SNORD103A
BTN2A2
DERL1
SNORD103B
ECI2
CHCHD7
MIR551A
PFDN6
GPR172A
CDK11A
HMGN4
HMBOX1
SLC35E2B
TRIM38
EFCAB1
LOC728716
CAP2
MCPH1
LOC729059
SYNCRIP
PPP1R3B
PRAMEF14
UBD
PLEKHF2
FLJ37453
AGPAT1
ZMAT4
LOC729737
SLC35A1
ZFAND1
OR4F29
SCGN
ZFHX4
LOC100129196
C6orf108
GSDMD
LOC100129534
C6orf10
NIPAL2

LOC100130417
FARS2
RNF122
LOC100132062
FUT9
CSPP1
LOC100132287
CNPY3
BAALC
LOC100133331
TRAF3IP2
NUDT18
LOC100133445
HBS1L
ZNF696
LOC100133612
SLC17A3
GRHL2
DDX11L1
RPP40
DOCK5
TTC34
FRS3
FLJ14107
LOC100288069
PDE10A
VCPIP1
MIR1976
SLC22A7
ZNF703
NPPA-AS1
HCP5
TTI2
MIR3115
EHMT2
RAB11FIP1
MIR4253
SMPDL3A
PREX2
MIR4251
APOBEC2
REEP4
MIR4252
PNRC1
ARHGAP39
MIR3917
ASCC3

ZNF34
MIR3675
RAB32
SLC25A32
ENO1-AS1
TRIM31
TM7SF4
LOC100506730
KATNA1
STMN4
LOC100506801
FGFR1OP
RNF170
LOC100506963
BTN3A2
SLCO5A1
APITD1-CORT
BTN3A1
SHARPIN
C1orf151-NBL1
BTN2A1
EPPK1
MIR4695
CAPN11
SCRT1
MIR4420
BVES
SOX7
MIR4684
NUDT3
FAM167A
MIR4689
SEC63
SLC35G5
MIR4632
NRM
LINC00208
MIR4417
STK38
C8orf12
MIR378F
SCAF8
CRISPLD1
RCAN3AS
KIAA1009
TRAPPC9
ICK

TM2D2
ENPP4
TATDN1
ANKRD6
NACAP1
RIMS1
NCALD
DOPEY1
SGK196
ZNF292
MAF1
FTSJD2
UTP23
CDK19
GINS4
CUL9
HOOK3
TAB2
PPAPDC1B
MDN1
MAK16
TSPYL4
TRIM55
ANKS1A
FUT10
UBR2
PARP10
SASH1
C8orf76
SYNE1
TIGD5
UFL1
NUDCD1
SIRT5
FAM83A
PHF3
PPP1R16A
HEY2
FAM86B1
DAAM2
LRRCC1
KIAA0240
TSPYL5
DDAH2
DNAJC5B
HEBP2
PSKH2

ORC3
FAM110B
CD2AP
MED30
BRD7P3
ERI1
MTCH1
ZNF251
ZNF318
KIFC2
SPDEF
TMEM67
MTO1
LONRF1
ASF1A
CHMP7
YIPF3
MTDH
USP49
CHMP4C
CCDC28A
PKHD1L1
MTHFD1L
NAPRT1
PNISR
WDR67
IBTK
HPYR1
MOXD1
RP1L1
IPCEF1
TP53INP1
ZNF451
TGS1
SENP6
MFSD3
OR2B6
MAL2
TIAM2
XKR4
FBXL4
CSMD3
C6orf123
RHPN1
FAM50B
FBXO32
FBXO9

C8orf40
FBXO5
SLC26A7
SLC17A5
PCMTD1
OR12D2
CTHRC1
OR11A1
OSR2
RGS17
C8orf34
OR2W1
TOP1MT
OR2J2
CLDN23
OR2H1
ZNF572
SNORD52
GOT1L1
SNORD50A
FAM92A1
SNORD48
VPS37A
PDE7B
C8orf38
FILIP1
TMEM68
BRPF3
ABRA
GNMT
LYPD2
TNFRSF21
NKX2-6
SESN1
TMEM71
TINAG
SGCZ
DLL1
ADHFE1
OSTM1
UBXN2B
MRPS18B
PXDNL
TMEM14A
AGPAT6
MRPL18
UNC5D

NDUFAF4
LETM2
C6orf15
DCAF4L2
MYLIP
RALYL
ABT1
HGSNAT
DSE
DEFB104A
PRICKLE4
LOC157273
PACSIN1
SGK223
ZNRD1
PEBP4
CLDN20
CDCA2
NOX3
TMEM65
DEF6
LOC157381
C6orf48
RDH10
SLC35B3
C8orf56
HDDC2
ANKRD46
GMNN
ESCO2
MRPL2
FBXO16
TFB1M
LOC157627
CYB5R4
FAM84B
TUBE1
C8orf37
C6orf203
VPS13B
TBC1D7
C8orf42
NRN1
ERICH1
CYP39A1
SLC7A13
CDC40

TDH
RWDD1
TMEM74
AIG1
FAM91A1
NOL7
C8orf48
SNX9
C8orf45
FAM8A1
CLVS1
UBE2J1
NKX6-3
DCDC2
KCNU1
ETV7
C8orf84
TMEM14C
CNBD1
VTA1
SLC30A8
LGSN
COL22A1
TDP2
SNX31
CUTA
TMEM64
PPIL1
ZNF596
BRP44L
IDO2
HECA
DEFT1P
RAB23
SDR16C5
COQ3
ADCK5
CLIC5
TSNARE1
IL20RA
R3HCC1
TREM2
PRSS55
TREM1
C8orf74
UNC93A
HTRA4

HCG4
ADAM32
GFOD1
C8orf47
HMGCLL1
LGI3
MTRF1L
MAPK15
CCHCR1
DEFB105A
GTPBP2
DEFB106A
BTN2A3P
DEFB107A
AHI1
DEFB109P1
UHRF1BP1
DEFB130
ELOVL2
ATP6V0D2
CDKAL1
NEIL2
PAK1IP1
YTHDF3
RMND1
C8orf46
PHIP
LOC254896
SOBP
REXO1L1
AKIRIN2
ADAM5P
CENPQ
FLJ10661
MRPS18A
XKR6
MRPS10
NSMCE2
LRRC1
LOC286059
PHF10
ZNF707
QRSL1
BREA2
VNN3
FAM83H
TMEM63B

LOC286083
AKIRIN2-AS1
LOC286094
DDX43
EFHA2
FAM46A
ZNF252
LRRC16A
TMED10P1
TBC1D22B
C8orf77
TMEM30A
LOC286114
SAYSD1
C8orf31
C6orf70
ZFP41
LMBRD1
SCARA5
TRERF1
LOC286135
ACOT13
RNF5P1
ECHDC1
C8orf83
APOM
DPY19L4
BTNL2
FBXO43
KCNQ5
LOC286177
TRIM39
NKAIN3
AGPAT4
LOC286184
TULP4
LOC286186
RARS2
PPP1R42
HYMAI
LOC286189
PDSS2
LOC286190
GOPC
GPIHBP1
LYRM4
LOC340357

C6orf162
KLHL38
VARS2
NRBP2
GPR126
ZNF517
KIAA1244
KIAA1875
NHSL1
C8ORFK29
LYRM2
RSPO2
SNX14
POTEA
MRS2
SLC10A5
PLEKHG1
LOC349196
ARID1B
SPATC1
LRFN2
USP17L2
AARS2
CA13
XPO5
XKR5
SERINC1
FAM90A25P
HACE1
LOC389641
FAM135A
C8orf80
TMEM181
C8orf86
ZBTB2
FAM150A
BEND3
XKR9
KIAA1586
LOC389676
CPNE5
RBM12B
LSM2
FLJ43860
C6orf47
MAFA
LY6G5B

LOC392196
C6orf115
LOC392232
RRAGD
GDF6
LY6G6D
LOC401463
ENPP5
C8orf59
PRDM13
SAMD12
PBOV1
MIR124-1
BACH2
MIR124-2
ELOVL5
MIR30B
TRMT11
MIR30D
SMAP1
MIR320A
ZFAND3
DEFB103A
SLC22A23
C8orf82
C6orf164
FER1L6-AS1
MUTED
OR4F21
CCDC90A
FAM90A13
GPSM3
FAM90A5
FKBPL
FAM90A7
KIF13A
FAM90A8
PERP
FAM90A18
SMOC2
FAM90A9
POPDC3
FAM90A10
ZNF323
FLJ39080
ALDH8A1
FLJ46284

C6orf106
FLJ42969
MICAL1
C8orf85
MRPL14
LRRC24
DLK2
DEFA10P
LY6G6E
C8orf22
ULBP3
MIR383
OR2A4
LINC00293
C6orf211
DEFB107B
OGFRL1
DEFB104B
FAM184A
DEFB106B
ZDHHC14
DEFB105B
ZNF322
C8orf58
MANEA
LINC00251
ADGB
SAMD12-AS1
TREML2
ZFAT-AS1
RPP21
HAS2-AS1
LINC00472
DEFB135
ATAT1
DEFB136
FRMD1
DEFB134
AGPAT4-IT1
ZNF704
C6orf208
C8orf69
C6orf97
C8orf75
MYCT1
MBOAT4
ZKSCAN3

LINC00051
ULBP2
MIR486
ULBP1
DEFB109P1B
ZSCAN16
SNHG6
LPAL2
SNORD87
RNF39
C8orf73
SLC44A4
SCXB
VWA7
LINC00535
C6orf25
UG0898H09
LY6G6C
RPL23AP53
LY6G5C
RAD21-AS1
PRR3
FAM90A14
KHDC1
FABP9
ZNRD1-AS1
FABP12
PRRT1
FAM86B2
EGFL8
SPAG11A
HCG4B
FER1L6
GPR63
MIR596
TXNDC5
MIR597
COL21A1
MIR598
C6orf62
MIR599
OR5V1
MIR661
OR2B2
LOC727677
PPP1R14C
HEATR7A

OR12D3
LOC728024
SPACA1
DEFA1B
RNF146
FAM90A20
TMEM14B
LOC728724
SF3B5
FAM90A19
TAAR8
ZNF705D
SH3BGRL2
OC90
RIOK1
LOC731779
TFAP2D
MIR875
KCNK16
MIR937
RSPH3
MIR939
TTLL2
LOC100127983
DTNBP1
LOC100128126
ARMC2
LOC100128338
FBXO30
LOC100128750
RPF2
FAM66B
MNF1
LOC100128993
L3MBTL3
TCF24
FAM120B
SCXA
MCHR2
LOC100130155
PGBD1
LOC100130231
FAXC
CCDC166
FNDC1
LOC100130298
TTBK1

LOC100130301
GJA10
LRRC69
RTN4IP1
LOC100130964
ADTRP
LOC100131726
RSPO3
ZNF705G
LRP11
FAM66E
LTV1
LOC100132396
SERAC1
LOC100132891
USP45
FAM66D
REPS1
FAM66A
HIST1H2AH
SBF1P1
HIST1H2BK
LOC100133267
PPIL4
LOC100133669
PAQR8
LOC100192378
SLC22A16
LOC100287015
RRP36
DEFT1P2
C6orf7
LOC100287846
KCNK17
LOC100288181
ABCC10
REXO1L2P
TRIM15
LOC100288748
KLC4
DEFB4B
UBE2CBP
MIR1205
MLIP
MIR1322
LINC00473
MIR1206

KIAA1919
MIR1207
TJAP1
MIR1204
ARHGAP18
MIR548I3
POM121L2
MIR1234
SYTL3
MIR2053
GTF3C6
MIR2052
MRAP2
MIR1208
RWDD2A
MIR4287
IL17F
MIR3148
FAM54A
MIR4288
SFT2D1
MIR3150A
EFHC1
MIR3151
BTBD9
LOC100499183
KLHL32
LOC100500773
TMEM200A
MIR3926-2
SCAND3
MIR3622A
MB21D1
MIR3926-1
FOXP4
MIR3622B
KLHDC3
MIR3150B
NUS1
MIR3610
C6orf72
LOC100505659
SLC26A8
LOC100505676
IL22RA2
LOC100505718
MAS1L

LOC100506990
C6orf192
LOC100507117
SLC16A10
LOC100507156
IP6K3
LOC100507341
TAGAP
LOC100507632
TAF8
LOC100507651
ADAT2
C8orf44-SGK3
RIPPLY2
ZHX1-C8ORF76
IRAK1BP1
MIR4469
CLVS2
MIR378D2
TAAR9
MIR548O2
TAAR1
MIR4661
STXBP5
MIR4663
NCOA7
MIR4472-1
HINT3
MIR4664
PACRG
MIR4659A
B3GAT2
MIR4660
C6orf57
MIR4659B
CD109
MIR4471
RAET1E
MIR4470
PM20D2
LOC100616530
SRSF12
LOC100652791
C6orf141
PCAT1
TRIM40
LINC00536

DPCR1
FSBP
NRSN1
LOC153910
ZC2HC1B
SNRNP48
CNKSR3
RAET1L
SAMD3
MGC34034
SLC2A12
LOC154092
MBOAT1
HDGFL1
PNLDC1
RNF217
NKAIN2
C6orf221
C6orf165
BVES-AS1
LOC154449
CCDC167
PRSS35
LCA5
OLIG3
TXLNB
DACT2
C6orf118
PSORS1C1
PSORS1C2
PPP1R18
PTCRA
OSTCP1
TCTE1
KHDRBS2
TRIM39-RPP21
TUBB
C6orf163
PIP5K1P1
CCDC162P
AKD1
NT5DC1
FAM26D
ZUFSP
FAM162B
C6orf170
BEND6

GSTA5
OPN5
GPR115
GPR116
TDRD6
SPATS1
C6orf223
RSPH9
LRRC73
TREML2P1
LOC221442
C6orf130
KIF6
TMEM217
FGD2
PI16
C6orf89
ARMC12
C6orf1
LEMD2
ZBTB9
ZBTB12
C6orf136
ZSCAN12P1
HIST1H2AA
KDM1B
RBM24
RNF182
PHACTR1
C6orf228
SYCP2L
LINC00518
PXDC1
HS3ST5
GPRC6A
RFX6
SLC35F1
FAM83B
GPR111
TSPO2
UNC5CL
KCTD20
PXT1
LHFPL5
SCUBE3
ZSCAN23
NKAPL

TOB2P1
FAM217A
VGLL2
DEFB110
DEFB112
DEFB113
DEFB114
LACE1
HCG27
C6orf191
MMS22L
WDR27
FAM26E
MCM9
RNF144B
HIST1H2BA
SCML4
GPX6
SHPRH
NCR3
LY6G6F
MDGA1
GPR110
ZNF311
LINC00326
LOC285740
CEP57L1
PPIL6
LOC285758
FLJ34503
DCBLD1
LOC285762
LY86-AS1
CAGE1
LOC285796
PRR18
LOC285819
HLA-F-AS1
HCG22
LOC285847
PNPLA1
TREML4
RPL7L1
RNF5P1
TAAR6
SLC35D3
ZC3H12D

DPPA5
IFITM4P
CLPSL1
TREML1
TREML3
RSPH4A
ECT2L
EYS
ZNF391
ZFP57
TUBB2B
SLC35B2
C6orf52
NUP43
HCG26
C6orf58
RAET1G
KAAG1
GUSBP4
GJB7
NHLRC1
ZKSCAN4
GUSBP2
HMGA1P7
SNHG5
SUMO4
C6orf147
CENPW
C6orf174
LINC00222
CEP85L
C6orf120
VN1R10P
HIST1H2APS1
THEMIS
PSMG4
SFTA2
CLPSL2
C6orf222
GLYATL3
GFRAL
LIN28B
SAMD5
IYD
MUC21
FLJ23152
LINC00340

LOC401242
MCCD1
SAPCD1
LINC00336
FLJ41649
CRIP3
KLHL31
DKFZp451B082
TCP10L2
LINC00242
DEFB133
C6orf201
GTF2H5
ERVFRD-1
MIR206
MIR219-1
MIR30A
MIR30C2
HCG23
HCG25
HCG18
C6orf226
TMEM151B
OOEP
FAM26F
FLJ46906
LOC441177
OR2B3
OR2J3
OR14J1
OR10C1
ATP6V0CP3
PTCHD4
MCART3P
GSTM2P1
RFPL4B
MIR133B
GGNBP1
HCG11
LOC554223
SNORD101
SNORD100
SNORA33
C6orf225
TSG1
LOC643623
TRAF3IP2-AS1

CTAGE9
LOC645434
RAET1K
C6orf132
RPS16P5
PPP1R3G
MLLT4-AS1
SNORA20
SNORA29
SNORA38
SNORD50B
SNORD32B
SNORD84
SNORD117
MIR548A1
MIR548B
MIR586
LOC728012
TPI1P3
C6orf186
HULC
LOC729176
LOC729177
LOC729178
TMEM242
LOC729603
LOC730101
GSTA7P
TDRG1
TMEM170B
SCARNA27
MIR877
HGC6.3
LOC100128176
BET3L
KHDC1L
LOC100129518
LOC100129636
LOC100130275
LOC100130357
PSORS1C3
LOC100130890
C6orf99
LOC100131047
LOC100131289
LINC00271
LOC100132354

LOC100132735
LINC00240
TOMM6
LOC100270746
LOC100287632
LOC100287718
LOC100288198
LOC100289495
LOC100293534
LOC100294145
NHEG1
MIR1275
MIR1913
MIR2113
MIR1236
MIR548H3
LOC100422737
MIR3143
MIR3145
MIR4282
MIR3918
MIR3939
MIR3668
MIR3662
MIR3925
MIR3692
MIR3691
LOC100506207
LOC100506409
LOC100506804
LOC100507173
LOC100507194
LOC100507203
LOC100507254
LOC100507362
MICA
LOC100507462
LOC100507463
LOC100507489
LOC100507547
LOC100507557
LOC100507584
MUC22
CAHM
MUTED-TXNDC5
EEF1E1-MUTED
RPS10-NUDT3

MSH5-SAPCD1
ATP6V1G2-DDX39B
PPT2-EGFL8
MIR4464
MIR4647
MIR4466
MIR4643
MIR4641
MIR4465
MIR4646
MIR4640
MIR4639
MIR4642
MIR4462
MIR4644
LOC100652739

PG		SARC				SKCM		
Subtype B		Subtype A		Subtype B		Subtype A		Subty
Amp	Del	Amp	Del	Amp	Del	Amp	Del	Amp
		CPM	CDKN2A	CPM	RB1	NOTCH2	CDKN2A	MITF
		hsa-mir-19	MGC45800	PRRC2C	hsa-mir-31	GAB2	PTEN	[CCND1]
01		hsa-mir-42	hsa-mir-19	VGLL3	CDKN2A	ADAM30	APLP2	TERT
		GPI	hsa-mir-12	JUN	ATP1B2	REG4	MGC45800	EP300
1a		hsa-mir-65	EPHA3	UST	ZMYND11	NBPF7	RPL22	HMGCS2
		hsa-mir-12	hsa-mir-54	CCNE1	hsa-mir-1305		hsa-mir-42	CPSF6
99		MDM2	CDKN2B	ATP4B	FBXO25		hsa-mir-14	AHCTF1
05		FASLG	LINC00290	hsa-mir-11	hsa-mir-551a		STX2	CDK4
		NDUFS6	EVI2A	DNAH5	ZNF135		GPR31	hsa-mir-12
		UBA2	hsa-mir-54	BIRC2	hsa-mir-1302-11		hsa-mir-63	BMP6
		BCR	HTR1F	SPRR1B	IGSF9B		hsa-mir-21	PTP4A1
0		hsa-mir-12	hsa-mir-12	EMR1	hsa-mir-595		hsa-mir-12	hsa-mir-13
		PIGC	MTAP	CAPG	HRAS		hsa-mir-34	hsa-mir-58
8		SLC6A3	EVI2B	hsa-mir-13	EVI2A		C10orf91	KIT
24		PDCD2L	hsa-mir-54	[KCNJ2]	RPL29P2		hsa-mir-62	hsa-mir-31
80		GNAZ	POU1F1	FGFR1	FOXK2		hsa-mir-18	BRAF
17		hsa-mir-12	hsa-let-7c	TFEC	hsa-mir-602		hsa-mir-15	hsa-mir-43
		DNM3	C9orf53	USP25	PTEN		hsa-mir-34	HNRNPH1
		IRX4	NF1	GNB2L1	ACP1		hsa-mir-19	KATNAL1
51		VPREB1	hsa-mir-19	BYSL	OR2G3		hsa-mir-31	hsa-mir-9-1
		hsa-mir-12	PROS1	hsa-mir-10	CYP2E1		hsa-mir-15	hsa-mir-19
2e		C1orf9	hsa-mir-31	hsa-mir-31	NFATC1		hsa-mir-19	RUNX3
76		MRPL36	CDKN2B-AS	MDM2	COL12A1		hsa-mir-10	CA9
		TOP3B	OMG	MYSM1	COL6A2		hsa-mir-43	hsa-mir-10
		hsa-mir-54	hsa-mir-56	LOC100128	hsa-mir-1302-9		C9orf53	RANGAP1
29		C1orf105	CGGBP1	F7	hsa-mir-4269		KLLN	NOTCH2
		LPCAT1	hsa-mir-31	hsa-mir-33	IRF4		NFRKB	SCCPDH
8-2		RAB36	RAB11FIP4	TRIO	hsa-mir-3201		LINC00290	CYP27B1
23		ANXA13	hsa-mir-56	BIRC3	ZNF423		KCNAB2	hsa-mir-93
8i-1		MIR199A2	CHMP2B	SPRR2A	CASP1		NDUFS6	DSP
09		SDHAP3	BAGE	INSR	hsa-mir-135a-1		GPC1	PHF3
		PPM1F	MIR193A	CD8A	hsa-mir-4277		RAN	hsa-mir-14
[illegible]		HAS2	AADAC	hsa-mir-14	CDKN2C		KIF25	hsa-mir-33
9		MIR214	ZNF654	STAR	ALG10		hsa-mir-43	hsa-mir-12
3		LOC72861	CXADR	MDFIC	ZFP36L1		hsa-mir-42	NDUFB2
8t		PRAME	MIR4724	BTNL3	hsa-mir-570		hsa-mir-12	CSPG4
		MYC	ACPP	CCND3	hsa-mir-613		hsa-mir-12	RUFY1
81		MIR3120	ARL13B	MLANA	hsa-mir-340		PWWP2B	LINC00426
81		MIR4277	TMPRSS15	hsa-mir-62	hsa-mir-647		ITPKA	hsa-mir-55
37		POM121L1	MIR4733	LOC100131	hsa-mir-569		hsa-mir-43	hsa-mir-54
4b		NDUFB9	ADCY5	F10	COL4A1		hsa-mir-31	SRRM1
2		DNM3OS	DHFRL1	ALDH3A1	hsa-mir-1268		hsa-mir-12	CCIN
		FBXW4P1	HSPA13	MMP7	hsa-mir-1234		hsa-mir-31	INSL4

02-2	POU5F1B	ADPRH	SPRR2B	ADCYAP1	hsa-mir-31	L3MBTL2
5	RTDR1	CADM2	ZNF557	GOLGA3	hsa-mir-11	ADAM30
79	PVT1	TPTE	CD8B	LPAR6	hsa-mir-12	TFB2M
	GGTLC2	AGTR1	ALDH1A3	BOK	hsa-mir-13	METTL1
71	SQLE	C3orf38	TACC1	CDKN2B	hsa-mir-32	hsa-mir-66
	LOC96610	NRIP1	FOXP2	TP53	ST14	F13A1
82	MTSS1	ALCAM	OR4F3	TUBB8	CHD5	EYS
8	ZNF280A	VGLL3	GUCA1A	SLC25A4	SLC6A3	hsa-mir-31
5-2	KIAA0196	BTG3	GLDC	C8orf42	CAPN10	ACTB
	ZNF280B	ATP1B3	hsa-mir-31	hsa-mir-4251	PIWIL1	hsa-mir-43
	TRIB1	STX19	GAS6	ZSCAN18	MLLT4	MRPS33
98	ZDHHC8P1	USP25	ALDH3A2	PPAP2C	hsa-mir-19	ETFA
40	RNF139	ATP6V1A	MMP20	LOC283174	hsa-mir-30	hsa-mir-92
	LOC648691	LOC44097	SPRR2D	hsa-mir-153-2	hsa-mir-21	EZH2
5	ZHX1	RBM11	MBD3L2	PSMD13	CANX	RCAN3
	CES5AP1	ATR	DNAH6	EVI2B	LTK	CD72
	ZHX2	MIR4795	IGF1R	DNAH2	hsa-mir-13	JAK2
8	MIR650	CHODL-AS1	ASH2L	ABCA2	hsa-mir-62	CHADL
	ATAD2	FOXL2	MIR3666	KLLN	hsa-mir-42	PHGDH
	IGLL5	C21orf15	BTNL8	SH3YL1	AFG3L1P	SMYD3
0	TRMT12	CASR	GUCA1B	OR2G2	hsa-mir-31	TSPAN31
	WDYHV1	C21orf91	INSL4	ECHS1	hsa-mir-32	hsa-mir-93
	GSDMC	CBLB	hsa-mir-43	CTDP1	hsa-mir-12	GCNT2
8-2	DERL1	SAMSN1	LAMP1	COX7A2	DMRT1	hsa-mir-31
	TATDN1	CD80	DRG2	FTCD	hsa-mir-49	GNA12
	C8orf76	BAGE5	YAP1	DMRT1	BARX2	hsa-mir-63
7	FAM83A	CD86	SPRR2E	NDUFA10	RNF207	ADCK2
	WDR67	BAGE4	MBD3L5	EXOC2	IRX4	PTPN9
	FBXO32	CD47	GGCX	ACR	ANKMY1	hsa-mir-42
	ZNF572	BAGE3	MEF2A	C16orf78	FZD10	ZNF212
	TMEM65	CP	BAG4	CASP4	TCP10	CLIC4
	LOC157381	BAGE2	OR4F16	hsa-mir-566	hsa-mir-31	CLTA
8-1-as	FAM84B	CPA3	PGC	NDUFS6	hsa-mir-30	RLN1
	FAM91A1	CHODL	JAK2	FAF1	ACTC1	REG4
	NSMCE2	CPB1	ARHGAP5	ALG10B	FLT4	LOC14913
73	KLHL38	ANKRD30B	GRK1	RAD51B	TYRO3	TSFM
	FER1L6-AS	CSTA	FOXO3B	APOD	hsa-mir-12	hsa-mir-13
	HAS2-AS1	LIPI	KIAA1377	CDKN1B	hsa-mir-43	HIVEP1
	FER1L6	DRD3	SPRR2F	hsa-mir-1229	hsa-mir-12	hsa-mir-9-
	LOC72767	ABCC13	EMR4P	NPBWR2	APRT	GPER
	LOC72872	EPHB1	MAT2A	hsa-mir-551b	hsa-mir-94	AANAT
6a	LOC10013	C21orf91-C	PCSK6	COL4A2	ACTA1	LOC10013
	LOC10013	GAP43	DDHD2	hsa-mir-3118-6	hsa-mir-31	RCN2
	MIR1205	POTED	TRIM7	hsa-mir-939	FOXD4	hsa-mir-19
24	MIR1206	GATA2	TFEB	CETN1	hsa-mir-12	ZNF282
	MIR1207	LOC38881	RLN1	POLE	ARHGAP32	NIPAL3
	MIR1204	GOLGB1	CFL2	DTYMK	MRPL36	NPR2

	MIR1208	LINC00478	TFDP1	C9orf53	RNPEPL1	RLN2
	ZHX1-C8OF	GP9	FLII	C10orf108	RIMBP2	NBPF7
	MIR4663	ANKRD20A	MMP27	CASP3	THBS2	CNST
42	PCAT1	GSK3B	FLJ25758	ERICH1	AES	METTL21B
		MIRLET7C	SFTPB	hsa-mir-429	BAI3	hsa-mir-15
		GTF2E1	SNRPA1	ZNF606	APBA2	MAK
84		MIR125B2	LSM1	THEG	GRM6	hsa-mir-7-2
		GYG1	TRIM52	LOC100128239	MAPKBP1	ICA1
		MIR99A	MED20	PTPRN2	hsa-mir-33	ACOX1
		HCLS1	RLN2	RNH1	RCBTB2	TYRO3P
		TEKT4P2	COCH	NF1	hsa-mir-58	hsa-mir-55
		HGD	CUL4A	CACNA1B	C16orf3	CUL1
03		MIR3156-3	LLGL1	FAM150B	hsa-mir-32	GRHL3
		ITGB5	C11orf70	OR2C3	ACTN2	RMRP
		MIR3687	MBD3L4	DUX4	hsa-mir-54	SLC1A1
36		KPNA1	RNF103	TXNL4A	MLANA	LOC25565
		MIR3648	NR2F2	HTR1B	hsa-mir-21	MARCH9
		LSAMP	WHSC1L1	C21orf56	ADAMTS8	hsa-mir-30
		C21orf37	TRIM41	FOXD4	LPCAT1	NEDD9
		TM4SF1	FRS3	HDAC4	OTOS	ACAN
		MBNL1	SLC1A1	DUSP22	TMEM132I	LFNG
41		MCM2	FOXG1	ARSA	C6orf123	ACTG1
		CD200	PROZ	CASP5	AMH	PSTPIP1
		MYLK	MFAP4	hsa-mir-4271	BCKDHB	hsa-mir-42
72		NCK1	TMEM123	SDHA	CHRM5	PDIA4
		NDUFB4	MBD3L3	DMRTA2	HNRNPAB	C1orf201
		P2RY1	VAMP8	CPNE8	RTF1	TESK1
		PCCB	CHSY1	MUC4	hsa-mir-49	RCL1
		PFN2	PPAPDC1B	APOLD1	CPB2	AGAP2
		PIK3CB	BTNL9	hsa-mir-4281	hsa-mir-21	ADCY8
94		PLOD2	USP49	MYT1	CA5A	RREB1
		PLS1	RCL1	hsa-mir-720	hsa-mir-42	ALDH1A3
		PLSCR1	GPR33	hsa-mir-3118-4	PARP1	NUDT1
5a-1		PLXNA1	RASA3	CYC1	hsa-mir-58	BIRC5
		PPP2R3A	MAPK7	TYMS	GLDC	SNUPN
		RAP2B	SUCLG1	PXMP2	hsa-mir-22	ADAR
		RASA2	SYNM	SEPT2	ZBTB44	SSPO
		RBP1	LETM2	CDKN2B-AS1	CLPTM1L	IL28RA
		RBP2	OR2V2	DCTD	DUSP28	TLN1
8		RHO	PRICKLE4	ZNF596	FLJ31485	INSL6
		RPN1	INSL6	hsa-mir-1302-2	UNC93A	BAI1
		RYK	FOXA1	ZSCAN1	ATP5D	SSR1
		SIAH2	ATP11A	MIER2	CGA	ANPEP
		SLC15A2	PRPSAP2	MIR4697	CHRNA7	PDGFA
		SLCO2A1	ST3GAL5	VIPR2	HNRNPH1	ARHGDIA
		HLTF	OR4F4	IFITM1	MGA	TSPAN3
		TF	RNF5P1	OMG	hsa-mir-61	ARNT

	TFDP2	SNORD95	ENTPD2	ELF1	CNTNAP2
5	TM4SF4	MRPS10	FAM110C	hsa-mir-10	LOC284632
	TRH	KDM4C	C1orf150	CBFA2T3	TPM2
	TRPC1	NFKBIA	DUX2	hsa-mir-19	CD274
	UPK1B	MCF2L	ADNP2	AGT	CYC1
	UMPS	SHMT1	IMPG1	ACAT2	TFAP2A
	CLRN1	TMSB10	DMRT2	INSL4	BLM
	ZIC1	LINS	MGC16025	hsa-mir-31	PMS2
	CNBP	C8orf86	FOXQ1	PRDM10	CD7
4	ZNF80	SNORD96A	CHKB	SDHAP3	HMG20A
	ZNF148	TRERF1	CARD18	MIR149	BGLAP
	RAB7A	RANBP6	hsa-mir-191	LOC10019	ZNF777
	SOX14	PAX9	SLC6A3	SMOC2	C1orf130
	BFSP2	TMCO3	SYT10	AZU1	RECK
	RUVBL1	SREBF1	PCYT1A	CNR1	AK3
	B4GALT4	GNLY	MIR613	GABRA5	CYP11B1
	SNX4	SELS	hsa-mir-1271	LTC4S	GCM2
4	NR1I2	ZFP62	OPRL1	RPAP1	CHD2
	MBD4	TAF8	hsa-mir-1263	hsa-mir-61	PRKAR1B
	H1FX	CD274	APBA2	ESD	CDK3
	KALRN	PRKD1	GPT	hsa-mir-14	SIN3A
	COPB2	DCUN1D2	YES1	CDH13	CHRNB2
	CHST2	TOP3A	ZNF10	hsa-mir-55	ZNF398
	STXBP5L	TGOLN2	PDCD1	ARF1	RCAN3AS
	RNF7	TTC23	F11	ARG1	RGP1
6	GUCA1C	LOC72967	OR4F21	JAK2	C9orf68
	IQCB1	C6orf132	CDK11B	ABR	CYP11B2
	DZIP3	AK3	LOC100128398	TMEM45B	CDYL
	TSC22D2	PSMA6	OR4F17	LOC72861	FES
	P2RY14	PCID2	NCAPG2	PP14571	RAC1
	COX17	RNF112	IFITM3	FRMD1	CSNK1D
	CD96	USP39	MIR4733	CIRBP	FBXO22
	STAG1	LRRK1	FUT7	COL9A1	CKS1B
89-2	POLQ	OR4F29	LOC148824	GABRB3	ZNF767
	ALDH1L1	TOMM6	MTG1	MGAT1	MELK
	PDIA5	C9orf68	KCNG2	MIR626	CDC37L1
	TOPBP1	SRP54	MYO6	hsa-mir-12	EEF1D
	HHLA2	GRTP1	KANK1	FOXO1	LY86
	FSTL1	ULK2	TWIST2	hsa-mir-31	IDH2
	MRPL3	VAMP5	HUS1B	CDH15	RPA3
	RNF13	TM2D3	CPT1B	hsa-mir-13	SLC25A10
44-2	MGLL	LOC100132	CARD16	CAPN2	SCAPER
	MRAS	CDC37L1	hsa-mir-711	CCR6	CLK2
	COPG	SSTR1	SLC9A3	PTPRD	KRBA1
	MYH15	ADPRHL1	PPP1R2	ACADVL	RUSC2
	TMCC1	PEMT	hsa-mir-585	ADAMTS15	C9orf46
	PLXND1	IMMT	PTK6	MIR4277	GLI4

	DNAJC13	ARRDC4	hsa-mir-16-2	TTLL2	EEF1E1
	U2SURP	LOC100132	NBEAP1	CNN2	IGF1R
8f-3	C3orf27	C9orf46	GRINA	COL12A1	FSCN1
	ANAPC13	NKX2-1	USP14	GABRG3	EVPL
	ARMC8	FAM70B	ZNF26	MAPK9	IMP3
	C3orf17	FBXW10	FARP2	JMJD7	CTSK
	WWTR1	POLR1A	ACSL1	APAF1	ZNF786
	PVRL3	LOC91948	RPL23AP53	MLNR	GNE
	ZBTB20	LOC10013	DFFB	hsa-mir-13	PDCD1LG2
	FAM162A	KIAA1432	C2CD4C	COX4I1	GML
8p	PCOLCE2	AKAP6	WDR60	hsa-mir-76	FARS2
	SEC22A	FLJ44054	IFITM2	LYST	ISG20
	NPHP3	RAI1	GRIN1	CTGF	ZNF12
	ATP2C1	RNF181	OR2W5	RFX3	FASN
	MORC1	TARSL2	SYCE1	ALOX12	ISL2
	SERP1	MIR4638	RBFA	SNX19	CTSS
	ACAD9	ERMP1	SENP6	DACT2	ZNF425
	ASTE1	KIAA0391	CBWD1	CSNK1G2	CREB3
3	KLF15	FLJ41484	OR6B3	COL19A1	GLIS3
	GTPBP8	GRAP	LOC285768	IPW	GPR20
	TAGLN3	MRPL35	TYMP	PROP1	RPP40
	GPR171	LRRC28	CARD17	ARL1	MAN2A2
	SEC61A1	LOC10085	hsa-mir-2115	GTF2F2	AIMP2
	PIK3R4	PDCD1LG2	TERT	hsa-mir-31	FDXR
	PODXL2	AP4S1	TFRC	CYBA	PEAK1
	TRAT1	LINC00552	hsa-mir-218-2	hsa-mir-18	ECM1
77	COMMD2	AKAP10	SRMS	CHRM3	LOC15506
	A4GNT	CHMP3	AADAC	EPB41L2	UNC13B
	DBR1	ASB7	GABRA5	RLN1	PPAPDC2
	CLDN18	TPD52L3	HSF1	ALOX12B	GPT
	TIMMDC1	BAZ1A	THOC1	LINC00167	SLC35B3
	PLA1A	MCF2L-AS1	ZNF84	TCP10L2	MEF2A
	AMOTL2	EPN2	STK25	DAPK3	MAFK
	CCRL1	RETSAT	FAT1	COX7A2	FOXJ1
2	RAB6B	LYSMD4	DVL1	MEIS2	C15orf5
	SELT	IL33	WASH5P	ZNF354A	EFNA1
	FBXO40	SCFD1	ESYT2	ASCL1	ZNF746
	P2RY13	LOC10050	PKP3	GUCY1B2	SPAG8
	IL20RB	USP22	SSNA1	hsa-mir-12	MIR101-2
	GPR87	PTCD3	PRAP1	DPEP1	GRINA
	SEMA5B	PGPEP1L	PQLC1	hsa-mir-12	NRN1
	XRN1	UHRF2	FILIP1	EPHX1	MFGE8
80	WDR5B	HECTD1	DMRT3	EYA4	MAD1L1
	PARP14	B9D1	LOC150935	RLN2	GAA
	GRAMD1C	KDM3A	MAPK11	ALOX12P2	LINGO1
	ROPN1	LOC14582	LOC643733	MLLT4-AS1	EFNA3
	SIDT1	KIAA2026	hsa-mir-1226	CFD	ATP6V0E2

	SLC41A3	HEATR5A	TRIP13	EEF1A1	SIT1
	SLC35A5	FAM18B1	TNK2	TRPM1	HSF1
	IFT57	KCMF1	hsa-mir-103-1-as	SQSTM1	TMEM14C
2	MSL2	SPATA8	TCEA2	ATP2B1	NTRK3
	FAIM	GLIS3	AGTR1	HTR2A	EIF3B
	SLC25A36	NKX2-8	GABRB3	hsa-mir-37	GALK1
	DPPA4	MYO15A	TONSL	FANCA	UBE2Q2
	TMEM39A	RMND5A	NDC80	hsa-mir-31	EFNA4
	ABHD10	ADAMTS17	ZNF140	GALNT2	ZNF467
	CDV3	PPAPDC2	ATG4B	ESR1	TMEM8B
	IFT122	STRN3	FRG1	SLC1A1	KCNQ3
9c	WDR52	RASD1	MEGF6	ALOX15	ELOVL2
	EAF2	REEP1	FLJ45445	HGC6.3	FURIN
	MUC13	DNM1P46	LOC154822	ARID3A	CYTH3
	SUCNR1	MIR101-2	SIRT3	EPHA7	GCGR
	C3orf37	MBIP	CLIC3	NDN	C15orf27
	MRPS22	ALKBH5	PAOX	ADAMTS2	ENSA
	POGLUT1	TCF7L1	PARD6G	BTG1	C7orf33
	BBX	CERS3	PHIP	KPNA3	OR2S2
1	PLSCR2	MIR4665	DOCK8	hsa-mir-14	LY6E
	PLSCR4	PPP2R3C	OR6B2	FOXF1	PAK1IP1
	ISY1	MED9	MAPK12	hsa-mir-10	PCSK6
	HEG1	ELMOD3	ACY1	GNG4	KIAA0415
	KIAA1257	FAM169B	PDCD6	FUCA2	UTS2R
	ARHGAP31	G2E3	ACAP2	SMARCA2	NRG4
	KIAA1407	SLC47A1	hsa-mir-146a	ALOX15B	FDPS
	KIAA1524	ATOH8	TPD52L2	EEF2	LOC401431
	SRPRB	WASH3P	BCHE	GABRR1	GBA2
	BPESC1	EAPP	GABRG3	OCA2	LY6H
	EEFSEC	C17orf39	RPL8	MAML1	LYRM4
	CLSTN2	LOC90784	SMCHD1	CRY1	PLIN1
	POPDC2	FLJ42289	ZNF268	LCP1	KDELR2
	ATG3	SNX6	THAP4	hsa-mir-58	GPS1
	CCDC14	FAM106A	HSP90AA4P	FOXL1	ODF3L1
ɔ	P2RY12	C2orf89	GABRD	hsa-mir-31	FLG
	TMEM108	OR4F6	FAM138F	GUK1	ZNF862
	ZXDC	NPAS3	MIR153-2	GPR31	HINT2
	ZBED2	LRRC48	BET1L	TYRP1	TONSL
	HSPBAP1	TMEM150	TUBB4B	ARRB2	MUTED
	C3orf52	OR4F15	C10orf125	EFNA2	POLG
	QTRTD1	NUBPL	ATP9B	GABRR2	ADAP1
	CCDC48	ATPAF2	TMEM30A	RYR3	GRB2
	NEK11	SH2D6	C9orf66	GFPT2	LOC25304
	UBA5	FAM138E	FLJ43879	DCN	GBA
	C3orf36	BRMS1L	SBF1	MAB21L1	ZNF783
	NAA50	SPECC1	ALAS1	hsa-mir-87	C9orf100
	CEP63	LOC28495	SLC12A7	FOXC2	PLEC

CEP70	GPCRLTM7	UBXN7	ABCA4	TXNDC5
ABTB1	INSM2	hsa-mir-3142	H3F3A	RLBP1
TMEM22	SMCR7	TNFRSF6B	GRM1	IQCE
PARP9	FUNDC2P2	CP	VLDLR	GRIN2C
ESYT3	DDX11L1	IPW	ASGR1	SNX33
EIF2A	ARHGAP5-/	ZNF7	ELANE	GBAP1
ZIC4	SLC5A10	CLUL1	HTR1B	MIR548F3
ACAD11	C2orf68	P2RX2	SCG5	FP588
CHCHD6	DDX11L9	GAL3ST2	GNB2L1	PTK2
HPS3	SLC25A21	ING2	EPYC	TMEM14B
SLC12A8	SMCR5	GNB1	SMAD9	SNRPA1
RETNLB	SNORD94	FAM138A	hsa-mir-88	SUN1
CCDC54	MIR1469	MIR595	GALNS	H3F3B
DIRC2	EGLN3	SIGIRR	ACADM	LOC645752
C3orf15	SMCR8	NOXA1	ITPKB	IL6R
PHLDB2	RNF103-CH	SPRNP1	HIVEP2	MIR548I4
LOC90246	LOC100507	HSBP1L1	KIAA0020	RNF38
C3orf25	C14orf126	IRAK1BP1	ASGR2	RPL8
BOC	TOM1L2	FAM138C	GAMT	RIOK1
SPSB4	MIR4779	PRR21	HTR1E	NR2F2
ACPL2	MIR4714	PPP6R2	SNRPN	WIPI2
TXNRD3	MIPOL1	AMT	BTNL3	ICT1
OSBPL11	SLC47A2	TPPP	DUSP6	FBXO22-AS
LRRC58	LOC10063(	TM4SF19	RB1	ILF2
TM4SF18	CLEC14A	hsa-mir-1303	hsa-mir-12	MIR548F4
MED12L	TRIM16L	ARFRP1	GAS8	GLIPR2
CLRN1-AS1	SPTSSA	CPA3	ADORA3	ST3GAL1
CPNE4	USP32P2	NDN	KCNK1	ADTRP
CCDC58	RALGAPA1	ZNF16	IFNGR1	ST8SIA2
CD200R1	TBC1D28	ENOSF1	RCL1	INTS1
TPRA1	SFTA3	ANKLE2	ASPA	FOXK2
UROC1	CDRT15L2	ING5	GNA11	DNM1P35
FAM194A	IGBP1P1	IRF2	IMPG1	IVL
NUDT16	CCDC144B	PEX10	TJP1	MIR548T
COL6A6	FAM177A1	LOC100506585	MGAT4B	LOC15837(
GRK7	LGALS9B	RIC8A	ELK3	SLA
RPL32P3	C14orf23	MAN1B1	RFC3	SNRNP48
H1FOO	CCDC144N	FRG2B	hsa-mir-12	PEX11A
PISRT1	MIR624	LOC100130522	GCSH	EIF2AK1
DTX3L	LOC33924(	LOC100506804	AGL	ITGB4
LOC15165	LOC10012	WASH1	LBR	MIR4313
DPPA2	CCDC144C	MIR4269	IGF2R	KCNN3
CCDC80	MIR4307	ZBED4	DMRT2	ATP8B5P
BTLA	GRAPL	APEH	ATP1B2	TG
ROPN1B	LOC10050	EXOC3	GNA15	PIP5K1P1
C3orf22	FAM83G	ZDHHC19	ME1	IQGAP1
SPICE1	LOC10050(	hsa-mir-3141	UBE3A	SNX8

NUDT16P1	KRT16P3	RGS19		B4GALT7	LGALS3BP
IGSF11	MIR4503	CPB1		HAL	LMNA
C3orf30	EVPLL	OCA2		RFXAP	CCDC107
PARP15	MIR548AI	DGAT1		ADRA1B	TSTA3
DNAJB8	LGALS9C	LINC00470		HSBP1	C6orf228
GPR156	SNORA59B	GALNT9		AK4	PRC1
CHST13	SNORA59A	NEU4		LGALS8	FTSJ2
KBTBD12	MIR33B	KLKB1		KIF25	LLGL2
DZIP1L	ZNF286B	PRKCZ		INSL6	LOR
ALG1L	MIR1180	ATHL1		ATP2A3	C9orf128
PTPLB		COBRA1		GNG7	ZNF7
TIGIT		SPRN		MYO6	SYCP2L
LOC201651		MIR2467		PAR5	SV2B
C3orf58		SCO2		TBC1D9B	NXPH1
KIAA2018		ARF4		IGF1	MAFG
ZBTB38		PP7080		TPT1	MCL1
ZDHHC23		OSTalpha		ADRB2	OR13J1
NUP210P1		hsa-mir-1294		HSD17B2	ZNF16
COL6A5		PRPF6		ALX3	LINC00518
GK5		ECT2		MTR	AP3S2
PLCXD2		SNRPN		LAMA2	GET4
GCET2		GPAA1		KDM4C	NPTX1
LSAMP-AS3		METTL4		C1QBP	SMCP
SLC9A9		CHFR		MKNK2	HRCT1
LOC285205		CXXC11		NT5E	PSCA
DNAJB8-AS1		MTNR1A		HERC2	LY86-AS1
RABL3		SCNN1D		N4BP3	SEMA4B
IGSF10		PTDSS2		LTA4H	CCZ1
SLC9A10		NELF		TRPC4	P4HB
ILDR1		LOC619207		ANXA6	MEF2D
TRIM42		MIR4440		IRF8	MSMP
RAB43		RABL2B		AMPD1	LY6D
KY		RHOA		NID1	CAGE1
LOC339874		IRX4		LPA	CIB1
H1FX-AS1		MUC20		KANK1	MIOS
LOC344595		hsa-mir-378		CD68	PDE6G
AADACL2		GMEB2		GPX4	MTX1
TMPRSS7		MECOM		PGM3	FAM166B
CD200R1L		TJP1		SNURF	JRK
PAQR9		FOXH1		OR4F3	C6orf52
NME9		COLEC12		LUM	ABHD2
CCDC37		FBRSL1		TNFSF11	RNF216
NPHP3-AS1		D2HGDH		ATOX1	PRPSAP1
NMNAT3		TLR3		MAF	MUC1
PRR23B		SKI		AMPD2	MIR4667
PRR23C		ODF3		NVL	DGAT1
PLSCR5		NDOR1		MAS1	ERVFRD-1

	ANKUB1	DUX4L7	RANBP6	CHSY1
	FLJ22763	MIR4786	CHD3	ZNF853
	FLJ25363	MLC1	MATK	PYCR1
	C3orf72	C3orf51	RNY4	NPR1
	LOC401093	CEP72	AQR	GPAA1
	MIR198	TCTEX1D2	ZNF354C	PPP1R3G
	ARGFX	hsa-mir-145	KITLG	SYNM
	ALG1L2	STMN3	SUCLA2	CYP2W1
	TXNRD3NB	GHSR	ALDH7A1	PCYT2
	TMEM14E	UBE3A	MC1R	PI4KB
	COL6A4P2	RECQL4	AMY1A	WISP1
	LOC646903	C18orf56	PSEN2	HULC
.S	LOC653712	NOC4L	MAP3K4	GABARAPL
	LINC00488	LOC728323	CD274	HEATR2
	SNORA7B	SORBS2	CHRNB1	RAC3
	SNORA58	TP73	GADD45B	PKLR
	MIR567	SCGB1C1	ELOVL4	FOXH1
	MIR568	ANAPC2	ARHGAP11	TMEM170
	FAM86HP	DUX4L6	C5orf45	VPS33B
	PRR23A	MIR4441	MYBPC1	ZDHHC4
	LOC100125556	MAPK8IP2	DLEU2	RFNG
	WWTR1-AS1	CACNA1D	BNIP1	PSMB4
	LOC100129550	AHRR	MVD	RECQL4
	ZBTB20-AS1	SDHAP1	AMY1B	SCARNA27
	LOC100289361	hsa-mir-584	RAB4A	MRPL46
	MIR1280	RTEL1	MAP3K5	CHST12
	MIR548I1	GYG1	AK3	MRPL12
	LOC100302640	MKRN3	CHRNE	PSMD4
	MIR548H2	LRRC14	NFIC	LRRC14
	PVRL3-AS1	CBX3P2	MAP3K7	LOC10013
	IGSF11-AS1	PGAM5	SLC12A6	OR4F4
	MYLK-AS1	BOK-AS1	TMED9	RADIL
	LOC100507032	DUX4	MYF5	SEC14L1
	LOC100507389	TNFRSF4	TSC22D1	RAB13
	NPHP3-ACAD11	NLRP6	CAMK2A	ZNF623
	ISY1-RAB43	DPP7	CHMP1A	MIR3691
	MIR4796	DUX4L5	AMY1C	SLCO3A1
	MIR4788	PLXNB2	RYR2	PAPOLB
	MIR4446	SLC25A20	MLLT4	SECTM1
		MRPL36	C9orf68	RFX5
		LOC401109	CLDN7	HHLA1
		ADRA1B	OAZ1	LOC10050
		SOX18	TPBG	NGRN
		IL12A	FAN1	C1GALT1
		PAR5	NHP2	SRSF2
		BOP1	MYF6	RIT1
		ROCK1P1	CCNA1	NDRG1

DDX51	CAMLG	LOC10050€
FAM149A	PLCG2	RHCG
MMP23B	AMY2A	FAM20C
ANO9	SRP9	SGSH
C9orf167	MYB	RORC
DUX4L3	CDC37L1	KHDRBS3
BRD1	CRK	MUTED-TX
CAMP	POLR2E	DET1
BRD9	TTK	RBAK
MIR570	GOLGA8A	SUMO2
ADRB2	RNF130	RPS27
LIME1	PPP1R12A	PTP4A3
KPNA4	DCLK1	EEF1E1-MU
HERC2	CANX	LINS
FBXL6	RPL13	C7orf26
LOC647589	AMY2B	SRP68
DUX2	TARBP1	S100A1
MMP23A	NMBR	PUF60
B4GALNT4	C9orf46	FANCI
EXD3	CTNS	MICALL2
DUX4L2	MAP2K2	TBCD
TBC1D22A	RNGTT	S100A2
CDC25A	FAM189A1	DENND3
IRX1	CLK4	MCTP2
SDHAP2	NFYB	FBXL18
ANXA6	KL	TIMP2
UCKL1	CCNG1	S100A3
TM4SF1	SPG7	ZC3H3
SNURF	RHOC	SELS
OPLAH	TBCE	TTYH3
LOC100130238	OPRM1	TK1
PDLIM3	CBWD1	S100A4
TNFRSF14	DLG4	EFR3A
IFITM5	PRTN3	MESP1
NPDC1	TBX18	USP42
MIR3944	C15orf2	CBX4
FAM19A5	CNOT6	S100A5
CISH	NTS	ARC
ZDHHC11	ITM2B	UNC45A
TM4SF19-TCTEX1D2	CD14	PSMG3
ATOX1	GAN	DNAH17
PCMTD2	ATP1A1	S100A6
MBNL1	LEFTY2	BOP1
CYFIP1	PARK2	RGMA
COMMD5	KIAA1432	C7orf50
ZNF605	DPH1	GALR2
CLDN22	RPS15	S100A7

	TNFRSF18	HMGN3	SCRIB
	LOC653486	DKFZP434L	WDR93
	MRPL41	GMCL1P1	CARD11
	NCAPH2	PAH	SPHK1
	COL7A1	MTRF1	S100A8
	LPCAT1	CD74	LRRC6
	BNIP1	SLC7A5	AEN
	SLC2A4RG	ATP5F1	TNRC18
	MLF1	TLR5	SOCS3
	FAM189A1	PCMT1	S100A9
	CPSF1	DMRT3	FBXL6
	LOC100507055	DVL2	CRTC3
	UFSP2	SGTA	C7orf70
	ISG15	FHL5	SLC16A5
	LOC100133161	GREM1	S100A10
	EHMT1	RMND5B	OPLAH
	MOV10L1	PAWR	TTC23
	DAG1	LPAR6	ZFAND2A
	CLPTM1L	CDC25C	SLC16A3
	CAMK2A	CDK10	S100A11
	ZNF512B	BCL9	EIF2C2
)	MME	GPR137B	MRPS11
	C15orf2	PDCD2	COX19
	CYHR1	ERMP1	SYNGR2
	CDKN2AIP	EFNB3	S100A12
	PLCH2	STK11	COMMD5
	C9orf37	SNAP91	LRRK1
	MIOX	TMEM85	GLCCI1
	DNASE1L3	BTNL8	HGS
	NKD2	CDK17	S100A13
	CANX	SLC25A15	ASAP1-IT1
	C20orf195	CDO1	TM2D3
	CLDN11	MBTPS1	KIAA1908
	DKFZP434L187	BRDT	CYTH1
	VPS28	TP53BP2	SHC1
	ODZ3	ENPP1	CPSF1
	CEP104	PDCD1LG2	C15orf42
	SAPCD2	EIF4A1	GPR146
	PANX2	TBXA2R	SLC9A3R1
	DOCK3	CASP8AP2	SPRR1A
	C5orf55	KLF13	CYHR1
	CCNG1	ZFP2	RCCD1
	DNAJC5	SLC25A3	AMZ1
	P2RY1	LHFP	RECQL5
	MAGEL2	CDX1	SPRR1B
	FAM203A	TAF1C	ASAP1
	LRP2BP	CAPZA1	ARRDC4

SLC35E2	TSNAX	TMEM184A
UAP1L1	ENPP3	PGS1
CERK	DOCK8	SPRR2A
DUSP7	EIF5A	FAM135B
CCDC127	TCF3	LOC91948
CD74	SYNCRIP	BRAT1
ZGPAT	MTMR10	AATK
PFN2	RUFY1	SPRR2B
NDNL2	PMCH	PHF20L1
EXOSC4	TRIM13	TARSL2
STOX2	AP3S1	SDK1
RER1	USP10	KIAA0195
ARRDC1	CASQ2	SPRR2C
ALG12	WNT9A	VPS28
CELSR3	PEX7	LRRC28
PLEKHG4B	GLIS3-AS1	FOXK1
CDX1	ENO3	EIF4A3
PRIC285	THOP1	SPRR2D
SERPINI1	SLC35A1	FAM203A
ATP10A	ZNF770	ASB7
SLC39A4	OR4F16	MMD2
KIAA1430	RFX4	ALYREF
NOC2L	MRPS31	SPRR2E
WDR85	CLTB	KCNK9
CRELD2	C16orf7	LYSMD4
FHIT	CD2	DAGLB
C5orf38	HIST3H3	BAIAP2
CLTB	PLAGL1	SPRR2F
ZBTB46	TPD52L3	C8orf55
SERPINI2	FGF11	PGPEP1L
NIPA2	TLE2	CCZ1B
ZNF250	FUT9	ATP5H
TRAPPC11	NOP10	SPRR2G
OR4F3	TRIM7	CHRAC1
TMEM203	SNRPF	LOC14582[illegible]
ADM2	DLEU1	SLC29A4
FLNB	CSF1R	ST6GALNA[illegible]
IRX2	KIAA0513	SPRR3
CSF1R	CD53	EXOSC4
SAMD10	GNPAT	C15orf32
PLD1	PLG	RSPH10B
CHRFAM7A	IL33	SEPT9
C8orf33	GP1BA	SSR2
MLF1IP	SF3A2	LY6K
ARHGEF16	PNRC1	HAPLN3
ZMYND19	NDNL2	TFAMP1
TRABD	TRIM52	RAB40B

GNAI2	SYT1	VPS72
LOC285577	POSTN	SLC39A4
CSNK1A1	CSF2	MESP2
ABHD16B	PIEZO1	UNCX
PLOD2	CD58	TMC6
ULK4P3	CDC42BPA	THBS3
GPR172A	PSMB1	SLURP1
WWC2	C9orf123	SPATA8
SSU72	GPS2	COL28A1
SLC34A3	S1PR4	AZI1
SELO	KIAA1009	TCHH
GNAT1	C15orf24	SLC45A4
SLC6A19	AGXT2L2	ADAMTS17
NKX2-5	TDG	LOC38945
LINC00266-1	WBP4	GGA3
PLS1	CSNK1A1	TPM3
ULK4P1	ATP2C2	ZFAT
ARHGAP39	CHI3L2	DNM1P46
SNX25	DEGS1	ELFN1
WRAP73	PTPRK	JMJD6
C9orf142	UHRF2	CCT3
HDAC10	GUCY2D	ZNF250
GPX1	AP3D1	CERS3
SLC6A18	ANKRD6	GRID2IP
DBN1	AVEN	EXOC7
C20orf201	TRIM41	TUFT1
PLSCR1	TMPO	PYCRL
ARHGAP11B	AKAP11	LOC25455
ZNF34	CSNK1G3	ZNF815
ZFP42	MPHOSPH	KCTD2
SDF4	CLCA1	DAP3
TPRN	TAF1A	C8orf33
SHANK3	RPS6KA2	LOC28373
GRM2	C9orf66	RNF216P1
LRRC14B	HIC1	WBP2
DOCK2	APBA3	PIP5K1A
LINC00176	RIMS1	LYNX1
PRKCI	ATP10A	LOC283761
SNORD107	COL23A1	PMS2CL
SHARPIN	NR2C1	FSCN2
ENPP6	EXOSC8	ANXA9
MXRA8	NKX2-5	C8orf51
PNPLA7	COX4NB	FAM169B
TUBGCP6	CNN3	FLJ44511
HYAL1	GGPS1	NAT9
SDHAP3	RPS12	ADAM15
DRD1	KIAA2026	GPR172A

)	MIR647	ITGAE	ZNF774
	PTX3	MED16	LOC442497
	TUBGCP5	DOPEY1	NARF
	SCRT1	GJD2	SELENBP1
	C4orf38	SCGB3A1	GSDMD
	CPSF3L	HSP90B1	C15orf38
	C9orf169	FNDC3A	MIR339
	LOC90834	CTNNA1	SAP30BP
	IMPDH2	TUBB3	PRPF3
	LOC728613	COL11A1	ZNF696
	SLC26A2	TMEM63A	KIF7
	UCKL1-AS1	SGK1	ZNF890P
	RAP2B	KCNV2	NT5C
	NIPA1	MNT	ARHGEF2
	MAF1	EBI3	ARHGAP39
	RWDD4	ZNF292	ZNF710
	C1orf159	C15orf29	OCM
	ENTPD8	ZNF354B	CDR2L
	LMF2	TXNRD1	SLC25A44
	ITIH1	PDS5B	ZNF34
	MIR4277	DBN1	HDDC3
	DUSP1	PRDM7	MIR589
	MIR941-1	CRYZ	MRPS7
	RARRES1	TOMM20	SETDB1
.	PAR1	SLC22A1	SHARPIN
	PPP1R16A	GLIS3	WASH3P
	CCDC111	MYH10	RSPH10B2
	AURKAIP1	APC2	HN1
	LRRC26	MDN1	UBAP2L
	KLHDC7B	C15orf41	EPPK1
	ITIH3	OR2Y1	FLJ42289
	LOC100506688	UBE2N	LOC729852
	EBF1	KIAA0564	DCXR
	MIR941-4	DMXL1	SV2A
	SHOX2	TCF25	SCRT1
	LOC283683	CSF1	C15orf58
	ZNF251	URB2	LOC100131
	TRIML2	SLC22A3	ANAPC11
	MRPL20	LURAP1L	DENND4B
	C9orf139	MYO1C	TRAPPC9
	LOC284933	ABCA7	OR4F6
	ITIH4	UFL1	LOC10028
0714	MIR4457	CHRFAM7A	SIRT7
	F12	BTNL9	SCAMP3
	MIR941-2	ALX1	MAF1
	SI	ZC3H13	OR4F15
	OR4N4	DIAPH1	RBAK-LOC3

	KIFC2	ZCCHC14	TMEM104
	CCDC110	CTBS	SF3B4
	ATAD3A	LEFTY1	PARP10
	FAM166A	SLC22A2	FAM174B
	RPL23AP82	FLJ35024	MIR4648
	LAMB2	NUP88	CCDC40
	MIR4456	HMG20B	HAX1
	FABP6	ORC3	TIGD5
	MIR941-3	ULK4P3	LOC40045
	SIAH2	LOC20218	MIR4655
	HERC2P3	EEA1	NPLOC4
	MFSD3	SPG20	C1orf61
	CYP4V2	DOCK2	PPP1R16A
	PANK4	KIAA0182	MIR7-2
	C9orf173	CTH	MIR4656
	LOC339685	SPHAR	ST6GALNA
	MAP4	SOD2	SEMA6C
	MIR4635	PPAPDC2	ZNF251
	FAT2	OR1D2	MIR9-3
	MIR1914	UQCR11	WDR45L
	SKIL	MTO1	PMVK
[illegible]168	GOLGA6L1	ULK4P1	KIFC2
	ADCK5	RASGEF1C	TTLL13
	LOC285441	PPFIA2	CBX8
	TP73-AS1	LRCH1	FAM189B
	NRARP	DPYSL3	NAPRT1
	C22orf34	ATMIN	ASB9P1
	MST1	CYP2J2	MIF4GD
	FGFR4	CAPN9	JTB
	LOC100505815	T	HPYR1
	SLC2A2	MIR101-2	FAM138E
	GOLGA8G	OR3A1	CASKIN2
	ZNF252	SBNO2	MTMR11
	LOC339975	IBTK	MFSD3
	LRRC47	ARHGAP11	LOC10014
	FLJ40292	FAM153A	RPTOR
	CHKB-CPT1B	GALNT4	MLLT11
	MST1R	INTS6	RHPN1
	FOXI1	DRD1	GPCRLTM7
	RTEL1-TNFRSF6B	COTL1	BAHCC1
	HLTF	DBT	SLC27A3
3	GOLGA8IP	COG2	TOP1MT
	TMED10P1	TBP	DDX11L1
	TRIML1	FAM138C	USP36
	HES4	OR3A2	TDRKH
	TUBBP5	ZFR2	LYPD2
	FLJ46257	SENP6	DDX11L9

PDHB	ATPBD4	RNF213
FLT4	OR2V2	CELF3
SSR3	CRADD	TMEM71
SNORD108	NUFIP1	MIR1179
C8orf77	SLC26A2	TNRC6C
ANKRD37	MLYCD	PMF1
PRDM16	GADD45A	COL22A1
RNF224	RBM34	MIR1469
IL17REL	TCF21	SLC25A19
PFKFB4	WASH1	VPS45
GABRA1	P2RX1	ADCK5
TERC	PIP5K1C	MIR3175
SNORD109A	SLC17A5	UBE2O
ZNF517	SNORD107	KIAA0907
LOC389247	ZNF454	TSNARE1
VWA1	LIN7A	LOC100507
MIR602	NBEA	DUS1L
FAM116B	HBEGF	POGZ
PLXNB1	CPNE7	MAPK15
GABRA6	DPYD	LOC100507
SEC62	FBXO28	FN3K
SNORD109B	TCP1	SYT11
KIAA1875	MIR4665	ZNF707
HELT	P2RX5	C15orf38-A
NADK	HMHA1	ENGASE
RNF208	SNORD50A	RPRD2
PIM3	PAR1	BREA2
PRKAR2A	C5orf60	MIR3529
GABRB2	SOCS2	MRPL38
TM4SF4	C13orf15	SMG5
SNORD115-1	DUSP1	FAM83H
C8ORFK29	IL17C	MIR4714
LOC401164	DR1	ASPSCR1
MMEL1	ABCB10	SNAPIN
LOC100129722	TCP10	LOC286094
ODF3B	PAFAH1B1	CARD14
PRKCD	TIMM13	RUSC1
GABRG2	FILIP1	ZNF252
TRPC1	LOC145845	MFSD11
WHAMMP3	ZNF879	CA14
SPATC1	RASSF9	TMED10P1
FAM92A3	MED4	C17orf62
OR4F5	EBF1	C1orf43
FAM157B	ANKRD11	C8orf77
SYCE3	S1PR1	ARMC7
PTPRG	TAF5L	CHTOP
GABRP	TCTE3	C8orf31

CLRN1	SERPINF1	CHMP6
POTEB	DAZAP1	LCE2B
C8orf82	NDUFAF4	ZFP41
C4orf47	TMCO5A	RHBDF2
LINC00115	MIR340	SLC39A1
LOC100289341	NUAK1	GPIHBP1
LOC100128946	DNAJC15	FN3KRP
QARS	EGR1	LAMTOR2
GLRA1	OSGIN1	NRBP2
ZIC1	CELSR2	C17orf101
SNORD64	DISC2	TMOD4
LRRC24	DYNLT1	ZNF517
DUX4L4	PER1	ZNF750
MORN1	SNORD37	CERS2
MIR3621	CYB5R4	KIAA1875
LOC100144603	CSNK1A1P	NUP85
RPL29	SNORD95	CRNN
GM2A	NR1H4	C8ORFK29
KCNAB1	ALG5	MYO15B
PAR4	ETF1	ZBTB7B
SCXB	GINS2	SPATC1
FRG2	EXTL2	C17orf70
GLTPD1	DISC1	APH1A
MIR3201	THBS2	FLJ43860
ATXN7	PFAS	TSPAN10
GRK6	TJP3	PLEKHO1
B3GALNT1	UBE2J1	MAFA
PAR-SN	OTUD7A	QRICH2
HEATR7A	SNORD96A	OAZ3
SLED1	PLXNC1	MIR30B
OR4F16	PHF11	CBX2
MIR4535	F12	DPM3
SEMA3F	TRAPPC2L	MIR30D
GPX3	F3	FBF1
TNFSF10	CNIH4	MRPS21
LOC348120	TNFAIP3	C8orf82
MIR939	PFN1	UNK
FLJ38576	ITGB1BP3	ADAMTSL4
CCNL2	PHIP	LRRC24
SMARCC1	SPRED1	TRIM47
GRIA1	ZFP62	CRCT1
GMPS	CKAP4	ZFAT-AS1
GOLGA8E	UFM1	MAFG-AS1
SCXA	FABP6	GON4L
DUX4L6	BCMO1	LINC00051
TAS1R3	FCGR1A	OTOP2
NEK4	PYCR2	C1orf56

GRM6	TPD52L1	C8orf73
SLC33A1	PITPNA	CYGB
OR4M2	SLC39A3	MSTO1
MIR1234	AKIRIN2	SCXB
DUX4L5	PGBD4	RNF157
ATAD3B	CBY3	GOLPH3L
TCTA	METAP2	MIR661
HK3	SOHLH2	C1QTNF1
CHST2	FAT2	YY1AP1
OR4N3P	NECAB2	HEATR7A
DUX4L3	FCGR1B	PPP1R27
PLEKHN1	TRIM17	UBE2Q1
TKT	UTRN	OC90
HMMR	PLD2	METTL23
TSC22D2	ZBTB7A	FAM63A
HERC2P2	AKIRIN2-A	LOC73177
LOC728175	LPCAT4	SLC38A10
C1orf170	FAM153C	ASH1L
TNNC1	KERA	MIR937
HNRNPAB	ENOX1	CANT1
P2RY14	FBN2	LENEP
NF1P2	KLHDC4	MIR939
DUX4L2	FMO5	USH1G
KIAA1751	ARID4B	SLC50A1
UBA7	EZR	LOC10012
HNRNPH1	SERPINF2	TBC1D16
SMC4	FZR1	CDC42SE1
HERC2P9	DDX43	SCXA
LOC731424	C15orf55	AFMID
LOC115110	LOC72855	UBQLN4
USP4	NUDT4	CCDC166
HRH2	RCBTB1	MGAT5B
PDCD10	FGF1	OTUD7B
WHAMMP2	DEF8	LOC10013
CLDN24	GBP1	C17orf56
ACAP3	EGLN1	RAB25
UQCRC1	VIP	LOC10028
NDST1	POLR2A	B3GNTL1
RNF13	SIRT6	PGLYRP4
LOC503519	FAM46A	MIR1234
LOC100288255	LOC28371	RBFOX3
UBE2J2	AACSP1	RHBG
WNT5A	UHRF1BP1	LOC10050
HTR4	NUDT15	CD300LF
SLITRK3	FGFR4	ATP8B2
CHEK2P2	BANP	MIR4472-1
MIR1305	GBP2	NOTUM

	PUSL1	KIAA1383	PBXIP1
	IFRD2	STL	MIR4664
	IL12B	PSMB6	TMC8
	NLGN1	PIAS4	S100A14
	LOC646214	TMEM30A	STRA13
9534	MIR3945	GOLGA8G	GATAD2B
	B3GALT6	LOC72967{	LRRC45
	MAPKAPK3	KIAA1033	CGN
	ITK	KIAA1704	TRIM65
	PLCH1	FOXI1	ZNF687
	CXADRP2	ZDHHC7	UNC13D
	LOC100506229	GBP3	HCN3
	TPRG1L	SUSD4	MYADML2
0417	SEMA3B	EPM2A	PRUNE
	KCNMB1	RPA1	NPB
	TNIK	LSM7	SEMA4A
	LOC646278	LMBRD1	FADS6
	FAM213B	SNORD108	MRPL9
	SLMAP	OR4F29	C17orf28
	LCP2	MGAT4C	INTS3
	U2SURP	FAM48A	TSEN54
2062	REREP3	FLT4	SCNM1
	ACTRT2	CENPN	FAM100B
	MANF	GFI1	TNFAIP8L2
	LTC4S	HEATR1	TMEM235
	WWTR1	STX7	C1orf54
	LOC653061	RPL26	CCDC57
	MIB2	MBD3	PAQR6
	ACOX2	KCNQ5	HEXDC
2287	MFAP3	SNORD109	TRIM46
	TIPARP	LOC10013;	SLC26A11
	LOC653075	UTP20	TARS2
	SAMD11	CYSLTR2	ENDOV
	BAP1	GABRA1	FLAD1
	MGAT1	C16orf61	C17orf89
	ARHGEF26	GJA5	SNX27
	SNORD116-19	ENAH	LINC00482
3331	LOC148413	PEX3	ANP32E
	HYAL3	SHBG	TMEM105
	MSX2	PCSK4	HORMAD1
	PCOLCE2	RARS2	METRNL
	GOLGA6L6	SNORD109	TMEM79
	CCDC27	LOC10013;	RAB37
	CADPS	MRPL42	LCE3D
	NPM1	SPRYD7	ENPP7
3445	GPR160	GABRA6	AQP10
	LOC727924	JPH3	C17orf90

	CALML6	GJA8	PYGO2
	HYAL2	NUP133	CCDC137
	PDE6A	RNASET2	NUP210L
	SERP1	SLC2A4	ARL16
	GOLGA8C	C19orf24	MEX3A
	C1orf86	C6orf162	OTOP3
612	HESX1	SNORD115	C1orf85
	PDGFRB	LOC10013	FAM195B
	GOLIM4	CCDC59	PGLYRP3
	PWRN1	COG6	ZACN
	ATAD3C	GABRB2	THEM4
	BSN	KIAA1609	AATK-AS1
	MAPK9	GCLM	GABPB2
	GPR171	ERO1LB	FLJ43681
	PWRN2	STX11	TCHHL1
	LOC254099	SOX15	FLJ45079
	RRP9	PLEKHJ1	RPTN
	PROP1	LYRM2	TEX19
	SCHIP1	FMN1	TDRD10
	SNORD116-1	MIR1229	MXRA7
	TTLL10	HCFC2	SHE
	CACNA2D2	NAA16	FLJ90757
	RARS	GABRG2	DCST2
	NMD3	WFDC1	MIR338
	SNORD116-2	GNAI3	C1orf182
	FAM41C	FMN2	C17orf109
	VPRBP	SYNJ2	S100A16
	RPS14	VAMP2	LINC00338
	COMMD2	BTBD2	CREB3L4
	SNORD116-3	SNX14	SCARNA16
069	C1orf174	SNORD64	C1orf51
	IP6K1	MIR4638	SNORD1A
	SGCD	SYCP3	LELP1
	RSRC1	RNASEH2B	SNORD1B
	SNORD116-4	GABRP	DCST1
	KLHL17	MTHFSD	SNORD1C
	PSMD6	GNAT2	BNIPL
	SLC6A7	ADCK3	MIR636
	SELT	VNN2	SPRR4
	SNORD116-5	TP53	MIR657
	TMEM240	CCDC94	LCE4A
	PARP3	FAM135A	PRCD
	SLC34A1	PAR4	KRTCAP2
	P2RY13	LOC10085	C17orf110
	SNORD116-6	CHST11	CRTC2
	TMEM52	KIAA0226L	LOC100131
	RBM6	GDF9	LCE5A

	SLIT3	DBNDD1	TEN1
	GPR87	GNG5	RIIAD1
	SNORD116-7	GJC2	C17orf99
	AGRN	VNN1	THEM5
	RBM5	UBE2G1	LOC100287
	SNCB	NCLN	RUSC1-AS1
	MYNN	RRAGD	LOC100294
	SNORD116-8	PAR-SN	S100A7A
	FAM132A	CCDC53	MIR1250
	NME6	PROSER1	LINGO4
	SPARC	GFRA3	MIR4316
	EIF5A2	KLHL36	RXFP4
	SNORD116-9	GSTM1	MIR3065
	HES5	SIPA1L2	LCE1A
	TRAIP	TAAR5	MIR3186
	STK10	TRPV1	LCE1B
	SUCNR1	SPPL2B	MIR3678
	SNORD116-10	BACH2	LCE1C
	LOC388588	MIR211	MIR3615
	ARIH2	CCDC41	LCE1D
	ZNF354A	CDADC1	LOC100507
	LXN	GLRA1	LCE1E
	SNORD116-11	FBXO31	LOC100507
	RNF223	GSTM2	LCE1F
	NPRL2	ZP4	LOC100507
	TCOF1	MAP7	LCE2A
	CCNL1	YWHAE	LOC100507
	SNORD116-12	SHD	LCE2C
	MIR200A	SMAP1	TEN1-CDK3
	CSPG5	HERC2P9	LCE2D
	TTC1	NT5DC3	MIR4739
	PLSCR2	CAB39L	LCE3A
[illegible]	SNORD116-13	GM2A	MIR4738
	MIR200B	CMIP	LCE3B
	USP19	GSTM3	MIR4740
	STC2	RHOU	LCE3C
	PLSCR4	LATS1	MIR4730
	SNORD116-14	ZNF232	LCE3E
	FLJ42875	WDR18	LOC100653
[illegible]	SLC38A3	C6orf164	LYSMD1
	ADAM19	WHAMMP[illegible]	HRNR
	NCEH1	PARPBP	FLG2
	SNORD116-15	COG3	C1orf189
	ANKRD65	GRK6	VHLL
	CYB561D2	CDT1	MIR9-1
	FGF18	GSTM4	KPRP
	IFT80	ACBD3	LCE6A

SNORD116-16	TAAR2	PRR9
MIR429	OR1A1	LOC64567[illegible]
TMEM115	REXO1	POU5F1P4
SQSTM1	OGFRL1	S100A7L2
SLC7A14	GOLGA8B	SCARNA4
SNORD116-17	SLC6A15	SNORA42
FAM138F	SETDB2	MIR554
RPP14	GPX3	MIR555
ATP6V0E1	MAP1LC3B	MIR92B
MFSD1	GSTM5	MIR190B
SNORD116-18	ARV1	C1orf68
LOC643837	TAAR3	MSTO2P
FAM107A	OR1D5	LOC10013[illegible]
PTTG1	ZNF77	C2CD4D
FNDC3B	MANEA	MIR4257
SNORD116-20	LOC50351[illegible]	MIR4258
TMEM88B	RIC8B	LOC10050[illegible]
WDR6	KBTBD7	PMF1-BGL[illegible]
PDLIM7	GRIA1	TNFAIP8L2[illegible]
P2RY12	DYNLRB2	
SNORD116-21	GTF2B	
C1orf233	AIDA	
RASSF1	MED23	
CNOT8	OR1E1	
VEPH1	C19orf29	
SNORD116-22	LINC00472	
FAM138A	LOC646278	
NISCH	APPL2	
HAND1	EBPL	
ZBBX	NR3C1	
SNORD116-23	HSDL1	
WASH7P	HMGCS2	
TREX1	JMJD4	
MED7	QKI	
LRRC31	OR1E2	
SNORD116-24	CELF5	
MIR551A	KHDC1	
TUSC2	LOC653075	
ADAMTS2	DRAM1	
PHC3	KBTBD6	
SNORD116-25	GRM6	
CDK11A	CRISPLD2	
TWF2	HSD3B1	
CLINT1	C1orf35	
ARL14	AKAP7	
SNORD115-2	OR1G1	
SLC35E2B	TLE6	

	DHX30	GPR63
[illegible]	MAML1	ANP32AP1
	SPATA16	STAB2
	SNORD116-26	STARD13
	LOC728716	HARS
	SCAP	SPIRE2
	GFPT2	HSD3B2
	EIF2A	TTC13
	SNORD116-27	TBPL1
	LOC729737	OR3A3
	LAMB2P1	LPPR3
	G3BP1	SPACA1
	ZIC4	SNORD116-19
	SNORD115-3	VEZT
	OR4F29	EPSTI1
	RAD54L2	HINT1
	TNIP1	ZNF469
	HPS3	IGSF3
	SNORD115-4	PGBD5
	LOC100129534	WTAP
	STAB1	SLC25A11
	GNB2L1	ZNF556
	ARPM1	SH3BGRL2
	SNORD115-5	PWRN1
	LOC100130417	GOLGA2P5
	FAM208A	ARL11
	SLU7	HK3
	GFM1	CENPBD1
	SNORD115-6	CYR61
	LOC100132062	HHIPL2
	KLHL18	AKAP12
	RGS14	DOC2B
	EGFEM1P	FAM108A1
	SNORD115-7	GJA10
	LOC100132287	PWRN2
	NAT6	SCYL2
S1	CPLX2	WDFY2
	TM4SF18	HMMR
	SNORD115-8	ZNF276
	LOC100133331	IL12RB2
.	ABHD14A	PCNXL2
	C5orf4	KIAA0408
	MED12L	SCARF1
	SNORD115-9	DOHH
	LOC100133445	C6orf7
	POC1A	SNORD116-1
	FAM114A2	POLR3B

CLRN1-AS1	LINC00284
SNORD115-10	HNRNPAB
LOC100133612	KCNG4
NDUFAF3	JAK1
BTNL3	WDR26
OTOL1	PHACTR2
SNORD115-11	TNK1
DDX11L1	KLF16
PTPN23	UBE2CBP
LMAN2	SNORD116-2
FAM194A	FGD6
SNORD115-12	CSNK1A1L
TTC34	HNRNPH1
DNAH1	SDR42E1
SPINK5	KCNA2
PPM1L	TRIM11
SNORD115-13	BCLAF1
LOC100288069	TNFSF13
ERC2	DOT1L
SOX30	MRAP2
WDR49	SNORD116-3
SNORD115-14	NDUFA12
MIR4251	FAM216B
APPL1	HRH2
MGAT4B	PKD1L2
LRRC34	KCNA3
SNORD115-15	MIXL1
GNL3	UST
B4GALT7	TNFSF12
C3orf55	KISS1R
SNORD115-16	RWDD2A
SPCS1	SNORD116-4
SYNPO	ANKS1B
C3orf79	LACC1
SNORD115-17	HSD17B4
SETD2	RNF166
RNF44	KCNA10
SPTSSB	C1orf124
SNORD115-18	TRDN
RBM15B	TM4SF5
ABLIM3	CREB3L3
DHX36	KLHL32
SNORD115-19	SNORD116-5
GMPPB	CHPT1
HMGXB3	LINC00330
RPL22L1	HSPA4
SNORD115-20	C16orf46

ARHGEF3	KCNC4
TBC1D9B	OBSCN
LOC201651	CITED2
SNORD115-21	USP6
C3orf18	LMNB2
ATP10B	MB21D1
C3orf58	SNORD116-6
SNORD115-22	TMCC3
SHISA5	ST13P4
N4BP3	HSPA9
NAALADL2	DNAAF1
SNORD115-23	KCND3
ZMYND10	NTPCR
FAF2	HBS1L
SLC9A9	RABEP1
SNORD115-25	RAX2
TEX264	RIPPLY2
WWC1	SNORD116-7
IGSF10	NTN4
SNORD115-26	DGKH
CCDC72	NDST1
FBXW11	SPATA2L
C3orf33	LEPR
SNORD115-29	KIAA1804
ZNF589	PDE10A
LARP1	KCNAB3
TRIM59	MUM1
SNORD115-30	IRAK1BP1
HEMK1	SNORD116-8
ZNF346	ACTR6
LOC339894	CCDC122
SNORD115-31	HTR4
IP6K2	C16orf55
GEMIN5	MOV10
LRRIQ4	C1orf198
SNORD115-32	RAB32
SFMBT1	AURKB
CCDC69	ATCAY
SAMD7	B3GAT2
SNORD115-33	SNORD116-9
NCKIPSD	NUP37
TSPAN17	STOML3
AADACL2	IK
SNORD115-34	ZC3H18
PHF7	MSH4
OR4F3	DISP1
GPR149	KATNA1

SNORD115-35	RPH3AL
TLR9	MIDN
HAVCR1	C6orf57
PAQR9	SNORD116-10
SNORD115-36	GNPTAB
P4HTM	FAM194B
CYFIP2	IL3
PLSCR5	CDYL2
SNORD115-37	NFIA
IL17RD	WNT3A
PRELID1	FGFR1OP
ANKUB1	FXR2
SNORD115-38	R3HDM4
C3orf75	CD109
IL17B	SNORD116-11
LEKR1	ACSS3
SNORD115-39	SPERT
QRICH1	IL4
MAT2B	SLC38A8
TMEM212	NGF
SNORD115-40	HIST3H2A
PXK	SCAF8
MRPL22	MPDU1
LOC401093	C19orf6
SNORD115-41	PM20D2
FEZF2	SNORD116-12
TLX3	CEP290
C3orf80	DLEU7
SNORD115-42	IL5
DALRD3	SLC22A31
KCNIP1	NHLH2
MIR15B	SNAP47
SNORD115-43	TAB2
PBRM1	SPAG7
ZNF354C	REEP6
MIR16-2	SRSF12
SNORD115-44	SNORD116-13
CHDH	MTERFD3
RPL26L1	FAM124A
TMEM14E	IL9
SNORD116-28	FLJ30679
IL17RB	NOTCH2
C5orf45	C1orf96
LOC646168	SASH1
SNORD116-29	ACAP1
CACNA2D3	IZUMO4
DCTN4	C6orf221

LOC646903	SNORD116-14
SNORD115-48	GLT8D2
DCP1A	CTAGE10P
DDX41	IL12B
PA2G4P4	LOC146513
SNORD115-24	NRAS
GLT8D1	DNAH14
NOP16	SYNE1
LOC647107	KIAA0753
SNORD115-27	SCAMP4
SEMA3G	C6orf165
HMP19	SNORD116-15
IQCJ	USP44
SNORD115-28	SLC25A30
ADAMTS9	IL13
UIMC1	ZFPM1
SCARNA7	ROR1
SNORD115-45	C1orf131
PCBP4	HEY2
RAB24	SGSM2
MIR551B	ADAT3
SNORD115-47	PRSS35
ABHD6	SNORD116-16
NEURL1B	SLC41A2
MIR569	SUGT1P3
LOC100128714	IRF1
LRTM1	ADAD2
FAM193B	OVGP1
LOC730091	EDARADD
HERC2P7	HEBP2
C3orf14	CLEC10A
TMED9	ZNF554
WWTR1-AS1	LCA5
GOLGA8F	SNORD116-17
SELK	LRRIQ1
CDHR2	SIAH3
LOC100128164	ITK
GOLGA8DP	MGC23284
RNF123	PDE4B
CCDC99	MRPL55
TIPARP-AS1	CCDC28A
ULK4P2	MYBBP1A
KIF9	GRIN3B
THG1L	C6orf163
LOC100289361	SNORD116-18
LOC100288637	C12orf26
CCDC71	KCNRG

	GALNT10	KCNMB1
4	MIR548H2	LINC00311
	LOC100289656	PDZK1
	NT5DC2	HIST3H2BB
	NHP2	MTHFD1L
	LOC100498859	PRPF8
	MIR4509-1	MRPL54
	SLC26A6	MMS22L
	RBM22	SNORD116-20
	MIR3919	C12orf23
	MIR4509-2	FREM2
	CAMKV	KCNN2
	RNF130	ZNF778
	IQCJ-SCHIP1	PGM1
	MIR4508	BROX
3255	CCDC51	MOXD1
	NMUR2	KIF1C
5	LOC100507389	ZNF57
	MIR4509-3	DPPA5
	THOC7	SNORD116-21
	KIAA1191	C12orf29
	ARHGEF26-AS1	KCTD4
	MIR4715	LCP2
	WDR82	ACSF3
7	ERGIC1	PIN1P1
	LOC100507537	SLC35F3
	ID2B	IPCEF1
	CLK4	GABARAP
	MIR4789	JSRP1
	ATRIP	EYS
	ODZ2	SNORD116-22
	NICN1	DEPDC4
	CNOT6	NHLRC3
	MON1A	LECT2
	C5orf54	LINC00304
	ABHD14B	PRKAB2
	NSD1	B3GALNT2
	UCN2	TIAM2
	GMCL1P1	NLRP1
7	RFT1	MOB3A
	RMND5B	GJB7
	ACTR8	SNORD116-23
	RANBP17	CCDC38
	GPR62	SERP2
	PCYOX1L	LMNB1
5	FAM3D	SNAI3
	CCNJL	PRKACB

	IQCF1	CNIH3
	SH3TC2	C6orf123
	GLYCTK	ARHGEF15
	PANK3	MFSD12
	PPM1M	SNHG5
	SAP30L	SNORD116-24
[illegible]	C3orf49	C12orf45
	BTNL8	SERTM1
	SNTN	LOX
	DOK3	FAM92B
989	SYNPR	PKN2
	ZFP2	EXOC8
	C3orf45	FBXO5
331	RUFY1	RAP1GAP2
	ASB14	GIPC3
	CPEB4	C6orf147
	PRICKLE2	SNORD116-25
013	PRR7	NEDD1
	C3orf67	LINC00547
	OR4F16	LTC4S
814	KCTD6	CTU2
	FBXO38	PSMA5
	KLHDC8B	RNF187
	TRIM7	RGS17
085	DNAH12	CAMTA2
	TIGD6	PLK5
	PDE12	MIR30A
1	MXD3	SNORD115-2
	FAM116A	SLC9A7P1
	THOC3	LINC00548
	FBXW12	SMAD5
122	SPINK7	PABPN1L
	CCDC66	PTGER3
[illegible]	TRIM52	C1orf55
	FLJ39534	PDE7B
	HAVCR2	KDM6B
	LOC285401	CIRBP-AS1
	AGXT2L2	MIR30C2
229	CCDC36	SNORD116-26
	C5orf62	IKBIP
[illegible]	SPATA12	MIR15A
	UNC5A	MFAP3
	C3orf62	LOC400548
	TRIM41	PTGFR
	TMEM110	C1orf65
	BOD1	DLL1
	AMIGO3	ZZEF1

	COL23A1	C19orf25
	CDHR4	OOEP
	TIMD4	SNORD116-27
	FAM212A	SPIC
	ZNF300	MIR16-1
	IQCF2	MGAT1
	MYOZ3	LOC400550
	IQCF5	PTGFRN
C1	UBTD2	IBA57
	MUSTN1	MRPL18
	SCGB3A1	KIAA0664
	IQCF3	ATP8B3
	SFXN1	MCART3P
)	MIRLET7G	SNORD115-3
	GPRIN1	ANO4
	MIR135A1	TSC22D1-AS1
	PWWP2A	MSX2
	MIR191	LOC400558
	C1QTNF2	ABCD3
	TMEM89	LIN9
	SCGB3A2	CLDN20
	IQCF6	SMG6
	ZNF354B	DIRAS1
	C3orf78	TSG1
	C5orf47	SNORD115-4
	MIR425	AMDHD1
	PPARGC1B	TTL
	SPINK8	NDUFA2
	C5orf58	C16orf74
	C3orf71	RABGGTB
	OR2Y1	ZNF678
	LOC646498	NOX3
	AFAP1L1	WSCD1
	SNORD19	ZNF555
	GRPEL2	SNORD50B
	SNORD69	SNORD115-5
	LSM11	TSPAN19
	MIR548A2	SERPINE3
5795	ZNF300P1	NEUROG1
	ESRG	SNORD68
	NUDCD2	RAP1A
5	SNORD19B	PRSS38
	UBLCP1	HDDC2
5619	C3orf74	CTDNEP1
5810	SLC36A2	ANKRD24
	NRADDP	KHDC1L
	SPINK13	SNORD115-6

	BSN-AS2	LOC144481
	C5orf41	SNORA31
5865	LOC100287879	NPM1
F695	BTNL9	LOC727710
	MIR1226	SNORD21
	PPP1R2P3	LOC339535
	MIR711	TFB1M
	FAM71B	C17orf81
M1	MIR4271	C19orf26
	RNF145	LOC100288198
	MIR3938	SNORD115-7
	HIGD2A	LOC144486
	IQCF4	MIR621
	FAM153B	NPY6R
	LOC100506994	LOC732275
	LOC202181	RPE65
	LOC100507062	IRF2BP2
	SLC36A1	AIG1
	ADAMTS9-AS2	SHPK
	EIF4E1B	CSNK1G2-AS1
	ABHD14A-ACY1	MIR2113
	RASGEF1C	SNORD115-8
	TMEM110-MUSTN1	KRT19P2
	LOC257358	CCDC169
	MIR4793	PCDH1
	EFCAB9	LOC100128881
	MIR4787	RPL5
	SH3PXD2B	MIA3
	MIR4443	SNX9
	LOC285593	AIPL1
	LOC100652759	C19orf77
	FAM153A	MIR548H3
	LUST	SNORD115-9
	ARL10	TMTC2
	LOC285626	TPT1-AS1
	LOC285627	PCDHGC3
	LOC285629	LOC100129617
	SLC36A3	SORT1
	KIF4B	C1orf95
	OR2V2	VTA1
2	ZNF454	RNF167
	C5orf60	ADAMTSL5
	LOC340037	MIR4282
	ARSI	SNORD115-10
	PFN3	TMTC3
	ZNF879	MIR548F5
	IRGM	PDE6A

FBLL1	LOC100130015
NIPAL4	SARS
C5orf25	FAM89A
FLJ38109	BRP44L
C5orf46	SENP3
ARHGEF37	TMPRSS9
SPINK6	LOC100506804
MIR103A1	SNORD115-11
MIR143	C12orf50
MIR145	MIR320D1
MIR146A	PDGFRB
MIR218-2	LOC100287036
SPINK14	SLC16A1
FNDC9	CAPN8
FLJ16171	HECA
MIR340	OR1A2
SNORD95	NDUFS7
SNORD96A	MIR4464
LOC643201	SNORD115-12
SPINK9	ALDH1L2
ZFP62	MIR4305
CBY3	PFDN1
FAM153C	MIR1910
SNORA74B	STXBP3
MIR585	C1orf31
MIR143HG	IL20RA
LOC728554	SNORA67
AACSP1	C19orf35
LOC729678	MIR4643
OR4F29	SNORD115-13
FAM196B	SLC5A8
LOC100132062	MIR3613
LOC100132287	PGGT1B
LOC100133331	MIR3182
C5orf52	SYCP1
LOC100268168	FAM177B
MIR1229	UNC93A
MIR103B1	PELP1
MIR3142	ONECUT3
MIR3141	SNORD115-14
MIR4281	FAM71C
MIR3912	OR7E37P
LOC100507387	PITX1
MIR4634	C16orf95
MIR4638	TAF13
MIR378E	ZNF847P
LOC100859930	MTRF1L

RANGRF
MEX3D
SNORD115-15
RMST
SPG20OS
POU4F3
MIR4720
TBX15
TRIM67
AHI1
TIMM22
C19orf29-AS1
SNORD115-16
C12orf12
LOC100507240
PPIC
MIR4722
TGFBR3
H3F3AP4
RMND1
TAX1BP3
LINGO3
SNORD115-17
SLC17A8
LOC100509894
PPP2CA
TSHB
DUSP5P
PHF10
MINK1
MIR637
SNORD115-18
GNN
CCDC169-SOHLH2
PPP2R2B
USP1
SNRPD2P2
VNN3
GEMIN4
C19orf71
SNORD115-19
TCP11L2
MIR4703
MAPK9
VCAM1
RPS7P5
C6orf70

MED31
LOC100288123
SNORD115-20
LOC256021
LOC100616668
PROP1
WNT2B
SNORA14B
ECHDC1
DERL2
MIR1909
SNORD115-21
POC1B
PURA
CSDE1
LOC100130093
AGPAT4
GLOD4
MIR1227
SNORD115-22
OTOGL
RARS
EVI5
LOC100130331
TULP4
YBX2
MIR3187
SNORD115-23
GAS2L3
RPS14
CDC7
LOC100287814
HYMAI
ANKFY1
MIR4321
SNORD115-25
LINC00485
SGCD
HIST2H2AA3
LGALS8-AS1
GPR126
INPP5K
SNORD115-26
LOC338758
SKP1
HIST2H2AC
MIR1182

KIAA1244
FAM64A
SNORD115-29
C12orf74
SLC6A7
HIST2H2BE
MIR1537
NHSL1
XAF1
SNORD115-30
PTPRQ
SLC12A2
HIST2H4A
LINC00184
PLEKHG1
C17orf59
SNORD115-31
C12orf42
SLC34A1
BCAR3
TSNAX-DISC1
ARID1B
GPR172B
SNORD115-32
C12orf75
SLC22A4
TTF2
MIR320B2
TMEM181
WRAP53
SNORD115-33
MKRN9P
SLC22A5
ITGA10
MIR3620
ZBTB2
RNMTL1
SNORD115-34
MIR135A2
SLIT3
LMO4
LOC100506795
C6orf115
VPS53
SNORD115-35
C12orf37
SNCB

CDC14A
LOC100506810
PBOV1
C17orf85
SNORD115-36
PLEKHG7
SNX2
RTCD1
MIR4753
TRMT11
TSR1
SNORD115-37
NUDT4P1
SPARC
FPGT
MIR4666A
PERP
DHX33
SNORD115-38
MIR331
SPINK1
PEX11B
MIR4671
SMOC2
PLSCR3
SNORD115-39
EID3
SPOCK1
FUBP1
MIR4427
ALDH8A1
NLGN2
SNORD115-40
CLLU1OS
STK10
BCL10
MIR4742
ULBP3
ZBTB4
SNORD115-41
CLLU1
TAF7
DIRAS3
OR2A4
CXCL16
SNORD115-42
MIR492

	TCF7
	SLC16A4
	C6orf211
	TRAPPC1
	SNORD115-43
	LOC643339
	ZNF354A
	SRSF11
	ZDHHC14
[illegible]	ALOXE3
	SNORD115-44
	LOC643770
	TCOF1
	CD101
	ADGB
	SRR
	SNORD116-28
	SNORA53
[illegible]	TGFBI
	SEPT15
	FRMD1
	NXN
M34	SNORD116-29
	MIR617
	TTC1
	ZRANB2
	AGPAT4-IT1
	MIS12
	SNORD115-48
	MIR618
	UBE2B
	ARHGAP29
	C6orf208
	METTL16
	SNORD115-24
	LOC728084
	UBE2D2
	SEC22B
[illegible]	C6orf97
	PHF23
	SNORD115-27
	C12orf73
[illegible]	VDAC1
	CHD1L
	MYCT1
	FAM57A
[illegible]	SNORD115-28

	LOC100128191
	WNT8A
	CLCA3P
	ULBP2
1	CTC1
	SNORD115-45
	LOC100287944
	NME5
	CLCA2
	ULBP1
	NDEL1
	SNORD115-47
	MIR1252
	PDLIM4
	HS2ST1
	LPAL2
	PITPNM3
	LOC100128714
	MIR1827
	STC2
	PDE4DIP
	PPP1R14C
	TMEM93
	GOLGA8F
	MIR1251
	EIF4EBP3
	DNAJC6
[illegible]	RNF146
	RILP
	ULK4P2
	MIR4303
	PCDHGB4
	LRIG2
	SF3B5
	TEKT1
	LOC100288615
	MIR3685
	CDC23
[illegible]	LPPR4
	TAAR8
	GSG2
	LOC100288637
	MIR3652
[illegible]161	ADAM19
	SV2A
	RSPH3
	ZMYND15

1	LOC100289656
	MIR3922
	FGF18
	RBM8A
	TTLL2
	CAMKK1
	MIR1233-1
	LOC100505978
	HDAC3
	INSL5
	FBXO30
	RPAIN
	MIR1233-2
	POC1B-GALNT4
	SQSTM1
	PIGK
	L3MBTL3
	TMEM107
	MIR3942
	MIR4699
	P4HA2
	TSPAN2
	FAM120B
	LSMD1
	LOC100507466
	MIR548AL
	ATP6V0E1
	INADL
	FNDC1
	NEURL4
	MIR4509-1
	ATG12
	SF3B4
	RSPO3
	ZNF594
	MIR4509-2
	PTTG1
L5	BCAS2
	LRP11
	HES7
	MIR4510
	PDLIM7
	WARS2
	LTV1
	SPATA22
	MIR4509-3
	CNOT8

CEPT1
SERAC1
TXNDC17
MIR4715
HAND1
PIAS3
REPS1
MIR22HG
TMCO5B
MED7
VAV3
PPIL4
TMEM88
MYOT
HBXIP
LINC00473
SAT2
ADAMTS2
IFI44
ARHGAP18
SMYD4
NRG2
POLR3C
SYTL3
CNTROB
CXCL14
TXNIP
FAM54A
RPL29P2
H2AFY
AP4B1
SFT2D1
CYB5D1
SMAD5-AS1
PHTF1
TMEM200A
OVCA2
RNF14
AHCYL1
C6orf72
KRBA2
SNCAIP
WDR3
IL22RA2
SLC43A2
CLINT1
MAN1A2

	C6orf192
	CYB5D2
	PCDHGA8
	IFI44L
	TAGAP
	C17orf49
	PCDHA9
	DNAJB4
	ADAT2
	ZFP3
	MATR3
	ADAM30
	CLVS2
	GGT6
[illegible]3	MAML1
	CD160
	TAAR9
	SPNS2
	KIAA0141
	GLMN
	TAAR1
	WDR81
	JAKMIP2
	HHLA3
[illegible]5	STXBP5
	DNAH2
	DDX46
	DDX20
	NCOA7
	RTN4RL1
	GFPT2
	CLCA4
	HINT3
[illegible]581	ODF4
	SLC23A1
	MTF2
[illegible]347	PACRG
	KCTD11
	GNPDA1
	NTNG1
	RAET1E
[illegible]5	TRPV3
	SRA1
	WDR47
	LOC153910
	SLC16A11
	RAD50

	USP33
938	ZC2HC1B
	FBXO39
	KIF20A
	CLCC1
	CNKSR3
	SLC16A13
	G3BP1
	SLC35D1
	RAET1L
576	C17orf74
	APBB3
	LPHN2
	SAMD3
	SPNS3
	TNIP1
	KIAA1107
	MGC34034
	C17orf61
782	GNB2L1
	ITGB3BP
	SLC2A12
	BCL6B
	SLU7
	SLC35A3
	LOC154092
	LOC284009
	RGS14
FFL	LRRC8B
	PNLDC1
	VMO1
	SEC24A
	LPAR3
	RNF217
	LOC284023
	CPLX2
	DDAH1
	NKAIN2
	LINC00324
	C5orf4
	NBPF14
	LOC154449
[illegible]	SLC13A5
	FAM114A2
	RWDD3
	OLIG3
	TMEM102

BRD8
ZZZ3
TXLNB
TUSC5
TCERG1
SERBP1
DACT2
LOC339166
BTNL3
PTPN22
C6orf118
TMEM95
HNRNPA0
PHGDH
OSTCP1
SMTNL2
LMAN2
AK5
C6orf191
ALOX15P1
SPINK5
SNORA66
WDR27
FAM101B
SOX30
SNORD45B
SHPRH
SPEM1
KIF3A
SNORD45A
LINC00326
GLTPD2
MGAT4B
FOXD3
LOC285740
INCA1
B4GALT7
CHIA
LOC285796
SCIMP
SYNPO
RNF115
PRR18
C17orf100
RNF44
ANGPTL3
TAAR6

OR3A4P
ABLIM3
GPSM2
SLC35D3
SLC25A35
HMGXB3
ALG6
ZC3H12D
C17orf97
TBC1D9B
SLC25A24
ECT2L
MED11
ARHGAP26
DNTTIP2
NUP43
MIR132
FSTL4
TMED5
C6orf58
MIR195
ATP10B
BOLA1
RAET1G
MIR212
N4BP3
TNNI3K
HMGA1P7
MIR22
SEPT8
SH3GLB1
SUMO4
TNFSF12-TNFSF13
FAF2
HAO2
CENPW
RNASEK
WWC1
ACP6
C6orf174
MIR324
FBXW11
HOOK1
C6orf120
MIR497
ACSL6
SNX7

THEMIS
SLC35G6
PHF15
GPR89B
SAMD5
RNF222
LARP1
TRIM33
IYD
SNORA48
HARS2
DPH5
DKFZp451B082
SNORD10
ZNF346
GPR88
TCP10L2
OR1D4
TNFAIP8
CCDC76
LINC00242
SCARNA21
GEMIN5
L1TD1
GTF2H5
SNORD91A
PCDHGA12
RSBN1
FLJ46906
SNORD91B
LRRTM2
ZNHIT6
LOC441177
BHLHA9
CCDC69
LEPROT
SNORD101
TLCD2
PCDHB5
GIPC2
SNORD100
LOC728392
FBXL21
GDAP2
SNORA33
LOC100128288
KLHL3

	FAM46C
	LOC643623
	C17orf107
	TSPAN17
	PALMD
	CTAGE9
	LOC100130950
	OR4F3
676	FNBP1L
	LOC645434
	DBIL5P
	HAVCR1
556	ST7L
	RAET1K
	MIR1253
	SNORD63
550	PRPF38B
	MLLT4-AS1
1	LOC100306951
	SNORA74A
	LRRC8D
	SNORA20
879	MIR3183
	CYFIP2
	PRMT6
	SNORA29
	MIR4314
	PKD2L2
	RAVER2
	LOC729176
	MIR3676
	UQCRQ
	FGGY
	LOC729178
640	LOC100506388
	AFF4
	MCOLN3
	TMEM242
	LOC100506713
	PRELID1
	SLC22A15
	LOC729603
	MIR497HG
	IL17B
	RNPC3
	HGC6.3
	RNASEK-C17ORF49

	MAT2B
	LRRC40
	LOC100128176
.	C17orf61-PLSCR3
	SLC27A6
	DEPDC1
	LOC100129518
	SENP3-EIF4A1
	SNX24
994	LRIF1
	C6orf99
032	P2RX5-TAX1BP3
	MRPL22
062	CTTNBP2NL
	LINC00271
AS2	MIR4520A
	PCDHB1
ACY1	FAM212B
	LOC100132735
MUSTN1	MIR4521
	TLX3
[illegible]	GNG12
	LOC100289495
	MIR4520B
	KCNIP1
	CCBL2
	NHEG1
	ZNF354C
	TMEM167B
	MIR1913
	TMED7
	OLFML3
	MIR3145
759	ISOC1
	FAM91A2
	MIR3918
	RPL26L1
	AMIGO1
	MIR3939
	SAR1B
	ODF2L
	MIR3668
	C5orf45
	KIAA1324
	MIR3662
	DCTN4
	LRRC7

MIR3692
MZB1
CACHD1
LOC100507203
PAIP2
MIER1
LOC100507254
CDKL3
PTBP2
LOC100507462
PCDH12
DNASE2B
LOC100507489
FAM13B
ELTD1
LOC100507557
FAM53C
HIAT1
CAHM
REEP2
RBM15
MIR4466
PRR16
DCLRE1B
MIR4465
COMMD10
WDR77
MIR4644
DDX41
DLEU2L
LOC100652739
NOP16
EPS8L3
LARS
VTCN1
CXXC5
TTLL7
HMP19
WDR78
UIMC1
RPAP2
RAPGEF6
DENND2D
KDM3B
WLS
PHAX
RPF1

RAB24
SIKE1
RBM27
TRIM45
NEURL1B
ANKRD13C
FAM193B
VANGL1
PCDHB18
ST6GALNAC5
PCDHB17
GPR61
TMED9
TM2D1
ZCCHC10
REG4
CDHR2
SYDE2
WDR55
ZNF644
ANKHD1
LRRC8C
CCDC99
SGIP1
THG1L
POLR3GL
TMCO6
PROK1
TRIM36
EFCAB7
GALNT10
PSRC1
NHP2
ATP1A1OS
RBM22
ATG4C
RNF130
FAM40A
PCDHGC5
DOCK7
PCDHGC4
ZNF697
PCDHGB7
NEXN
PCDHGB6
DNAJA1P5
PCDHGB5

NL

HENMT1
PCDHGB3
GNRHR2
PCDHGB2
GBP4
PCDHGB1
GBP5
PCDHGA11
SSX2IP
PCDHGA10
OLFM3
PCDHGA9
WDR63
PCDHGA7
MAB21L3
PCDHGA6
HIST2H3C
PCDHGA5
SLC44A3
PCDHGA4
ATXN7L2
PCDHGA3
C1orf194
PCDHGA2
ASB17
PCDHGA1
TYW3
PCDHGB8P
C1orf173
PCDHB15
LRRIQ3
PCDHB14
LRRC39
PCDHB13
C1orf87
PCDHB12
LIX1L
PCDHB11
HSD3BP4
PCDHB10
DRAM2
PCDHB9
C1orf88
PCDHB8
C1orf162
PCDHB7
SYT6

	PCDHB6
	SAMD13
	PCDHB4
	C1orf52
AS1	PCDHB3
	TMEM56
	PCDHB2
	NBPF4
	PCDHAC2
	UBE2U
	PCDHAC1
	HFE2
	PCDHA13
	ANKRD35
	PCDHA12
	SLC30A7
	PCDHA11
	MGC27382
	PCDHA10
	IL23R
	PCDHA8
	RP11-165H20.1
	PCDHA7
	DENND2C
	PCDHA6
	GBP6
	PCDHA5
	LPPR5
	PCDHA4
	FNDC7
	PCDHA3
	KANK4
	PCDHA2
	SASS6
	PCDHA1
	PPIAL4A
	VTRNA1-3
	HFM1
	VTRNA1-2
	UBL4B
	VTRNA1-1
	PDIA3P
	NMUR2
	ALG14
	FEM1C
	LINC00466
	C5orf15

NBPF11
CDC42SE2
NUDT17
TRPC7
TCTEX1D1
KIAA1191
SPAG17
ERGIC1
HIPK1
CLK4
SLC44A5
ODZ2
AKR7A2P1
CNOT6
EPHX4
ZNF608
AKNAD1
KCTD16
MCOLN2
SEMA6A
COL24A1
PCDHB16
ST6GALNAC3
HMHB1
NEGR1
C5orf54
MAGI3
NSD1
FAM19A3
SIL1
NBPF15
GMCL1P1
FAM102B
ARAP3
SYPL2
RMND5B
CYB561D1
YTHDC2
ANKRD34A
RANBP17
ZNF326
GRAMD3
BTBD8
PCYOX1L
HIST2H2AB
CCNJL
PPM1J

SH3TC2
HIST2H3A
PANK3
HIST2H2BC
SAP30L
HIST2H2BA
TXNDC15
LOC339524
BTNL8
CCDC18
DOK3
MYBPHL
ZFP2
BARHL2
RUFY1
HSP90B3P
CPEB4
NBPF7
PRR7
FAM73A
NDFIP1
LOC375010
OR4F16
GBP7
FBXO38
C1orf146
YIPF5
FAM69A
TRIM7
SLC6A17
TIGD6
NOTCH2NL
SPRY4
FLJ39739
MXD3
LOC388692
SLC4A9
UOX
SLC25A2
FRRS1
PCDHB19P
C1orf141
PCBD2
GBP1P1
PSD2
FLJ27354
THOC3

	MIR137HG
2	C5orf32
	NBPF9
	MEGF10
	MIR101-1
	SPINK7
	MIR137
	TRIM52
F4EBP3	MIR186
	HAVCR2
	MIR197
	AGXT2L2
	LHX8
	C5orf62
	C1orf180
	FCHSD1
	FLJ31662
	UNC5A
	LOC440600
	LYRM7
	BCL2L15
	TRIM41
	HIST2H2BF
	BOD1
	PGCP1
	CDKN2AIPNL
3	GEMIN8P4
	COL23A1
	RBMXL1
	TIMD4
	HIST2H4B
	ZNF300
5	SRG7
	MYOZ3
	CYMP
1	UBTD2
	LOC643441
	SCGB3A1
	LOC644242
0	PRDM6
	PPIAL4G
5	FTMT
	PPIAL4D
	SFXN1
	LOC645166
0	FNIP1
	LOC646626

	SLC35A4
	EMBP1
	GPRIN1
	SRGAP2P2
	PWWP2A
	LOC648740
	C1QTNF2
	NBPF6
	MARCH3
	PPIAL4B
	LEAP2
	LOC653513
	SCGB3A2
[illegible]	GPR89A
	ZNF354B
[illegible]	PPIAL4C
[illegible]	C5orf47
[illegible]	HIST2H3D
	PPARGC1B
[illegible]	FAM72B
	PRRC1
	SCARNA2
	C5orf58
	SNORD45C
	ZNF474
716	MIR548D1
	OR2Y1
067	MIR553
	AFAP1L1
062	HIST2H2AA4
287	GRPEL2
	FAM72D
050	LSM11
331	LOC728855
939	GPR151
	LOC728875
168	ZNF300P1
216	NBPF24
230	NUDCD2
673	GPR89C
	UBLCP1
	NBPF16
	SOWAHA
	PDZK1P1
AM2	SHROOM1
	PPIAL4F
749	C5orf24

	LOC728989
	C5orf20
	LOC729970
	SLC36A2
	LOC729987
	SPINK13
	PPIAL4E
	C5orf41
	PFN1P2
	CEP120
	MIR942
	SLC25A48
	MIR760
	SRFBP1
	LOC100128787
658	LOC153469
678	LOC100129046
841	ZMAT2
894	LOC100129138
	BTNL9
387	LOC100129269
	CCDC112
	LOC100129620
	PPP1R2P3
	LOC100130000
	FAM71B
	LOC100131564
	PRELID2
	NBPF10
	SH3RF2
930	FCGR1C
	PLAC8L1
	ZRANB2-AS1
	RNF145
	LOC100286793
	ADAMTS19
	LOC100287722
	HIGD2A
	LOC100289178
	SPATA24
	LOC100289211
	DNAJC18
	MIR320B1
	FAM153B
	MIR1262
	LOC202181
	MIR3117

STK32A
MIR4256
AQPEP
MIR3671
SLC36A1
MIR548AA1
EIF4E1B
LOC100505768
RASGEF1C
LOC100506343
LOC257358
FPGT-TNNI3K
EFCAB9
TMEM56-RWDD3
SH3PXD2B
MIR4794
LOC285593
MIR4711
FAM153A
MIR2682
ARL10
MIR4423
DTWD2
NEGR1-IT1
RELL2
ZRANB2-AS2
LOC285626
LOC285627
LOC285629
SLC36A3
KIF4B
OR2V2
ZNF454
C5orf60
CHSY3
LOC340037
TMEM173
FAM170A
LOC340073
LOC340074
ARSI
PFN3
ZNF879
IRGM
FBLL1
CATSPER3
NIPAL4

TICAM2
DND1
C5orf25
FLJ38109
C5orf48
LOC389332
C5orf65
C5orf46
ARHGEF37
C5orf63
SPINK6
ANKHD1-EIF4EBP3
MIR103A1
MIR143
MIR145
MIR146A
MIR218-2
SPINK14
FNDC9
C5orf56
FLJ16171
MIR340
IGIP
TIFAB
LOC553103
CTXN3
SNORD95
SNORD96A
ECSCR
LOC643201
GRXCR2
SPINK9
ZFP62
LOC644100
FLJ33630
CCNI2
CBY3
FAM153C
SNORA74B
MIR585
SNHG4
MIR143HG
LOC728342
LOC728554
LOC729080
AACSP1
LOC729678

OR4F29
VTRNA2-1
MIR874
FAM196B
LOC100132062
LOC100132287
LOC100133331
C5orf52
LOC100268168
MIR1289-2
MIR1229
MIR103B1
MIR1244-1
TMED7-TICAM2
MIR1244-3
MIR1244-2
MIR3142
MIR3141
MIR4281
MIR3655
MIR3912
MIR3936
MIR3661
LOC100505658
LOC100505841
LOC100507387
MIR4633
MIR4634
MIR4461
MIR4460
MIR4638
MIR378E
LOC100859930

ype B
Del
CDKN2A
PTEN
PVRL1
LINC00290
AGXT
hsa-mir-760
RPL22
hsa-mir-582
ZMYND11
hsa-mir-378
hsa-mir-202
PARK2
LSAMP-AS3
SPRED1
RBFOX1
FMN1
B2M
hsa-mir-3187
PTPRD
hsa-mir-1267
FBXO25
hsa-mir-1910
hsa-mir-605
hsa-mir-887
GPHN
hsa-mir-203
WWOX
NAALADL2
hsa-mir-943
hsa-mir-3201
PHF2P1
GOLGA3
hsa-mir-3181
hsa-mir-3135
hsa-mir-3183
hsa-mir-148b
hsa-mir-587
hsa-mir-3185
C9orf53
KLLN
KIF1A
ABCA4
KCNAB2

PDE4D
TUBB8
hsa-mir-145
ADAM8
EIF3J
ATP5D
C9orf123
ATP4B
C8orf42
AFG3L1P
PRKG1
hsa-mir-4278
hsa-mir-4309
MIR4789
hsa-mir-571
hsa-let-7b
ANKRD20A9P
POLE
hsa-mir-1826
hsa-mir-563
ABR
hsa-mir-615
hsa-mir-2113
hsa-mir-196a-1
GPR35
BRDT
ACOT7
PART1
hsa-mir-584
UTF1
CTDSPL2
AZU1
COL4A1
ERICH1
APRT
CSTF2T
hsa-mir-4277
hsa-mir-1247
ADD1
hsa-mir-1249
LINC00442
PXMP2
ADCY7
hsa-mir-3134
DOC2B
hsa-mir-196a-2
AIM1

hsa-mir-10a
C2orf54
CNN3
ICMT
ADRB2
VENTX
SPG11
HCN2
COL4A2
ZNF596
C16orf3
MIR605
ADCY2
hsa-mir-656
ADRA2C
ACR
ZNF10
CBLN1
hsa-mir-4270
RPH3AL
hsa-mir-1293
AMD1
hsa-mir-1203
LOC200772
DR1
CHD5
ANXA6
KNDC1
PATL2
BSG
EFNB2
LOC286083
CA5A
CTNND2
hsa-mir-370
ATP5I
ARSA
ZNF26
CYLD
hsa-mir-885
TIMM22
hsa-mir-1291
PRDM1
hsa-mir-152
AQP12A
F3
HES2

ATOX1
MIR202
LOC645212
CDC34
F7
OR4F21
CBFA2T3
DAP
hsa-mir-345
CRMP1
CHKB
ZNF84
MMP2
ATP2B2
GEMIN4
ACCN2
CCNC
hsa-mir-4315-1
AQP12B
GBP2
NPHP4
CAMK2A
CIRBP
F10
RPL23AP53
CDH15
DNAH5
hsa-mir-342
CTBP1
CPT1B
ZNF140
PHKB
BTD
GLOD4
ACVR1B
COL10A1
hsa-mir-2117
GFI1
GPR153
CD14
CNN2
GAS6
COX4I1
MTRR
hsa-mir-3173
DGKQ
TYMP

ZNF268
RBL2
CAV3
RNMTL1
ACVRL1
EPHA7
hsa-mir-2909
GCLM
RNF207
CD74
CFD
ING1
CYBA
MYO10
SERPINA3
EVC
FBLN1
P2RX2
SALL1
DAZL
VPS53
ADCY6
FOXO3
hsa-mir-632
ABCD3
HES3
CDX1
ARID3A
LAMP1
DPEP1
NDUFS6
AKT1
FGFR3
PPARA
ANKLE2
SIAH1
FANCD2
NXN
ABCD2
FRK
hsa-mir-365-2
SNORD21
MIR4689
CSF1R
EFNA2
LIG4
FANCA

SDHA
BDKRB1
GAK
MAPK11
CHFR
N4BP1
FBLN2
FAM57A
AMHR2
FYN
hsa-mir-3184
RPL5
MIR4417
CSNK1A1
ELANE
GRK1
FOXF1
SLC6A3
BDKRB2
GRK4
MAPK12
FBRSL1
IRX5
GRM7
FAM101B
AQP2
GPR6
hsa-mir-144
TGFBR3
CTNNA1
GPX4
SOX1
FOXL1
SLC9A3
CALM1
HTT
SBF1
PGAM5
DNAJA2
HRH1
C17orf97
AQP5
GRIK2
ACACA
EVI5
DIAPH1
GZMM

TFDP1
FOXC2
SRD5A1
SERPINA6
HGFAC
UPK3A
LOC647589
ZNF267
IL5RA
BHLHA9
AQP6
HDAC2
ACCN1
CDC7
DPYSL3
PALM
CUL4A
GALNS
TERT
FOXN3
IDUA
WNT7B
ZNF605
ZNF423
IRAK2
DBIL5P
ARF3
KPNA5
ACLY
BCAR3
SLC26A2
POLR2E
IRS2
GAS8
TRIO
CHGA
LETM1
CELSR1
LOC100507055
RPGRIP1L
ITPR1
MIR3183
ATF1
LAMA4
AP2B1
ARHGAP29
HBEGF

POLRMT
PROZ
HSBP1
SEMA5A
CKB
LRPAP1
PPP6R2
ORC6
OGG1
LOC100506388
ATP5G2
MARCKS
ALDOC
GLMN
FAT2
PRTN3
ARHGEF7
IRF8
TRIP13
CRIP1
MSX1
ZBED4
TP53TG3
OXTR
CACNB3
POU3F2
AOC2
MTF2
FGF1
PTBP1
CDC16
MC1R
PDCD6
CRIP2
MYL5
SCO2
TOX3
PPARG
CCNT1
PREP
ARL4D
KIAA1107
GLRA1
STK11
TUBGCP3
MVD
MARCH6

DIO2
PDE6B
PKDREJ
BRD7
RAB5A
CNTN1
REV3L
ATP5G1
LRRC8B
GM2A
MADCAM1
TNFSF13B
CHMP1A
BASP1
DIO3
RGS12
NUP50
HEATR3
RAF1
COL2A1
ROS1
ATP6V0A1
RWDD3
GPX3
MED16
RASA3
RPL13
SLC12A7
DYNC1H1
RNF4
RABL2B
VPS35
RPL32
EIF4B
SIM1
BLMH
SNORA66
NR3C1
FSTL3
MYO16
SPG7
PAPD7
EIF5
SH3BP2
GRAMD4
NOD2
SATB1

CELA1
SMPD2
BRCA1
DNTTIP2
HARS
ABCA7
ATP11A
SLC7A5
TPPP
ELK2AP
WFS1
MLC1
PAPD5
SEC13
GPD1
MAP3K7
FMNL1
TMED5
NDST1
SBNO2
MCF2L
CDK10
EXOC3
EML1
WHSC1
KIAA0930
AKTIP
SETMAR
NCKAP1L
NR2E1
CACNB1
FNBP1L
HTR4
HMHA1
TMCO3
MBTPS1
CCT5
GALC
WHSC2
MAPK8IP2
FTO
SLC6A1
NR4A1
TSPYL1
CDC6
LRRC8D
IK

FGF22
ARGLU1
TAF1C
FBXL7
GTF2A1
ZNF141
PLXNB2
IRX6
SLC6A6
HNRNPA1
DDO
CDC27
RPAP2
NDUFA2
C19orf24
DCUN1D2
USP10
KIAA0947
BRF1
SLBP
BRD1
IRX3
SLC6A11
HOXC4
SNX3
CCR7
ZNF644
PCDH1
RNF126
ANKRD10
C16orf7
PP7080
HSP90AA1
NOP14
ARHGAP8
SHCBP1
SYN2
HOXC5
CD164
CNP
LRRC8C
PCDHGC3
WDR18
RAB20
KIAA0513
IRX4
IFI27

FAM193A
TBC1D22A
CHD9
TIMP4
HOXC6
WISP3
CPD
GBP4
PDE6A
LPPR3
CARKD
PIEZO1
TAS2R1
ITPK1
C4orf6
ATXN10
ITFG1
NR2C2
HOXC8
WASF1
CRHR1
GBP5
PDGFRB
KISS1R
PCID2
ATP2C2
FAM134B
JAG2
MFSD10
FAM19A5
NETO2
UBE2E2
HOXC9
FHL5
CRYBA1
SLC44A3
PFDN1
MIDN
UPF3A
COX4NB
FAM105A
KLC1
MAEA
RIBC2
LONP2
VHL
HOXC10

ATG5
CSF3
TMEM56
POU4F3
R3HDM4
CARS2
TUBB3
NSUN2
MARK3
PCGF3
SMC1B
CAPNS2
WNT7A
HOXC11
ZBTB24
DHX8
GBP6
PPP2R2B
C19orf6
GRTP1
PRDM7
CEP72
ATXN3
SPON2
NCAPH2
GPT2
XPC
HOXC12
FIG4
DUSP3
HFM1
PURA
TPGS1
ABHD13
TCF25
ANKH
NDUFB1
TACC3
GTSE1
ABCC11
BRPF1
HOXC13
CASP8AP2
ERBB2
ALG14
RPS14
GRIN3B

ADPRHL1
ZCCHC14
AHRR
SERPINA5
MXD4
MOV10L1
NKD1
COLQ
IGFBP6
FUT9
ETV4
EPHX4
SLC6A7
C19orf21
TEX29
KIAA0182
MRPL36
SERPINA1
CPLX1
FAM118A
MYLK3
CAMK1
ITGA5
TRAF3IP2
EVI2A
ZNF326
SPARC
CIRBP-AS1
SPACA7
COTL1
BRD9
SERPINA4
SLC26A1
TTC38
ABCC12
BHLHE40
ITGB7
ASCC3
EVI2B
BTBD8
SPINK1
C19orf26
DAOA
MLYCD
FASTKD3
PPP2R5C
D4S234E

C22orf26
C16orf78
KAT2B
KRT1
BVES
EZH1
CCDC18
TAF7
ODF3L2
DAOA-AS1
CPNE7
IRX1
LGMN
STX18
MIOX
ANKRD26P1
SH3BP5
KRT2
SEC63
FLOT2
BARHL2
TCOF1
PRSS57
LINC00346
IL17C
ZDHHC11
PSMC1
FGFRL1
PRR5
SNX20
VGLL4
KRT3
ANKRD6
GAST
HSP90B3P
UBE2D2
MIR3187
CHAMP1
ANKRD11
LPCAT1
MOK
CYTL1
TRMU
LOC146481
EDEM1
KRT4
CDK19

FZD2
GBP7
EIF4EBP3
MIR4745
FAM70B
OSGIN1
CLPTM1L
SEL1L
PIGG
PANX2
CNEP1R1
TBC1D5
KRT5
MDN1
G6PC
C1orf146
PCDHGB4
C13orf35
GINS2
ROPN1L
TNFAIP2
STK32B
CERK
LOC283914
TATDN2
KRT6A
TSPYL4
KAT2A
FAM69A
HDAC3
FLJ44054
TRAPPC2L
MED10
TRAF3
LYAR
ALG12
SLC6A10P
SRGAP3
KRT6B
UFL1
GFAP
GBP1P1
NRG2
FLJ41484
NECAB2
NKD2
TSHR

KIAA1530
CRELD2
C16orf87
IQSEC1
KRT7
PNISR
CCR10
FLJ27354
RNF14
LINC00460
KLHDC4
FAM105B
VRK1
ZFYVE28
ADM2
LOC388276
ARPC4
KRT8
FBXL4
GRB7
FLJ31662
PCDHGA8
FAM155A
DEF8
ZNF622
WARS
TNIP2
TRABD
LOC390705
TADA3
KRT18
SESN1
GRN
GEMIN8P4
PCDHA9
LINC00552
BANP
C5orf55
XRCC3
HAUS3
SELO
FLJ26245
ATG7
KRT81
OSTM1
HCRT
LOC729970

MATR3
MCF2L-AS1
ZDHHC7
CCDC127
YY1
ABCA11P
HDAC10
HERC2P4
CHL1
KRT82
NDUFAF4
HOXB1
MIR760
KIAA0141
LOC100506394
JPH3
UBE2QL1
GPR68
MFSD7
LDOC1L
UBE2MP1
CAND2
KRT83
DSE
HOXB2
LOC100129046
JAKMIP2
KIAA1609
C5orf49
TCL1A
TMEM175
SHANK3
LOC643714
RFTN1
KRT84
TUBE1
HOXB3
LOC100131564
SLC23A1
WFDC1
FAM173B
GPR65
TMEM128
TUBGCP6
LOC643802
NUP210
KRT85

C6orf203
HOXB4
TMEM56-RWDD3
GNPDA1
MTHFSD
CMBL
ADAM6
C4orf42
LOC90834
CRNDE
PLCL2
KRT86
CDC40
HOXB5
SRA1
DBNDD1
PLEKHG4B
DLK1
TMEM129
LMF2
LINC00273
ANKRD28
LALBA
RWDD1
HOXB6
G3BP1
KLHL36
C5orf38
CCNK
EVC2
PHF21B
TP53TG3C
CAPN7
NELL2
COQ3
HOXB7
APBB3
FBXO31
IRX2
MTA1
OTOP1
KLHDC7B
LOC729264
MKRN2
NFE2
SOBP
HOXB8

TNIP1
CDT1
ADAMTS16
RPS6KA5
ZNF595
LOC150381
TP53TG3B
THUMPD3
SLC11A2
QRSL1
HOXB9
TCERG1
MAP1LC3B
LOC255167
TRIP11
JAKMIP1
C22orf40
LOC100130700
HACL1
PCBP2
PDSS2
HSD17B1
SPINK5
HSDL1
LOC285577
NRXN3
FAM53A
CN5H6.4
LOC100505619
TTLL3
PDE1B
GOPC
IFI35
SYNPO
CRISPLD2
LOC285692
BAG5
ZBTB49
LOC284933
LOC100507577
CNTN6
PFDN5
LYRM2
IGFBP4
ABLIM3
SPIRE2
LOC285696

C14orf2
ZNF721
RPL23AP82
MIR548AE2
LSM3
PFKM
HACE1
ITGA2B
HMGXB3
ZNF469
SLC6A19
CDC42BPB
ZNF718
LOC339685
LINC00312
POU6F1
BEND3
ITGB3
ARHGAP26
CENPBD1
LOC340094
TCL1B
CRIPAK
C22orf34
LMCD1
PPP1R1A
PRDM13
JUP
HARS2
ZNF276
SLC6A18
KIAA0125
LOC285484
CHKB-CPT1B
C3orf32
PRKAG1
BACH2
KCNJ12
PCDHGA12
KCNG4
LRRC14B
TECPR2
DOK7
LINC00207
TRNT1
PRPH
POPDC3

KPNB1
LRRTM2
RNF166
FLJ33360
GOLGA5
FLJ35424
MIRLET7BHG
CRBN
TWF1
MICAL1
KRT9
CCDC69
DNAAF1
TAG
FBLN5
RNF212
FLJ46257
C3orf19
RARG
MANEA
KRT10
PCDHB5
SPATA2L
MARCH11
SIVA1
C4orf10
IL17REL
GHRL
RNY5
GPR63
KRT12
SNORA74A
C16orf55
LOC442132
CYP46A1
NAT8L
MIRLET7A3
ARL8B
SCN8A
ARMC2
KRT13
IL17B
ZC3H18
ANKRD33B
PAPOLA
C4orf44
MIRLET7B

SETD5
SMARCD1
RPF2
KRT14
PCDHB1
SLC38A8
SDHAP3
PTPN21
FAM86EP
LINC00229
TMEM40
SP1
MCHR2
KRT15
DCTN4
SLC22A31
LOC728613
RCOR1
POLN
FAM116B
TMEM111
TARBP2
FAXC
KRT16
MZB1
FLJ30679
LOC729506
PACS2
C4orf48
PIM3
BRK1
TMBIM6
GJA10
KRT17
PAIP2
LOC146513
SNORD123
PPP1R13B
LOC402160
ODF3B
RAD18
TFCP2
RTN4IP1
KRT19
PCDH12
ZFPM1
LOC100130744

DICER1
ZNF876P
PRR5-ARHGAP8
LRRN1
VDR
USP45
KRT31
LARS
ADAD2
MIR4277
FLRT2
ZNF732
SYCE3
CIDEC
WNT1
SLC22A16
KRT32
CXXC5
MGC23284
MIR4278
KIF26A
SCARNA22
LOC730668
ZFYVE20
WNT10B
KIAA1919
KRT33A
RBM27
LINC00311
LOC100505738
C14orf109
MIR943
LOC100128946
MTMR14
MAP3K12
GTF3C6
KRT33B
PCDHB18
ZNF778
LOC100505806
TCL6
LOC100129917
LOC100144603
MRPS25
TUBA1A
KLHL32
KRT34

PCDHB17
ACSF3
LOC100506688
PRO1768
LOC100130872
LOC100271722
CRELD1
MLL2
NUS1
KRT35
WDR55
LINC00304
MIR4458
GPR132
LOC100133461
MIR1249
TMEM43
AAAS
SLC16A10
LASP1
ANKHD1
SNAI3
MIR4454
SERPINA10
TMED11P
MIR3201
ZNF385D
SOAT2
BVES-AS1
LGALS9
TMCO6
FAM92B
MIR4457
GLRX5
LOC100507266
MIR3619
HDAC11
NPFF
CCDC162P
LHX1
RBM22
CTU2
MIR4637
EVL
MIR378D1
LOC100506714
TSEN2

ENDOU
AKD1
LIG3
PCDHGC5
PABPN1L
MIR4636
C14orf129
MIR4800
MIR4763
GRIP2
KRT75
NT5DC1
NBR1
PCDHGC4
LOC400548
MIR4456
CINP
HTT-AS1
MIR4762
C3orf20
SCAF11
FAM26D
MAPT
PCDHGB7
LOC400550
MIR4635
ASB2
MIR4535
JAGN1
DDX23
ZUFSP
ADAM11
PCDHGB6
LOC400558
CPSF2
GHRLOS2
SLC4A8
FAM162B
MEOX1
PCDHGB5
C16orf74
KCNK10
IL17RC
ESPL1
HS3ST5
MLLT6
PCDHGB3

SNORD68
CDCA4
EAF1
DAZAP2
GPRC6A
MPP2
PCDHGB2
LOC727710
C14orf102
OXNAD1
TROAP
RFX6
MPP3
PCDHGB1
LOC732275
ATG2B
C3orf24
YAF2
VGLL2
MYL4
PCDHGA11
LOC100128881
UBR7
GALNTL2
TUBA1B
LACE1
MYO1D
PCDHGA10
LOC100130015
MEG3
KCNH8
RAPGEF3
MMS22L
NAGLU
PCDHGA9
LOC100287036
SMEK1
CHCHD4
MCRS1
FAM26E
NEUROD2
PCDHGA7
MIR1910
BTBD7
METTL6
ATF7
SCML4

NF1
PCDHGA6
C16orf95
TDP1
TAMM41
GALNT6
CEP57L1
NFE2L1
PCDHGA5
MIR4722
ZNF839
IL17RE
COPZ1
PPIL6
NMT1
PCDHGA4
SPATA7
SGOL1
FAIM2
LOC285758
NOS2
PCDHGA3
KCNK13
PP2D1
DDN
FLJ34503
NSF
PCDHGA2
C14orf132
EFHB
TENC1
DCBLD1
OMG
PCDHGA1
DDX24
CPNE9
KCNH3
RSPH4A
PEX12
PCDHGB8P
UNC79
FGD5
CBX5
LINC00222
PNMT
PCDHB15
BEGAIN

CIDECP
ANP32D
LIN28B
MED1
PCDHB14
PPP4R4
CNTN4
SMUG1
FAM26F
PPY
PCDHB13
MOAP1
SUMF1
PRPF40B
GSTM2P1
PSMB3
PCDHB12
DIO3OS
RPUSD3
METTL7A
RFPL4B
PYY
PCDHB11
INF2
PRRT3
LETMD1
C6orf225
PSMD3
PCDHB10
BCL11B
LOC285370
ZNF385A
TSG1
PSMD11
PCDHB9
OTUB2
LOC285375
RND1
TRAF3IP2-AS1
RAB5C
PCDHB8
ZFYVE21
DPH3
RACGAP1
LOC728012
RAD51D
PCDHB7

MEG8
LOC339862
SENP1
TPI1P3
RARA
PCDHB6
WDR25
COL6A4P1
PDZRN4
C6orf186
RPL19
PCDHB4
LINC00341
TPRXL
DHH
BET3L
RPL23A
PCDHB3
CLMN
LHFPL4
PLEKHA8P1
LOC100130890
RPL27
PCDHB2
CATSPERB
VENTXP7
IRAK4
MIR2113
CCL1
PCDHAC2
ZC3H14
LOC401052
FKBP11
MIR548H3
CCL2
PCDHAC1
RIN3
LOC440944
KRT76
LOC100422737
CCL3
PCDHA13
C14orf159
SNORA7A
CSAD
MIR4464
CCL3L1

PCDHA12
TMEM121
MIR563
BIN2
MIR4643
CCL4
PCDHA11
AMN
MIR885
LIMA1
CCL5
PCDHA10
IFI27L2
EGOT
PPHLN1
CCL7
PCDHA8
SETD3
GHRLOS
HDAC7
CCL8
PCDHA7
APOPT1
LOC100129480
GPR84
CCL11
PCDHA6
HHIPL1
LOC100132526
SLC38A2
CCL13
PCDHA5
C14orf142
LOC100288428
PRR13
CCL14
PCDHA4
STON2
MIR4270
KANSL2
CCL15
PCDHA3
FAM181A
MIR3714
SLC38A4
CCL16
PCDHA2

BTBD6
FGD5-AS1
KIF21A
CCL18
PCDHA1
EFCAB11
LOC100505696
SLC48A1
CCL23
VTRNA1-3
EXOC3L4
LOC100507582
LMBR1L
SDF2
VTRNA1-2
WDR20
ARPC4-TTLL3
SMAGP
SH3GL1P1
VTRNA1-1
AHNAK2
MIR4791
DIP2B
SH3GL1P2
NMUR2
TRMT61A
MIR4790
CALCOCO1
SLC4A1
KCTD16
TDRD9
MIR548AC
NCKAP5L
SLC6A4
PCDHB16
ANKRD9
C12orf10
SMARCE1
HMHB1
AK7
C12orf44
SP2
SIL1
IFI27L1
SPATS2
STAT3
ARAP3

C14orf79
TMEM106C
STAT5A
PCYOX1L
PLD4
RPAP3
STAT5B
SH3TC2
ADSSL1
DNAJC22
SUPT6H
NDFIP1
TTC8
ADAMTS20
TADA2A
FBXO38
TC2N
SLC38A1
HNF1B
YIPF5
SLC24A4
CSRNP2
MLX
TIGD6
SLC25A29
PUS7L
THRA
SPRY4
DEGS2
RACGAP1P
TNFAIP1
SLC4A9
LINC00239
FAM186B
TOP2A
SLC25A2
LOC145216
TMEM117
DNAJC7
PCDHB19P
GSC
TUBA1C
TUBG1
PSD2
SERPINA12
ALG10
UBTF

C5orf32
PRIMA1
SPRYD3
VTN
SPINK7
CEP128
MFSD5
WNT3
C5orf62
TTC7B
C12orf62
WNT9B
FCHSD1
C14orf49
ZCRB1
PCGF2
ZNF300
EML5
CCDC65
ZNF207
MYOZ3
MGC23270
LACRT
TAF15
SLC35A4
NUDT14
FMNL3
RND2
SCGB3A2
LINC00521
CERS5
PIP4K2B
PPARGC1B
SERPINA11
FAM113B
FOXN1
AFAP1L1
LOC283585
KRT71
CNTNAP1
GRPEL2
LOC283587
LARP4
TCAP
GPR151
FAM181A-AS1
SLC2A13

SKAP1
ZNF300P1
SNHG10
DCD
AOC3
SLC36A2
C14orf177
MUCL1
BECN1
SPINK13
SLC25A47
LRRK2
KRT38
LOC153469
LINC00523
FAM186A
KRT37
ZMAT2
KIAA0284
RHEBL1
KRT36
PRELID2
C14orf80
C12orf54
KSR1
SH3RF2
CCDC85C
ZNF641
CDK5R1
PLAC8L1
ITPK1-AS1
OR10AD1
HAP1
SPATA24
SERPINA9
SP7
MAP3K14
DNAJC18
LINC00226
GTSF1
SLC13A2
STK32A
LINC00221
KRT74
UNC119
SLC36A1
COX8C

ASB8
TIAF1
RELL2
ASPG
KRT72
ZNHIT3
SLC36A3
SERPINA13
PRICKLE1
EFTUD2
TMEM173
C14orf64
BCDIN3D
RPL23
ARSI
RTL1
ALG10B
NPEPPS
IRGM
TMEM179
CPNE8
GOSR1
DND1
LOC400236
KRT80
CCL4L1
C5orf65
LOC400238
GRASP
GOSR2
C5orf46
DICER1-AS1
KRT78
NR1D1
ARHGEF37
C14orf180
ANO6
TRAF4
SPINK6
MIR127
ARID2
KIAA0100
ANKHD1-EIF4EBP3
MIR134
LOC255411
TBKBP1
MIR143

MIR136
LOC283332
PLEKHM1
MIR145
MIR154
LOC283335
MED24
SPINK14
MIR203
ZNF740
LRRC37A
IGIP
MIR299
LOC283403
HDAC5
ECSCR
CCDC88C
LOC283404
GJC1
GRXCR2
MIR323A
C12orf40
PSME3
SPINK9
MIR337
GXYLT1
EIF1
SNHG4
MIR345
TMPRSS12
NBR2
MIR143HG
MIR376C
KRT6C
CALCOCO2
LOC729080
MIR369
KRT73
RAMP2
MIR3655
MIR376A1
C1QL4
HOXB13
LOC100505658
MIR377
KRT79
VAT1

MIR379
SYT10
LEPREL4
MIR380
ANKRD33
HEXIM1
MIR381
H1FNT
SPAG5
MIR382
OR8S1
CCT6B
MIR433
AMIGO2
C1QL1
MIR431
KRT77
RUNDC3A
MIR329-1
C12orf68
STARD3
MIR329-2
LOC400027
CBX1
MIR323B
LOC400043
DDX52
MIR409
FIGNL2
DUSP14
MIR412
MIR196A2
SYNRG
MIR410
DBX2
CASC3
MIR376B
FLJ12825
IKZF3
MIR485
MIR148B
SARM1
MIR493
HIGD1C
GPATCH8
MIR432
GLYCAM1

KCNH4
MIR494
HNRNPA1P10
SUZ12
MIR495
SNORA2A
FAM215A
MIR496
SNORA2B
PPY2
MIR487A
SNORA34
PYY2
TEX22
MIR615
KRT23
MIR539
HOTAIR
TMEM98
MIR376A2
LOC100233209
POLDIP2
MIR487B
LOC100240734
TBC1D29
SCARNA13
LOC100240735
WSB1
SNORA28
LOC100286844
ERAL1
SNORA79
MIR1293
AATF
MIR411
MIR1291
SNORD4B
MIR654
LOC100335030
SNORD4A
MIR655
MIR4701
SNORD42B
MIR656
MIR3198-2
SNORD42A
SNORD113-1

MIR4698
TUBG2
SNORD113-2
LOC100652999
TMEM97
SNORD113-4
NKIRAS2
SNORD113-5
CCDC56
SNORD113-6
GIT1
SNORD113-7
PSMC3IP
SNORD113-9
SNX11
SNORD114-1
TBX21
SNORD114-2
SOCS7
SNORD114-3
SOST
SNORD114-4
RAPGEFL1
SNORD114-5
COPZ2
SNORD114-6
PIPOX
SNORD114-7
ARL17A
SNORD114-8
CRLF3
SNORD114-9
SLC25A39
SNORD114-10
NLK
SNORD114-11
HIGD1B
SNORD114-12
CDK12
SNORD114-13
KRT20
SNORD114-14
NLE1
SNORD114-15
FNDC8
SNORD114-16
CWC25

SNORD114-17
LRRC37A4
SNORD114-18
SLFN12
SNORD114-19
PNPO
SNORD114-20
KLHL11
SNORD114-21
RHOT1
SNORD114-22
C17orf79
SNORD114-23
C17orf63
SNORD114-24
ADAP2
SNORD114-25
UTP6
SNORD114-26
GSDMB
SNORD114-27
ATXN7L3
SNORD114-28
PLXDC1
SNORD114-29
LYZL6
SNORD114-30
NUFIP2
SNORD114-31
TAOK1
MIR758
ARHGAP23
MIR668
PHF12
MIR770
FKBP10
SNORA11B
C17orf75
MIR300
UBE2Z
MIR541
WNK4
MIR665
C17orf53
MIR543
TMUB2
MIR889

DHX58
ZBTB42
MMP28
LOC100129345
DHRS11
LOC100131366
PRR15L
MIR1247
TEFM
MIR1185-1
ACBD4
MIR1185-2
DCAKD
MIR1197
GGNBP2
MIR1193
ATAD5
MIR4309
MRM1
MIR3173
PLEKHH3
LOC100507043
DBF4B
MIR3545
MYO19
MIR4710
CDK5RAP3
MIR2392
SP6
LOC100628307
COASY
SRCIN1
AARSD1
KRTAP1-3
KRTAP1-1
KRTAP9-9
KRTAP4-6
KRTAP2-1
TTC25
KRTAP4-12
RAB34
KRTAP1-5
KRTAP3-1
KRTAP3-2
KRTAP9-2
KRTAP9-3
KRTAP9-8

KRTAP17-1
NSRP1
PPP1R1B
TBC1D3F
RNF135
MIEN1
MRPL45
VPS25
TMEM101
PRAC
RAB11FIP4
GHDC
KRTAP4-4
CORO6
TNS4
FBXL20
KRTAP9-4
KRTAP4-1
KRTAP4-5
KRTAP4-3
KRTAP4-2
KRTAP3-3
KRTAP2-4
SSH2
TP53I13
IFT20
LRRC46
SCRN2
AOC4
ZNF830
SLFN11
RASL10B
G6PC3
ASB16
PGAP3
PIGS
HSPB9
ORMDL3
PLCD3
SLC46A1
TMEM106A
LRRC37B
OSBPL7
NT5C3L
ABHD15
TLCD1
RFFL

ZPBP2
C17orf46
HEXIM2
LSM12
CCDC43
CNTD1
TMEM132E
SPACA3
SGK494
SEZ6
ANKRD13B
C17orf57
MRPL10
GJD3
KRT222
KRT40
C17orf50
SLFN13
SLC35G3
UNC45B
CD300LG
KIF18B
RUNDC1
TMEM199
PROCA1
DHRS13
C17orf69
LOC147093
LRRC37BP1
WIPF2
KRT25
TMEM99
SLFN5
NAGS
FAM134C
RHBDL3
IMP5
KRT28
KRT24
ARHGAP27
ZNF385C
C17orf108
RDM1
GAS2L2
STH
C17orf66
KIAA1267

C17orf105
FAM171A2
C17orf104
TTLL6
KRT18P55
NEK8
PIGW
C17orf78
LOC284100
CISD3
GSDMA
KRT42P
PTRF
FAM27L
FLJ36000
KLHL10
C17orf65
C17orf51
MSL1
KRT27
SLFN14
FLJ43826
STAC2
CCL14-CCL15
KRT26
HOXB13-AS1
EFCAB5
TMIGD1
CCL4L2
C17orf98
LOC388387
CCDC103
RPRML
ARL5C
KRT39
MYO18A
C17orf102
MGC57346
LOC404266
MIR10A
MIR144
MIR152
MIR193A
MIR196A1
TBC1D3B
TBC1D3C
CCL3L3

TBC1D3P5
SUZ12P
LOC440434
GPR179
LRRC37A2
FBXO47
MIR423
ARGFXP2
DPRXP4
MIR451A
SNORA21
LOC644172
LOC644246
SEBOX
KRTAP4-11
MRPL45P2
TBC1D3G
SNORD7
MIR632
KRTAP4-8
KRTAP2-2
KRTAP9-1
NSFP1
TBC1D3
TBC1D3H
LOC730755
SNORD124
MIR365B
LOC100128977
LOC100130148
LOC100130581
LOC100131347
KRTAP4-9
KRTAP4-7
LOC100133991
KCNJ18
C17orf96
LOC100190938
MIR1203
MIR2117
MIR4315-2
MIR2909
MIR3185
MIR3184
MIR4315-1
MTRNR2L1
LOC100505576

LRRC3C
KRTAP16-1
LOC100505782
ARL17B
SPAG5-AS1
AA06
SLFN12L
RAD51L3-RFFL
MIR4523
MIR4728
MIR4726
MIR4734
MIR4724
MIR4733
MIR451B
MIR4522
MIR4732
MIR4723
MIR4727
MIR4725
RAB34

Supplementary Table 5. Immune subtypes in 7 cancer types

TCGA_type	TCGA_Barcode	Subtype
COAD	TCGA-CM-5864-01A	A
COAD	TCGA-AD-6890-01A	A
COAD	TCGA-AA-3516-01A	B
COAD	TCGA-AA-A00K-01A	A
COAD	TCGA-A6-2679-01A	B
COAD	TCGA-CM-4747-01A	B
COAD	TCGA-AA-3833-01A	B
COAD	TCGA-CM-6675-01A	A
COAD	TCGA-AA-A00E-01A	B
COAD	TCGA-AA-3544-01A	B
COAD	TCGA-AA-3655-01A	A
COAD	TCGA-DM-A280-01A	A
COAD	TCGA-AA-3666-01A	A
COAD	TCGA-AA-3818-01A	A
COAD	TCGA-F4-6569-01A	B
COAD	TCGA-AA-3522-01A	B
COAD	TCGA-AA-3548-01A	B
COAD	TCGA-CM-5344-01A	A
COAD	TCGA-D5-6538-01A	A
COAD	TCGA-AA-3939-01A	B
COAD	TCGA-D5-6539-01A	B
COAD	TCGA-CA-5797-01A	A
COAD	TCGA-AA-3495-01A	B
COAD	TCGA-G4-6586-01A	A
COAD	TCGA-AY-A69D-01A	A
COAD	TCGA-D5-7000-01A	B
COAD	TCGA-G4-6294-01A	A
COAD	TCGA-AA-3531-01A	A
COAD	TCGA-AD-6899-01A	B
COAD	TCGA-A6-5662-01A	A
COAD	TCGA-F4-6460-01A	A
COAD	TCGA-G4-6320-01A	A
COAD	TCGA-AA-3552-01A	B
COAD	TCGA-CK-4952-01A	A
COAD	TCGA-AZ-6605-01A	B
COAD	TCGA-DM-A0X9-01A	A
COAD	TCGA-A6-2684-01C	A
COAD	TCGA-AA-3842-01A	A
COAD	TCGA-D5-6929-01A	A
COAD	TCGA-G4-6293-01A	B
COAD	TCGA-AA-A010-01A	B
COAD	TCGA-G4-6303-01A	A
COAD	TCGA-A6-5659-01B	A
COAD	TCGA-D5-6924-01A	A
COAD	TCGA-AA-A00Z-01A	A

COAD	TCGA-AA-3526-01A	B
COAD	TCGA-AD-6901-01A	A
COAD	TCGA-AA-A00F-01A	B
COAD	TCGA-CM-6162-01A	B
COAD	TCGA-AA-3989-01A	B
COAD	TCGA-AZ-6606-01A	A
COAD	TCGA-D5-6928-01A	B
COAD	TCGA-G4-6317-01A	A
COAD	TCGA-A6-2675-01A	B
COAD	TCGA-D5-6531-01A	B
COAD	TCGA-AD-6888-01A	A
COAD	TCGA-A6-2672-01B	A
COAD	TCGA-G4-6297-01A	B
COAD	TCGA-AA-3664-01A	A
COAD	TCGA-CM-6674-01A	A
COAD	TCGA-A6-6137-01A	B
COAD	TCGA-AA-3527-01A	A
COAD	TCGA-AA-3519-01A	B
COAD	TCGA-F4-6856-01A	B
COAD	TCGA-AA-3949-01A	B
COAD	TCGA-G4-6323-01A	B
COAD	TCGA-AY-6196-01A	B
COAD	TCGA-AA-3852-01A	B
COAD	TCGA-AA-3534-01A	A
COAD	TCGA-AA-A01X-01A	A
COAD	TCGA-AZ-5403-01A	A
COAD	TCGA-A6-A565-01A	B
COAD	TCGA-AA-3955-01A	A
COAD	TCGA-DM-A28E-01A	A
COAD	TCGA-AA-3530-01A	A
COAD	TCGA-CM-5868-01A	A
COAD	TCGA-AA-3841-01A	B
COAD	TCGA-AA-3856-01A	B
COAD	TCGA-DM-A1HA-01A	A
COAD	TCGA-DM-A0XD-01A	A
COAD	TCGA-5M-AAT5-01A	A
COAD	TCGA-AZ-6607-01A	B
COAD	TCGA-AD-6963-01A	A
COAD	TCGA-G4-6304-01A	A
COAD	TCGA-AZ-6603-01A	A
COAD	TCGA-AA-A00O-01A	A
COAD	TCGA-4N-A93T-01A	A
COAD	TCGA-5M-AAT4-01A	A
COAD	TCGA-AA-3496-01A	B
COAD	TCGA-NH-A50V-01A	B
COAD	TCGA-A6-6782-01A	A
COAD	TCGA-CK-5912-01A	A

COAD	TCGA-AA-3696-01A	A
COAD	TCGA-G4-6306-01A	A
COAD	TCGA-AA-3844-01A	A
COAD	TCGA-AA-A03J-01A	A
COAD	TCGA-CA-6719-01A	A
COAD	TCGA-AA-3966-01A	A
COAD	TCGA-AA-3517-01A	B
COAD	TCGA-AA-3553-01A	B
COAD	TCGA-QG-A5YV-01A	A
COAD	TCGA-AA-3662-01A	B
COAD	TCGA-AA-3511-01A	A
COAD	TCGA-G4-6317-02A	A
COAD	TCGA-AZ-4682-01B	A
COAD	TCGA-DM-A1HB-01A	A
COAD	TCGA-A6-2680-01A	B
COAD	TCGA-AZ-6600-01A	B
COAD	TCGA-AA-3697-01A	B
COAD	TCGA-A6-6781-01B	B
COAD	TCGA-AA-A02R-01A	A
COAD	TCGA-F4-6805-01A	B
COAD	TCGA-AA-3976-01A	A
COAD	TCGA-D5-6532-01A	A
COAD	TCGA-AA-A00L-01A	A
COAD	TCGA-CM-4743-01A	A
COAD	TCGA-4T-AA8H-01A	A
COAD	TCGA-AA-3542-01A	A
COAD	TCGA-AA-3941-01A	A
COAD	TCGA-AA-A024-01A	A
COAD	TCGA-AZ-4615-01A	A
COAD	TCGA-AA-3970-01A	B
COAD	TCGA-CK-4947-01B	B
COAD	TCGA-D5-6533-01A	A
COAD	TCGA-D5-6535-01A	B
COAD	TCGA-NH-A8F7-01A	A
COAD	TCGA-G4-6315-01A	A
COAD	TCGA-AA-3660-01A	B
COAD	TCGA-CA-5254-01A	B
COAD	TCGA-D5-5538-01A	B
COAD	TCGA-G4-6298-01A	A
COAD	TCGA-F4-6570-01A	B
COAD	TCGA-3L-AA1B-01A	B
COAD	TCGA-AA-3549-01A	A
COAD	TCGA-DM-A28G-01A	A
COAD	TCGA-A6-3808-01A	B
COAD	TCGA-AA-3875-01A	B
COAD	TCGA-CM-5341-01A	A
COAD	TCGA-DM-A1D8-01A	A

COAD	TCGA-AA-3555-01A	A
COAD	TCGA-AA-3982-01A	B
COAD	TCGA-A6-3810-01B	A
COAD	TCGA-A6-6138-01A	B
COAD	TCGA-AY-6386-01A	B
COAD	TCGA-D5-6923-01A	A
COAD	TCGA-A6-2671-01A	A
COAD	TCGA-D5-6920-01A	B
COAD	TCGA-CK-6747-01A	A
COAD	TCGA-AA-3994-01A	A
COAD	TCGA-AA-3510-01A	B
COAD	TCGA-AZ-4684-01A	B
COAD	TCGA-T9-A92H-01A	A
COAD	TCGA-AA-3538-01A	B
COAD	TCGA-A6-5664-01A	B
COAD	TCGA-AA-3713-01A	B
COAD	TCGA-F4-6463-01A	A
COAD	TCGA-AA-3867-01A	B
COAD	TCGA-AA-3715-01A	B
COAD	TCGA-AZ-4308-01A	A
COAD	TCGA-AA-3984-01A	B
COAD	TCGA-DM-A28M-01A	A
COAD	TCGA-AA-A02K-01A	A
COAD	TCGA-AA-A01Z-01A	A
COAD	TCGA-CM-4752-01A	B
COAD	TCGA-F4-6809-01A	A
COAD	TCGA-CA-5796-01A	B
COAD	TCGA-AU-3779-01A	A
COAD	TCGA-AA-3968-01A	B
COAD	TCGA-DM-A28K-01A	A
COAD	TCGA-AA-3685-01A	B
COAD	TCGA-DM-A1DB-01A	A
COAD	TCGA-AA-3675-01A	A
COAD	TCGA-AD-6964-01A	B
COAD	TCGA-AZ-6599-01A	A
COAD	TCGA-CA-6718-01A	B
COAD	TCGA-AA-3672-01A	B
COAD	TCGA-AA-3562-01A	B
COAD	TCGA-AZ-4313-01A	A
COAD	TCGA-AA-3811-01A	B
COAD	TCGA-AA-3861-01A	B
COAD	TCGA-AA-A02W-01A	A
COAD	TCGA-A6-3807-01A	A
COAD	TCGA-CM-6172-01A	B
COAD	TCGA-A6-5665-01A	B
COAD	TCGA-AA-3979-01A	A
COAD	TCGA-AA-A01K-01A	B

COAD	TCGA-AA-3858-01A	B
COAD	TCGA-D5-6931-01A	B
COAD	TCGA-AD-A5EK-01A	A
COAD	TCGA-DM-A1D6-01A	A
COAD	TCGA-AA-3869-01A	B
COAD	TCGA-AA-3525-01A	B
COAD	TCGA-A6-2678-01A	B
COAD	TCGA-A6-5661-01B	B
COAD	TCGA-AA-3529-01A	A
COAD	TCGA-AA-A02F-01A	A
COAD	TCGA-CK-6746-01A	A
COAD	TCGA-A6-6654-01A	B
COAD	TCGA-AA-3972-01A	A
COAD	TCGA-G4-6311-01A	B
COAD	TCGA-AA-3667-01A	A
COAD	TCGA-F4-6854-01A	A
COAD	TCGA-A6-6650-01B	A
COAD	TCGA-AA-A02E-01A	A
COAD	TCGA-CA-6715-01A	A
COAD	TCGA-G4-6626-01A	A
COAD	TCGA-CM-6164-01A	A
COAD	TCGA-AA-3663-01A	A
COAD	TCGA-AA-3855-01A	B
COAD	TCGA-QL-A97D-01A	B
COAD	TCGA-AA-3710-01A	B
COAD	TCGA-A6-5657-01A	B
COAD	TCGA-A6-2683-01A	A
COAD	TCGA-G4-6307-01A	A
COAD	TCGA-AA-A01D-01A	B
COAD	TCGA-F4-6807-01A	B
COAD	TCGA-AA-3973-01A	A
COAD	TCGA-AA-3862-01A	B
COAD	TCGA-A6-3809-01A	B
COAD	TCGA-A6-3809-01A	B
COAD	TCGA-AZ-4323-01A	B
COAD	TCGA-CK-4950-01A	B
COAD	TCGA-A6-2674-01B	B
COAD	TCGA-AZ-4614-01A	A
COAD	TCGA-AA-A00Q-01A	A
COAD	TCGA-NH-A6GB-01A	A
COAD	TCGA-CM-6167-01A	B
COAD	TCGA-G4-6322-01A	B
COAD	TCGA-AA-3514-01A	A
COAD	TCGA-CM-6679-01A	A
COAD	TCGA-AA-A02J-01A	A
COAD	TCGA-AA-3956-01A	B
COAD	TCGA-AA-A017-01A	A

COAD	TCGA-F4-6855-01A	B
COAD	TCGA-NH-A50U-01A	A
COAD	TCGA-D5-6541-01A	B
COAD	TCGA-D5-6540-01A	A
COAD	TCGA-QG-A5YX-01A	A
COAD	TCGA-AY-4071-01A	B
COAD	TCGA-AA-3831-01A	B
COAD	TCGA-AA-3930-01A	B
COAD	TCGA-AA-3488-01A	A
COAD	TCGA-A6-6653-01A	A
COAD	TCGA-G4-6321-01A	B
COAD	TCGA-AA-A01T-01A	A
COAD	TCGA-AA-3814-01A	B
COAD	TCGA-A6-6648-01A	B
COAD	TCGA-AD-6965-01A	A
COAD	TCGA-AA-A01V-01A	A
COAD	TCGA-AD-5900-01A	B
COAD	TCGA-A6-6650-01A	A
COAD	TCGA-A6-6650-01A	A
COAD	TCGA-A6-5661-01A	B
COAD	TCGA-AA-3866-01A	A
COAD	TCGA-DM-A28A-01A	A
COAD	TCGA-NH-A6GC-01A	B
COAD	TCGA-AA-3986-01A	B
COAD	TCGA-AA-A01S-01A	A
COAD	TCGA-F4-6808-01A	A
COAD	TCGA-AA-3837-01A	B
COAD	TCGA-AD-6889-01A	A
COAD	TCGA-AA-3688-01A	B
COAD	TCGA-A6-4107-01A	B
COAD	TCGA-CM-4750-01A	B
COAD	TCGA-QG-A5Z1-01A	A
COAD	TCGA-AA-3845-01A	B
COAD	TCGA-AA-3980-01A	A
COAD	TCGA-D5-6529-01A	B
COAD	TCGA-A6-5666-01A	A
COAD	TCGA-G4-6295-01A	B
COAD	TCGA-A6-5656-01B	A
COAD	TCGA-A6-3810-01A	A
COAD	TCGA-A6-3810-01A	A
COAD	TCGA-AD-6895-01A	A
COAD	TCGA-AA-A00W-01A	A
COAD	TCGA-AA-A02H-01A	A
COAD	TCGA-CM-5861-01A	A
COAD	TCGA-NH-A5IV-01A	B
COAD	TCGA-A6-6651-01A	B
COAD	TCGA-DM-A28F-01A	A

COAD	TCGA-G4-6627-01A	B
COAD	TCGA-D5-6927-01A	B
COAD	TCGA-AA-3860-01A	B
COAD	TCGA-D5-5537-01A	A
COAD	TCGA-CK-5914-01A	A
COAD	TCGA-CM-5348-01A	B
COAD	TCGA-AA-A02O-01A	A
COAD	TCGA-CK-5915-01A	A
COAD	TCGA-CM-5863-01A	B
COAD	TCGA-AD-6548-01A	B
COAD	TCGA-AA-3502-01A	B
COAD	TCGA-A6-2677-01A	A
COAD	TCGA-AA-3673-01A	A
COAD	TCGA-A6-6649-01A	B
COAD	TCGA-AA-A022-01A	B
COAD	TCGA-CM-6163-01A	B
COAD	TCGA-WS-AB45-01A	B
COAD	TCGA-DM-A1D9-01A	A
COAD	TCGA-CM-6168-01A	B
COAD	TCGA-DM-A1D0-01A	A
COAD	TCGA-AA-3821-01A	A
COAD	TCGA-G4-6625-01A	B
COAD	TCGA-NH-A50T-01A	A
COAD	TCGA-AA-3532-01A	B
COAD	TCGA-AA-3819-01A	B
COAD	TCGA-D5-6930-01A	B
COAD	TCGA-AA-3950-01A	A
COAD	TCGA-A6-3809-01B	B
COAD	TCGA-D5-6530-01A	B
COAD	TCGA-CK-6748-01A	A
COAD	TCGA-AA-3848-01A	A
COAD	TCGA-AA-3850-01A	B
COAD	TCGA-QG-A5YW-01A	B
COAD	TCGA-AA-A03F-01A	B
COAD	TCGA-A6-2682-01A	B
COAD	TCGA-SS-A7HO-01A	A
COAD	TCGA-AA-A029-01A	A
COAD	TCGA-A6-6781-01A	B
COAD	TCGA-A6-6781-01A	B
COAD	TCGA-AA-3556-01A	B
COAD	TCGA-AU-6004-01A	B
COAD	TCGA-A6-6142-01A	A
COAD	TCGA-QG-A5Z2-01A	B
COAD	TCGA-D5-6922-01A	A
COAD	TCGA-AZ-5407-01A	B
COAD	TCGA-D5-6898-01A	A
COAD	TCGA-AA-3680-01A	B

COAD	TCGA-D5-5540-01A	A
COAD	TCGA-A6-4105-01A	B
COAD	TCGA-CM-6677-01A	B
COAD	TCGA-CM-6676-01A	A
COAD	TCGA-AA-3524-01A	A
COAD	TCGA-A6-A566-01A	B
COAD	TCGA-AY-A8YK-01A	A
COAD	TCGA-AA-3692-01A	B
COAD	TCGA-CM-6171-01A	A
COAD	TCGA-AA-3846-01A	A
COAD	TCGA-AA-A00J-01A	A
COAD	TCGA-A6-A56B-01A	A
COAD	TCGA-A6-6140-01A	A
COAD	TCGA-DM-A1D4-01A	A
COAD	TCGA-CM-6170-01A	A
COAD	TCGA-CM-6165-01A	A
COAD	TCGA-AY-6197-01A	A
COAD	TCGA-G4-6299-01A	B
COAD	TCGA-AA-A01I-01A	A
COAD	TCGA-AA-3684-01A	B
COAD	TCGA-AY-A54L-01A	A
COAD	TCGA-A6-2684-01A	B
COAD	TCGA-A6-2684-01A	B
COAD	TCGA-AA-3815-01A	B
COAD	TCGA-AA-3509-01A	A
COAD	TCGA-DM-A0XF-01A	A
COAD	TCGA-CM-6166-01A	A
COAD	TCGA-AA-3854-01A	B
COAD	TCGA-A6-2681-01A	B
COAD	TCGA-AA-3977-01A	A
COAD	TCGA-AA-A01G-01A	A
COAD	TCGA-AA-3520-01A	B
COAD	TCGA-CM-5349-01A	A
COAD	TCGA-G4-6588-01A	A
COAD	TCGA-AY-5543-01A	A
COAD	TCGA-5M-AAT6-01A	B
COAD	TCGA-A6-A567-01A	A
COAD	TCGA-AZ-6598-01A	A
COAD	TCGA-RU-A8FL-01A	A
COAD	TCGA-AA-3554-01A	B
COAD	TCGA-A6-A5ZU-01A	A
COAD	TCGA-AZ-4315-01A	A
COAD	TCGA-AD-A5EJ-01A	A
COAD	TCGA-AA-A00U-01A	A
COAD	TCGA-AA-3506-01A	B
COAD	TCGA-NH-A8F7-06A	A
COAD	TCGA-AA-3947-01A	A

COAD	TCGA-A6-6780-01A	B
COAD	TCGA-A6-6780-01A	B
COAD	TCGA-A6-5659-01A	A
COAD	TCGA-A6-5659-01A	A
COAD	TCGA-AZ-4616-01A	A
COAD	TCGA-AA-3877-01A	A
COAD	TCGA-AA-3543-01A	B
COAD	TCGA-AA-3681-01A	B
COAD	TCGA-AA-3693-01A	A
COAD	TCGA-A6-6780-01B	B
COAD	TCGA-DM-A28H-01A	A
COAD	TCGA-AA-3518-01A	B
COAD	TCGA-AA-3561-01A	A
COAD	TCGA-AM-5820-01A	A
COAD	TCGA-AA-3872-01A	B
COAD	TCGA-G4-6628-01A	B
COAD	TCGA-DM-A28C-01A	A
COAD	TCGA-AA-A00N-01A	A
COAD	TCGA-CK-6751-01A	A
COAD	TCGA-NH-A8F8-01A	A
COAD	TCGA-F4-6461-01A	B
COAD	TCGA-5M-AATA-01A	A
COAD	TCGA-CK-4948-01B	A
COAD	TCGA-AA-A01C-01A	A
COAD	TCGA-AA-A01F-01A	A
COAD	TCGA-G4-6314-01A	A
COAD	TCGA-DM-A285-01A	A
COAD	TCGA-A6-5660-01A	A
COAD	TCGA-CA-6717-01A	B
COAD	TCGA-CM-6678-01A	A
COAD	TCGA-CK-4951-01A	A
COAD	TCGA-AY-4070-01A	B
COAD	TCGA-A6-5665-01B	A
COAD	TCGA-AZ-6601-01A	B
COAD	TCGA-D5-5541-01A	A
COAD	TCGA-DM-A1D7-01A	A
COAD	TCGA-CA-5255-01A	A
COAD	TCGA-D5-6932-01A	A
COAD	TCGA-AZ-6608-01A	A
COAD	TCGA-G4-6309-01A	A
COAD	TCGA-F4-6703-01A	B
COAD	TCGA-CK-5913-01A	A
COAD	TCGA-AA-3521-01A	B
COAD	TCGA-G4-6310-01A	A
COAD	TCGA-AA-3679-01A	B
COAD	TCGA-AA-3492-01A	A
COAD	TCGA-CM-4746-01A	A

COAD	TCGA-CM-6169-01A	B
COAD	TCGA-AA-A01P-01A	B
COAD	TCGA-AA-3489-01A	B
COAD	TCGA-DM-A282-01A	A
COAD	TCGA-F4-6704-01A	B
COAD	TCGA-AA-3560-01A	B
COAD	TCGA-AA-A00R-01A	B
COAD	TCGA-CM-4751-01A	B
COAD	TCGA-A6-2686-01A	B
COAD	TCGA-F4-6806-01A	B
COAD	TCGA-D5-6537-01A	A
COAD	TCGA-A6-2674-01A	B
COAD	TCGA-A6-2674-01A	B
COAD	TCGA-A6-2677-01B	A
COAD	TCGA-CA-5256-01A	A
COAD	TCGA-F4-6459-01A	A
COAD	TCGA-A6-2685-01A	B
COAD	TCGA-AA-A02Y-01A	A
COAD	TCGA-A6-2676-01A	A
COAD	TCGA-D5-5539-01A	B
COAD	TCGA-AA-3952-01A	B
COAD	TCGA-AM-5821-01A	A
COAD	TCGA-AA-A00D-01A	B
COAD	TCGA-AA-3851-01A	B
COAD	TCGA-CM-5862-01A	A
COAD	TCGA-NH-A6GA-01A	B
COAD	TCGA-AA-3870-01A	B
COAD	TCGA-D5-6926-01A	A
COAD	TCGA-CM-5860-01A	B
COAD	TCGA-A6-2672-01A	A
COAD	TCGA-AA-A01Q-01A	A
COAD	TCGA-DM-A288-01A	A
COAD	TCGA-AA-3678-01A	B
COAD	TCGA-AA-3812-01A	B
COAD	TCGA-CM-4748-01A	B
COAD	TCGA-D5-6536-01A	A
COAD	TCGA-DM-A1DA-01A	A
COAD	TCGA-AA-A004-01A	B
COAD	TCGA-CK-5916-01A	B
COAD	TCGA-AY-A71X-01A	A
COAD	TCGA-CM-4744-01A	A
COAD	TCGA-CM-6161-01A	A
COAD	TCGA-A6-5656-01A	A
COAD	TCGA-A6-5656-01A	A
COAD	TCGA-G4-6302-01A	B
COAD	TCGA-AA-A01R-01A	A
COAD	TCGA-AA-3712-01A	B

COAD	TCGA-CA-6716-01A	A
COAD	TCGA-AA-3864-01A	A
COAD	TCGA-AA-3975-01A	B
COAD	TCGA-D5-6534-01A	B
COAD	TCGA-5M-AATE-01A	A
COAD	TCGA-A6-5667-01A	A
COAD	TCGA-A6-6652-01A	A
COAD	TCGA-AA-3971-01A	B
COAD	TCGA-CM-6680-01A	B
COAD	TCGA-A6-6141-01A	B
COAD	TCGA-AA-3494-01A	A
COAD	TCGA-AA-A00A-01A	A
GBM	TCGA-27-2521-01A	B
GBM	TCGA-19-0957-02A	A
GBM	TCGA-26-5139-01A	B
GBM	TCGA-19-2620-01A	B
GBM	TCGA-02-2483-01A	B
GBM	TCGA-28-2499-01A	B
GBM	TCGA-14-1034-02B	B
GBM	TCGA-06-0174-01A	A
GBM	TCGA-06-0125-02A	B
GBM	TCGA-06-0238-01A	A
GBM	TCGA-76-4928-01B	B
GBM	TCGA-14-1825-01A	B
GBM	TCGA-76-4927-01A	B
GBM	TCGA-14-1034-01A	B
GBM	TCGA-06-0747-01A	B
GBM	TCGA-06-0130-01A	B
GBM	TCGA-06-0125-01A	B
GBM	TCGA-28-2514-01A	A
GBM	TCGA-06-0158-01A	B
GBM	TCGA-06-0139-01A	B
GBM	TCGA-76-4925-01A	B
GBM	TCGA-19-2629-01A	B
GBM	TCGA-06-0743-01A	B
GBM	TCGA-06-5412-01A	B
GBM	TCGA-28-5207-01A	A
GBM	TCGA-28-5204-01A	B
GBM	TCGA-14-0781-01B	B
GBM	TCGA-27-1832-01A	B
GBM	TCGA-06-5408-01A	B
GBM	TCGA-32-2632-01A	B
GBM	TCGA-06-2557-01A	B
GBM	TCGA-76-4931-01A	B
GBM	TCGA-06-2565-01A	B
GBM	TCGA-19-5960-01A	A
GBM	TCGA-06-5411-01A	A

GBM	TCGA-06-0745-01A	B
GBM	TCGA-41-5651-01A	A
GBM	TCGA-41-2571-01A	A
GBM	TCGA-28-1753-01A	B
GBM	TCGA-27-1830-01A	B
GBM	TCGA-12-3653-01A	B
GBM	TCGA-28-5218-01A	B
GBM	TCGA-19-1389-02A	B
GBM	TCGA-06-2559-01A	A
GBM	TCGA-26-5133-01A	B
GBM	TCGA-06-0168-01A	A
GBM	TCGA-19-2619-01A	B
GBM	TCGA-26-5132-01A	B
GBM	TCGA-06-0686-01A	A
GBM	TCGA-28-5215-01A	B
GBM	TCGA-06-0210-01A	B
GBM	TCGA-06-1804-01A	B
GBM	TCGA-06-0190-01A	B
GBM	TCGA-14-2554-01A	B
GBM	TCGA-06-0882-01A	B
GBM	TCGA-41-3915-01A	B
GBM	TCGA-32-5222-01A	B
GBM	TCGA-14-1402-02A	B
GBM	TCGA-06-0184-01A	B
GBM	TCGA-26-5134-01A	A
GBM	TCGA-06-5859-01A	B
GBM	TCGA-27-1831-01A	B
GBM	TCGA-02-0047-01A	A
GBM	TCGA-06-5413-01A	B
GBM	TCGA-28-5213-01A	B
GBM	TCGA-06-0649-01B	B
GBM	TCGA-14-0790-01B	B
GBM	TCGA-26-1442-01A	A
GBM	TCGA-06-2569-01A	B
GBM	TCGA-06-0132-01A	A
GBM	TCGA-28-2509-01A	B
GBM	TCGA-14-1829-01A	B
GBM	TCGA-06-2561-01A	B
GBM	TCGA-28-5209-01A	B
GBM	TCGA-27-1835-01A	B
GBM	TCGA-28-1747-01C	B
GBM	TCGA-26-5135-01A	B
GBM	TCGA-32-1982-01A	B
GBM	TCGA-06-0129-01A	B
GBM	TCGA-14-1823-01A	B
GBM	TCGA-76-4932-01A	B
GBM	TCGA-27-1837-01A	B

GBM	TCGA-06-0178-01A	A
GBM	TCGA-32-2615-01A	B
GBM	TCGA-06-0187-01A	B
GBM	TCGA-06-0219-01A	B
GBM	TCGA-06-2567-01A	B
GBM	TCGA-06-5858-01A	B
GBM	TCGA-12-5299-01A	B
GBM	TCGA-27-2524-01A	B
GBM	TCGA-06-5414-01A	B
GBM	TCGA-12-5295-01A	B
GBM	TCGA-28-5208-01A	B
GBM	TCGA-32-1970-01A	B
GBM	TCGA-06-0171-02A	B
GBM	TCGA-06-2558-01A	A
GBM	TCGA-19-2624-01A	A
GBM	TCGA-12-3650-01A	B
GBM	TCGA-06-2564-01A	B
GBM	TCGA-06-0644-01A	B
GBM	TCGA-27-2523-01A	B
GBM	TCGA-28-5216-01A	A
GBM	TCGA-06-5416-01A	A
GBM	TCGA-32-2638-01A	B
GBM	TCGA-12-3652-01A	B
GBM	TCGA-12-0618-01A	A
GBM	TCGA-14-0789-01A	B
GBM	TCGA-27-2528-01A	B
GBM	TCGA-08-0386-01A	B
GBM	TCGA-32-1980-01A	A
GBM	TCGA-12-1597-01B	A
GBM	TCGA-06-0646-01A	A
GBM	TCGA-06-0221-02A	A
GBM	TCGA-14-0871-01A	B
GBM	TCGA-19-1787-01B	B
GBM	TCGA-06-0152-02A	B
GBM	TCGA-14-0787-01A	B
GBM	TCGA-06-5417-01A	B
GBM	TCGA-02-0055-01A	B
GBM	TCGA-12-0619-01A	B
GBM	TCGA-28-5220-01A	B
GBM	TCGA-12-0616-01A	B
GBM	TCGA-32-2616-01A	B
GBM	TCGA-27-2526-01A	B
GBM	TCGA-19-4065-01A	B
GBM	TCGA-76-4926-01B	B
GBM	TCGA-14-0736-02A	A
GBM	TCGA-06-0141-01A	B
GBM	TCGA-16-1045-01B	B

GBM	TCGA-32-2634-01A	A
GBM	TCGA-41-2572-01A	B
GBM	TCGA-28-2510-01A	A
GBM	TCGA-02-2485-01A	B
GBM	TCGA-16-0846-01A	A
GBM	TCGA-06-5856-01A	B
GBM	TCGA-14-0817-01A	B
GBM	TCGA-19-1390-01A	B
GBM	TCGA-06-0157-01A	B
GBM	TCGA-06-0190-02A	B
GBM	TCGA-12-0821-01A	A
GBM	TCGA-06-0750-01A	B
GBM	TCGA-06-0156-01A	B
GBM	TCGA-06-0156-01A	B
GBM	TCGA-28-2513-01A	B
GBM	TCGA-02-2486-01A	B
GBM	TCGA-27-1834-01A	A
GBM	TCGA-06-0744-01A	B
GBM	TCGA-32-4213-01A	B
GBM	TCGA-06-5410-01A	B
GBM	TCGA-06-2563-01A	B
GBM	TCGA-06-0749-01A	A
GBM	TCGA-19-4065-02A	A
GBM	TCGA-19-2625-01A	B
GBM	TCGA-06-5418-01A	B
GBM	TCGA-41-4097-01A	A
GBM	TCGA-06-0211-01B	B
GBM	TCGA-06-0138-01A	A
GBM	TCGA-06-0878-01A	B
GBM	TCGA-06-0645-01A	B
GBM	TCGA-27-2519-01A	B
GBM	TCGA-26-5136-01B	B
GBM	TCGA-06-2570-01A	A
GBM	TCGA-15-0742-01A	B
GBM	TCGA-15-1444-01A	B
GBM	TCGA-06-0211-02A	B
GBM	TCGA-76-4929-01A	B
GBM	TCGA-06-0210-02A	B
GBM	TCGA-06-2562-01A	B
GBM	TCGA-06-0211-01A	B
KIRP	TCGA-GL-A4EM-01A	B
KIRP	TCGA-A4-7584-01A	A
KIRP	TCGA-IZ-8195-01A	A
KIRP	TCGA-HE-7129-01A	A
KIRP	TCGA-IA-A40U-01A	B
KIRP	TCGA-B9-4116-01A	B
KIRP	TCGA-B9-4115-01A	A

KIRP	TCGA-P4-A5EA-01A	B
KIRP	TCGA-BQ-7062-01A	A
KIRP	TCGA-HE-7128-01A	B
KIRP	TCGA-B9-4617-01A	A
KIRP	TCGA-DW-7839-01A	B
KIRP	TCGA-AL-A5DJ-01A	B
KIRP	TCGA-UZ-A9PV-01A	A
KIRP	TCGA-V9-A7HT-01A	B
KIRP	TCGA-AL-3466-01A	B
KIRP	TCGA-B9-A69E-01A	B
KIRP	TCGA-BQ-5875-01A	B
KIRP	TCGA-GL-7966-01A	B
KIRP	TCGA-UZ-A9Q0-01A	A
KIRP	TCGA-2Z-A9J8-01A	A
KIRP	TCGA-5P-A9JW-01A	A
KIRP	TCGA-BQ-5887-01A	B
KIRP	TCGA-BQ-7050-01A	A
KIRP	TCGA-2Z-A9J3-01A	A
KIRP	TCGA-A4-A57E-01A	A
KIRP	TCGA-B9-A44B-01A	A
KIRP	TCGA-UZ-A9PN-01A	B
KIRP	TCGA-UZ-A9PM-01A	B
KIRP	TCGA-DW-7834-01A	A
KIRP	TCGA-DW-5561-01A	A
KIRP	TCGA-BQ-7046-01A	A
KIRP	TCGA-UN-AAZ9-01A	B
KIRP	TCGA-SX-A71R-01A	A
KIRP	TCGA-UZ-A9Q1-01A	A
KIRP	TCGA-G7-A8LE-01A	B
KIRP	TCGA-A4-A5Y0-01A	A
KIRP	TCGA-BQ-7045-01A	B
KIRP	TCGA-SX-A7SO-01A	A
KIRP	TCGA-G7-6796-01A	A
KIRP	TCGA-B1-7332-01A	A
KIRP	TCGA-IZ-A6M8-01A	A
KIRP	TCGA-5P-A9K2-01A	A
KIRP	TCGA-5P-A9K4-01A	B
KIRP	TCGA-2Z-A9JE-01A	A
KIRP	TCGA-UZ-A9PS-05A	A
KIRP	TCGA-5P-A9K9-01A	B
KIRP	TCGA-B1-A656-01A	B
KIRP	TCGA-Y8-A894-01A	A
KIRP	TCGA-2Z-A9J2-01A	A
KIRP	TCGA-A4-A5Y1-01A	A
KIRP	TCGA-AL-3472-01A	A
KIRP	TCGA-SX-A7SL-01A	A
KIRP	TCGA-DW-7838-01A	B

KIRP	TCGA-B3-4104-01A	B
KIRP	TCGA-B1-A47O-01A	B
KIRP	TCGA-PJ-A5Z9-01A	A
KIRP	TCGA-HE-A5NL-01A	A
KIRP	TCGA-DW-7837-01A	A
KIRP	TCGA-A4-8311-01A	A
KIRP	TCGA-A4-A4ZT-01A	A
KIRP	TCGA-EV-5902-01A	A
KIRP	TCGA-B9-A5W9-01A	A
KIRP	TCGA-IA-A83S-01A	A
KIRP	TCGA-DW-7841-01A	B
KIRP	TCGA-A4-A6HP-01A	A
KIRP	TCGA-B3-3925-01A	B
KIRP	TCGA-AL-3467-01A	B
KIRP	TCGA-UZ-A9PR-01A	A
KIRP	TCGA-F9-A7Q0-01A	B
KIRP	TCGA-MH-A55W-01A	A
KIRP	TCGA-B9-5155-01A	A
KIRP	TCGA-G7-A8LB-01A	A
KIRP	TCGA-BQ-5883-01A	B
KIRP	TCGA-HE-A5NJ-01A	A
KIRP	TCGA-DW-7840-01A	A
KIRP	TCGA-SX-A7SM-01A	A
KIRP	TCGA-BQ-5889-01A	B
KIRP	TCGA-B9-4117-01A	B
KIRP	TCGA-Y8-A895-01A	A
KIRP	TCGA-Y8-A896-01A	B
KIRP	TCGA-5P-A9JV-01A	A
KIRP	TCGA-BQ-7044-01A	B
KIRP	TCGA-UZ-A9PS-01A	A
KIRP	TCGA-GL-6846-01A	A
KIRP	TCGA-G7-7502-01A	B
KIRP	TCGA-SX-A7SU-01A	A
KIRP	TCGA-AL-3468-01A	A
KIRP	TCGA-IZ-8196-01A	A
KIRP	TCGA-5P-A9K6-01A	A
KIRP	TCGA-2Z-A9JS-01A	A
KIRP	TCGA-BQ-7053-01A	B
KIRP	TCGA-4A-A93X-01A	A
KIRP	TCGA-5P-A9KC-01A	A
KIRP	TCGA-J7-6720-01A	B
KIRP	TCGA-2Z-A9JL-01A	B
KIRP	TCGA-AT-A5NU-01A	B
KIRP	TCGA-BQ-5890-01A	A
KIRP	TCGA-J7-A8I2-01A	B
KIRP	TCGA-SX-A71S-01A	A
KIRP	TCGA-AL-3471-01A	A

KIRP	TCGA-B1-A47N-01A	A
KIRP	TCGA-A4-8516-01A	A
KIRP	TCGA-UZ-A9PX-01A	A
KIRP	TCGA-B9-5156-01A	A
KIRP	TCGA-DW-7836-01A	A
KIRP	TCGA-5P-A9KF-01A	A
KIRP	TCGA-P4-A5E7-01A	B
KIRP	TCGA-IA-A83V-01A	B
KIRP	TCGA-BQ-7058-01A	A
KIRP	TCGA-A4-8098-01A	A
KIRP	TCGA-BQ-5879-01A	B
KIRP	TCGA-MH-A855-01A	B
KIRP	TCGA-4A-A93W-01A	B
KIRP	TCGA-2Z-A9JN-01A	B
KIRP	TCGA-SX-A7SS-01A	A
KIRP	TCGA-A4-A7UZ-01A	B
KIRP	TCGA-Y8-A898-01A	A
KIRP	TCGA-IA-A40X-01A	A
KIRP	TCGA-HE-A5NK-01A	B
KIRP	TCGA-BQ-5882-01A	B
KIRP	TCGA-SX-A7SQ-01A	B
KIRP	TCGA-P4-AAVL-01A	A
KIRP	TCGA-MH-A856-01A	B
KIRP	TCGA-B9-A8YI-01A	A
KIRP	TCGA-2Z-A9JJ-01A	B
KIRP	TCGA-O9-A75Z-01A	A
KIRP	TCGA-PJ-A5Z8-01A	B
KIRP	TCGA-A4-8312-01A	B
KIRP	TCGA-EV-5901-01A	B
KIRP	TCGA-5P-A9KA-01A	B
KIRP	TCGA-BQ-5893-01A	B
KIRP	TCGA-2Z-A9JP-01A	A
KIRP	TCGA-5P-A9KH-01A	B
KIRP	TCGA-DZ-6135-01A	B
KIRP	TCGA-GL-A59R-01A	A
KIRP	TCGA-A4-7583-01A	A
KIRP	TCGA-KV-A6GD-01A	B
KIRP	TCGA-A4-7287-01A	B
KIRP	TCGA-B9-A8YH-01A	A
KIRP	TCGA-DW-5560-01A	A
KIRP	TCGA-2Z-A9JM-01A	A
KIRP	TCGA-5P-A9JY-01A	A
KIRP	TCGA-P4-AAVO-01A	A
KIRP	TCGA-BQ-5880-01A	B
KIRP	TCGA-BQ-7061-01A	A
KIRP	TCGA-G7-6793-01A	B
KIRP	TCGA-A4-7732-01A	A

KIRP	TCGA-B1-A654-01A	B
KIRP	TCGA-A4-8517-01A	A
KIRP	TCGA-BQ-5888-01A	B
KIRP	TCGA-IA-A83T-01A	B
KIRP	TCGA-WN-AB4C-01A	A
KIRP	TCGA-2Z-A9J1-01A	B
KIRP	TCGA-GL-7773-01A	B
KIRP	TCGA-5P-A9KE-01A	A
KIRP	TCGA-5P-A9K0-01A	A
KIRP	TCGA-B1-5398-01A	B
KIRP	TCGA-Y8-A897-01A	A
KIRP	TCGA-B1-A47M-01A	B
KIRP	TCGA-DW-7963-01B	A
KIRP	TCGA-B3-8121-01A	B
KIRP	TCGA-KV-A6GE-01A	A
KIRP	TCGA-P4-AAVK-01A	A
KIRP	TCGA-A4-A772-01A	A
KIRP	TCGA-UZ-A9PZ-01A	A
KIRP	TCGA-HE-A5NF-01A	B
KIRP	TCGA-G7-A8LD-01A	B
KIRP	TCGA-SX-A7SR-01A	A
KIRP	TCGA-A4-8630-01A	A
KIRP	TCGA-MH-A562-01A	A
KIRP	TCGA-MH-A561-01A	B
KIRP	TCGA-B9-A5W7-01A	A
KIRP	TCGA-HE-A5NI-01A	A
KIRP	TCGA-A4-7996-01A	A
KIRP	TCGA-BQ-5876-01A	A
KIRP	TCGA-2Z-A9J9-01A	B
KIRP	TCGA-2Z-A9JD-01A	A
KIRP	TCGA-2Z-A9JQ-01A	A
KIRP	TCGA-B3-A6W5-01A	A
KIRP	TCGA-BQ-7048-01A	B
KIRP	TCGA-WN-A9G9-01A	A
KIRP	TCGA-A4-7286-01A	B
KIRP	TCGA-MH-A560-01A	A
KIRP	TCGA-BQ-7049-01A	B
KIRP	TCGA-5P-A9JZ-01A	A
KIRP	TCGA-P4-AAVM-01A	A
KIRP	TCGA-A4-8310-01A	A
KIRP	TCGA-UZ-A9PU-01A	A
KIRP	TCGA-A4-7828-01A	B
KIRP	TCGA-P4-A5E6-01A	A
KIRP	TCGA-BQ-5886-01A	A
KIRP	TCGA-SX-A71W-01A	A
KIRP	TCGA-BQ-5881-01A	A
KIRP	TCGA-UZ-A9PL-01A	A

KIRP	TCGA-A4-8515-01A	A
KIRP	TCGA-P4-A5EB-01A	A
KIRP	TCGA-BQ-7060-01A	A
KIRP	TCGA-UZ-A9PK-01A	A
KIRP	TCGA-A4-A5XZ-01A	A
KIRP	TCGA-5P-A9K8-01A	B
KIRP	TCGA-G7-6795-01A	A
KIRP	TCGA-BQ-5878-01A	A
KIRP	TCGA-G7-6790-01A	A
KIRP	TCGA-SX-A7SN-01A	A
KIRP	TCGA-BQ-5885-01A	A
KIRP	TCGA-BQ-7055-01A	B
KIRP	TCGA-EV-5903-01A	B
KIRP	TCGA-F9-A97G-01A	B
KIRP	TCGA-MH-A854-01A	B
KIRP	TCGA-DZ-6134-01A	B
KIRP	TCGA-B9-A5W8-01A	B
KIRP	TCGA-GL-A9DC-01A	A
KIRP	TCGA-4A-A93Y-01A	B
KIRP	TCGA-G7-6789-01A	B
KIRP	TCGA-G7-6797-01A	A
KIRP	TCGA-SX-A71U-01A	A
KIRP	TCGA-P4-A5ED-01A	B
KIRP	TCGA-A4-7997-01A	A
KIRP	TCGA-G7-A8LC-01A	A
KIRP	TCGA-G7-7501-01A	A
KIRP	TCGA-B3-4103-01A	A
KIRP	TCGA-A4-A5DU-01A	A
KIRP	TCGA-DZ-6133-01A	A
KIRP	TCGA-KV-A74V-01A	A
KIRP	TCGA-A4-7734-01A	B
KIRP	TCGA-G7-6792-01A	B
KIRP	TCGA-BQ-5892-01A	A
KIRP	TCGA-AL-7173-01A	B
KIRP	TCGA-BQ-5884-01A	B
KIRP	TCGA-IA-A83W-01A	B
KIRP	TCGA-Q2-A5QZ-01A	B
KIRP	TCGA-BQ-7056-01A	A
KIRP	TCGA-A4-A48D-01A	A
KIRP	TCGA-GL-A59T-01A	A
KIRP	TCGA-UZ-A9PP-01A	A
KIRP	TCGA-B1-A657-01A	A
KIRP	TCGA-Y8-A8RZ-01A	A
KIRP	TCGA-2Z-A9JO-01A	B
KIRP	TCGA-5P-A9K3-01A	A
KIRP	TCGA-B9-4113-01A	B
KIRP	TCGA-G7-A4TM-01A	B

KIRP	TCGA-SX-A7SP-01A	A
KIRP	TCGA-BQ-5877-01A	B
KIRP	TCGA-F9-A8NY-01A	B
KIRP	TCGA-BQ-7059-01A	B
KIRP	TCGA-2K-A9WE-01A	B
KIRP	TCGA-UZ-A9PQ-01A	A
KIRP	TCGA-2Z-A9J5-01A	B
KIRP	TCGA-A4-7288-01A	A
KIRP	TCGA-Y8-A8S1-01A	B
KIRP	TCGA-GL-A9DD-01A	B
KIRP	TCGA-B3-3926-01A	A
KIRP	TCGA-HE-A5NH-01A	A
KIRP	TCGA-UZ-A9PJ-01A	A
KIRP	TCGA-F9-A7VF-01A	A
KIRP	TCGA-A4-8518-01A	A
KIRP	TCGA-HE-7130-01A	B
KIRP	TCGA-2Z-A9J7-01A	A
KIRP	TCGA-Y8-A8S0-01A	A
KIRP	TCGA-A4-7915-01A	B
KIRP	TCGA-DZ-6132-01A	B
KIRP	TCGA-MH-A55Z-01A	A
KIRP	TCGA-DW-7842-01A	A
KIRP	TCGA-B9-7268-01A	A
KIRP	TCGA-P4-A5E8-01A	B
KIRP	TCGA-2Z-A9JT-01A	A
KIRP	TCGA-GL-A9DE-01A	A
KIRP	TCGA-2Z-A9JR-01A	A
KIRP	TCGA-IA-A40Y-01A	B
KIRP	TCGA-UZ-A9PO-01A	A
KIRP	TCGA-F9-A4JJ-01A	B
KIRP	TCGA-2Z-A9JK-01A	B
KIRP	TCGA-Y8-A8RY-01A	A
KIRP	TCGA-J7-8537-01A	B
KIRP	TCGA-BQ-7051-01A	A
KIRP	TCGA-5P-A9JU-01A	A
KIRP	TCGA-2Z-A9JG-01A	B
KIRP	TCGA-AL-3473-01A	A
KIRP	TCGA-B1-A655-01A	A
KIRP	TCGA-BQ-5894-01A	B
KIRP	TCGA-IZ-A6M9-01A	A
KIRP	TCGA-A4-7585-01A	B
KIRP	TCGA-2Z-A9J6-01A	A
KIRP	TCGA-SX-A71V-01A	A
KIRP	TCGA-2Z-A9JI-01A	B
KIRP	TCGA-MH-A857-01A	B
KIRP	TCGA-GL-8500-01A	A
KIRP	TCGA-BQ-5891-01A	A

PAAD	TCGA-IB-7646-01A	B
PAAD	TCGA-IB-A5SQ-01A	B
PAAD	TCGA-S4-A8RP-01A	B
PAAD	TCGA-H6-8124-01A	B
PAAD	TCGA-HZ-8317-01A	B
PAAD	TCGA-F2-A8YN-01A	B
PAAD	TCGA-IB-AAUN-01A	B
PAAD	TCGA-FB-AAQ0-01A	B
PAAD	TCGA-HZ-8638-01A	B
PAAD	TCGA-US-A77G-01A	B
PAAD	TCGA-HZ-A49I-01A	B
PAAD	TCGA-IB-7649-01A	B
PAAD	TCGA-IB-A7LX-01A	B
PAAD	TCGA-RL-AAAS-01A	B
PAAD	TCGA-2J-AABI-01A	B
PAAD	TCGA-IB-A6UG-01A	B
PAAD	TCGA-IB-7893-01A	B
PAAD	TCGA-2L-AAQL-01A	B
PAAD	TCGA-FB-AAPY-01A	B
PAAD	TCGA-FB-A545-01A	B
PAAD	TCGA-IB-A6UF-01A	B
PAAD	TCGA-IB-7889-01A	B
PAAD	TCGA-3A-A9IC-01A	B
PAAD	TCGA-IB-AAUT-01A	B
PAAD	TCGA-HV-AA8X-01A	B
PAAD	TCGA-IB-7651-01A	B
PAAD	TCGA-2J-AABA-01A	B
PAAD	TCGA-Z5-AAPL-01A	B
PAAD	TCGA-2L-AAQA-01A	B
PAAD	TCGA-H6-A45N-01A	B
PAAD	TCGA-IB-AAUV-01A	B
PAAD	TCGA-3E-AAAY-01A	B
PAAD	TCGA-IB-7644-01A	B
PAAD	TCGA-YY-A8LH-01A	B
PAAD	TCGA-LB-A8F3-01A	B
PAAD	TCGA-HZ-8315-01A	B
PAAD	TCGA-IB-AAUP-01A	B
PAAD	TCGA-FB-A78T-01A	B
PAAD	TCGA-IB-AAUU-01A	B
PAAD	TCGA-2L-AAQJ-01A	B
PAAD	TCGA-M8-A5N4-01A	B
PAAD	TCGA-RB-A7B8-01A	B
PAAD	TCGA-IB-A5SP-01A	B
PAAD	TCGA-S4-A8RO-01A	B
PAAD	TCGA-3A-A9IV-01A	A
PAAD	TCGA-FB-AAPQ-01A	B
PAAD	TCGA-IB-A5SO-01A	B

PAAD	TCGA-IB-7652-01A	B
PAAD	TCGA-3A-A9IX-01A	B
PAAD	TCGA-HZ-A8P1-01A	B
PAAD	TCGA-FB-AAPZ-01A	B
PAAD	TCGA-XN-A8T5-01A	B
PAAD	TCGA-2J-AAB1-01A	B
PAAD	TCGA-IB-7885-01A	B
PAAD	TCGA-2J-AABH-01A	B
PAAD	TCGA-2J-AABO-01A	B
PAAD	TCGA-3A-A9J0-01A	B
PAAD	TCGA-IB-7888-01A	B
PAAD	TCGA-F2-6880-01A	B
PAAD	TCGA-HZ-A49H-01A	B
PAAD	TCGA-F2-6879-01A	B
PAAD	TCGA-IB-A7M4-01A	B
PAAD	TCGA-HZ-8002-01A	B
PAAD	TCGA-2J-AAB8-01A	B
PAAD	TCGA-FB-AAQ6-01A	B
PAAD	TCGA-XN-A8T3-01A	B
PAAD	TCGA-2J-AABR-01A	B
PAAD	TCGA-FB-AAPS-01A	B
PAAD	TCGA-HZ-8519-01A	B
PAAD	TCGA-HV-A5A6-01A	B
PAAD	TCGA-2J-AABK-01A	B
PAAD	TCGA-HZ-A77Q-01A	B
PAAD	TCGA-HV-AA8V-01A	B
PAAD	TCGA-3A-A9IO-01A	A
PAAD	TCGA-HZ-7926-01A	B
PAAD	TCGA-HZ-7924-01A	B
PAAD	TCGA-2J-AABP-01A	B
PAAD	TCGA-US-A774-01A	B
PAAD	TCGA-XD-AAUL-01A	B
PAAD	TCGA-2L-AAQM-01A	A
PAAD	TCGA-FB-A4P6-01A	B
PAAD	TCGA-2J-AABV-01A	B
PAAD	TCGA-3A-A9IB-01A	B
PAAD	TCGA-IB-A5ST-01A	B
PAAD	TCGA-FB-A4P5-01A	B
PAAD	TCGA-IB-AAUR-01A	B
PAAD	TCGA-HZ-7289-01A	B
PAAD	TCGA-FB-AAQ1-01A	B
PAAD	TCGA-F2-A44G-01A	B
PAAD	TCGA-US-A77J-01A	B
PAAD	TCGA-IB-7645-01A	B
PAAD	TCGA-HV-A7OL-01A	B
PAAD	TCGA-FB-AAPU-01A	B
PAAD	TCGA-PZ-A5RE-01A	B

PAAD	TCGA-HZ-7920-01A	B
PAAD	TCGA-IB-7887-01A	B
PAAD	TCGA-HZ-A77P-01A	B
PAAD	TCGA-US-A77E-01A	B
PAAD	TCGA-2L-AAQI-01A	B
PAAD	TCGA-3A-A9I7-01A	B
PAAD	TCGA-FB-A5VM-01A	B
PAAD	TCGA-2J-AAB6-01A	B
PAAD	TCGA-3A-A9IU-01A	B
PAAD	TCGA-F2-7276-01A	B
PAAD	TCGA-F2-A44H-01A	B
PAAD	TCGA-2J-AABE-01A	B
PAAD	TCGA-HV-A5A3-01A	B
PAAD	TCGA-HZ-8637-01A	B
PAAD	TCGA-F2-A7TX-01A	B
PAAD	TCGA-IB-7891-01A	B
PAAD	TCGA-IB-7897-01A	B
PAAD	TCGA-HZ-A9TJ-06A	B
PAAD	TCGA-3A-A9IJ-01A	A
PAAD	TCGA-IB-AAUS-01A	B
PAAD	TCGA-IB-8127-01A	B
PAAD	TCGA-HZ-A4BH-01A	B
PAAD	TCGA-HZ-8001-01A	B
PAAD	TCGA-3A-A9IN-01A	A
PAAD	TCGA-3E-AAAZ-01A	B
PAAD	TCGA-LB-A9Q5-01A	B
PAAD	TCGA-FB-AAPP-01A	B
PAAD	TCGA-US-A776-01A	B
PAAD	TCGA-HZ-7922-01A	B
PAAD	TCGA-3A-A9IZ-01A	B
PAAD	TCGA-IB-AAUW-01A	B
PAAD	TCGA-HZ-8005-01A	B
PAAD	TCGA-2J-AAB4-01A	B
PAAD	TCGA-XD-AAUH-01A	B
PAAD	TCGA-2J-AABT-01A	B
PAAD	TCGA-IB-A5SS-01A	B
PAAD	TCGA-S4-A8RM-01A	B
PAAD	TCGA-FB-AAQ2-01A	B
PAAD	TCGA-F2-7273-01A	B
PAAD	TCGA-IB-8126-01A	B
PAAD	TCGA-XD-AAUG-01A	B
PAAD	TCGA-HV-A7OP-01A	B
PAAD	TCGA-XD-AAUI-01A	B
PAAD	TCGA-HV-A5A4-01A	B
PAAD	TCGA-H8-A6C1-01A	B
PAAD	TCGA-HV-A5A5-01A	B
PAAD	TCGA-OE-A75W-01A	B

PAAD	TCGA-US-A779-01A	B
PAAD	TCGA-RB-AA9M-01A	B
PAAD	TCGA-IB-7886-01A	B
PAAD	TCGA-IB-AAUO-01A	B
PAAD	TCGA-2J-AABU-01A	B
PAAD	TCGA-HZ-8636-01A	B
PAAD	TCGA-HZ-A9TJ-01A	B
PAAD	TCGA-LB-A7SX-01A	B
PAAD	TCGA-IB-7890-01A	B
PAAD	TCGA-HZ-A49G-01A	B
PAAD	TCGA-3A-A9IL-01A	A
PAAD	TCGA-YB-A89D-01A	B
PAAD	TCGA-HZ-7925-01A	B
PAAD	TCGA-3A-A9IS-01A	A
PAAD	TCGA-2J-AAB9-01A	B
PAAD	TCGA-3A-A9IH-01A	B
PAAD	TCGA-IB-7654-01A	B
PAAD	TCGA-HZ-8003-01A	B
PAAD	TCGA-FB-AAQ3-01A	B
PAAD	TCGA-2J-AABF-01A	B
PAAD	TCGA-YH-A8SY-01A	B
PAAD	TCGA-HZ-A4BK-01A	B
PAAD	TCGA-3A-A9I5-01A	B
PAAD	TCGA-Q3-A5QY-01A	B
PAAD	TCGA-HZ-7918-01A	B
PAAD	TCGA-3A-A9I9-01A	B
PAAD	TCGA-2L-AAQE-01A	B
PAAD	TCGA-HZ-A77O-01A	B
PAAD	TCGA-L1-A7W4-01A	B
PAAD	TCGA-HZ-7923-01A	B
PAAD	TCGA-HZ-7919-01A	B
PAAD	TCGA-Q3-AA2A-01A	B
PAAD	TCGA-IB-AAUM-01A	B
PAAD	TCGA-HZ-A8P0-01A	B
PAAD	TCGA-IB-AAUQ-01A	B
PAAD	TCGA-FB-A7DR-01A	B
PAAD	TCGA-3A-A9IR-01A	A
PCPG	TCGA-QR-A7IP-01A	A
PCPG	TCGA-QR-A70I-01A	A
PCPG	TCGA-TT-A6YJ-01A	A
PCPG	TCGA-W2-A7H7-01A	A
PCPG	TCGA-S7-A7WX-01A	A
PCPG	TCGA-SR-A6MX-05A	A
PCPG	TCGA-SP-A6QF-01A	A
PCPG	TCGA-QR-A70X-01A	B
PCPG	TCGA-WB-A80Y-01A	A
PCPG	TCGA-WB-A81N-01A	A

PCPG	TCGA-QR-A70C-01A	A
PCPG	TCGA-TT-A6YP-01A	A
PCPG	TCGA-QT-A5XM-01A	A
PCPG	TCGA-PR-A5PG-01A	A
PCPG	TCGA-SP-A6QH-01A	A
PCPG	TCGA-P7-A5NX-01A	A
PCPG	TCGA-SR-A6N0-01A	B
PCPG	TCGA-SR-A6MS-01A	A
PCPG	TCGA-QR-A705-01A	A
PCPG	TCGA-RW-A8AZ-01A	A
PCPG	TCGA-RT-A6Y9-01A	A
PCPG	TCGA-W2-A7HF-01A	A
PCPG	TCGA-SP-A6QI-01A	A
PCPG	TCGA-QR-A70R-01A	A
PCPG	TCGA-WB-A81W-01A	A
PCPG	TCGA-WB-A80V-01A	A
PCPG	TCGA-QR-A707-01A	A
PCPG	TCGA-QR-A708-01A	A
PCPG	TCGA-SR-A6MR-01A	A
PCPG	TCGA-SP-A6QK-01A	B
PCPG	TCGA-QR-A6ZZ-01A	A
PCPG	TCGA-W2-A7HH-01A	A
PCPG	TCGA-WB-A818-01A	A
PCPG	TCGA-WB-A820-01A	A
PCPG	TCGA-RM-A68T-01A	A
PCPG	TCGA-SR-A6MU-01A	B
PCPG	TCGA-QR-A6GO-01A	A
PCPG	TCGA-QR-A6H2-01A	A
PCPG	TCGA-WB-A822-01A	A
PCPG	TCGA-QR-A6H6-01A	A
PCPG	TCGA-QR-A70M-01A	A
PCPG	TCGA-QR-A70N-01A	A
PCPG	TCGA-RW-A68G-01A	B
PCPG	TCGA-WB-A81H-01A	B
PCPG	TCGA-P7-A5NY-01A	A
PCPG	TCGA-QR-A706-01A	A
PCPG	TCGA-SR-A6MV-01A	A
PCPG	TCGA-WB-A81T-01A	B
PCPG	TCGA-WB-A80M-01A	A
PCPG	TCGA-SP-A6QG-01A	A
PCPG	TCGA-S7-A7WL-01A	A
PCPG	TCGA-WB-A81V-01A	A
PCPG	TCGA-WB-A816-01A	B
PCPG	TCGA-S7-A7WN-01A	A
PCPG	TCGA-W2-A7HD-01A	A
PCPG	TCGA-RX-A8JQ-01A	A
PCPG	TCGA-QR-A70W-01A	A

PCPG	TCGA-QT-A5XP-01A	A
PCPG	TCGA-WB-A81G-01A	A
PCPG	TCGA-SR-A6MZ-01A	B
PCPG	TCGA-QR-A70H-01A	A
PCPG	TCGA-QR-A703-01A	A
PCPG	TCGA-S7-A7WV-01A	B
PCPG	TCGA-RW-A68A-01A	A
PCPG	TCGA-S7-A7WR-01A	A
PCPG	TCGA-RW-A68B-01A	A
PCPG	TCGA-WB-A80P-01A	A
PCPG	TCGA-W2-A7HA-01B	A
PCPG	TCGA-WB-A81F-01A	A
PCPG	TCGA-RW-A686-01A	A
PCPG	TCGA-QT-A5XN-01A	A
PCPG	TCGA-W2-A7HB-01A	A
PCPG	TCGA-S7-A7WW-01A	B
PCPG	TCGA-RW-A685-01A	B
PCPG	TCGA-QR-A6H5-01A	A
PCPG	TCGA-QR-A6H3-01A	A
PCPG	TCGA-QR-A6GY-01A	A
PCPG	TCGA-S7-A7X1-01A	A
PCPG	TCGA-WB-A819-01A	A
PCPG	TCGA-RW-A68F-01A	A
PCPG	TCGA-S7-A7X2-01A	A
PCPG	TCGA-W2-A7HC-01A	A
PCPG	TCGA-RW-A68C-01A	A
PCPG	TCGA-RW-A686-06A	A
PCPG	TCGA-QR-A6H4-01A	A
PCPG	TCGA-WB-A81D-01A	A
PCPG	TCGA-QT-A69Q-01A	A
PCPG	TCGA-RW-A688-01A	A
PCPG	TCGA-WB-A81P-01A	A
PCPG	TCGA-SR-A6MQ-01A	A
PCPG	TCGA-QR-A6GT-01A	A
PCPG	TCGA-SQ-A6I6-01A	A
PCPG	TCGA-QT-A5XL-01A	A
PCPG	TCGA-RW-A67W-01A	A
PCPG	TCGA-QR-A70D-01A	A
PCPG	TCGA-QR-A702-01A	A
PCPG	TCGA-WB-A81E-01A	A
PCPG	TCGA-QT-A7U0-01A	A
PCPG	TCGA-QR-A6GS-01A	A
PCPG	TCGA-RW-A67X-01A	A
PCPG	TCGA-TT-A6YK-01A	A
PCPG	TCGA-SQ-A6I4-01A	A
PCPG	TCGA-QR-A70T-01A	A
PCPG	TCGA-S7-A7WT-01A	A

PCPG	TCGA-S7-A7X0-01A	A
PCPG	TCGA-WB-A815-01A	A
PCPG	TCGA-QT-A5XK-01A	A
PCPG	TCGA-QR-A6GZ-05A	A
PCPG	TCGA-P8-A5KC-01A	A
PCPG	TCGA-WB-A81A-01A	A
PCPG	TCGA-RM-A68W-01A	A
PCPG	TCGA-SR-A6MY-01A	A
PCPG	TCGA-QR-A70U-01A	A
PCPG	TCGA-W2-A7H5-01B	A
PCPG	TCGA-WB-A80Q-01A	A
PCPG	TCGA-RW-A7CZ-01A	A
PCPG	TCGA-RT-A6YA-01A	A
PCPG	TCGA-SR-A6MX-06A	A
PCPG	TCGA-RW-A7D0-01A	A
PCPG	TCGA-WB-A81Q-01A	A
PCPG	TCGA-WB-A80O-01A	A
PCPG	TCGA-QR-A70G-01B	B
PCPG	TCGA-SP-A6QC-01A	A
PCPG	TCGA-WB-A81J-01A	A
PCPG	TCGA-QR-A6GX-01A	A
PCPG	TCGA-P8-A5KD-01A	A
PCPG	TCGA-QR-A6GW-01A	A
PCPG	TCGA-SR-A6MX-01A	A
PCPG	TCGA-SP-A6QJ-01A	A
PCPG	TCGA-RT-A6YC-01A	A
PCPG	TCGA-WB-A821-01A	A
PCPG	TCGA-QR-A700-01A	A
PCPG	TCGA-QR-A70V-01A	A
PCPG	TCGA-RW-A681-01A	A
PCPG	TCGA-WB-A81K-01A	B
PCPG	TCGA-RW-A67V-01A	A
PCPG	TCGA-QT-A5XJ-01A	A
PCPG	TCGA-QR-A70A-01A	A
PCPG	TCGA-QR-A70P-01A	A
PCPG	TCGA-TT-A6YO-01A	B
PCPG	TCGA-QR-A70O-01A	A
PCPG	TCGA-XG-A823-01A	B
PCPG	TCGA-WB-A80K-01A	A
PCPG	TCGA-SR-A6MT-01A	A
PCPG	TCGA-RW-A67Y-01A	A
PCPG	TCGA-S7-A7WU-01A	A
PCPG	TCGA-RW-A68D-01A	A
PCPG	TCGA-P8-A6RX-01A	A
PCPG	TCGA-PR-A5PF-01A	A
PCPG	TCGA-QR-A6H0-01A	A
PCPG	TCGA-W2-A7UY-01A	A

PCPG	TCGA-RW-A680-01A	A
PCPG	TCGA-QT-A5XO-01A	A
PCPG	TCGA-P8-A6RY-01A	A
PCPG	TCGA-S7-A7WQ-01A	A
PCPG	TCGA-QR-A70E-01A	A
PCPG	TCGA-PR-A5PH-01A	A
PCPG	TCGA-QR-A70J-01A	A
PCPG	TCGA-WB-A814-01A	A
PCPG	TCGA-TT-A6YN-01A	A
PCPG	TCGA-WB-A81S-01A	A
PCPG	TCGA-WB-A81I-01A	A
PCPG	TCGA-S7-A7WP-01A	A
PCPG	TCGA-QR-A6GR-01A	A
PCPG	TCGA-QR-A6GU-01A	A
PCPG	TCGA-W2-A7HE-01A	B
PCPG	TCGA-WB-A81M-01A	A
PCPG	TCGA-QR-A6H1-01A	A
PCPG	TCGA-SP-A6QD-01A	A
PCPG	TCGA-P7-A5NY-05A	A
PCPG	TCGA-RW-A689-01A	A
PCPG	TCGA-WB-A817-01A	B
PCPG	TCGA-QR-A70Q-01A	A
PCPG	TCGA-SA-A6C2-01A	A
PCPG	TCGA-S7-A7WM-01A	A
PCPG	TCGA-QR-A70K-01A	A
PCPG	TCGA-S7-A7WO-01A	B
PCPG	TCGA-WB-A80L-01A	A
PCPG	TCGA-RW-A684-01A	A
PCPG	TCGA-SR-A6MP-01A	A
PCPG	TCGA-QR-A7IN-01A	A
PCPG	TCGA-WB-A80N-01A	A
PCPG	TCGA-WB-A81R-01A	A
SARC	TCGA-KD-A5QU-01A	B
SARC	TCGA-HS-A5N8-01A	A
SARC	TCGA-K1-A42X-02A	B
SARC	TCGA-DX-A8BL-01A	B
SARC	TCGA-SI-A71P-01A	B
SARC	TCGA-WK-A8XS-01A	B
SARC	TCGA-DX-AB2Z-01A	B
SARC	TCGA-SI-AA8B-01A	B
SARC	TCGA-3B-A9HU-01A	B
SARC	TCGA-DX-A6YS-01A	B
SARC	TCGA-DX-AB2H-01A	A
SARC	TCGA-X6-A8C5-01A	B
SARC	TCGA-HB-A43Z-01A	B
SARC	TCGA-3B-A9HQ-01A	B
SARC	TCGA-DX-A6YU-01A	B

SARC	TCGA-DX-A3UE-01A	B
SARC	TCGA-DX-A3M1-01A	B
SARC	TCGA-DX-AB2P-01A	B
SARC	TCGA-DX-A48J-01A	B
SARC	TCGA-DX-A7EI-01A	A
SARC	TCGA-DX-A6YX-01A	B
SARC	TCGA-DX-A6B7-01A	B
SARC	TCGA-FX-A3TO-01A	B
SARC	TCGA-DX-A8BK-01A	B
SARC	TCGA-DX-AB2S-01A	B
SARC	TCGA-FX-A8OO-01A	A
SARC	TCGA-DX-A1L0-01A	B
SARC	TCGA-3B-A9HV-01A	B
SARC	TCGA-X9-A973-01A	B
SARC	TCGA-DX-A6YR-01A	B
SARC	TCGA-QQ-A5VC-01A	B
SARC	TCGA-DX-A3UA-01A	B
SARC	TCGA-PT-A8TR-01A	A
SARC	TCGA-3B-A9HZ-01A	B
SARC	TCGA-QQ-A8VF-01A	B
SARC	TCGA-FX-A3NJ-01A	B
SARC	TCGA-K1-A3PO-01A	B
SARC	TCGA-IS-A3K8-01A	B
SARC	TCGA-DX-A1L2-01A	B
SARC	TCGA-DX-A7EN-01A	B
SARC	TCGA-X6-A8C3-01A	B
SARC	TCGA-X2-A95T-01A	B
SARC	TCGA-X6-A7WA-01A	B
SARC	TCGA-DX-A6YZ-01A	B
SARC	TCGA-SG-A6Z7-01A	A
SARC	TCGA-DX-A6Z0-01A	B
SARC	TCGA-DX-A6Z2-01A	B
SARC	TCGA-K1-A3PN-02A	B
SARC	TCGA-DX-AB2L-01A	B
SARC	TCGA-WK-A8Y0-01A	B
SARC	TCGA-DX-A23Z-01A	B
SARC	TCGA-3B-A9I0-01A	B
SARC	TCGA-DX-A1KZ-01A	B
SARC	TCGA-HB-A3YV-01A	A
SARC	TCGA-DX-A8BO-01A	B
SARC	TCGA-MJ-A68H-01A	B
SARC	TCGA-DX-AB37-01A	B
SARC	TCGA-DX-A7EF-01A	B
SARC	TCGA-DX-A7ES-01A	B
SARC	TCGA-SI-AA8C-01A	A
SARC	TCGA-3R-A8YX-01A	B
SARC	TCGA-WK-A8XO-01A	B

SARC	TCGA-PC-A5DP-01A	B
SARC	TCGA-WK-A8XX-01A	B
SARC	TCGA-QQ-A8VH-01A	A
SARC	TCGA-DX-A48R-01A	B
SARC	TCGA-FX-A3NK-01A	B
SARC	TCGA-DX-AB36-01A	B
SARC	TCGA-3B-A9I1-01A	B
SARC	TCGA-IW-A3M5-01A	A
SARC	TCGA-UE-A6QT-01A	B
SARC	TCGA-KD-A5QT-01A	B
SARC	TCGA-DX-AB3A-01A	B
SARC	TCGA-Z4-AAPG-01A	B
SARC	TCGA-DX-A3U6-01A	B
SARC	TCGA-MB-A5YA-01A	B
SARC	TCGA-QQ-A5VD-01A	B
SARC	TCGA-SG-A849-01A	B
SARC	TCGA-IE-A4EH-01A	B
SARC	TCGA-QC-AA9N-01A	B
SARC	TCGA-3B-A9HO-01A	B
SARC	TCGA-DX-A7EM-01A	B
SARC	TCGA-DX-AB2Q-01A	B
SARC	TCGA-IE-A4EK-01A	B
SARC	TCGA-DX-AB2V-01A	B
SARC	TCGA-DX-A7EU-01A	B
SARC	TCGA-DX-A1KX-01A	B
SARC	TCGA-DX-A23R-01A	A
SARC	TCGA-3B-A9HI-01A	B
SARC	TCGA-WK-A8XY-01A	B
SARC	TCGA-3B-A9HP-01A	B
SARC	TCGA-DX-A1L1-01A	B
SARC	TCGA-MB-A5Y9-01A	B
SARC	TCGA-DX-A8BN-01A	B
SARC	TCGA-K1-A6RT-01A	B
SARC	TCGA-DX-AB2X-01A	B
SARC	TCGA-QQ-A8VG-01A	B
SARC	TCGA-IF-A4AK-01A	B
SARC	TCGA-WP-A9GB-01A	B
SARC	TCGA-MO-A47P-01A	B
SARC	TCGA-3B-A9HS-01A	B
SARC	TCGA-IE-A3OV-01A	B
SARC	TCGA-DX-A1L3-01A	B
SARC	TCGA-DX-A23T-01A	B
SARC	TCGA-3B-A9HY-01A	B
SARC	TCGA-DX-A3LY-01B	B
SARC	TCGA-LI-A9QH-01A	B
SARC	TCGA-DX-A48N-01A	B
SARC	TCGA-DX-AB32-01A	B

SARC	TCGA-DX-A6B8-01A	B
SARC	TCGA-3B-A9HT-01A	B
SARC	TCGA-DX-A8BQ-01A	B
SARC	TCGA-DX-A23Y-01A	B
SARC	TCGA-DX-AB2G-01A	B
SARC	TCGA-VT-AB3D-01A	B
SARC	TCGA-DX-A8BT-01A	B
SARC	TCGA-DX-A8BJ-01A	B
SARC	TCGA-DX-A3UC-01A	B
SARC	TCGA-MO-A47R-01A	B
SARC	TCGA-QQ-A8VB-01A	A
SARC	TCGA-K1-A42W-01A	B
SARC	TCGA-DX-A8BX-01A	B
SARC	TCGA-VT-A80J-02A	B
SARC	TCGA-PC-A5DM-01A	B
SARC	TCGA-IS-A3KA-01A	B
SARC	TCGA-K1-A3PN-01A	B
SARC	TCGA-X6-A8C7-01A	A
SARC	TCGA-DX-A6YT-01A	B
SARC	TCGA-DX-A3LU-01A	B
SARC	TCGA-DX-A1KW-01A	B
SARC	TCGA-SG-A6Z4-01A	B
SARC	TCGA-DX-A23U-01A	B
SARC	TCGA-WK-A8XZ-01A	B
SARC	TCGA-IS-A3K7-01A	B
SARC	TCGA-DX-A8BH-01A	B
SARC	TCGA-DX-A2J0-01A	B
SARC	TCGA-FX-A48G-01A	B
SARC	TCGA-PC-A5DL-01A	B
SARC	TCGA-3B-A9HX-01A	B
SARC	TCGA-HS-A5N7-01A	B
SARC	TCGA-PC-A5DO-01A	B
SARC	TCGA-MJ-A68J-01A	B
SARC	TCGA-IF-A3RQ-01A	B
SARC	TCGA-IS-A3K6-01A	B
SARC	TCGA-DX-A8BS-01A	B
SARC	TCGA-X6-A7WB-01A	B
SARC	TCGA-IE-A4EJ-01A	B
SARC	TCGA-DX-A1KU-01A	B
SARC	TCGA-HS-A5N9-01A	B
SARC	TCGA-QQ-A8VD-01A	B
SARC	TCGA-DX-A8BP-01A	B
SARC	TCGA-DX-A6YV-01A	B
SARC	TCGA-WK-A8XQ-01A	B
SARC	TCGA-DX-A3LT-01A	B
SARC	TCGA-DX-A3M2-01A	B
SARC	TCGA-DX-AB2T-01A	B

SARC	TCGA-VT-A80J-01A	B
SARC	TCGA-X6-A8C6-01A	B
SARC	TCGA-X6-A7WC-01A	B
SARC	TCGA-DX-A8BR-01A	B
SARC	TCGA-DX-A48K-01A	B
SARC	TCGA-SI-A71Q-01A	A
SARC	TCGA-SI-A71O-06A	B
SARC	TCGA-VT-A80G-01A	B
SARC	TCGA-DX-A240-01A	B
SARC	TCGA-K1-A42X-01A	B
SARC	TCGA-3B-A9HJ-01A	B
SARC	TCGA-QQ-A5VA-01A	B
SARC	TCGA-JV-A5VF-01A	A
SARC	TCGA-DX-AB2F-01A	B
SARC	TCGA-DX-AB35-01A	B
SARC	TCGA-KF-A41W-01A	B
SARC	TCGA-DX-A48P-01A	B
SARC	TCGA-3B-A9I3-01A	B
SARC	TCGA-DX-A7EL-01A	B
SARC	TCGA-DX-A6YQ-01A	B
SARC	TCGA-3B-A9HR-01A	B
SARC	TCGA-DX-A2J4-01A	B
SARC	TCGA-Z4-AAPF-01A	A
SARC	TCGA-HB-A3L4-01A	B
SARC	TCGA-FX-A3RE-01A	B
SARC	TCGA-N1-A6IA-01A	B
SARC	TCGA-HB-A2OT-01A	B
SARC	TCGA-DX-AB30-01A	A
SARC	TCGA-X6-A7W8-01A	B
SARC	TCGA-DX-A3U5-01A	B
SARC	TCGA-DX-A3UD-01A	B
SARC	TCGA-SI-A71O-01A	B
SARC	TCGA-UE-A6QU-01A	B
SARC	TCGA-QQ-A5VB-01A	B
SARC	TCGA-DX-A8BU-01A	B
SARC	TCGA-3B-A9HL-01A	B
SARC	TCGA-IW-A3M4-01A	B
SARC	TCGA-LI-A67I-01A	B
SARC	TCGA-IE-A4EI-01A	B
SARC	TCGA-DX-A7ER-01A	A
SARC	TCGA-DX-A3UF-01A	B
SARC	TCGA-DX-A3U9-01A	B
SARC	TCGA-X9-A971-01A	B
SARC	TCGA-DX-AB2E-01A	B
SARC	TCGA-DX-AB2W-01A	B
SARC	TCGA-DX-A7ET-01A	B
SARC	TCGA-PC-A5DN-01A	A

SARC	TCGA-DX-A8BV-01A	B
SARC	TCGA-X6-A8C2-01A	B
SARC	TCGA-DX-A8BG-01A	B
SARC	TCGA-DX-A3LS-01A	B
SARC	TCGA-JV-A5VE-01A	B
SARC	TCGA-X6-A7WD-01A	B
SARC	TCGA-Z4-A9VC-01A	B
SARC	TCGA-DX-A1KY-01A	A
SARC	TCGA-JV-A75J-01A	A
SARC	TCGA-HB-A5W3-01A	B
SARC	TCGA-DX-AB2J-01A	A
SARC	TCGA-FX-A2QS-01A	B
SARC	TCGA-Z4-A8JB-01A	B
SARC	TCGA-DX-A23V-01A	B
SARC	TCGA-DX-A3U7-01A	B
SARC	TCGA-DX-A6BG-01A	B
SARC	TCGA-WK-A8XT-01A	A
SARC	TCGA-MJ-A850-01A	A
SARC	TCGA-DX-A48O-01A	B
SARC	TCGA-DX-A2IZ-01A	A
SARC	TCGA-FX-A76Y-01A	B
SARC	TCGA-KD-A5QS-01A	B
SARC	TCGA-X6-A8C4-01A	B
SARC	TCGA-DX-A3LW-01A	B
SARC	TCGA-MB-A8JK-01A	B
SARC	TCGA-QC-A6FX-01A	B
SARC	TCGA-IW-A3M6-01A	B
SARC	TCGA-DX-AB2O-01A	B
SARC	TCGA-QC-A7B5-01A	B
SARC	TCGA-DX-A6BB-01A	B
SARC	TCGA-RN-A68Q-01A	B
SARC	TCGA-DX-A7EO-01A	A
SARC	TCGA-K1-A6RV-01A	B
SARC	TCGA-DX-A8BZ-01A	B
SARC	TCGA-IE-A6BZ-01A	B
SARC	TCGA-DX-A48L-01A	B
SARC	TCGA-IF-A4AJ-01A	B
SARC	TCGA-DX-A1L4-01A	B
SARC	TCGA-MB-A8JL-01A	B
SARC	TCGA-RN-AAAQ-01A	B
SARC	TCGA-DX-AATS-01A	B
SARC	TCGA-DX-A48U-01A	B
SARC	TCGA-DX-A8BM-01A	B
SARC	TCGA-PC-A5DK-01A	B
SARC	TCGA-HS-A5NA-01A	B
SARC	TCGA-K1-A6RU-01A	B
SARC	TCGA-DX-A6B9-01A	B

SARC	TCGA-DX-A6BF-01A	B
SARC	TCGA-DX-A6BE-01A	B
SARC	TCGA-DX-A3U8-01A	B
SARC	TCGA-MB-A5Y8-01A	B
SARC	TCGA-DX-A6BH-01A	B
SARC	TCGA-DX-A7EQ-01A	A
SARC	TCGA-QQ-A5V9-01A	B
SARC	TCGA-DX-AB3C-01A	A
SARC	TCGA-DX-A3UB-01A	B
SARC	TCGA-DX-A6BA-01A	B
SARC	TCGA-QQ-A5V2-01A	B
SARC	TCGA-DX-AB3B-01A	A
SARC	TCGA-DX-A2J1-01A	B
SKCM	TCGA-YG-AA3N-01A	A
SKCM	TCGA-ER-A193-06A	B
SKCM	TCGA-ER-A19A-06A	A
SKCM	TCGA-FS-A4FC-06A	A
SKCM	TCGA-EE-A2MN-06A	B
SKCM	TCGA-GN-A4U9-06A	B
SKCM	TCGA-EB-A5UN-06A	B
SKCM	TCGA-D9-A1JX-06A	B
SKCM	TCGA-WE-A8ZX-06A	B
SKCM	TCGA-BF-A1PX-01A	A
SKCM	TCGA-RP-A694-06A	B
SKCM	TCGA-EE-A2ML-06A	B
SKCM	TCGA-FS-A1ZR-06A	B
SKCM	TCGA-DA-A3F5-06A	B
SKCM	TCGA-D3-A5GR-06A	B
SKCM	TCGA-EE-A29A-06A	B
SKCM	TCGA-EB-A3XC-01A	A
SKCM	TCGA-FS-A1ZY-06A	B
SKCM	TCGA-EB-A4XL-01A	A
SKCM	TCGA-3N-A9WD-06A	B
SKCM	TCGA-D3-A51N-06A	B
SKCM	TCGA-EE-A2MT-06A	B
SKCM	TCGA-WE-A8ZM-06A	B
SKCM	TCGA-D3-A51J-06A	B
SKCM	TCGA-QB-AA9O-06A	B
SKCM	TCGA-FR-A2OS-01A	B
SKCM	TCGA-EE-A2GJ-06A	B
SKCM	TCGA-D3-A3C8-06A	B
SKCM	TCGA-LH-A9QB-06A	B
SKCM	TCGA-EE-A3AB-06A	B
SKCM	TCGA-EE-A2M5-06A	B
SKCM	TCGA-EB-A4OZ-01A	A
SKCM	TCGA-D3-A3BZ-06A	B
SKCM	TCGA-EE-A2MK-06A	B

SKCM	TCGA-FS-A4F5-06A	B
SKCM	TCGA-YG-AA3P-06A	B
SKCM	TCGA-GF-A6C8-06A	B
SKCM	TCGA-BF-A3DN-01A	B
SKCM	TCGA-YD-A89C-06A	B
SKCM	TCGA-D3-A5GO-06A	B
SKCM	TCGA-D9-A3Z3-06A	B
SKCM	TCGA-W3-AA1W-06A	B
SKCM	TCGA-EE-A2GL-06A	B
SKCM	TCGA-D3-A8GM-06A	B
SKCM	TCGA-BF-AAP6-01A	B
SKCM	TCGA-ER-A19E-06A	B
SKCM	TCGA-EE-A29H-06A	B
SKCM	TCGA-EE-A2MQ-06A	B
SKCM	TCGA-EE-A2A0-06A	B
SKCM	TCGA-EE-A20H-06A	B
SKCM	TCGA-EE-A2MP-06A	B
SKCM	TCGA-D3-A3C1-06A	B
SKCM	TCGA-ER-A194-01A	B
SKCM	TCGA-BF-AAOU-01A	B
SKCM	TCGA-D3-A2JA-06A	B
SKCM	TCGA-EE-A3AF-06A	B
SKCM	TCGA-XV-A9W5-01A	A
SKCM	TCGA-EB-A3XD-01A	A
SKCM	TCGA-ER-A2NH-06A	B
SKCM	TCGA-QB-A6FS-06A	B
SKCM	TCGA-GN-A266-06A	B
SKCM	TCGA-ER-A2ND-06A	B
SKCM	TCGA-W3-AA1Q-06A	B
SKCM	TCGA-EE-A2GC-06A	B
SKCM	TCGA-D3-A1QA-07A	B
SKCM	TCGA-BF-A3DL-01A	B
SKCM	TCGA-EE-A2MG-06A	B
SKCM	TCGA-EB-A5KH-06A	B
SKCM	TCGA-EE-A29P-06A	B
SKCM	TCGA-GN-A4U4-06A	B
SKCM	TCGA-D3-A2JE-06A	B
SKCM	TCGA-D3-A3C7-06A	B
SKCM	TCGA-FS-A4F4-06A	B
SKCM	TCGA-EE-A3J4-06A	B
SKCM	TCGA-D3-A51E-06A	B
SKCM	TCGA-D3-A5GT-01A	B
SKCM	TCGA-RP-A693-06A	B
SKCM	TCGA-D9-A149-06A	B
SKCM	TCGA-GN-A9SD-06A	B
SKCM	TCGA-EB-A6R0-01A	B
SKCM	TCGA-FW-A3R5-06A	B

SKCM	TCGA-W3-A825-06A	B
SKCM	TCGA-EB-A3XE-01A	A
SKCM	TCGA-ER-A2NF-06A	B
SKCM	TCGA-FR-A8YE-06A	B
SKCM	TCGA-XV-AAZV-01A	A
SKCM	TCGA-BF-AAP4-01A	B
SKCM	TCGA-IH-A3EA-01A	B
SKCM	TCGA-EE-A3AA-06A	B
SKCM	TCGA-GN-A263-01A	B
SKCM	TCGA-EB-A299-01A	A
SKCM	TCGA-GN-A26D-06A	B
SKCM	TCGA-D3-A2J7-06A	B
SKCM	TCGA-EB-A82C-01A	A
SKCM	TCGA-FS-A1ZD-06A	B
SKCM	TCGA-D9-A4Z6-06A	B
SKCM	TCGA-FS-A1ZH-06A	B
SKCM	TCGA-D3-A51F-06A	B
SKCM	TCGA-BF-AAP2-01A	A
SKCM	TCGA-BF-A9VF-01A	A
SKCM	TCGA-W3-AA1O-06A	B
SKCM	TCGA-EE-A20B-06A	B
SKCM	TCGA-D3-A8GC-06A	B
SKCM	TCGA-GN-A4U7-06A	B
SKCM	TCGA-EE-A2GI-06A	B
SKCM	TCGA-RP-A695-06A	B
SKCM	TCGA-DA-A95V-06A	B
SKCM	TCGA-GN-A8LN-01A	B
SKCM	TCGA-ER-A19D-06A	B
SKCM	TCGA-BF-A1PV-01A	B
SKCM	TCGA-ER-A19H-06A	B
SKCM	TCGA-W3-A828-06A	B
SKCM	TCGA-D9-A6EG-06A	B
SKCM	TCGA-EB-A82B-01A	B
SKCM	TCGA-EB-A5UM-01A	B
SKCM	TCGA-EB-A1NK-01A	A
SKCM	TCGA-FS-A1ZQ-06A	B
SKCM	TCGA-DA-A95Y-06A	B
SKCM	TCGA-EE-A29W-06A	B
SKCM	TCGA-FS-A1ZW-06A	B
SKCM	TCGA-DA-A960-01A	B
SKCM	TCGA-D3-A3MO-06A	B
SKCM	TCGA-EE-A3J8-06A	B
SKCM	TCGA-D9-A1JW-06A	B
SKCM	TCGA-EE-A3AG-06A	B
SKCM	TCGA-EE-A2A6-06A	B
SKCM	TCGA-EB-A4IS-01A	B
SKCM	TCGA-GN-A26A-06A	B

SKCM	TCGA-ER-A19M-06A	B
SKCM	TCGA-EE-A183-06A	B
SKCM	TCGA-EB-A4OY-01A	B
SKCM	TCGA-GN-A267-06A	B
SKCM	TCGA-FS-A4FB-06A	B
SKCM	TCGA-GF-A3OT-06A	B
SKCM	TCGA-EE-A2GB-06A	B
SKCM	TCGA-D9-A148-06A	B
SKCM	TCGA-EE-A2MH-06A	B
SKCM	TCGA-FS-A4F9-06A	B
SKCM	TCGA-FS-A1Z3-06A	B
SKCM	TCGA-D3-A51T-06A	B
SKCM	TCGA-EB-A57M-01A	A
SKCM	TCGA-D3-A51G-06A	B
SKCM	TCGA-EE-A3JI-06A	B
SKCM	TCGA-D3-A3C3-06A	B
SKCM	TCGA-EE-A2GK-06A	B
SKCM	TCGA-Z2-AA3S-06A	B
SKCM	TCGA-BF-A1PU-01A	B
SKCM	TCGA-ER-A19S-06A	B
SKCM	TCGA-EB-A6QZ-01A	B
SKCM	TCGA-EB-A550-01A	A
SKCM	TCGA-EE-A2GR-06A	B
SKCM	TCGA-FS-A1YW-06A	B
SKCM	TCGA-D3-A8GK-06A	B
SKCM	TCGA-FS-A1ZU-06A	B
SKCM	TCGA-EE-A3AH-06A	B
SKCM	TCGA-D9-A4Z3-01A	B
SKCM	TCGA-EB-A3HV-01A	A
SKCM	TCGA-XV-AAZY-01A	B
SKCM	TCGA-D3-A1Q3-06A	B
SKCM	TCGA-ER-A195-06A	B
SKCM	TCGA-EE-A29S-06A	B
SKCM	TCGA-EE-A2GM-06B	B
SKCM	TCGA-EE-A3JE-06A	B
SKCM	TCGA-EE-A2GN-06A	B
SKCM	TCGA-D3-A1Q6-06A	B
SKCM	TCGA-D3-A8GN-06A	B
SKCM	TCGA-ER-A3ET-06A	B
SKCM	TCGA-WE-A8K6-06A	B
SKCM	TCGA-ER-A198-06A	B
SKCM	TCGA-XV-AAZW-01A	A
SKCM	TCGA-D9-A4Z5-01A	B
SKCM	TCGA-ER-A19L-06A	B
SKCM	TCGA-ER-A42H-01A	B
SKCM	TCGA-WE-A8JZ-06A	B
SKCM	TCGA-EB-A5SF-01A	A

SKCM	TCGA-EE-A2GS-06A	B
SKCM	TCGA-ER-A3ES-06A	B
SKCM	TCGA-EB-A5VV-06A	B
SKCM	TCGA-ER-A19T-06A	B
SKCM	TCGA-HR-A2OG-06A	B
SKCM	TCGA-D3-A1QA-06A	B
SKCM	TCGA-EE-A2MU-06A	B
SKCM	TCGA-EB-A97M-01A	B
SKCM	TCGA-EB-A553-01A	B
SKCM	TCGA-XV-A9VZ-01A	A
SKCM	TCGA-D9-A3Z4-01A	B
SKCM	TCGA-Z2-A8RT-06A	B
SKCM	TCGA-3N-A9WB-06A	A
SKCM	TCGA-D9-A4Z2-01A	B
SKCM	TCGA-EB-A44P-01A	B
SKCM	TCGA-ER-A19G-06A	B
SKCM	TCGA-D3-A2JC-06A	B
SKCM	TCGA-EB-A44O-01A	A
SKCM	TCGA-EE-A29E-06A	B
SKCM	TCGA-FR-A44A-06A	B
SKCM	TCGA-ER-A42L-06A	B
SKCM	TCGA-DA-A95W-06A	B
SKCM	TCGA-ER-A19K-01A	A
SKCM	TCGA-ER-A3EV-06A	B
SKCM	TCGA-FS-A1YX-06A	B
SKCM	TCGA-EB-A3XF-01A	A
SKCM	TCGA-EE-A2GE-06A	B
SKCM	TCGA-WE-A8ZO-06A	B
SKCM	TCGA-EE-A29N-06A	B
SKCM	TCGA-D3-A3MU-06A	B
SKCM	TCGA-EE-A3JH-06A	B
SKCM	TCGA-WE-AA9Y-06A	B
SKCM	TCGA-D3-A3ML-06A	B
SKCM	TCGA-ER-A19Q-06A	B
SKCM	TCGA-DA-A1IA-06A	B
SKCM	TCGA-ER-A19W-06A	B
SKCM	TCGA-BF-A5EQ-01A	B
SKCM	TCGA-FS-A1ZG-06A	B
SKCM	TCGA-D3-A1Q8-06A	B
SKCM	TCGA-ER-A1A1-06A	B
SKCM	TCGA-EB-A6L9-06A	B
SKCM	TCGA-FR-A7UA-06A	B
SKCM	TCGA-EE-A3J7-06A	B
SKCM	TCGA-EB-A24C-01A	A
SKCM	TCGA-GN-A4U5-01A	A
SKCM	TCGA-EE-A2MF-06A	B
SKCM	TCGA-EE-A2GU-06A	B

SKCM	TCGA-DA-A1IB-06A	B
SKCM	TCGA-FS-A4F8-06A	B
SKCM	TCGA-ER-A2NC-06A	B
SKCM	TCGA-EE-A3JA-06A	B
SKCM	TCGA-D9-A3Z1-06A	B
SKCM	TCGA-EB-A44R-06A	B
SKCM	TCGA-D3-A1Q4-06A	B
SKCM	TCGA-D3-A51K-06A	B
SKCM	TCGA-EE-A29T-06A	B
SKCM	TCGA-FS-A1ZS-06A	B
SKCM	TCGA-FW-A5DY-06A	B
SKCM	TCGA-BF-A3DJ-01A	A
SKCM	TCGA-D9-A6E9-06A	B
SKCM	TCGA-BF-AAP8-01A	B
SKCM	TCGA-EE-A29B-06A	B
SKCM	TCGA-EE-A3AD-06A	B
SKCM	TCGA-EE-A2GO-06A	B
SKCM	TCGA-GF-A6C9-06A	B
SKCM	TCGA-EB-A5SE-01A	A
SKCM	TCGA-YD-A9TA-06A	B
SKCM	TCGA-EE-A2MJ-06A	B
SKCM	TCGA-D3-A2J8-06A	B
SKCM	TCGA-GN-A4U8-06A	B
SKCM	TCGA-EE-A2MC-06A	B
SKCM	TCGA-FR-A728-01A	B
SKCM	TCGA-FS-A4FD-06A	B
SKCM	TCGA-EE-A2M8-06A	B
SKCM	TCGA-GN-A264-06A	B
SKCM	TCGA-FS-A1ZN-01A	A
SKCM	TCGA-D9-A6EA-06A	B
SKCM	TCGA-FS-A1ZE-06A	B
SKCM	TCGA-EB-A5VU-01A	A
SKCM	TCGA-D3-A51H-06A	B
SKCM	TCGA-ER-A197-06A	B
SKCM	TCGA-3N-A9WC-06A	B
SKCM	TCGA-GN-A8LL-06A	B
SKCM	TCGA-FS-A4F2-06A	B
SKCM	TCGA-EE-A3AC-06A	B
SKCM	TCGA-FS-A1ZT-06A	B
SKCM	TCGA-DA-A1I2-06A	B
SKCM	TCGA-WE-A8ZN-06A	B
SKCM	TCGA-EE-A17Z-06A	B
SKCM	TCGA-WE-A8ZY-06A	B
SKCM	TCGA-EE-A20F-06A	B
SKCM	TCGA-FS-A1YY-06A	B
SKCM	TCGA-RP-A690-06A	B
SKCM	TCGA-D3-A3MR-06A	B

SKCM	TCGA-FR-A726-01A	B
SKCM	TCGA-D3-A8GQ-06A	B
SKCM	TCGA-ER-A19T-01A	B
SKCM	TCGA-ER-A19O-06A	B
SKCM	TCGA-D3-A2JH-06A	B
SKCM	TCGA-EE-A2GD-06A	B
SKCM	TCGA-EE-A2MD-06A	B
SKCM	TCGA-EE-A2MS-06A	B
SKCM	TCGA-EB-A44N-01A	A
SKCM	TCGA-W3-A824-06A	B
SKCM	TCGA-EB-A41A-01A	B
SKCM	TCGA-EE-A2ME-06A	B
SKCM	TCGA-EE-A20C-06A	B
SKCM	TCGA-D3-A8GR-06A	B
SKCM	TCGA-FW-A3TU-06A	B
SKCM	TCGA-EE-A29D-06A	B
SKCM	TCGA-ER-A2NB-01A	A
SKCM	TCGA-D9-A6EC-06A	B
SKCM	TCGA-EE-A29M-06A	B
SKCM	TCGA-FR-A729-06A	B
SKCM	TCGA-FR-A69P-06A	B
SKCM	TCGA-FS-A4F0-06A	B
SKCM	TCGA-EB-A42Y-01A	B
SKCM	TCGA-BF-A5EP-01A	B
SKCM	TCGA-FR-A3YO-06A	B
SKCM	TCGA-FR-A8YD-06A	B
SKCM	TCGA-FR-A7U9-06A	B
SKCM	TCGA-GN-A4U3-06A	B
SKCM	TCGA-ER-A19J-06A	B
SKCM	TCGA-D3-A3CF-06A	B
SKCM	TCGA-GN-A265-06A	B
SKCM	TCGA-GN-A26C-01A	B
SKCM	TCGA-EB-A41B-01A	B
SKCM	TCGA-OD-A75X-06A	B
SKCM	TCGA-EE-A2M7-06A	B
SKCM	TCGA-GF-A769-01A	B
SKCM	TCGA-EB-A430-01A	B
SKCM	TCGA-W3-AA1R-06A	B
SKCM	TCGA-EB-A3Y7-01A	A
SKCM	TCGA-EB-A3Y6-01A	B
SKCM	TCGA-ER-A199-06A	B
SKCM	TCGA-FR-A7U8-06A	B
SKCM	TCGA-W3-AA21-06A	B
SKCM	TCGA-WE-AAA4-06A	B
SKCM	TCGA-EB-A24D-01A	B
SKCM	TCGA-D3-A1Q1-06A	B
SKCM	TCGA-D3-A2JO-06A	B

SKCM	TCGA-EE-A2MM-06A	B
SKCM	TCGA-ER-A19B-06A	B
SKCM	TCGA-DA-A1IC-06A	A
SKCM	TCGA-D3-A8GL-06A	B
SKCM	TCGA-FS-A1ZM-06A	B
SKCM	TCGA-D3-A2JG-06A	B
SKCM	TCGA-BF-AAOX-01A	A
SKCM	TCGA-D3-A3C6-06A	B
SKCM	TCGA-EE-A2GT-06A	B
SKCM	TCGA-FS-A1ZJ-06A	B
SKCM	TCGA-BF-A5EO-01A	A
SKCM	TCGA-EE-A20I-06A	B
SKCM	TCGA-BF-A1PZ-01A	B
SKCM	TCGA-EE-A2MI-06A	B
SKCM	TCGA-EB-A5SG-06A	B
SKCM	TCGA-FS-A1ZC-06A	B
SKCM	TCGA-D3-A1Q5-06A	B
SKCM	TCGA-ER-A19F-06A	B
SKCM	TCGA-WE-AAA3-06A	B
SKCM	TCGA-ER-A19P-06A	B
SKCM	TCGA-RP-A6K9-06A	B
SKCM	TCGA-BF-AAP0-06A	B
SKCM	TCGA-EB-A4P0-01A	B
SKCM	TCGA-D3-A2J9-06A	B
SKCM	TCGA-EB-A3XB-01A	B
SKCM	TCGA-GN-A8LK-06A	B
SKCM	TCGA-FR-A3R1-01A	B
SKCM	TCGA-BF-A3DM-01A	B
SKCM	TCGA-EE-A2MR-06A	B
SKCM	TCGA-FS-A1ZA-06A	B
SKCM	TCGA-D3-A2JK-06A	B
SKCM	TCGA-WE-A8ZR-06A	B
SKCM	TCGA-D3-A2J6-06A	B
SKCM	TCGA-EE-A185-06A	A
SKCM	TCGA-DA-A1HW-06A	B
SKCM	TCGA-D9-A1X3-06A	B
SKCM	TCGA-EB-A5UL-06A	B
SKCM	TCGA-D3-A8GD-06A	B
SKCM	TCGA-D3-A2JD-06A	B
SKCM	TCGA-EB-A5SH-06A	B
SKCM	TCGA-WE-AAA0-06A	B
SKCM	TCGA-WE-A8ZT-06A	B
SKCM	TCGA-EB-A5FP-01A	A
SKCM	TCGA-D3-A2JN-06A	B
SKCM	TCGA-EE-A29Q-06A	B
SKCM	TCGA-EB-A44Q-06A	B
SKCM	TCGA-EE-A3JD-06A	B

SKCM	TCGA-D3-A1Q9-06A	B
SKCM	TCGA-EE-A3JB-06A	B
SKCM	TCGA-EE-A182-06A	A
SKCM	TCGA-EE-A17X-06A	B
SKCM	TCGA-EE-A29L-06A	B
SKCM	TCGA-EE-A3J3-06A	B
SKCM	TCGA-EB-A85I-01A	B
SKCM	TCGA-DA-A95X-06A	B
SKCM	TCGA-FR-A3YN-06A	B
SKCM	TCGA-D3-A8GE-06A	B
SKCM	TCGA-D3-A1Q7-06A	B
SKCM	TCGA-WE-A8ZQ-06A	B
SKCM	TCGA-EB-A85J-01A	B
SKCM	TCGA-D3-A8GP-06A	B
SKCM	TCGA-EB-A42Z-01A	B
SKCM	TCGA-EB-A4IQ-01A	A
SKCM	TCGA-D3-A5GS-06A	B
SKCM	TCGA-DA-A1I7-06A	B
SKCM	TCGA-FS-A1ZP-06A	B
SKCM	TCGA-D3-A3CE-06A	B
SKCM	TCGA-ER-A3PL-06A	B
SKCM	TCGA-FS-A1Z0-06A	B
SKCM	TCGA-DA-A1HV-06A	B
SKCM	TCGA-EE-A2GH-06A	B
SKCM	TCGA-D3-A51R-06A	B
SKCM	TCGA-DA-A1I4-06A	B
SKCM	TCGA-GF-A2C7-01A	A
SKCM	TCGA-FW-A5DX-01A	B
SKCM	TCGA-WE-A8K1-06A	B
SKCM	TCGA-EE-A2A2-06A	B
SKCM	TCGA-EE-A2GP-06A	B
SKCM	TCGA-ER-A19N-06A	B
SKCM	TCGA-FS-A1Z4-06A	B
SKCM	TCGA-FS-A1ZB-06A	A
SKCM	TCGA-GN-A268-06A	B
SKCM	TCGA-FS-A1ZZ-06A	B
SKCM	TCGA-DA-A95Z-06A	B
SKCM	TCGA-EE-A29G-06A	A
SKCM	TCGA-EE-A2M6-06A	B
SKCM	TCGA-EE-A29X-06A	B
SKCM	TCGA-EE-A184-06A	B
SKCM	TCGA-BF-A1Q0-01A	B
SKCM	TCGA-EB-A6QY-01A	A
SKCM	TCGA-D3-A5GL-06A	B
SKCM	TCGA-DA-A3F2-06A	B
SKCM	TCGA-EE-A17Y-06A	B
SKCM	TCGA-D3-A2JL-06A	B

SKCM	TCGA-D3-A8GJ-06A	B
SKCM	TCGA-D3-A8GO-06A	B
SKCM	TCGA-EE-A2A1-06A	B
SKCM	TCGA-GF-A4EO-06A	B
SKCM	TCGA-ER-A2NG-06A	B
SKCM	TCGA-D3-A5GN-06A	B
SKCM	TCGA-D3-A3CC-06A	B
SKCM	TCGA-ER-A42K-06A	B
SKCM	TCGA-EE-A2A5-06A	B
SKCM	TCGA-D3-A8GS-06A	B
SKCM	TCGA-GN-A262-06A	B
SKCM	TCGA-EE-A181-06A	B
SKCM	TCGA-DA-A1HY-06A	B
SKCM	TCGA-BF-AAP7-01A	A
SKCM	TCGA-EE-A29R-06A	B
SKCM	TCGA-HR-A5NC-01A	A
SKCM	TCGA-EE-A29C-06A	B
SKCM	TCGA-EB-A551-01A	B
SKCM	TCGA-EB-A51B-01A	A
SKCM	TCGA-ER-A2NE-06A	B
SKCM	TCGA-D3-A2JP-06A	B
SKCM	TCGA-FR-A8YC-06A	B
SKCM	TCGA-D3-A5GU-06A	B
SKCM	TCGA-D3-A1QB-06A	B
SKCM	TCGA-YG-AA3O-06A	B
SKCM	TCGA-D3-A8GI-06A	B
SKCM	TCGA-DA-A1I0-06A	B
SKCM	TCGA-ER-A19C-06A	B
SKCM	TCGA-DA-A1I8-06A	B
SKCM	TCGA-ER-A196-01A	A
SKCM	TCGA-FS-A1ZK-06A	B
SKCM	TCGA-ER-A2NF-01A	B
SKCM	TCGA-EB-A431-01A	B
SKCM	TCGA-D3-A2JF-06A	B
SKCM	TCGA-DA-A3F8-06A	B
SKCM	TCGA-D3-A8GV-06A	B
SKCM	TCGA-FW-A3TV-06A	B
SKCM	TCGA-YD-A9TB-06A	B
SKCM	TCGA-Z2-AA3V-06A	B
SKCM	TCGA-BF-AAP1-01A	B
SKCM	TCGA-WE-A8K5-06A	B
SKCM	TCGA-W3-AA1V-06B	B
SKCM	TCGA-DA-A1I5-06A	B
SKCM	TCGA-D3-A2JB-06A	B
SKCM	TCGA-D3-A3MV-06A	B
SKCM	TCGA-DA-A1I1-06A	B
SKCM	TCGA-WE-A8K4-01A	B

SKCM	TCGA-BF-A5ES-01A	B
SKCM	TCGA-EE-A29V-06A	A
SKCM	TCGA-D3-A8GB-06A	B
SKCM	TCGA-EE-A180-06A	B
SKCM	TCGA-XV-A9W2-01A	A
SKCM	TCGA-FW-A3I3-06A	A
SKCM	TCGA-FS-A1ZF-06A	B
SKCM	TCGA-BF-A5ER-01A	A
SKCM	TCGA-D3-A3CB-06A	B
SKCM	TCGA-HR-A2OH-06A	B
SKCM	TCGA-DA-A3F3-06A	B
SKCM	TCGA-EE-A3J5-06A	B
SKCM	TCGA-XV-AB01-06A	B
SKCM	TCGA-FS-A1Z7-06A	B
